# Effective biomarkers and therapeutic targets of nerve-immunity interaction in the treatment of depression: an integrated investigation of the miRNA-mRNA regulatory networks

**DOI:** 10.18632/aging.204030

**Published:** 2022-04-25

**Authors:** Zixuan Wu, Zhixiang Cai, Hongshuo Shi, Xuyan Huang, Minjie Cai, Kai Yuan, Peidong Huang, Guoqi Shi, Tao Yan, Zhichao Li

**Affiliations:** 1 Guangzhou University of Chinese Medicine, Guangzhou 510006, Guangdong Province, China; 2 Shandong University of Traditional Chinese Medicine, Jinan 250355, Shandong Province, China; 3 The Second Affiliated Hospital of Shandong University of Traditional Chinese Medicine, Jinan 250355, Shandong Province, China; 4 Shantou Health School, Shantou 515061, Guangdong Province, China; 5 Yunnan University of Chinese Medicine, Kunming 650500, Yunnan Province, China; 6 Department of Cardiovascular Surgery, General Hospital of Southern Theater Command, PLA 510010, Guangdong Province, China

**Keywords:** major depressive disorder (MMD), microRNAs (miRNAs), DE-miRNAs, regulatory networks, bioinformatics

## Abstract

Background: Major depressive disorder (MDD) is an emotional condition that interferes with sufferers’ work and daily life. Numerous studies have found that miRNAs play a significant role in the development of MDD and can be utilized as a biomarker for its diagnosis and therapy. However, there have been few studies on nerve-immunity interaction treatment for the brains of MMD patients. Methods: The work is performed on microarray data. We analyzed the differences of miRNAs (GSE58105, GSE81152, GSE152267, and GSE182194) and mRNA (GSE19738, GSE32280, GSE44593, GSE53987, and GSE98793) in MDD and healthy samples from GEO datasets. FunRich was used to predict the transcription factors and target genes of the miRNAs, and TF and GO enrichment analyses were performed. Then, by comparing the differential expression of the anticipated target genes and five mRNAs, intersecting mRNAs were discovered. The intersecting genes were submitted to GO and KEGG analyses to determine their functions. These intersecting potential genes and pathways that linked to MDD in neurological and immunological aspects have been identified for future investigation. Results: We discovered five hub genes: KCND2, MYT1L, GJA1, CHL1, and SNAP25, which were all up-regulated genes. However, in MMD, the equivalent miRNAs, hsa-miR-206 and hsa-miR-338-3p, were both down-regulated. These miRNAs can activate or inhibit the T cell receptor signal pathway, JAK-STAT and other signal pathways, govern immune-inflammatory response, neuronal remodeling, and mediate the onset and development of MMD Conclusions: The results of a thorough bioinformatics investigation of miRNAs and mRNAs in MDD showed that miR-338-3P and miR-206 might be effective biomarkers and possible therapeutic targets for the treatment of MDD via nerve-immunity interaction.

## INTRODUCTION

Major depressive disorder (MDD) is a crippling mental illness characterized by depression and emotional disturbance brought on by a variety of psychological factors [1]. The high prevalence, recurrence, and disability rates are all difficult issues to address in MDD prevention and treatment [2]. Depression currently affects at least 20% of the world’s population, and the WHO predicts that depression will overtake heart disease as the leading disorder by 2030 [3]. The occurrence of MDD not only reduces patients’ quality of life, but also has a significant financial impact on the families and society. Hypotheses based on monoamine transmitter, immune-inflammation, the stress, neuroplasticity are now being researched [4]. In recent years, scholars have placed a greater emphasis on the study of the pathophysiology of immunity-inflammation and MDD [5]. According to various studies, the activation of the peripheral immune system is associated with MDD [6]. However, the pathobiology of MDD inflammation is poorly understood, and additional research is needed to identify the accurate diagnostic biomarkers. Chronic moderate stress, neuroinflammation, and immune response changes have all been connected to the genesis of MDD [7]. More and more studies have shown that the neurological system is inextricably linked to the immune system.

Chemical medications are currently used primarily in the clinical treatment of MMD in Western medicine, whereas Chinese materia medica, acupuncture and moxibustion are commonly used in traditional Chinese medicine (TCM). Traditional antidepressants, on the other hand, can only relieve a portion of MMD patients’ symptoms, and 20% to 30% of MMD patients do not respond to antidepressant medications. Neuroinflammation is thought to be one of the causes for ineffective treatment [8]. Following an injury, neuroinflammation occurs as a result of an imbalance between the synthesis and release of pro-inflammatory and anti-inflammatory cytokines from central or peripheral sources [9]. The most visible aspect of neuroinflammation is the activation of microglia. Microglia activation results in the synthesis of nutritional and anti-inflammatory chemicals under physiological conditions. Microglia shows excessive activation in pathological condition such as chronic stress or infection, resulting increased not only in levels of inflammatory chemicals in the brain, but also neuronal damage and death [10]. In order to effectively manage neuroinflammation and restore neurotropism and neurotransmitter function, it is critical for basic and clinical MMD research to identify specific diagnostic biomarkers from the perspective of nerve-immunity interaction.

Endogenous intracellular and extracellular small non-coding RNAs are known as microRNAs (MiRNAs). It may govern cell proliferation, division, apoptosis, and metabolism as regulators of numerous biological processes by promoting the degradation of mRNAs and inhibiting their translation, so that its targeted mRNAs play a negative feedback regulatory function [11]. Liguori discovered that combining microRNA and mRNA expression analysis in pediatric multiple sclerosis is an integrated approach to uncovering novel disease pathogenic mechanisms [12]. Many other studies have shown a close relationship between microRNAs and mRNAs in disease regulation, particularly the negative feedback regulation of targeted mRNAs by their miRNAs [13–14], which allows us to further investigate the mechanisms and functions of miRNAs in relation to MMD development. MiRNAs have been investigated as potential therapeutic targets for a variety of central nervous system diseases due to their involvement in nearly all fundamental cellular activities. Changes in miRNA expression are well known to be linked to the pathogenesis of numerous neurodegenerative diseases and hold significant therapeutic promise in the treatment of mood disorders, including clinical MDD [15]. Detecting the differences in pathogenic miRNA levels in the brain will be an excellent method for identifying people with early MMD. Bioinformatics is a critical tool for furthering biological understanding and therapeutic development [16].

**Figure 1:**
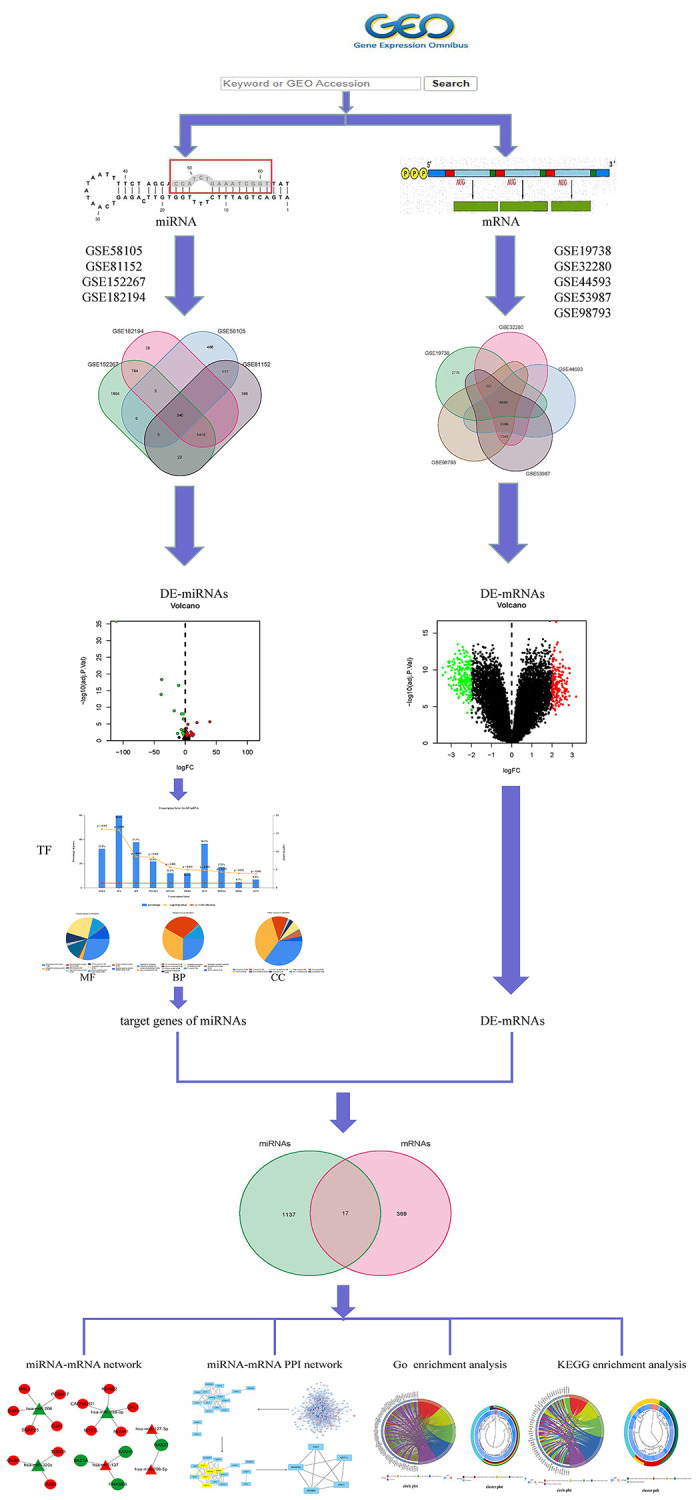
Framework based on an integration method for integrating miRNA-mRNA regulatory network analysis.

In MMD, it has been reported that miRNAs are not only involved in the pathogenesis of the disease [17], but are also associated with depression caused by various illnesses [18, 19], as well as the response to specific treatments such as isoliquiritin [20], oligonucleotides [21], or purinergic receptor [22], confirming their potential as disease monitoring biomarkers. So far, little miRNAs research has been done in the Nerve-immunity Interaction of Depression. Therefore, understanding the role of miRNA in the development of MMD may lead to the discovery of a biomarker that can be used as a neuroimmune interaction in the treatment of MDD. The work is performed on microarray data and Figure 1 show Framework.

## MATERIALS AND METHODS

### Data processing of DE-miRNAs

**Figure 2:**
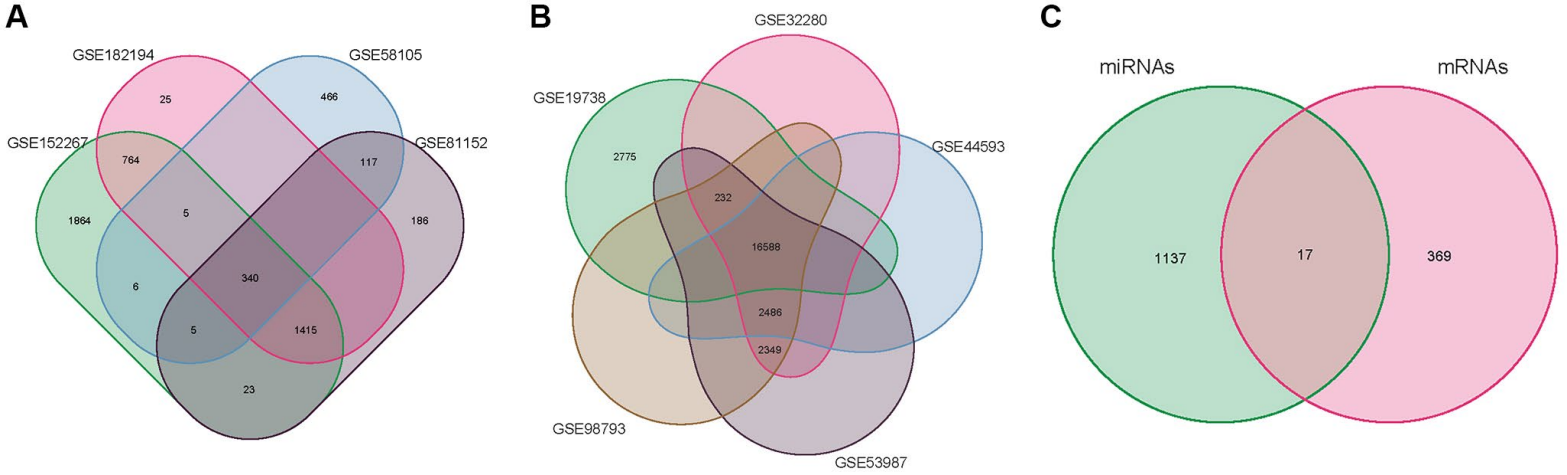
**Venn diagram.** (**A**) miRNA of the merger GSE58105, GSE81152, GSE152267 and GSE182194. (**B**) mRNA of the merger GSE19738, GSE32280, GSE44593, GSE53987, and GSE98793. (**C**) overlapping DE-mRNAs.

We selected datasets from the Gene Expression Omnibus (GEO). Strategy for searching (‘depression’ [MeSH] and miRNA [All Fields] and normal) AND (‘Homo sapiens’ [Organism] AND ‘Non-coding RNA profiling by array’ [Filter]). The following were the inclusion criteria: Plasma miRNA levels in MMD patients and healthy persons. Finally, four MMD miRNA expression profile data were compiled. The GSE58105 was based on the GPL18743 platform; the GSE81152 was based on the GPL21814 platform; the GSE152267 was based on the GPL21572-124634 platform, and the GSE182194 was based on the GPL24741 platform, and it contained 88 MMD and 42 normal samples. The four miRNA chips mentioned above were merged by Perl, and the VENN package of the four miRNA chips were formed (Figure 2A) in the R4.1.0 VENN package. The R4.1.0 Sva and Limma were used to multi-chips for data rectification (batch Normalize). Genes acquired by the Limma software were determined to be significantly differentially expressed miRNAs using the corrected miRNA chip dataset and log2 (fold change) >2 or log2 (fold change) <−2 as screening thresholds (DE-miRNAs). In R4.1.0, the pheatmap package was used to construct DEGS heat maps and volcanoes.

### Identification of TF and target genes of DE-miRNAs

The FunRich3.1.3 software was used to annotate DEMs’ GO function, which included annotations of pathways and Transcription Factor (TF), as well as biological processes (BP), cellular components (CC), molecular functions (MF), etc. FunRich was used to classify miRNAs with statistical significance, up-regulation, and down-regulation in this work, and the top 10 Transcription Factor and target genes of DE-miRNAs were found.

### Data processing of DE-mRNAs

The GEO was searched for micro data on mRNA and miRNA expression (‘depression’ [MeSH] and mRNA [All Fields] and normal) AND (‘Homo sapiens’ [Organism] AND ‘Expression profiling by array’ [Filter]). The following were the inclusion criteria: MMD sufferers’ or healthy people’s plasma mRNA. Finally, five MMD mRNA expression profile data were acquired. The GSE19738 was based on the GPL6848-9572 platform, while the GSE32280, GSE44593, GSE53987, and GSE98793 were based on the GPL570-55999 platform, which included 80 MMD and 175 normal samples. Perl was used to integrate the aforementioned five mRNA chips, and VENN diagrams were created using the R4.1.0 VENN package (Figure 2B). The Sva and Limma of R4.1.0 were then employed exclusively for multi-chips data rectification (batch normalize). Adjusted *P* < 0.05 and log2 (fold change) >2 or log2(fold change) <−2 were used to screen corrected mRNA data. Limma identified genes were considered significantly differentially expressed mRNAs (DE-mRNAs). The pheatmap package in R software is used to construct DEGS heat maps and volcanoes.

### Intersection genes of target mRNAs and DE-mRNAs

Given the negative regulation relationship between miRNA and target genes, Perl software intersected target genes that have up-regulated DE-miRNAs and down-regulated DE-mRNAs, and target genes that have down-regulated DE-miRNAs were intersected with up-regulated DE-mRNAs, and the intersection genes were identified as core genes. The target genes of DE-miRNAs predicted by FunRich were then compared with those of DE-miRNAs from GEO. A Venn diagram was utilized to depict the crossing genes, and overlapping mRNAs were found.

### Construction of a miRNA-mRNA network and a protein-protein interaction network

Cytoscape3.7.2 was utilized to build the miRNA-mRNA network of mRNAs. The String online tool (https://string-db.org/) was used to create the PPI network of aberrant mRNA. With the protein type "Homo sapiens" and the highest level of confidence (0.150). The PPI network model was then created by Cytoscapee3.7.2. Despite the fact that PPI networks are less closely associated to mRNA expressions, our goal after differential analysis was to screen by PPI networks to identify hub genes, which had no influence on our results.

### GO and KEGG enrichment analysis of the miRNA-mRNA regulatory network

Following the acquisition of the core target, the ClusterProfiler, Colorspace, stringi, ggplot2, DOSE, enrichplot, and org. The R4.1.0 Hs.eg.db was utilized to examine the enrichment of GO and KEGG of the core target. These seven packages can be obtained from Bioconductor. GO enrichment primarily examined the target’s BP, CC, and MF, whereas KEEG enrichment examined the target’s potential biological pathways and activities.

### Identification of potential hub mRNAs and hub miRNAs

To filter the hub mRNAs, the CytoNCA plug-in selected the three most relevant characteristics based on the PPI network: Degree Centrality (DC), Closeness Centrality (CC), and Betweenness Centrality (BC). The values of these three factors showed the nodes’ topological importance in the network, and they mirrored the respective nodes’ function and impact in the whole network.

### Data availability statement

The datasets generated during and/or analyzed during the current study are available in the [GEO] repository, https://www.ncbi.nlm.nih.gov/geo/. The datasets generated during and/or analyzed during the current study are available from Supplementary.

### Ethics approval and consent to participation

This manuscript is not a clinical trial, hence the ethics approval and consent to participation are not applicable.

### Consent for publication

All authors have read and approved this manuscript to be considered for publication.

## RESULTS

The purpose of this study is to investigate, evaluate, and summarize the evidence of nerve-immunity-related miRNA in the plasma of MMD patients, so as to determine useful biomarkers, new therapeutic targets, and prognosis evaluation methodologies in the nerve-immunity interaction therapy of MDD. We predicted miRNAs and constructed a miRNA-mRNA network.

### Identification of DE-miRNAs and DE-mRNAs

**Figure 3:**
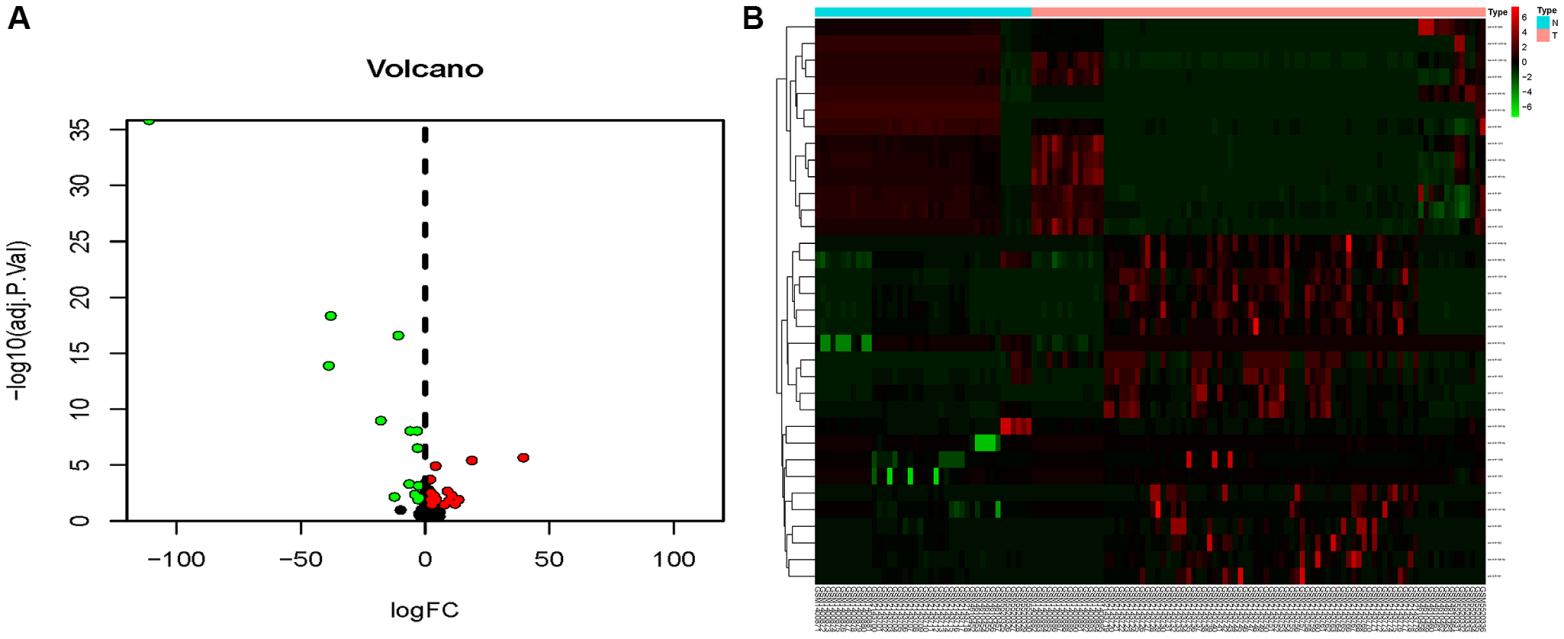
**Volcano map and heat map of DE-miRNAs.** (**A**) The volcano plot using log2|FC|≥2 and an adjusted *p* < 0.05 as cut off values for miRNAs; red dots represent upregulated DE-miRNAs (*n* = 15), blue dots represent downregulated DE-miRNAs (*n* = 12), and black dots represent non-DEmiRs. (**B**) A heat map depicting the expression variety of 34 DE-miRNAs across four miRNA chips; the color scale from blue to red represents expression levels ranging from low to high.

**Figure 4:**
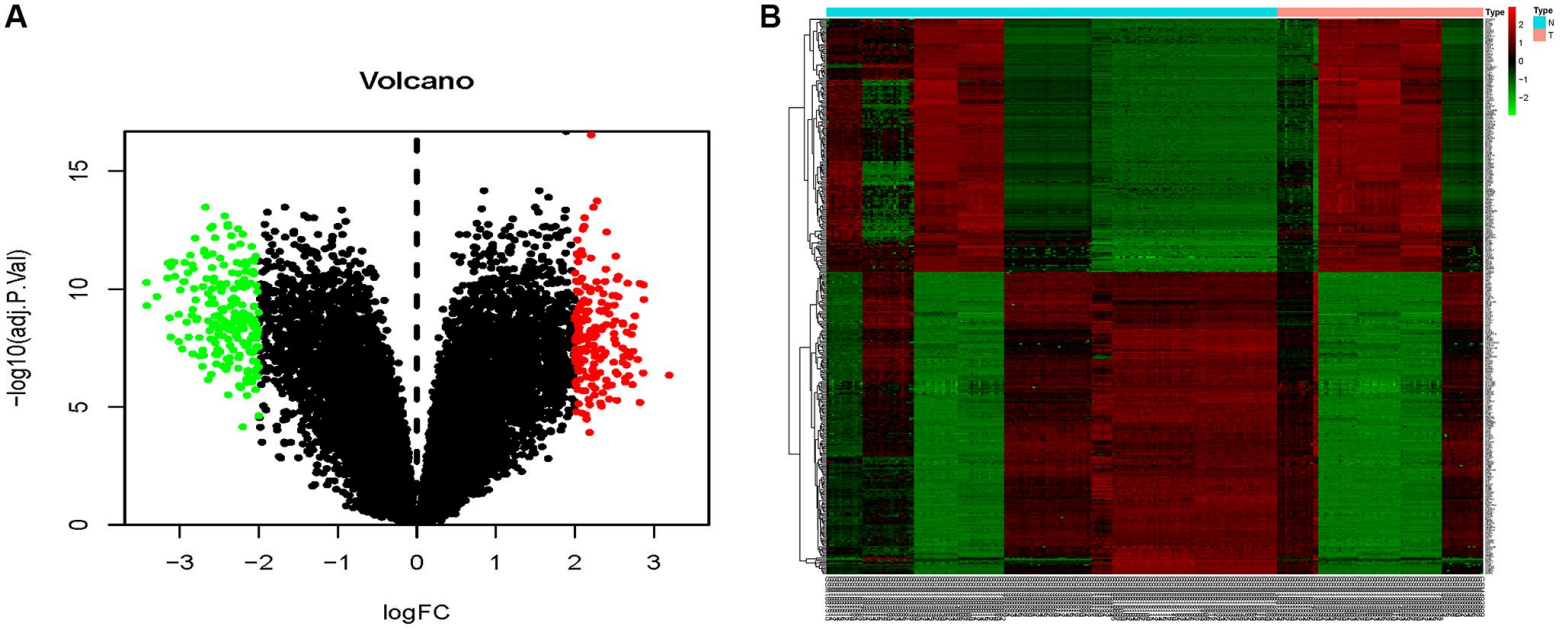
**Volcano map and heat map of DE-mRNAs.** (**A**) The volcano plot using log2|FC|≥2 and an adjusted *p* < 0.05 as cut off values for miRNAs; red dots represent upregulated DE-mRNAs (*n* = 181), blue dots represent downregulated DE-mRNAs (*n* = 205), and black dots represent non-DEmiRs. (**B**) A heat map depicting the expression variety of 386 DE-mRNAs across four miRNA chips; the color scale from blue to red represents expression levels ranging from low to high.

The combined dataset of 42 healthy people and 88 MMD patients revealed 34 DE-miRNAs (20 up-regulated and 14 down-regulated), following the screening with *P* < 0.05 and log2 (fold change) ≥2. To visualize the DE-miRNAs, a volcano map and a heat map were constructed (Figure 3). The DE-mRNAs of 80 MMD patients and 175 healthy controls were analyzed. A total of 386 DE-mRNAs were identified after screening with *P* < 0.05 and log2(fold change) ≥2 (181up-regulated and 205 down-regulated). To visualize the DE-mRNAs, the volcano map and heat map were utilized (Figure 4). The top 20 miRNAs and mRNAs exhibited differential expression (Table 1A, 1B).

**Table 1A:** Top 20 differentially expressed miRNAs.

**id**	**logFC**	**adj.P.Val**	**Regulated**
hsa-miR-574-5p	−110.9542898	1.49E-36	down
hsa-miR-940	39.47752153	2.16E-06	up
hsa-miR-486-5p	−38.71374032	1.32E-14	down
hsa-miR-1225-5p	−37.92807878	4.51E-19	down
hsa-miR-1207-3p	18.78576063	3.88E-06	up
hsa-miR-1207-5p	−17.826831	1.07E-09	down
hsa-miR-137	13.40197958	0.012279941	up
hsa-miR-320c	−12.34701801	0.007034566	down
hsa-miR-568	12.06373429	0.030023	up
hsa-miR-595	−10.80147681	2.64E-17	down
hsa-miR-1825	10.66823191	0.005454226	up
hsa-miR-1290	9.531297239	0.016235855	up
hsa-miR-450b-3p	8.988693552	0.002201271	up
hsa-miR-621	7.682599653	0.035136805	up
hsa-miR-1246	−6.396313788	0.000500662	down
hsa-miR-630	−6.024802257	8.95E-09	down
hsa-miR-875-3p	4.4232763	0.012241154	up
hsa-miR-325	4.178569237	1.23E-05	up
hsa-miR-127-3p	4.163199662	0.015960629	up
hsa-miR-483-5p	−4.110638734	0.00430359	down

**Table 1B:** Top 20 differentially expressed mRNAs.

**id**	**logFC**	**adj.P.Val**	**Regulated**
P2RY8	−3.420464032	5.27E-11	down
C16orf54	−3.414050815	5.07E-10	down
PPBP	−3.288699839	2.10E-10	down
PLP1	3.192684606	4.51E-07	up
PLAC8	−3.1541482	3.41E-11	down
IL2RG	−3.145809707	7.92E-12	down
S100A9	−3.127308745	1.67E-09	down
CXCR2	−3.112538855	1.08E-08	down
MPEG1	−3.112269482	1.12E-11	down
CSF2RB	−3.102043325	7.06E-12	down
CMTM2	−3.069217774	7.29E-12	down
CSTA	−3.061488684	2.90E-11	down
C5AR1	−3.014644308	1.18E-09	down
NCF2	−3.012015175	1.70E-08	down
SELL	−2.992395615	1.10E-10	down
S100A8	−2.96753421	3.42E-08	down
IL7R	−2.949694197	4.84E-11	down
EOMES	−2.941185679	4.82E-11	down
PLBD1	−2.936378649	7.59E-12	down
FCGR3B	−2.910908361	2.51E-09	down

### Predicting TF and target genes of miRNAs

**Figure 5:**
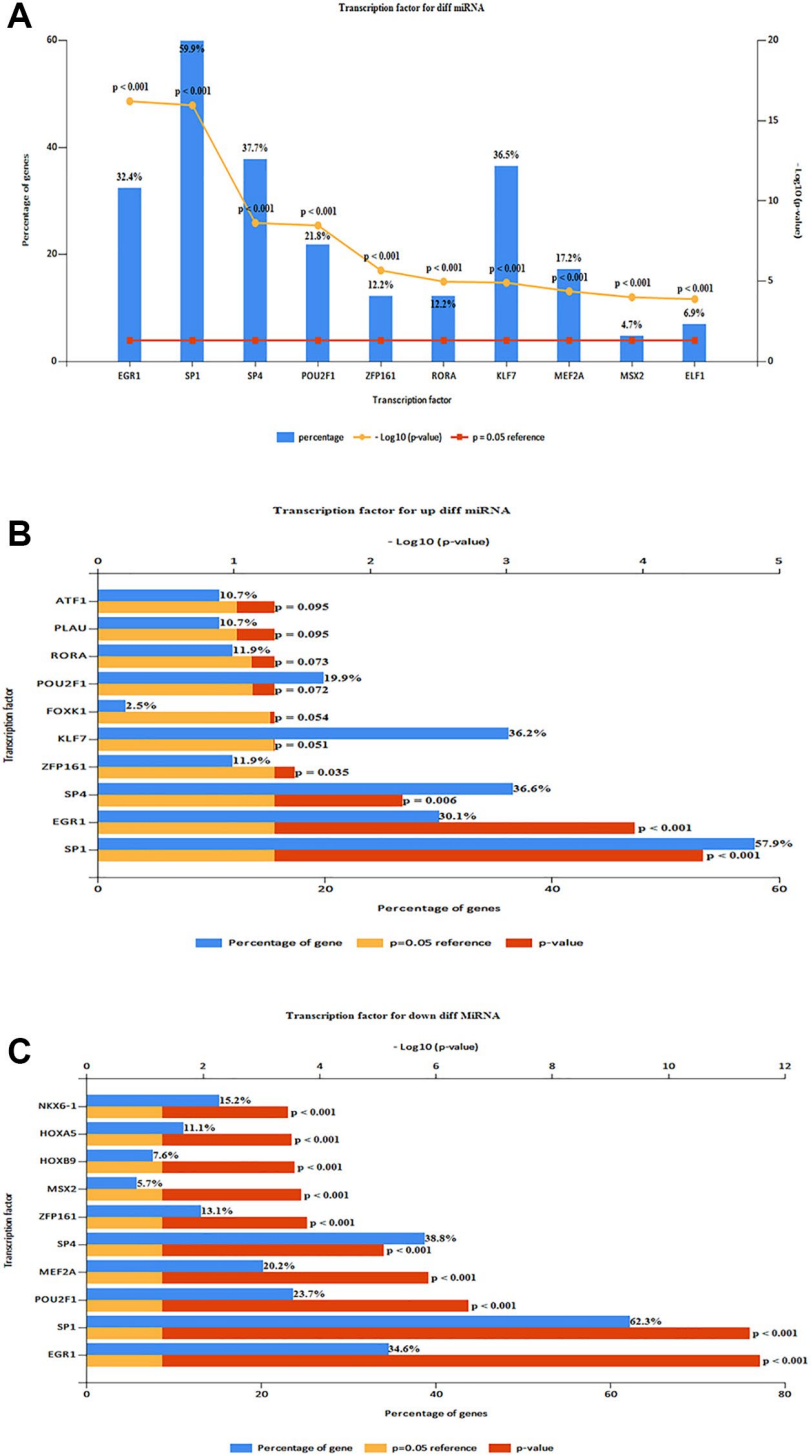
**Predicting TF and target genes of miRNAs.** (**A**) Top 10 TF of DE-miRNAs (SP1, SP4, KLF7EGR1, HNF4A, POU2F1, GABPA, ETS1, CTCF, and RREB1). (**B**) Top 10 TF of up-regulated DE-miRNAs (SP1, SP4, KLF7, EGR1, HNF4A, CTCF, POU2F1, NFYA, GABPA, and RREB1). (**C**) Top 10 TF of down-regulated DE-miRNAs (SP1, SP4, KLF7, EGR1, HNF4A, POU2F1, GABPA, ETS1, MEF2A, and NFIC).

The top 10 TF in DE-miRNAs included SP1, SP4, KLF7EGR1, HNF4A, POU2F1, GABPA, ETS1, CTCF, and RREB1 (Figure 5A). The top 10 TFs of up-regulated DE-miRNAs were SP1, SP4, KLF7, EGR1, HNF4A, CTCF, POU2F1, NFYA, GABPA, and RREB1 (Figure 5B) The top 10 TFs of down-regulated DE-miRNAs were SP1, SP4, KLF7, EGR1, HNF4A, POU2F1, GABPA, ETS1, MEF2A, and NFIC. 578 up-regulated target mRNA and 635 down-regulated DE-miRNAs were found (Figure 5C) For TF analysis and miRNA target genes, see (Supplementary Tables 1, 2).

### Functional annotation of DE-miRNAs

**Figure 6:**
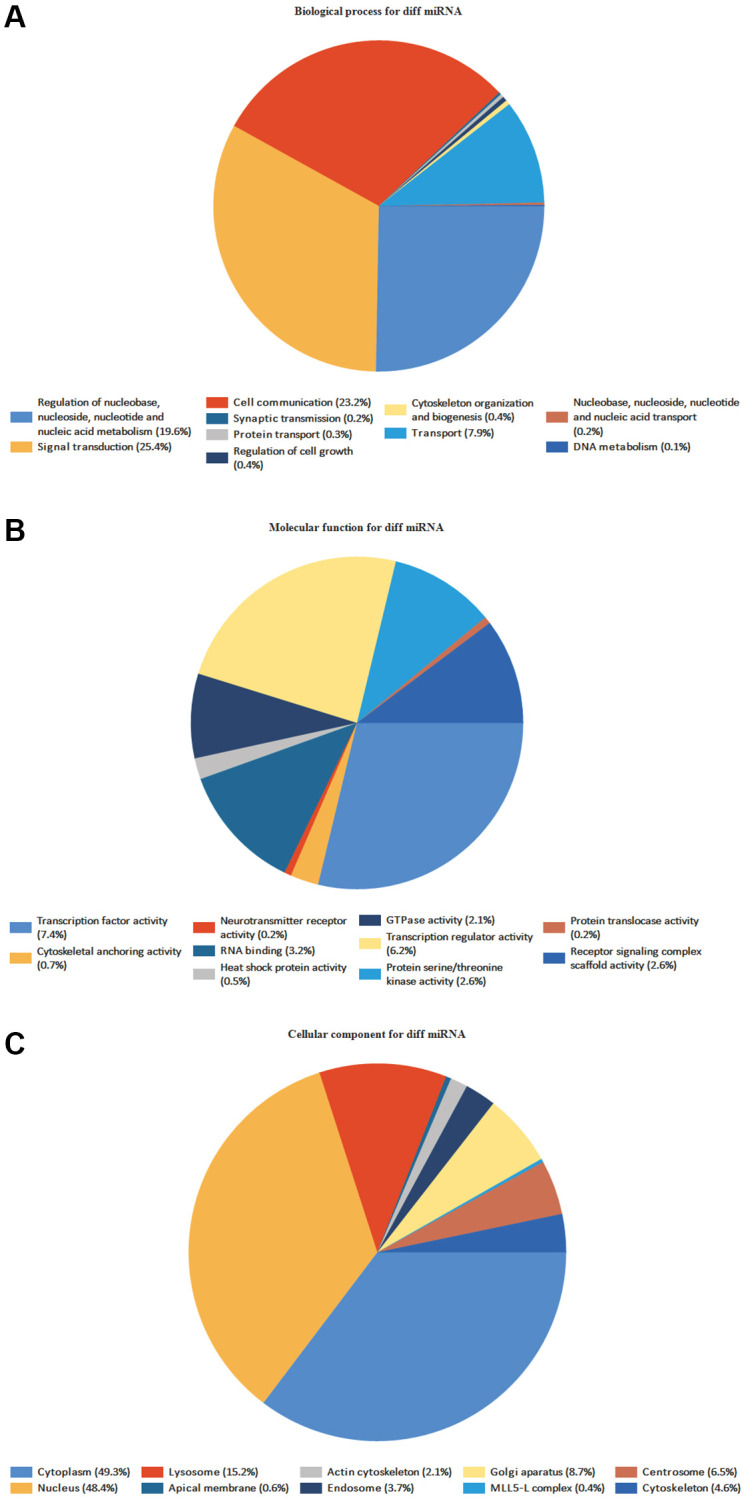
**GO enrichment analyses of DE-miRNAs.** The names and percentages of the top 10 (**A**) BPs, (**B**) MFs and (**C**) CCs are shown in a circle chart.

FunRich program discovered 59 BP, 267 CC, and 116 MF (Figure 6). MF mainly includes transcription activity, and DNA-RNA-Protein activity in the MF, MF uncertain, transcription factor activity, transcription regulator activity, DNA-RNA binding, protein serine/threonine kinase activity, etc. It is primarily connected to signal and cell communication in the BP, such as signal transduction, cell communication, unknown biological process, and regulation of nucleobase, nucleoside, nucleotide, and nucleic acid metabolism. It is mainly associated with Cytoplasm, Nucleus, Plasma membrane, Exosomes, and Lysosomes in the CC (Supplementary Tables 3–5).

### Identifying the intersection mRNAs

The 34 DE-miRNAs (20 up-regulated and 14 down-regulated) and 578 up-regulated and 635 down-regulated target mRNAs effectively predicted were compared to the 386 DE-mRNAs (181 up-regulated and 205 down-regulated) acquired from GEO data screening. The VENN map (Figure 2C) was constructed, which included 17 overlapping DE-mRNAs. Crossover genes are represented in (Table 2).

**Table 2:** The information of intersection genes.

**miRNA**	**mRNA**	**Target**	**mirnaLogFC**	**mrnaLogFC**
hsa-miR-206	GJA1	target	−3.251413682	2.682957112
hsa-miR-206	MAL2	target	−3.251413682	2.253644367
hsa-miR-206	PCDH17	target	−3.251413682	2.035934044
hsa-miR-206	SNAP25	target	−3.251413682	2.533742062
hsa-miR-206	SOX9	target	−3.251413682	2.070698257
hsa-miR-320c	GNAI1	target	−12.34701801	2.155037624
hsa-miR-320c	LMO3	target	−12.34701801	2.604451601
hsa-miR-320c	TUSC3	target	−12.34701801	2.034421167
hsa-miR-338-3p	CACNA2D1	target	−2.295135247	2.092690723
hsa-miR-338-3p	CHL1	target	−2.295135247	2.234787943
hsa-miR-338-3p	KCND2	target	−2.295135247	2.141220267
hsa-miR-338-3p	MYT1L	target	−2.295135247	2.069355937
hsa-miR-338-3p	NOVA1	target	−2.295135247	2.202273914
hsa-miR-296-5p	RAB37	target	2.171635035	−2.470323316
hsa-miR-137	BAZ1A	target	13.40197958	−2.100731
hsa-miR-137	RNASE6	target	13.40197958	−2.659559722
hsa-miR-137	SASH3	target	13.40197958	−2.661132519
hsa-miR-127-3p	RAB37	target	4.163199662	−2.470323316

### Construction of miRNA-mRNA network and PPI network

**Figure 7:**
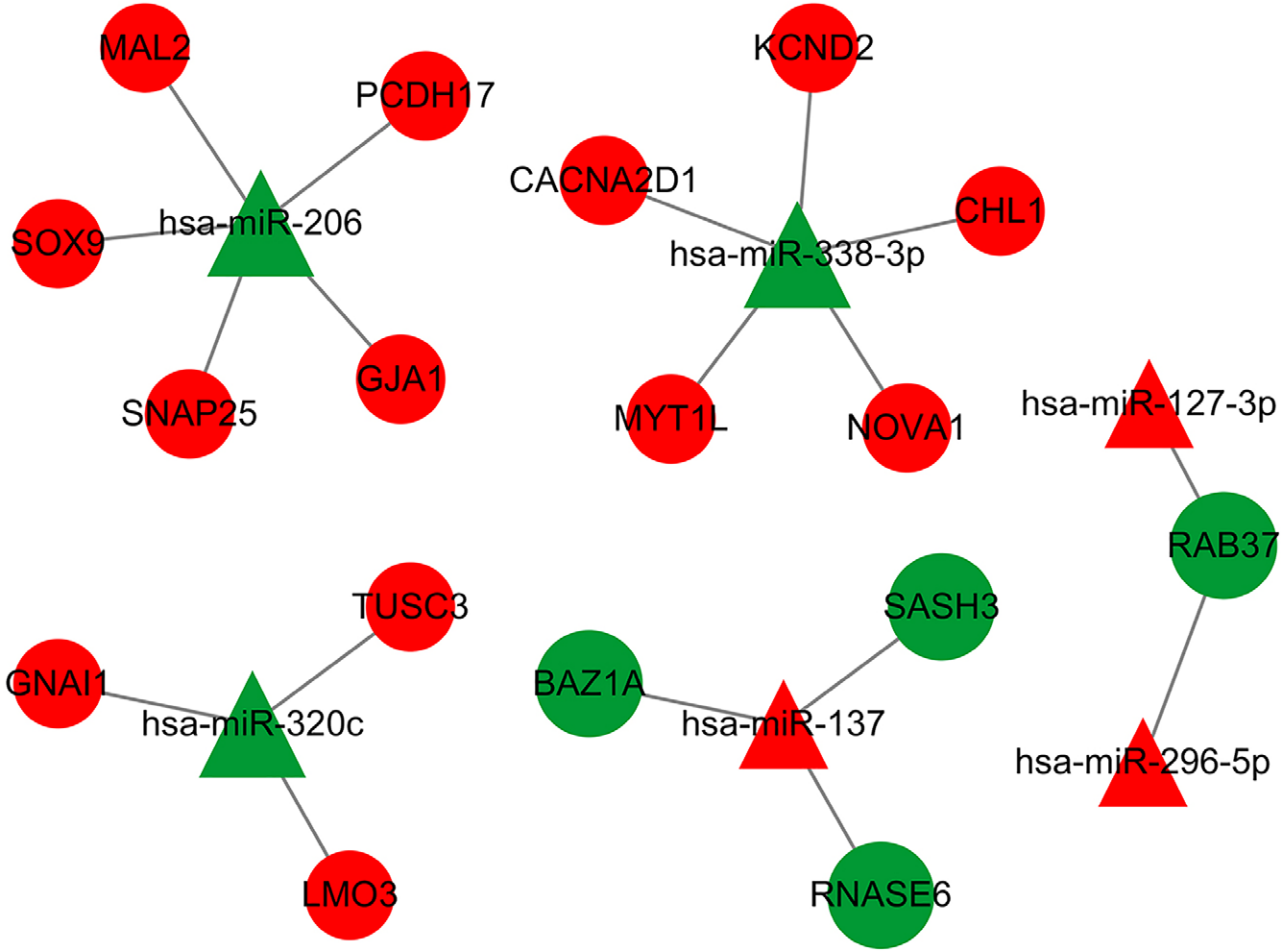
**The DEmiRNA-DEG regulatory network in MDD has been identified.** Red represents up-regulation, green represents down-regulation, a triangle represents miRNA, and a prototype represents mRNA. There were 23 nodes and 18 interactions in the DEmiR-downDEG regulatory network. There were 23 nodes, with 4 down-regulated DEGs and 3 DEmiRNAs, 13 upregulated DEGs and 3 DEmiRNAs.

Because miRNA and mRNA have a negative regulatory relationship, the previously collected intersecting DE-mRNAs were used to build a miRNA-mRNA regulatory network in Cytoscape3.7.2. The network contained 6 miRNA and 17 mRNA (Figure 7). Table 1 indicates the comparable expression of miRNA-mRNA in MMD.

### PPI network construction and screening of hub mRNAs and miRNAs

**Figure 8:**
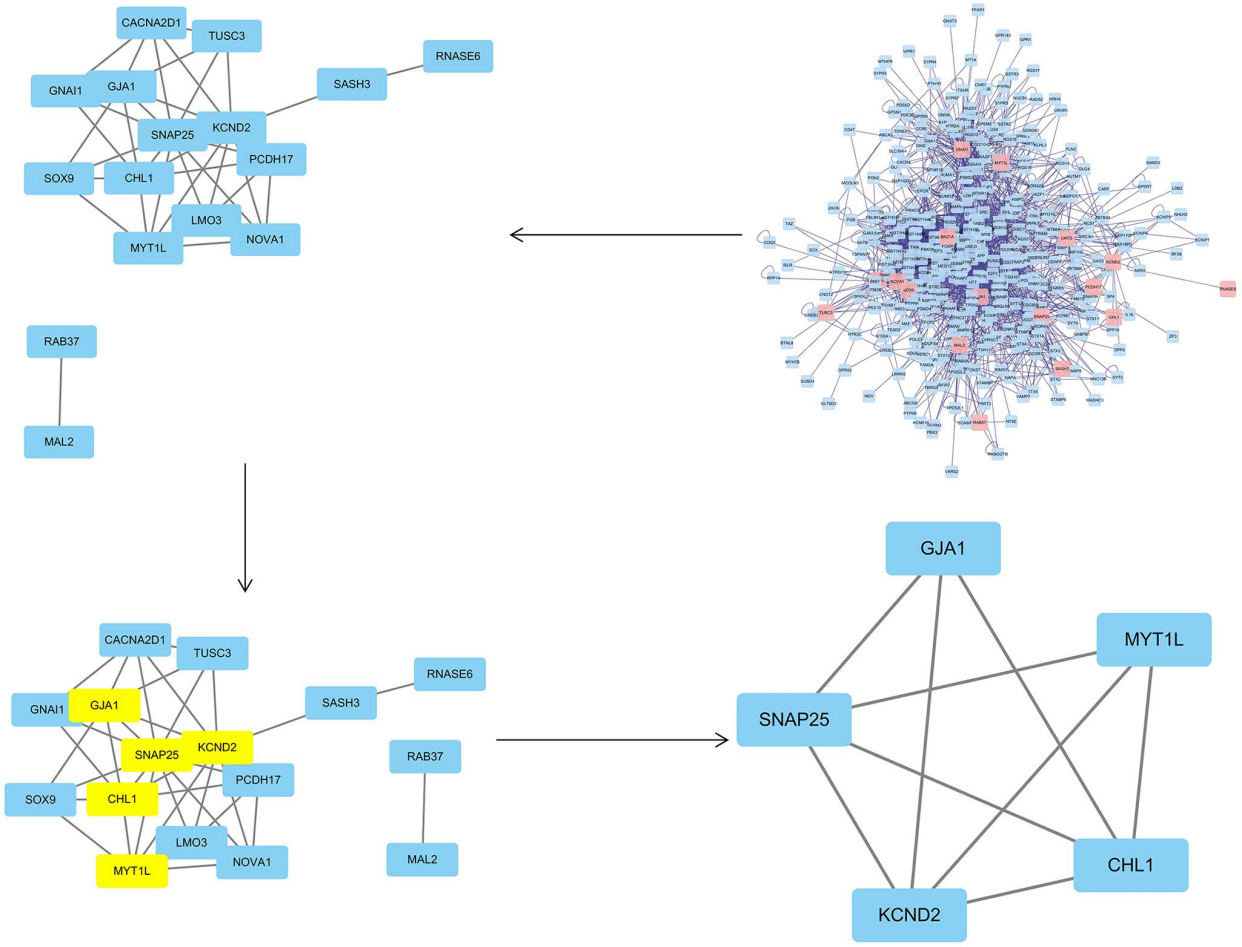
**The topological screening procedure for the PPI network.** 5 hub genes (KCND2, MYT1L, GJA1, CHL1, and SNAP25) were found.

To obtain the PPI network, the differentially expressed common genes from the two datasets were introduced in the String database, and the unconnected targets were deleted. Cytoscape3.7.2 BisoGenet revealed that the network had 369 nodes and 2485 edges. The CytoNCA plug-in was then used to further evaluate the network’s core targets, depending on the features of the network architecture. The target with twice the median value was picked depending on the DC, whereas the target with the median value was decided by BC and CC. Following screening, 5 hub mRNAs and their corresponding hub miRNAs (Supplementary Tables 6, 7) were identified, and node transmission information and transmission efficiency were selected as the "hub target" (Figure 8) for future research. It can be pointed out that the core genes include KCND2, MYT1L, GJA1, CHL1, and SNAP25 (Table 3).

**Table 3:** Information on 5 core targets.

**SUID**	**Gene symbol**	**Protein name**	**Betweenness**	**Closeness**	**Degree**
77	KCND2	Potassium voltage-gated channel subfamily D member 2	0.666666667	1	4
83	CHL1	Neural cell adhesion molecule L1-like protein	0.666666667	1	4
73	SNAP25	Synaptosomal-associated protein 25	0.666666667	1	4
92	MYT1L	Myelin transcription factor 1-like protein	0	0.8	3
75	GJA1	Gap junction alpha-1 protein	0	0.8	3

### GO and KEGG enrichment analyses of the miRNA-mRNA regulatory network

**Figure 9:**
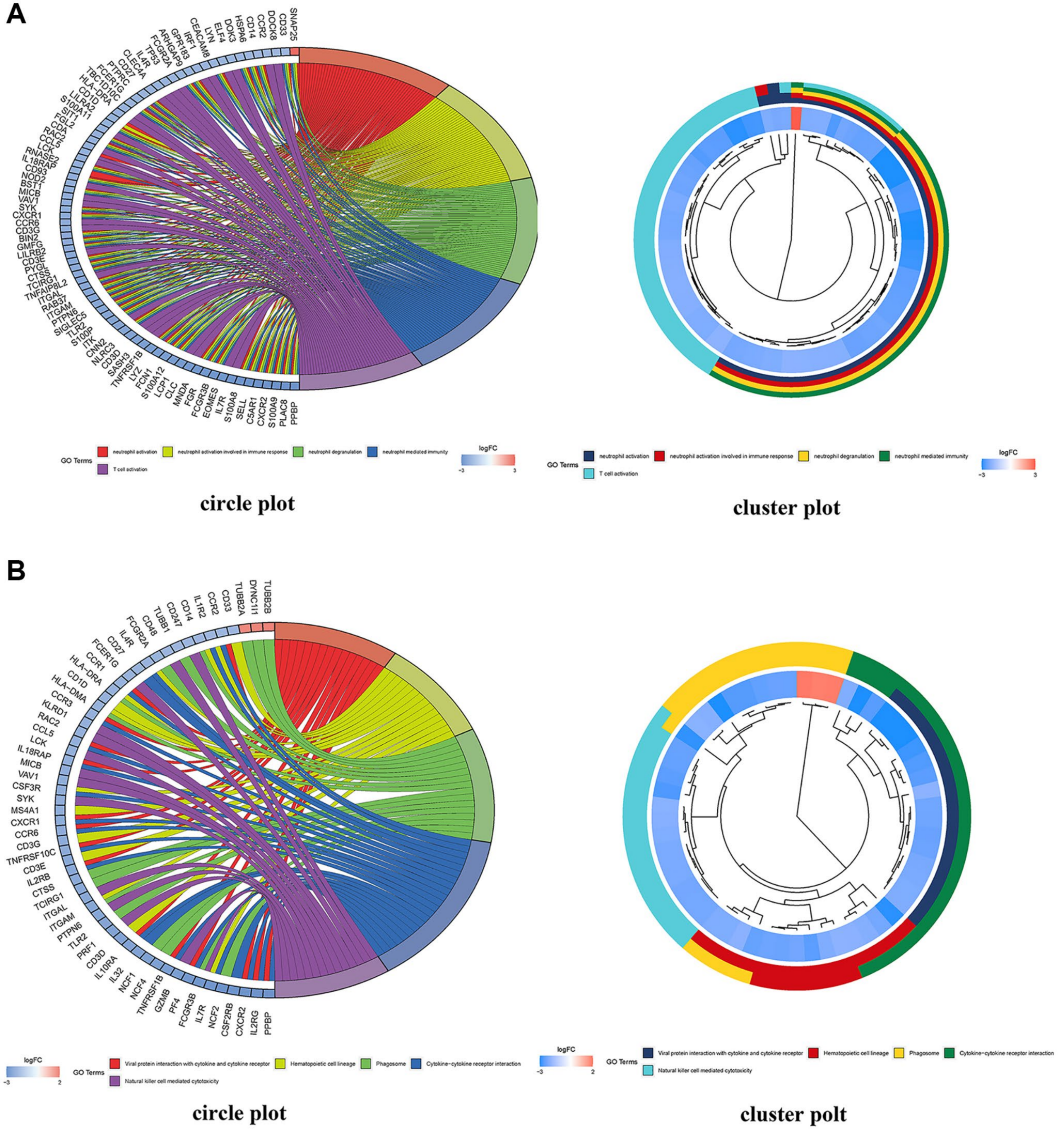
**DEG enrichment analyses using GO and KEGG.** (**A**) GO functional annotation enriched by DEGs in the GO database; red indicates a low *p* value and blue indicates a high *p* value; the size of the bubbles indicates the degree of enrichment, with larger bubbles reflecting a higher gene ratio. A circle plot depicting the top 5 GO functional annotations enriched by DEGs. (**B**) Pathways significantly enriched by DEGs in the KEGG database; red indicates a low *p* value and blue indicates a high *p* value; the size of the bubbles indicates the degree of enrichment, with larger bubbles indicating a higher gene ratio. A circle plot depicting the top 5 KEGG pathways enriched by DEGs.

470 BP, 83 CC, and 84 MF (Figure 9A) were identified by GO enrichment analysis of common DEMs by R4.1.0 software. In terms of molecular function, it mainly included channel activity (GO:0015267), passive transmembrane transporter activity (GO:0022803), immune receptor activity (GO:0140375), and nucleoside binding (GO:0001882). In the biological process, it mainly included neutrophil activation (GO:0042119), neutrophil activation involved in immune response (GO:0002283), neutrophil degranulation (GO:0043312), and neutrophil mediated immunity (GO:0002446). In the cellular composition, it mainly included presynapse (GO:0098793), external side of plasma membrane (GO:0009897), secretory granule membrane (GO:0030667), and synaptic membrane (GO:0097060) (Supplementary Tables 8–10). We selected the first 20 feature-rich processes to draw clusters and circles. In addition, we identified the main signal pathways involved in the occurrence and development of MMD in KEGG enrichment analysis and screened out the first 20 signal pathways related to MMD and significantly enriched. It included Cytokine-cytokine receptor interaction (hsa04060), Chemokine signaling pathway (hsa04062), Phagosome (hsa04145), Tuberculosis (hsa05152), Viral protein interaction with cytokine and cytokine receptor (hsa04061), Neutrophil extracellular trap formation (hsa04613) (Supplementary Table 11). We selected the first 20 principal signal pathways to draw cluster diagrams and circles, see figure (Figure 9B).

## DISCUSSION

Due to the stresses of work and life, the prevalence of MMD is growing year by year. Patients with MMD not only suffer from the disorder, but also have an enormous burden on their families and society as a whole. According to researches, nerve-immunity network dysfunction generates excessive production of inflammatory components and stimulates glucocorticoid release, both of which contribute to the initiation of MMD [23]. Increased expression of pro-inflammatory factors such as TNF-α, IL-6, and IL-1 in MMD patients’ and animal models’ serum, cerebrospinal fluid, and hippocampus forms a negative feedback regulation of the anti-inflammatory factor IL-10, resulting in neuronal inflammation and neuronal damage in the central nervous system [24].

At the moment, academics place a high value on nerve-immunity interaction treatment MDD. Nerve-immunity interaction therapy is associated with commonly used therapeutic therapies, such as escitalopram, fluoxetine, acupuncture, and Chinese materia medica. Common antidepressants, such as escitalopram and fluoxetine, have been shown to improve central function by increasing central serotonergic nerve activity. It can also successfully regulate the levels of brain-derived neurotrophic factors and inflammatory factors in patients’ blood [25]. Acupuncture can increase the content of serum 5-HT, up-regulate the content of anti-inflammatory cytokine IL-10, and reduce the content of proinflammatory cytokine IL-6 and TNF-α [26]. Chinese materia medica such as *Rhizoma Cyperi, Rhizoma Coptidis, Cinnamomum,* and *Radix Paeoniae Alba* can improve neuronal growth and development by mediating neurotrophic factors and other signal pathways, resist MMD-induced neuronal damage, and regulate signal transduction pathways such as NLR, ErbB, and chemokines, thus regulating inflammatory and immune responses, resulting in antidepressant effects [27]. Although it has been demonstrated that miRNAs play an important role in the initiation and progression of MMD, the link between miRNAs and mRNA remains unknown. As a result, it is critical to investigate MMD at the molecular level and to discover the mechanism of nerve-immunity interaction therapy of MDD, thus providing reference ideas for future fundamental research and clinical application.

We discovered 5 hub DE-mRNAs: KCND2, MYT1L, GJA1, CHL1, and SNAP25, which were all up-regulated genes. Both hsa-miR-206 and hsa-miR-338-3p, which are down-regulated in MMD, are similar miRNAs. Although 5 pairs of regulatory relationships between miRNA and mRNA have never been reported before, this study validated hsa-miR-206 with GJA1, SNAP25, hsa-miR-338-3p with KCND2, MYT1L via the miRDB database, and hsa-miR-338-3p via the TargetScan database, and predicted the possibility of interaction between 5 pairs of miRNA-mRNA. It provided theoretical groundwork for further experimental confirmation of the interaction of these miRNA and mRNA.

MiRNAs are tiny non-coding RNAs that directly regulate more than 30% of the genes in cells by inhibiting the degradation or translation of target mRNAs [28]. It links to nearly every fundamental biological function, including MMD and anxiety disorders [29]. Changes in miRNA expression are thought to be connected to the pathogenesis of a variety of neurodegenerative diseases [30]. MiR-206, a member of the muscle-specific miR-1 family, was first thought to regulate skeletal muscle embryonic development [31]. MiR-338-3p was first thought to be a cancer-related miRNA, but it was later found to be involved in neuronal regulation, including the Parkinson’s disease pathway [32]. From the neurological aspect: BDNF participates in neuroplasticity and protection throughout brain development, as well as playing an important role in emotion-related brain activities [33]. MiR-206 can suppress the production of BDNF following transcription, which is vital for the regulation and participation in MMD [34]. It was also discovered that miR-206 antagonists could attenuate stress-induced aggressive behavior while increasing BDNF expression in SI mice. Up-regulation of miR-206 has been demonstrated to impair neuron function and result in poor adaptive behavior [35]. MiR-338 can promote myelination in the nervous system, and over-expression of miR-338 can enhance oligodendrocyte differentiation while inhibiting myelin negative regulators Sox6 and Hes5, therefore promoting myelin formation [36]. MiR-338-5p has been found to safeguard the cognitive function of transgenic APP/PS1 mice by lowering neuronal death [37]. Other researches have shown that silencing miR-338-5p can result in neuronal polarity loss and a considerable decrease in the number of neurons. Thus, activating miR-338, to some extent, can influence the emotional function of the brain [38]. From the immune aspect: MiR-206 also links to the TNF signaling pathway [39]. The activated TNF pathway mediated by miR-206 can, in turn, drive NF-κB to enter the nucleus and boost the creation and release of inflammatory mediators such as TNF-α, IL-8, IL-6, and others to involve in inflammatory and immunological processes [40]. TNF inflammatory cytokines, which are plentiful in tumor microenvironments, can promote tumor growth, disrupt cell proliferation and death, and impair the innate immune response to cancer cells [41]. MiR-338-3p links to the TNF signaling pathway as well. TNF may induce inflammation by stimulating the NF-κB/MAPK signaling pathway and decreasing miR-338-3p levels [42]. Over-expression of miR-338-3p has been shown to block the ERK/p38MAPK signaling pathway and reduce the expression of pro-inflammatory genes VCAM-1 and ICAM-1RNA [43].

KCND2 is a potassium voltage-gated channel subfamily D protein-coding gene that can encode a type A potassium channel protein, which is important during the repolarization stage of an action potential [44]. By controlling potassium transport across the excitatory membrane in the brain, voltage-gated potassium channels regulate dendritic A-type current I (SA) in brain neurons [45]. Myt1L is a member of the Myt/NZF family, which encodes a neuronal transcription factor with 6 zinc fingers. Its expression has only been found in brain tissue thus far [46]. This gene is important to neural differentiation, and mutations in it have been linked to autism spectrum disorder and an autosomal dominant form of cognitive dysfunction [47]. Mutations in Myt1L in chromosomal band 2p25.3 have been linked to intellectual disability, while Myt1L repetition has been linked to schizophrenia and MMD [48]. Gap junction alpha-1 protein (GJA-1) expression had proven to be down-regulated in MDD patients, as was the Cx43 mRNA that encoded It [49]. CHL1 is a cell adhesion molecule from the L1 family. The deletion and repetition of this gene have been linked to a number of neurological disorders, including autism, Parkinson’s disease, and mental disability [50]. This study revealed that the level of serum CHL1 in patients with MMD declined dramatically and was inversely proportional to the severity of the disease, implying that CHL1 may be involved in the pathogenesis of MMD and immune cell dysfunction [51]. SNAP-25 has been proved to be associated with the intensity of depressive symptoms in patients with MMD as judged by the Beck MMD Rating Scale in numerous studies [52]. Both the hub miRNA and the mRNA created by constructing a miRNA-mRNA network may have a role in the incidence and progression of MMD via nerve-immunity interaction.

We explored the major targets of MDD using GO and KEGG enrichment to study the molecular mechanism. According to GO and KEGG enrichment analyses, the key targets contributed value in immunological and neuronal aspects of BP, CC, and MF. 83 CC mainly involves the presynaptic, synaptic membrane, neuronal cell body, synaptic connections between neurons, and voltage-gated sodium channel complex. 84 MF mainly involves immune receptor function, neurotransmitter receptor activity and chemokine binding. 470 BP mainly involves neutrophil, T cell and B cell activation, activation of surface receptors and immune response signal pathways, and regulation of chemical synaptic transmission. In addition, 39 MMD-related pathways, including immunological and neuroregulatory pathways, were identified and assessed.

Among the signaling pathways studied in this study, the signal pathways mentioned below are linked to immune and neurological regulation. The B cell and T cell receptor signal pathways, the chemokine signal pathway, and the JAK-STAT signal pathway. Th1 and Th2 cell differentiation, and Th17 cell differentiation are all involved. The human immune system’s Th17, Th1, and Th2 CD4+T lymphocyte subsets have critical roles in promoting inflammation and immunosuppression, respectively, and contribute to immune system homeostasis while supporting B cell activation and playing a role in humoral immunity. ① Th17 cell differentiation: Th17 contributes to the pathogenesis of MDD by boosting autoantibody production and the predisposition for autoimmunity [53]. Furthermore, Th17 and Treg are connected in differentiation and function, and they are frequently in a state of dynamic equilibrium. If the proportion of them is no longer balanced, it will result in aberrant immunological responses such as inflammatory, tumor and autoimmune reactions [54]. Th17 had been proven to be elevated in the brains of MMD model mice, but RORt defective mice demonstrated resistance to learning helplessness. As a result, an increase in Th17 can cause MMD, whereas suppressing Th17 production or function can lessen the risk of MMD in mice [55]. ② Th1 and Th2 cell differentiation: Both Th1 and Th2 cells can release cytokines, which can boost their proliferation while inhibiting each other to maintain a relative equilibrium. When the equilibrium is disrupted, also known as "Th1/Th2 drift," it can be expected to result in the emergence and development of a variety of disorders associated with immunological escape [56]. ③ T and B signaling pathways: In MMD patients, the immunological function was compromised, for T cell apoptosis increased, neutrophils rose, and total lymphocytes decreased. T cell function degradation could be due to inflammatory substances such as tumor necrosis factor-α destroying T cell function. B cells mediate humoral immunity via antibodies; as the number of B cells falls, so does the rate of transformation [57]. ④ Chemokine signaling pathway: the major purpose of chemokines is to control leukocyte migration (homing) to their proper regions during inflammation and homeostasis. Some control immune cell chemotaxis during the immune surveillance process, while others attract neutrophils to the site of infection or tissue injury for anti-infection, which acts as a directing role in the innate and adaptive immune systems. Furthermore, chemokines have an important role in the progression of disease [58]. ⑤ JAK/STAT signal pathway: This is not only one of the most important transmembrane signal transduction routes, but also the most important signal pathway activated by inflammatory cytokines [59]. Furthermore, JAK and STAT are key components of many signaling pathways that regulate cell growth, differentiation, survival, and pathogen resistance, including those involving the IL-6 (gp130) receptor family, which aids in the regulation of B cell differentiation, plasma cell production, and acute phase response [60]. Neural aspect: The JAK/STAT signaling pathway is widely implicated in the proliferation and differentiation of neural stem cells (NSC) throughout central nervous system development, and it is important in controlling hippocampal nerve reorganization [61]. A member of its family, the JAK2/STAT3 signal pathway, is involved in neuronal death and nervous reorganization in ischemic encephalopathy [62]. Numerous studies have revealed that TNF-α activates JAK/STAT signal pathway via the protein-gp130 on the cell membrane’s surface, and so contributes to the control of central nervous system degenerative alterations [63]. In addition, the JAK/STAT signaling pathway can influence neuron reorganization via inflammatory cytokines, BDNF, neurotransmitters, etc., [64]. As a consequence, medications and other treatment interventions can suppress neuronal death and promote nerve reorganization by altering the JAK/STAT signal pathway’s associated molecules.

Existing knowledge holds that the central nervous system is usually regarded as an immune zone due to the blood-brain barrier. However, as more researches are conducted, this idea is increasingly changing [65]. Chemokines, which include Th17, Th1, and Th2, are key chemicals in the immunological and neurological systems. They can coordinate this migration of T and B cells to inflammatory areas, promote T cell activity and help B cell activation, and play a role in regulating immunity-inflammation in the immune system [66]. Following psychological stress, activated T cells can enter the central nervous system in a variety of ways, reduce inflammation, and support neuronal integrity via cellular immunity, thus modifying depressed behavior [67]. Furthermore, the JAK/STAT signaling system influences brain remodeling by activating inflammatory cytokines and altering the metabolism of the neuro-endocrine-immune network.

Depending on the integrated analysis of miRNA-mRNA regulatory networks, miR-338-3p and miR-206 can coordinate the regulation of the neuro-endocrine-immune network by regulating chemokines such as Th17, Th1 and Th2, immune cells such as T/B and the JAK/STAT signaling pathway, among other things. The realization of bi-directional regulation of the HPA axis, which can normalize nerve conduction, minimize immuno-inflammatory responses, and restore normal neural, endocrine, and immunological function, plays a therapeutic role in MMD. To research useful biomarkers and treatment targets from the perspective of MMD and the host neuro-endocrine-immune network to regulate the level of immune-inflammatory cytokines in the microenvironment and balance neuro-endocrine-immune homeostasis at the molecular level. It is crucial to improve the critical mechanism of neuro-endocrine-immune imbalance in MMD patients.

There are quite a few papers published in recent years focusing on the relationship between miRNA and MDD [68–69]. However, compared to previous studies, we have focused more on the neurological and immunological aspects. First of all, we have made a new addition to miRNA regarding MMD in neuroimmunity compared to previous articles. Secondly, we used a joint analysis of multiple GEO data, although there were individual differences, we performed a batch Normalize data correction before the statistics to improve the stability and confidence of the results. It also reduced the small sample bias caused by the reduced sample size. Third, we not only performed TF analysis (including BP, CC, (MF)) using FunRich, but also GO and KEGG analyses, which allowed the results to be verified against each other to increase the feasibility of the results. Our research has limitations, although it provides theoretical underpinnings and research suggestions. First, we were unable to acquire sufficient external data from other publicly available sources to evaluate model’s dependability. Second, we focused more on miRNAs linked with neuroimmunity in our early expression analyses, but did not undertake any functional or mechanistic research. What’s more, although we batched Normalize various GEO datasets before analysis, it has little effect on the results, and there may still be bias when compared to analysis from the same dataset. We will undertake further research at a later time. Finally, no MDD studies were conducted to validate the relationship between prognostic genes and miRNA. As a result, additional research is required to confirm the above-mentioned findings and limitations.

## CONCLUSIONS

To summarize, MMD occurs and progresses as a result of multi-target, multi-pathway, multi-signal pathway, and multi-mechanism interactions, and the regulatory process is synergistic and bi-directional. MiRNA affects the production of KCND2, Myt1L, GJA-1, CHL1, and SNAP-25, which can activate or inhibit the B cell receptor signal pathway, T cell receptor signal pathway, and chemokine signal pathway. JAK-STAT and other signaling pathways control immune-inflammatory responses, neuron remodeling, and other processes, as well as affecting the onset and course of MMD.

Despite the fact that this study provides some theoretical underpinnings and research suggestions for nerve-immunity interaction, it has several limitations. The following suggestions are given for future improvement: ① Because the current data is derived from the GEO database, it is challenging to determine the dependability and quality of the statistical data. In the future, the number of data sources will be increased while decreasing the data offset. ② Conduct more scientific and clinical research to determine whether TCM therapies such as acupuncture and other therapies can enhance MMD patients’ neurological and immunological function by regulating the amount of miRNA in the brain.

## Supplementary Materials

Supplementary Tables 1-4 and 10

Supplementary Tables 5-9 and 11
