# Supplementary material for: Effective biomarkers and therapeutic targets of nerve-immunity interaction in the treatment of depression: an integrated investigation of the miRNA-mRNA regulatory networks
Source: Aging (Albany NY). 2022 Apr 25;14(8):3569–96. doi: 10.18632/aging.204030 (PMC9085226; doi:10.18632/aging.204030)
Supplement: Supplementary Tables 1-4 and 10 [file aging-14-204030-s001.doc]

**Supplementary Table 1. Results of transcription factor (TF) analysis of DE-miRNAs.**

| **Transcription factor** | **Percentage of genes** | **Fold enrichment** | **P-value (Hypergeometric test)** | **Mapped gene names** |
| --- | --- | --- | --- | --- |
| EGR1 | 32.41965974 | 1.538120059 | 2.9275E-19 | ARHGAP5; ARID4B; BTBD3; CELF2; COPS7B; CTDSPL2; DYNLL1; EIF5A2; FGF9; H3F3B; HAT1; MAP3K7; NFE2L1; PIM1; RAPGEFL1; SEMA3A; SMARCD2; SMOC1; SORCS1; ST6GALNAC6; TANC1; TSC22D2; TXLNG; ABHD2; ACTB; AKAP12; ANKRD34B; ANP32B; ANP32E; ASPH; AXL; AZIN1; BDNF; BLCAP; C2CD5; C2orf69; CALM2; CASK; CBL; CD164; CDC42SE1; CDK14; CDON; CITED2; CLTC; CNN3; CORO1C; CPEB1; CPLX2; CTTNBP2NL; DDX5; DGKH; DGKZ; DHX15; DLG4; EAF1; ELF1; ELMO1; EPB41L4B; EYA4; FBXW7; FGF17; FLI1; FOSB; GDF6; GLCCI1; GPR137C; HNRNPK; HNRNPU; HS3ST3B1; HSP90B1; JARID2; JUND; KALRN; KIF2A; KLF4; LASP1; LRCH1; MAB21L1; MAN1C1; MAP4K3; MATR3; MBLAC2; MCTP1; MIER1; MPZL1; MSANTD2; MXD1; NAB1; NETO1; NINJ1; NME1-NME2; NME2; NR1H3; NR4A2; PCDH17; PDGFA; PGD; PHAX; PHF6; PICALM; POLR3G; PRKRIR; PTMA; RNF111; RNF145; RSBN1; SDCBP; SEC63; SFPQ; SH3BGRL3; SLC35F1; SMIM14; SOX9; SP2; SPRED1; SRSF1; STC2; SYN3; TGIF2; THBS1; TIMP3; TMEM55B; TNPO2; UST; VAMP4; VASP; WDR1; WDR61; YPEL2; YRDC; YWHAZ; ZBTB8A; ZNF280C; ZNF281; ZNF800; ARPC2; CBFB; CCDC6; CPNE8; ELOVL6; IL6ST; KLF10; MAP3K3; MARCKS; MSX1; PDIK1L; PTEN; SRSF7; TOMM70A; ZFP91; DLX1; FOXQ1; FUS; GNAI1; GRASP; KCNS3; KITLG; KLF5; MDK; NABP1; NPAS2; PCSK7; PPCS; RGS9BP; SMIM15; STARD4; TMEM106B; TPD52L2; TSC22D4; VDAC1; VIM; CHTOP; COL1A1; DES; FAF1; FGD1; FKBP1A; FRS2; GNAQ; HNF4G; KCNA4; MBD5; N4BP2L1; NOL4; NONO; NOVA1; NUFIP2; PIP5KL1; PPM1B; PTPN12; RAB14; RNF114; SCAI; SEC61A2; SLC35F5; UBE2G1; UBE2Q1; USF1; ZBTB18; APOLD1; ATG2B; ATP8B2; BBC3; C16orf45; CNTFR; DYNLL2; DYRK1B; EPHA10; EPN1; HMGA1; KAZALD1; KCTD15; KMT2B; LARGE; LYPLA2; MLLT3; NRG1; NUMBL; NYNRIN; ONECUT2; PCDH1; PLEKHA2; PPFIA3; PPM1N; RAB37; RAB4B; RNF44; RNF5; RTN2; SLC30A3; SMG7; SOX12; SRF; TGFB1; THRA; ZC3H7B; ADAM23; AJUBA; AKT2; AP3S1; APPL2; ATP1B1; ATPAF1; BAZ2A; BCAP31; C21orf91; C3orf58; C6orf47; CA7; CLDN11; CSNK1A1; CTDSP2; CXXC4; CYYR1; DCAF8; DEXI; DNAJB5; DTX2; DUSP10; DVL2; E2F6; EBAG9; EFNA3; EPHA7; ERG; ESRRA; FAM98A; FHL3; FKBP4; FMNL2; GREM2; GRM7; HAPLN2; HLF; HLTF; HNRNPDL; HOXC4; HOXD10; IDH1; ING2; KCNAB3; KDM4A; KLF15; LBX1; LONRF3; MAPKAPK2; MARK3; MBD6; MPP1; MYO1D; NAB2; NAT8L; NCK1; NCOA2; NIPBL; NRXN1; NUCKS1; PAQR3; PDLIM3; PHF3; PLCB1; PPP1R14B; PPP1R37; PPTC7; PRADC1; PRDM1; PXN; RAB11B; RASIP1; RCOR2; RNF4; SCRT2; SLC25A5; SLC6A9; SPHK2; SPTY2D1; SS18; SSBP3; SUZ12; SYT1; TAF12; TCF12; TCF4; TCTN3; TDRD7; TFAP2C; THNSL1; TMEM218; TMEM229B; VARS; VASH2; WBP1L; WNT2B; ZBTB7A; ZFP36L2; ZFPM2; ZNF385D; ATP2B2; C17orf96; KLHL14; MEGF8; ZC3H4; |
| SP1 | 59.92438563 | 1.280156514 | 5.18865E-19 | ABHD17A; ABHD17B; ARHGAP5; ARID4B; ARMC8; ATXN7L3; BTBD3; CELF2; COPS7B; CSPG5; CTDSPL2; DCBLD2; DYNLL1; EIF5A2; FGF9; FOXO1; GOLGA3; HAT1; HCN1; MAP3K7; NFE2L1; PIK3R1; PIM1; RAPGEFL1; RASSF3; RFFL; SMAD2; SMARCD2; SMOC1; ST6GALNAC6; STK4; TSC22D2; TXLNG; ABHD2; ACER2; ACTB; ADPGK; AKAP11; AKAP12; ANKRD34B; ANP32B; ANP32E; ARFIP1; ARG2; ARID2; ARPC3; ASH2L; ASPH; ATG13; ATP6V1A; AXL; AZIN1; BAG4; BDNF; BLCAP; BRI3BP; BSCL2; C1GALT1; C2CD5; CALM2; CASK; CBL; CDC42; CDC42SE1; CDK14; CDK4; CDON; CHSY1; CITED2; CLCN3; CNN3; CORO1C; CPEB1; CPLX2; CTBP2; CTTNBP2NL; CXCR4; DCAF12L1; DDX5; DGKE; DGKZ; DLG4; E2F5; EAF1; EIF1AX; EIF4E; ELF1; EPB41L4B; EYA4; FAM155A; FAM91A1; FBXO33; FBXW7; FLI1; FN1; FNBP1L; FOSB; FUBP1; FZD7; G6PD; GDF6; GLCCI1; GLS; GPR137C; HIAT1; HMGN1; HNRNPK; HNRNPU; HOXB4; HS3ST3B1; HSP90B1; HSPD1; HSPE1; JARID2; JUND; KALRN; KANK4; KCNJ2; KIF2A; KLF4; KRAS; KTN1; LASP1; LIN7C; LRRC8A; MAN1C1; MAP4K3; MATR3; MBLAC2; MCTP1; MIER1; MMD; MON2; MSANTD2; MTX1; MXD1; MYEF2; NAP1L5; NBEA; NCL; NDRG3; NET1; NETO1; NINJ1; NR1H3; NR4A2; NXT2; OSBPL7; PCDH17; PDE7A; PDGFA; PEA15; PFN2; PGD; PHF6; PICALM; POGK; POLR3G; PPIB; PPP4R2; PRKRIR; PTBP1; PTMA; PTPRS; RAB11FIP1; RABEPK; RARB; RASA1; RNF111; RNF138; RNF141; RRAGC; RRBP1; RSPO3; RYBP; SDCBP; SEC23B; SEC63; SEPT2; SFPQ; SH3BGRL; SH3BGRL3; SKIDA1; SLC10A7; SLC25A22; SLC35B3; SLC44A1; SMAP1; SMARCB1; SNAP25; SNX2; SOX9; SP2; SPRED1; SRSF1; SRSF9; STARD7; STC2; STX12; SYN3; TAGLN2; TGIF2; THBS1; TIGD6; TIMP3; TMEM135; TMEM55B; TMEM68; TMSB4X; TMX1; TNPO2; TPK1; TPM3; TSPAN4; USP33; UST; UTRN; VAMP2; VAMP4; VASP; WDR1; WDR61; XPO6; YPEL2; YRDC; YWHAQ; YWHAZ; ZC2HC1C; ZNF148; ZNF280C; ARPC2; ATP6V1G1; C9orf72; CBFB; CCDC6; CCNG1; CCNT2; CD2AP; CTNNBIP1; DLG5; DNAJB4; EFNB2; FOXN2; FRMD5; IL6ST; KCNG3; KLF10; MAP1LC3B; MAP3K3; MARCKS; MSX1; NEK6; NMNAT3; NRG3; OPRK1; PALM2-AKAP2; PDIK1L; PTEN; SRSF7; TOMM70A; XRCC5; ZFP91; ARMCX2; BANP; CCR7; CD274; DAZAP1; DBN1; DDX42; DPY30; EFS; FOXQ1; FTL; FUS; GRASP; HECTD2; INSM2; IRF6; KCNS3; KLF13; KLF5; LMO3; MDK; MED21; MYL12A; NKX2-4; PAPD5; PBX3; PCSK7; PHF1; PLK3; POLE4; RAP1A; RBM24; RCN2; RGS9BP; SCOC; SLC10A3; SMIM15; STARD4; SYNGR2; TMEM108; TSC22D4; VIM; YWHAH; ARGLU1; ARHGEF10L; ARL5A; ATP10D; C12orf4; C16orf87; CELSR2; CHL1; CHTOP; COL1A1; COPS4; DCAF12; DES; FAF1; FAM169A; FGD1; FKBP1A; FOPNL; FRS2; GNG12; HMOX2; HNF4G; HUNK; KCNA4; LGALSL; LTA4H; MAFB; MBD5; MTHFD1L; N4BP2L1; NIPAL1; NNT; NOL4; NOVA1; NTPCR; NUFIP2; PABPC1L; PELI3; PI4K2B; PIP5KL1; PPM1B; PTPN12; PVALB; RAB14; RAB23; RNF114; SEC61A2; SLCO3A1; TFG; TM6SF1; UBE2G1; UBE2Q1; USF1; WNK4; ZBTB18; ACTR1A; ADAMTS10; AMMECR1L; ATG2B; ATP8B2; BBC3; CAD; CAPN6; CNTFR; DYNLL2; DYRK1B; EPHA10; EPN1; FBRS; FKBP8; GAB2; GDI1; HM13; HMGA1; HOXB6; IPO11; ISCA2; KAZALD1; KCNA6; KCTD15; LARGE; LDLRAP1; LYPLA2; LZIC; MACF1; MLLT3; NRG1; NUAK2; NUMBL; NYNRIN; ONECUT2; PCDH1; PHC2; PLEKHA2; PPAN; PPFIA3; PPM1N; PRRT2; RAB37; RAB4B; RNF44; RPRD2; RTN2; SLC30A3; SMG7; SOX12; SRF; STOML1; TEAD3; TGFB1; THRA; TSFM; VKORC1L1; AACS; ACSL6; ACVR1; AHR; AJUBA; AKT2; ANKRD13A; ANXA7; AP3S1; APPL2; ARHGEF18; ATP1B1; ATPAF1; BAZ1A; BAZ2A; BCAP31; BEND3; C11orf87; C3orf58; C6orf47; CA7; CACFD1; CCNG2; CCNI; CD69; CDC37L1; CDC42EP2; CLDN11; CNEP1R1; CTDSP2; CXXC4; CYB5R4; CYYR1; DCAF8; DENND1A; DEXI; DHX40; DNAJB5; DSP; DTX2; DUSP1; DUSP10; E2F7; EBAG9; EFEMP2; EFNA3; EHBP1L1; ELOVL2; ENHO; EPHA5; EPHA7; ERG; ESRRA; ESRRG; ETFB; EZH2; FAM126B; FAM168A; FAM98A; FHL3; FKBP4; FMNL2; FURIN; FXYD6; GJC1; GLIS2; GPR137B; GPX7; GRIN2A; GRM7; HERPUD2; HIC1; HLF; HLTF; HNRNPDL; HOXC4; HTR2C; IDH1; IMPA2; ING2; INPP5A; KCNAB3; KDM1A; KDM4A; KDM5B; KIT; KLF11; KLF15; KLHL17; LBX1; LEPROT; LMBR1L; LRRC10B; LURAP1L; LUZP2; MAFK; MBD6; MIA3; MOB4; MPC1; MPP1; MSI1; MTA3; MTDH; MTF1; MYO1D; NAB2; NCK1; NCOA2; NIPBL; NKPD1; NPC1; NRXN1; NUCKS1; OSER1; PAQR3; PDCL; PDHB; PDLIM3; PHF3; PIAS2; PIAS4; PLA2G15; PPP1R14B; PPP1R37; PPTC7; PRADC1; PRDM1; PROX1; PRR16; PTGES2; PTGFRN; PTPN2; PTPN5; PXN; RAB11B; RAB9A; RASIP1; RCOR2; RNF165; RNF38; RNF4; RPGRIP1L; RPL15; RPL28; RREB1; RRM2B; SAMD8; SIRT6; SLC12A2; SLC1A5; SLC22A18; SLC46A3; SLC6A8; SPHK2; SPTLC1; SPTY2D1; SRC; SRSF6; SS18; SSBP3; SSFA2; ST18; STAC; STK38; SUZ12; SWT1; SZRD1; TADA2B; TCF12; TCF4; TFAP2A; TFAP2C; THNSL1; TMED5; TMEM218; TMEM229B; TPCN1; TSNAX; UBXN4; UGCG; UNC79; UNC93B1; USF2; VASH2; WBP1L; WNT2B; WNT7A; YBX1; YTHDC1; ZBTB7A; ZC3H3; ZC3H6; ZCCHC2; ZFP36L2; ZFPM2; ZFYVE16; ZMAT4; ZNF385D; ZNF449; ZNF516; ZNF710; ATP2B2; C17orf96; CYB561D1; CYB5D1; ETNK2; GINS4; ITGA6; KIF3B; KLHL14; MAPK4; MEGF8; MTSS1L; SGSM2; ZC3H4; |
| SP4 | 37.71266541 | 1.323910975 | 1.11829E-11 | ARID4B; ATXN7L3; BTBD3; CELF2; COPS7B; CSPG5; DCBLD2; FGF9; HAT1; HCN1; MAP3K7; NFE2L1; SEMA3A; SMOC1; SP5; ST6GALNAC6; TSC22D2; TWF1; TXLNG; ABHD2; ACER2; ACTB; AKAP11; ANKRD34B; ANP32E; ARG2; ARID2; ASH2L; ASPH; ATG13; ATP6V1A; AZIN1; BACH2; BAG4; BDNF; BET1; BLCAP; C2CD5; CALM2; CBL; CCNC; CDC42SE1; CDK4; CITED2; CORO1C; CPLX2; CTTNBP2NL; CXCR4; DGKZ; DLG4; E2F5; EAF1; EIF1AX; EIF4E; ELF1; ELMO1; EPB41L4B; EYA4; FAM107B; FBXO33; FBXW7; FLI1; FOSB; FZD7; GCLC; GDF6; GLS; GPR137C; HIAT1; HNRNPK; HNRNPU; HS3ST3B1; HSP90B1; HSPD1; JARID2; KCNIP3; KCNJ2; KLF4; LASP1; LIN7C; MATR3; MBLAC2; MCTP1; MIER1; MMD; MOB3C; MXD1; MYEF2; MYLK; NAB1; NBEA; NCK2; NDFIP2; NET1; NR1H3; PCDH17; PFN2; PGD; PLEKHA1; POLR3G; PPP1R3B; PTBP1; PTMA; PTPRS; RASA1; RNF111; RNF138; RNF141; RRAGC; RSBN1; RSPO3; SDCBP; SEC23B; SEC63; SEPT2; SH3BGRL3; SKIDA1; SLC10A7; SMAP1; SMARCB1; SNAP25; SNX2; SOX9; SP2; SPRED1; STC2; STX12; SYN3; TGIF2; THBS1; TIMP3; TMEM135; TMEM55B; TMEM68; TMX1; TPM4; USP33; UST; UTRN; VAMP4; VASP; WDR1; XPO6; YPEL2; YRDC; YWHAQ; ZNF280C; C6orf106; CBFB; CCDC6; CPNE8; FBXO9; FOXN2; IL6ST; KLF10; MARCKS; MSX1; NEK6; NMNAT3; NRG3; PDIK1L; PTEN; RAP2C; SRSF7; TMEM39A; TOMM70A; ZFP91; ARMCX2; CD274; EFS; FUS; IRF6; KITLG; KLF13; KLF5; MDK; MSI2; PBX3; PCSK7; PHF1; PLK3; RAP1A; RBM24; RCN2; RGS9BP; SLC10A3; SMIM15; STARD4; TPD52L2; TUSC3; C16orf87; COL1A1; COPS4; DCAF12; DES; FAF1; FAM169A; FGD1; FOPNL; FRS2; HEXIM1; HMOX2; HUNK; KCNA4; MAFB; MBD5; MTHFD1L; MTUS1; NOL4; NTPCR; NUFIP2; PABPC1L; PELI3; PI4K2B; PIP5KL1; PPM1B; RAB14; RGS7BP; RNF114; SEC61A2; SLCO3A1; UBE2G1; UBE2Q1; ZBTB18; ACTR1A; ADAMTS10; ATG2B; ATP8B2; BBC3; DYRK1B; EPHA10; FKBP8; GAB2; GDI1; HM13; HMGA1; INPP5K; IPO11; ISCA2; KAZALD1; KCTD15; LARGE; LDLRAP1; LRFN1; LYPLA2; MACF1; NUAK2; NUMBL; NYNRIN; ONECUT2; PCDH1; PHC2; PLEKHA2; PPM1N; RAB37; RAB4B; RNF44; RNF5; RTN2; SLC30A3; SMG7; SOX12; TEAD3; TGFB1; VKORC1L1; ZC3H7B; AACS; ADAM23; AHR; AJUBA; AP3S1; APPL2; ARHGEF18; ATPAF1; BCAP31; BEND3; BNIP3L; C3orf58; CA7; CACFD1; CCNG2; CD69; CEP63; CHGA; CHST1; CLDN11; CTDSP2; CXXC4; CYYR1; DENND1A; DEXI; DHX40; DNAJB5; DSP; DTX2; DUSP10; DUSP4; DVL2; EBAG9; EFEMP2; EFNA3; EPHA7; ERG; ESRRA; ESRRG; ETFB; EZH2; FAM172A; FHL3; FKBP4; FMNL2; FSTL1; GLIS2; GPR137B; GPX7; GRIN2A; GRM7; HAPLN2; HLTF; HMGN3; HOXC4; IDH1; ING2; INPP5A; KDM4A; KLF11; KLF15; LBX1; LEPROT; LONRF3; LRRC10B; METTL9; MITF; MOB4; MPC1; MPP1; MTA3; MTDH; MYO1D; NAT8L; NCOA2; NKPD1; NRXN1; NUCKS1; OSER1; PDHB; PDLIM3; PEX3; PHF3; PIAS4; PPP1R14B; PPP1R37; PPTC7; PRDM1; PTGES2; PTPN2; RAB11B; RASIP1; RCOR2; RNF165; RNF4; RPL15; RRM2B; SH3TC1; SIRT6; SLC1A5; SLC30A4; SLC6A9; SPTY2D1; SRPR; SS18; ST3GAL3; SUZ12; TBX3; TCF12; TCF4; TFAP2C; THNSL1; TMEM218; TMEM229B; VASH2; VAX1; WIF1; WNT2B; WNT7A; ZCCHC2; ZFP36L2; ZFPM2; ZFYVE16; ZNF710; ATP2B2; C17orf96; CYB561D1; GINS4; KLHL14; MEGF8; MTSS1L; SGSM2; ZC3H4; |
| POU2F1 | 21.83364839 | 1.507644267 | 1.61226E-11 | AFF3; ARHGAP44; ARID4B; ARMC8; BTAF1; BTBD3; CDH7; CTDSPL2; DYNLL1; H3F3B; MAP3K7; NFE2L1; SEMA3A; SMAD2; SMOC1; SORCS1; ANP32B; ANXA2; BDNF; BET1; C2CD5; CASK; CCNC; CDK14; CDK4; CLCN3; CLTC; CTBP2; DGKH; EDN1; EHMT2; ELF1; ELMO1; EYA4; FAM63B; FBXW7; FLI1; FN1; FUBP1; GCFC2; GJA1; GLCCI1; GLS; GPD2; GPR6; HACE1; HIAT1; HNRNPK; IGF1; JARID2; JUND; KALRN; KCNJ2; MAB21L1; MON2; MXD1; MYLK; NBEA; NETO1; NR4A2; OTX2; PEA15; PHF6; PLEKHA1; RABGAP1L; RARB; RNF14; SH3BGRL; SKIDA1; SLC35F1; SNAI2; SNAP25; SP2; SPRED1; STX12; TERF2; TMCC1; TMEM178A; TMSB4X; TPK1; UST; UTRN; YWHAQ; ZC2HC1C; ZNF148; ZNF281; CCNT2; CD2AP; PALM2-AKAP2; PTEN; SRSF7; ZFP91; CCR7; CD274; CDK13; DLX1; GRASP; HECTD2; KITLG; KLF5; LMO3; MSI2; NPAS2; PAPD5; PBX3; PLEKHA5; RBM24; SCOC; SMIM15; SPOPL; VIM; YWHAH; ARGLU1; ARL5A; CACNA2D1; CHL1; DGKB; FAM120A; FRMD3; FRS2; HNF4G; HUNK; MACROD2; MBD5; MTUS1; MYT1L; NOVA1; NUFIP2; PPM1B; PTPN12; SCN1A; SEC61A2; SEPT8; SLC35F5; SLCO3A1; WAPAL; ZBTB18; AAK1; AMMECR1L; CAD; GAB2; HIPK1; HMGA1; HOXB6; IPO11; LARGE; LDLRAP1; MACF1; MLLT3; NRG1; PHC2; RAB4B; SOX12; SRF; THRA; ACSL6; ACVR1; AHCYL1; AHR; AJUBA; ATP1B1; BAZ1A; BEND3; BNIP2; CACFD1; CEP128; CTNNA3; CXXC4; CYYR1; DENND1A; DIRAS2; DMRT3; E2F7; EBAG9; EN2; EPHA5; ESRRA; ESRRG; FAM126B; FAM98A; GALNT1; GPCPD1; GPR158; GPRC5A; GREM2; GRIP1; GRM7; HTR2C; KDM4A; KDM5B; KLF12; KLF15; L3MBTL4; LINGO2; MAPK10; MBD6; MBNL2; METTL9; MITF; MTA3; NR1H4; NREP; NRXN1; NRXN3; PDLIM3; PPARGC1A; PRDM1; PRKAB1; PROX1; RAPGEF5; RCAN2; RNF38; SIPA1L2; SLC17A6; SLMAP; SPTLC1; SPTY2D1; SSFA2; SUGP2; SYT1; TNC; TSNAX; UBXN4; UNC79; VASH2; WIF1; ZBTB7A; ZFPM2; ZNF516; ATP2B2; ITGA6; |
| ZFP161 | 12.19281664 | 1.635779922 | 1.02136E-08 | ARHGAP5; ATXN7L3; BTBD3; CDH7; FGF9; HCN1; ACTB; ADAM12; ASPH; BACH2; CDC42; CPEB1; CTBP2; EIF4E; ELMO1; FAM155A; FN1; G6PD; GCFC2; KLF4; MATR3; MBLAC2; MCTP1; NECAB1; NETO1; NR1H3; OSTF1; PAX3; PDE7A; PFN2; POLR3G; PTBP1; PTMA; PTPRS; RASA1; RYBP; SH3GL1; SMARCB1; SPRED1; TMCC1; TMEM135; ZNF26; CCNT2; ELOVL6; MAP3K3; PDIK1L; PTEN; RNF170; SLC22A3; ZFP91; EFS; FOXQ1; GNAI1; KCNS3; KLF13; KLHL36; LMO3; NPAS2; PBX3; PHF1; POLE4; CHL1; FAF1; FAM120A; FGD1; FKBP1A; FRMD3; GNG12; HUNK; MAFB; METTL21A; NNT; NOL4; PELI3; PTPN12; SLCO3A1; ATP8B2; BBC3; HMGA1; KCTD15; LDLRAP1; NRG1; SMG7; VKORC1L1; ACTN2; AIDA; AP3S1; BEND6; BNIP2; BNIP3L; C21orf91; CDC42EP2; CEP63; CUL4A; CXCL12; EBAG9; EIF2S3; GPR158; GRIN2A; HMGCLL1; HNRNPDL; HOXD10; KDM1A; KLF15; METTL9; MRGBP; MYO1C; NEUROD1; NREP; NRXN3; NSF; PDS5A; PIAS4; PLCB1; QKI; RCOR2; RNF165; SLC6A9; SRSF6; SSFA2; TADA2B; TAF12; TBX3; UBXN4; UNC79; USF2; VAX1; YBX1; ZFP36L2; |
| RORA | 12.19281664 | 1.592364902 | 5.0818E-08 | ABHD17A; ABHD17B; ARHGAP44; ARID4B; COL6A6; FGF9; HCN1; MAP3K7; NFE2L1; PIK3R1; PIM1; RAPGEFL1; SEMA3A; SORCS1; TANC1; ANP32B; ANP32E; ASPH; ATP6V1A; BACH2; BDNF; CAPZA1; CBL; CCNC; CLCN3; CLTC; CNN3; ELMO1; EYA4; FAM91A1; GPR137C; KTN1; LASP1; LRCH1; MTSS1; NBEA; NCK2; NDRG3; PDE7A; PEA15; RABGAP1L; RARB; SFPQ; SKIDA1; SLC10A7; STARD7; TPM3; USP33; VPS45; ZNF800; FBXO9; FRMD5; MARCKS; PALM2-AKAP2; ZFP91; BANP; CD274; ETFA; IRF6; LMO3; MSI2; NABP1; NPAS2; PBX3; ARGLU1; DGKB; GNG12; MACROD2; MYT1L; PABPC1L; PPM1B; SLC35F5; USF1; WAPAL; ATP8B2; GAB2; HIPK1; HM13; KCTD15; LARGE; MACF1; MLLT3; ONECUT2; PHC2; RNF5; SLC4A5; TSFM; ACSL6; ACVR1; ARHGAP42; ATP1B1; B3GALT2; CCNY; CHST1; CTDSP2; CXXC4; EPHA7; FAM196B; FBXL7; GPR158; GRM7; HOXD10; IDH1; IFT20; INPP5A; KCNAB3; LONRF3; LURAP1L; MARCH7; MBNL2; MTA3; MTDH; NECAP1; NRXN1; PDE1B; PLCB1; PTPN2; RAB11B; RAPGEF5; SLC17A6; SLC46A3; SLMAP; SSBP3; STK38; SWT1; SYT1; ZNF385D; ZNF516; SETD8; |
| KLF7 | 36.48393195 | 1.251118895 | 5.94921E-08 | ABHD17A; ARID4B; ARMC8; ATXN7L3; CELF2; COPS7B; CSPG5; DYNLL1; EIF5A2; FOXO1; GOLGA3; HAT1; MAP3K7; NFE2L1; PRRC2C; RAPGEFL1; SMOC1; ST6GALNAC6; TSC22D2; TXLNG; ABHD2; ACER2; ACTB; ADPGK; ANKRD34B; ANXA2; ARG2; ASPH; AZIN1; BPNT1; BSCL2; C2CD5; CBL; CDC42SE1; CDK4; CHSY1; CORO1C; CPEB1; CTBP2; CTTNBP2NL; CXCR4; DDX5; DGKZ; DLG4; ELF1; EPB41L4B; EYA4; FAM107B; FAM63B; FAM91A1; FBXL14; FBXO33; FNBP1L; FOSB; G6PD; GDF6; GLCCI1; HIAT1; HMGN1; HNRNPU; HSPD1; HSPE1; JUND; KCNJ2; KIF2A; KLF4; LASP1; LIN7C; LRRC59; MAN1C1; MBLAC2; MMD; MSANTD2; MXD1; NCL; NDRG3; NET1; NR1H3; NXT2; OSBPL7; PCDH17; POLR3G; PPIB; PTPRS; RAB11FIP1; RAB5A; RABEPK; RASA1; RNF138; RNF141; RYBP; SEC23B; SEC63; SEPT2; SKIDA1; SLC25A22; SLC25A30; SMARCB1; SMIM14; SNX2; SOX9; SPRED1; STARD7; STX12; SYN3; TAGLN2; TIGD6; TIMP3; TMEM55B; TNPO2; TPM3; USP33; VASP; WDR1; WDR61; XPO6; YRDC; YWHAQ; YWHAZ; ATP6V1G1; C6orf106; C9orf72; CBFB; CCDC6; CCNG1; CCNT2; CD2AP; EFNB2; FOXN2; IL6ST; KCNG3; KLF10; MAP1LC3B; MARCKS; MSX1; NMNAT3; PDIK1L; PTEN; RAP2C; SRSF7; TOMM70A; ZFP91; ARMCX2; DAZAP1; DBN1; DDX42; DPY30; EFS; FOXQ1; FTL; FUS; GRASP; INSM2; IRF6; KLF13; KLF5; MDK; MYL12A; PBX3; PCSK7; PHF1; PLK3; POLE4; PPCS; RAI2; RAP1A; RGS9BP; SCOC; SLC10A3; SLC28A3; SMIM15; STARD4; STAT4; SYNGR2; TSC22D4; TUSC3; YWHAH; ARHGEF10L; ATP10D; C16orf87; CELSR2; CHTOP; COL1A1; FAF1; FGD1; FOPNL; FRS2; GNG12; HMOX2; KCNA4; LTA4H; MBD5; NIPAL1; NNT; NOVA1; NTPCR; NUFIP2; PABPC1L; PELI3; PI4K2B; PIP5KL1; PLA2G3; PPM1B; RAB14; RAB23; RNF114; TFG; TM6SF1; UBE2G1; UBE2Q1; USF1; ACTR1A; AMMECR1L; APOLD1; ATG2B; ATP8B2; CAD; CAPN6; FKBP8; GAB2; GDI1; HM13; HMGA1; INPP5K; ISCA2; KCNA6; LDLRAP1; LUZP1; MACF1; NRG1; NUMBL; NYNRIN; ONECUT2; PCDH1; PPM1N; PPP2R5B; PRRT2; RAB37; RAB4B; RNF44; RPRD2; RTN2; SLC30A3; SLC5A2; SOX12; SRF; STOML1; TEAD3; TGFB1; THRA; VKORC1L1; AACS; AHR; AJUBA; ANKRD13A; AP3S1; B4GALT5; BAZ2A; BCAP31; C3orf58; CACFD1; CAPN2; CCNG2; CDC37L1; CDC42EP2; CEP63; CHST1; CSNK1A1; CTDSP2; CXXC4; DEXI; DFFB; DNAJB5; DUSP10; EBAG9; EFEMP2; EFNA3; EIF1; EPHA7; ERG; ESRRA; ESRRG; ETFB; EZH2; FAM126B; FAM98A; FHL3; FKBP4; FMNL2; FSTL1; GJC1; GORASP2; GRIN2A; HERPUD2; HK3; HLF; HLTF; HNRNPDL; HOXC4; ICMT; IDH1; IMPA2; ING2; INPP5A; KDM1A; KDM5B; KIT; KLF11; LEPROT; LMBR1L; LONRF3; LRRC10B; MBD6; MIA3; MITF; MOB4; MPC1; MSI1; MTA3; MTDH; MTF1; MYBPC3; NAB2; NCOA2; NKPD1; NREP; NRXN1; NUCKS1; PDE1B; PEX3; PIAS2; PIAS4; PLA2G15; PPP1R14B; PPP1R37; PPTC7; PRDM1; PRKAB1; PROX1; PRR16; PTGES2; PTPN2; PXN; QKI; RAB11B; RAB9A; RASIP1; RNF165; RNF38; RPGRIP1L; RPL28; RREB1; RRM2B; SLC1A5; SPTLC1; SPTY2D1; SRPR; SRSF6; SSFA2; SSR1; SWT1; SZRD1; TADA2B; TCF4; TFAP2A; TPCN1; WNT2B; WNT7A; YBX1; ZBTB7A; ZC3H6; ZFYVE16; ZNF385D; ZNF449; ZNF516; ZNF710; C17orf96; CYB561D1; ETNK2; GINS4; ITGA6; KIF3B; MAPK4; MTSS1L; SETD8; ZC3H4; |
| MEF2A | 17.20226843 | 1.429955745 | 2.02556E-07 | ARID4B; ARMC8; BTAF1; BTBD3; COPS7B; FGF9; HAT1; MAP3K7; PIK3R1; PRRC2C; TANC1; ARG2; ASPH; BACH2; BDNF; C1GALT1; CASK; CCNC; CHSY1; CITED2; CLCN3; CTBP2; DGKH; DLG4; EBPL; ELF1; ELMO1; ETS1; FBXW7; FN1; FNBP1L; FUBP1; GLCCI1; GLS; GPR137C; GPR6; HNRNPK; IGF1; KALRN; KCNIP3; KTN1; MAP4K3; MEOX2; MIER1; NBEA; NET1; NETO1; NR4A2; PAX3; PICALM; PLEKHA1; POGK; RNF145; RRBP1; SEC61A1; SKIDA1; SLC29A3; SLC44A1; SMAP1; SNAP25; SP2; TAGLN2; TMCC1; TTR; UST; VPS45; YPEL2; ARPC2; CBFB; CCNT2; CD2AP; CTNNBIP1; EVI5; NEK6; PALM2-AKAP2; RAP2C; TMEM39A; TOMM70A; VAV3; ARL8B; ARPP19; CD274; ETFA; MSI2; NPAS2; PBX3; SCOC; STAT4; TSC22D4; YWHAH; ARGLU1; ARHGEF10L; CACNA2D1; CHTOP; COL1A1; DGKB; DMRT2; FRMD3; HNF4G; IPO9; KCNA4; LGALSL; MAFB; MBD5; MTUS1; MYT1L; NNT; NTPCR; PPM1B; PPP1CB; PVALB; RGS7BP; SLC35F5; SLCO3A1; TBC1D8; UBE2G1; ZBTB18; ATP8B2; DIRAS1; EPHA10; HIPK1; LDLRAP1; MACF1; MLLT3; PHC2; SMG7; TEAD3; ACSL6; ACTN2; ACVR1; ADAM23; APPL2; B3GALT2; BCL2L13; BEND3; BLOC1S5; CA7; CTNNA3; CXXC4; DENND1A; DMRT3; DNAJB5; DR1; DUSP10; EN2; EPHA7; ESRRG; FBXL7; GRIP1; GRM7; KIT; LBX1; LINGO2; LUZP2; MARCH7; MBNL2; MITF; MYBPC3; NIPBL; NRXN1; PPARGC1A; PPTC7; PRDM1; PTGFRN; PXN; QKI; RAPGEF5; RCAN2; RREB1; SGPL1; SLMAP; SSFA2; ST3GAL3; STAC; STK3; SYT1; TWIST1; WBP1L; ZFPM2; ZNF385D; ZNF516; BCAS3; |
| MSX2 | 4.725897921 | 2.095808367 | 4.79814E-07 | RFFL; SORCS1; BET1; BLCAP; CBL; CCNC; ELMO1; FBXO33; FNBP1L; FUBP1; GCH1; HACE1; KALRN; MAB21L1; OSTF1; PEA15; SFPQ; TMEM178A; TNKS2; VAMP4; ZBTB8A; CCNG1; EVI5; LMO3; MSI2; NPAS2; PAPD5; PBX3; FKBP1A; HUNK; MBD5; MYT1L; PTPN12; APOLD1; ATG2B; LUZP1; ALDH1A2; BEND3; DCAF8; ESRRG; EZH2; GRAMD3; MITF; RNF38; SSFA2; ST3GAL3; TBC1D12; UBXN4; ZFPM2; KLHL14; |
| ELF1 | 6.899810964 | 1.799222366 | 6.27298E-07 | BTBD3; CTDSPL2; PIK3R1; SMAD2; ARPC3; BRI3BP; CLCN3; CTTNBP2NL; DGKZ; ELMO1; FAM91A1; FBXW7; GPD2; HOXB4; JARID2; KCNJ2; MAB21L1; MMD; NAB1; NR1H3; OSTF1; PHF6; RAP1B; RRAGC; SFPQ; SNX2; STX12; TMEM178A; ARPC2; ELOVL6; MARCKS; PDIK1L; CD274; FUS; GRASP; KLF13; RAC1; VIM; DCAF12; MBD5; NUFIP2; ZBTB18; HM13; KAZALD1; LDLRAP1; NRG1; PLEKHA2; RNF5; TGFB1; WASF2; ACVR1; CCNG2; CTDSP2; DENND1A; EPHA7; GRM7; HIC1; MARCH8; MITF; MYO1C; NCK1; NSF; PPARGC1A; PRDM1; SCRT2; SGPL1; SLC5A7; SSFA2; TFAP2C; TPCN1; TWIST1; WNT2B; KLHL14; |
| HOXB7 | 5.103969754 | 2.006767418 | 6.8575E-07 | ARHGAP5; DCBLD2; SEMA3A; SMAD2; SORCS1; TWF1; ACER2; ARFIP1; BLCAP; ELF1; EYA4; FBXW7; FNBP1L; IGF1; KALRN; PAX3; TMEM178A; UTRN; ZNF800; CCDC6; MARCKS; PTEN; XRCC5; ZFP91; KITLG; LMO3; NPAS2; RAI2; SMIM15; ARHGEF10L; KCNA4; NOL4; PTPN12; SCAI; ZBTB18; DYNLL2; LUZP1; NRG1; ONECUT2; BAZ1A; FMNL2; GRIP1; HERPUD2; MITF; NRXN1; NUCKS1; PPTC7; PRDM1; PRKAB1; RNF38; SCRT2; SSFA2; ST3GAL3; VMA21; |
| ARID3A | 12.38185255 | 1.510973132 | 7.82344E-07 | BTBD3; CDH7; FGF9; RFFL; SEMA3A; SMAD2; ADAM12; ANKRD29; C5orf51; CASK; CBL; CCNC; CD164; CDK14; CTBP2; ELF1; ELMO1; EYA4; FAM107B; FLI1; FN1; GJA1; GLS; GPD2; GPR6; HIAT1; KALRN; KCNJ2; MIER1; MIPOL1; MON2; MPP5; MPZL1; MTSS1; NBEA; NET1; PAX3; PDE7A; PIGF; RAB5A; RABGAP1L; RAP1B; RARB; RRAGC; RSPO3; SFPQ; SMIM14; SNAP25; SYN3; TMSB4X; TPK1; UTRN; YPEL2; ZBTB8A; MARCKS; PTEN; TAB3; VAV3; ARPP19; CNKSR2; HECTD2; KITLG; KLF5; LMO3; SCOC; ARGLU1; ARHGEF10L; CACNA2D1; CHL1; DES; DGKB; F10; MBD5; MYT1L; NOL4; NUFIP2; PPM1B; SEC61A2; YBX3; AAK1; IPO11; KAZALD1; MACF1; MLLT3; NRG1; PHC2; ACSL6; ACVR1; ADCY2; AIDA; ANO4; BAZ1A; BNIP2; CTNNA3; CXXC4; DCDC2; DENND1A; ERG; ESRRG; FAM126B; GPCPD1; GPR158; GRIP1; GRM7; IDH1; KDM1A; LINGO2; MAPK10; MBNL2; MBTPS2; MTA3; NR1H4; NRXN1; OSER1; PIAS2; PLCB1; PRDM1; RAPGEF5; RNF165; SLMAP; SSFA2; STAC; STK38; SYT1; TBC1D12; TCF4; TDRD7; UBXN4; WIF1; WNT7A; ZFPM2; |
| SRF | 5.765595463 | 1.903774177 | 8.21363E-07 | RASSF3; ACTB; AKAP12; ASPH; ATG13; FAM63B; FN1; FOSB; KALRN; KRAS; LRRC8A; MAP4K3; PDGFA; PICALM; RAB11FIP1; SFPQ; SNAP25; STX12; THBS1; TIMP3; TPM3; TPM4; UTRN; VPS45; WDR1; ARPC2; DNAJB4; BANP; IRF6; NPAS2; SCOC; CACNA2D1; MLIP; MYT1L; NOVA1; TM6SF1; YBX3; AAK1; FBRS; KCTD15; MACF1; MLLT3; PHC2; SRF; ACVR1; CAPN2; DNAJB5; DUSP10; ESRRG; FAM126B; HOXD10; LRRC10B; LURAP1L; MAPKAPK2; MITF; NREP; PPARGC1A; SCRT2; TCF4; TNC; MAPK4; |
| NFIC | 18.43100189 | 1.378130576 | 9.53964E-07 | ARHGAP44; ARID4B; CDH7; COPS7B; DCBLD2; PIK3R1; PIM1; PRRC2C; SMARCD2; SMOC1; SORCS1; ST5; STK4; ADAM12; ANP32E; ANXA4; ARCN1; AXL; AZIN1; BDNF; BET1; BLCAP; CALM2; CBL; CDC42SE1; CXCL11; DLG4; ELMO1; EPB41L4B; ETS1; FGF17; FLI1; FOSB; GJA1; GLS; HACE1; HSP90B1; KANK4; KLF4; KRAS; LRCH1; MATR3; MIPOL1; MMD; MOB3C; MTX1; NCK2; NFATC2; NR4A2; PAX3; PEA15; PTPRS; RARB; SEC23B; SEC61A1; SEC63; SERP1; SKIDA1; SLC44A1; SNAP25; SP2; SPRED1; TAGLN2; TGIF2; THBS1; TMEM135; TMEM178A; USP33; UTRN; XPO6; YWHAZ; ZNF148; CTNNBIP1; ELOVL6; EVI5; FRMD5; IL6ST; KLF10; MSX1; NEK6; PTEN; RAP2C; UBE2I; ZFP91; DBN1; IRF6; KCNS3; KLF5; KLHDC9; NAA20; PBX3; PLEKHA5; RAI2; SCOC; TSC22D4; VIM; CHL1; CHTOP; COL1A1; DES; FRMD3; FRS2; LGALSL; MAFB; MBD5; N4BP2L1; NOL4; NUFIP2; PELI3; PPM1B; SEPT8; SLC30A9; UBE2G1; YBX3; AAK1; ATP8B2; BBC3; FBRS; HIPK1; HOXB6; JDP2; LARGE; LRFN1; MLLT3; NRG1; POLL; PPAN; PPFIA3; SLC30A3; SMG7; STOML1; TEAD3; TGFB1; ZC3H7B; ACSL6; AHCYL1; AKT2; ANO4; AP3S1; ATP1B1; BAZ1A; BEND6; CA7; CD69; CD83; CEND1; CHGA; DENND1A; DNAJB5; DSP; DUSP10; EFNA3; EIF4G2; EPHA5; FAM98A; GRIN2A; GRM7; HMGN3; ING2; MAPKAPK2; MBD6; MBNL2; MITF; MPP1; NCOA2; NRXN1; NUCKS1; PDE1B; PPTC7; PRKAA1; PXN; RAB9A; RAPGEF5; RASIP1; RCOR2; RNF165; SASH3; SCRT2; SGPL1; SIK1; SIPA1L2; SIRT6; SLAMF9; SLC12A2; SLC43A2; SRC; SSBP3; SYT1; TPCN1; ZFP36L2; ZNF710; ATP2B2; ETNK2; KLHL14; ZC3H4; |
| PDX1 | 4.914933837 | 2.015570632 | 9.63573E-07 | CDH7; MYLK2; TXLNG; ARID2; BACH2; CASK; DHX15; ELMO1; FBXW7; FLI1; HNRNPU; LRCH1; NBEA; PEA15; PIGF; PLEKHA1; RRAGC; UTRN; CTNNBIP1; FRMD5; MARCKS; RAP2C; TMEM39A; XRCC5; ARPP19; KLHDC9; LMO3; NPAS2; PBX3; FGD1; MTUS1; ACTR1A; HMGA1; IPO11; PHC2; TSFM; ACVR1; CTNNA3; GREM2; HERPUD2; KDM4A; MARCH7; NUCKS1; PRDM1; SH3TC1; SIPA1L2; SSBP3; ST3GAL3; TNC; ZNF385D; ZNF516; BCAS3; |
| E2F1 | 11.8147448 | 1.517016978 | 1.17303E-06 | ARHGAP44; ATXN7L3; CTDSPL2; MAP3K7; PIM1; PRRC2C; SMAD2; TANC1; BACH2; CALM2; CDK14; CEP44; CTBP2; DHX15; EHMT2; EIF1AX; GPD2; GPR137C; HNRNPK; HOXB4; HS3ST3B1; KALRN; KCNIP3; MATR3; MPP5; MTSS1; NCL; NXT2; PTBP1; PTMA; SH3GL1; SLC10A7; SMARCB1; SRSF1; TMX1; UTRN; YWHAQ; C9orf72; CCNT2; FOXN2; PALM2-AKAP2; SRSF7; UBE2I; VAV3; CDK13; DLX1; EFS; FUS; GNAI1; NKX2-4; PBX3; POLE4; RAD51; RAP1A; TMEM108; VPS37B; ARGLU1; ARL5A; CHL1; COL1A1; FRMD3; GNAQ; LEMD3; MAFB; MTUS1; NUFIP2; PPM1B; PVALB; SLCO3A1; UBE2Q1; WNK4; APOLD1; CAD; CCAR2; HMGA1; IPO11; KAZALD1; KCNA6; KCTD15; MACF1; PCDH1; RNF5; SLC30A3; ABHD6; ATP1B1; C18orf25; CD83; CEP63; CHST1; CSTF2T; CUL4A; DHX40; EBAG9; EIF4G2; ELOVL2; EN2; FXYD6; HLTF; HNRNPDL; LUZP2; MSI1; NAB2; NIPBL; NRXN1; PDE1B; PDS5A; PHF3; RAB11B; RANBP1; RNF165; SIRT6; SLC1A5; SRSF6; SSBP3; SSFA2; SUZ12; SYNDIG1; VAX1; WNT7A; ZFP36L2; ZNF385D; ZNF516; ITGA6; MAPK4; SGSM2; |
| STAT1 | 12.38185255 | 1.497792662 | 1.25571E-06 | ARHGAP44; BTBD3; DCBLD2; EIF5A2; HAT1; RAPGEFL1; RFFL; SORCS1; TANC1; ACTB; ADAM12; ADPGK; ARPC3; ASPH; AZIN1; BDNF; BET1; CBL; CD164; CNN3; CXCL11; EHMT2; EIF4E; ELMO1; FGF17; FN1; HACE1; HOXB4; HSPD1; HSPE1; IGF1; MAP4K3; MOB3C; MON2; NAB1; NECAB1; NFATC2; NR4A2; PICALM; RABGAP1L; RAP1B; RARB; RASA1; SH3BGRL; SNAP25; STX12; THBS1; TIGD6; TMCC1; TSPAN4; UTRN; VASP; VPS45; WDR1; ZNF148; ARPC2; CCDC6; IL6ST; NEK6; BANP; FUS; GNAI1; KLF5; MYL12A; NPAS2; RAI2; SMIM15; VIM; ARHGEF10L; ATP10D; CACNA2D1; FKBP1A; GNG12; HNF4G; MBD5; NUFIP2; PLA2G3; SEPT8; TNFSF11; YBX3; AAK1; EPHA10; KMT2B; MACF1; MLLT3; PCDH1; PPM1N; RTN2; ACSL6; ACVR1; ADAM23; AHCYL1; AKT2; ARHGAP42; ATPAF1; C21orf91; CEP63; DENND1A; DTX2; EHBP1L1; EPHA5; ESRRG; FAM126B; FAM98A; FBXL7; GABRA1; GREM2; HERPUD2; HOXC4; IFT20; LAPTM4B; LINGO2; MAPK10; MBD6; MED27; MSI1; NSF; PLCB1; PRDM1; PTGFRN; RAB11B; SLC1A5; SNX25; SPHK2; SPTY2D1; STK3; TBX3; TNFAIP1; VAX1; ZFYVE16; ZNF385D; |
| SOAT1 | 12.38185255 | 1.497792662 | 1.25571E-06 | ARHGAP44; BTBD3; DCBLD2; EIF5A2; HAT1; RAPGEFL1; RFFL; SORCS1; TANC1; ACTB; ADAM12; ADPGK; ARPC3; ASPH; AZIN1; BDNF; BET1; CBL; CD164; CNN3; CXCL11; EHMT2; EIF4E; ELMO1; FGF17; FN1; HACE1; HOXB4; HSPD1; HSPE1; IGF1; MAP4K3; MOB3C; MON2; NAB1; NECAB1; NFATC2; NR4A2; PICALM; RABGAP1L; RAP1B; RARB; RASA1; SH3BGRL; SNAP25; STX12; THBS1; TIGD6; TMCC1; TSPAN4; UTRN; VASP; VPS45; WDR1; ZNF148; ARPC2; CCDC6; IL6ST; NEK6; BANP; FUS; GNAI1; KLF5; MYL12A; NPAS2; RAI2; SMIM15; VIM; ARHGEF10L; ATP10D; CACNA2D1; FKBP1A; GNG12; HNF4G; MBD5; NUFIP2; PLA2G3; SEPT8; TNFSF11; YBX3; AAK1; EPHA10; KMT2B; MACF1; MLLT3; PCDH1; PPM1N; RTN2; ACSL6; ACVR1; ADAM23; AHCYL1; AKT2; ARHGAP42; ATPAF1; C21orf91; CEP63; DENND1A; DTX2; EHBP1L1; EPHA5; ESRRG; FAM126B; FAM98A; FBXL7; GABRA1; GREM2; HERPUD2; HOXC4; IFT20; LAPTM4B; LINGO2; MAPK10; MBD6; MED27; MSI1; NSF; PLCB1; PRDM1; PTGFRN; RAB11B; SLC1A5; SNX25; SPHK2; SPTY2D1; STK3; TBX3; TNFAIP1; VAX1; ZFYVE16; ZNF385D; |
| MSX1 | 3.497164461 | 2.28008192 | 1.99394E-06 | ARHGAP44; RFFL; SEMA3A; SORCS1; CDK14; EYA4; FBXW7; KALRN; RARB; TMEM178A; ZNF800; MARCKS; PTEN; KLF5; LMO3; NPAS2; PBX3; SMIM15; ARGLU1; KCNA4; MYT1L; PTPN12; ZBTB18; NRG1; BAZ1A; CXXC4; CYYR1; DENND1A; ESRRG; GRM7; LBX1; MITF; PPARGC1A; PRDM1; SCRT2; SSFA2; ATP2B2; |
| RREB1 | 18.90359168 | 1.349939206 | 2.80572E-06 | ARHGAP5; CELF2; COPS7B; FGF9; MAML3; MAP3K7; MYLK2; PIM1; RAPGEFL1; TANC1; ACTB; ANP32B; ANP32E; ARID2; BDNF; BET1; BLCAP; CASK; CDK4; CLTC; CPEB1; CTBP2; DGKZ; DLG4; ELF1; ELMO1; ETS1; FLI1; FN1; FNBP1L; FUBP1; GCH1; GDF6; GJA1; GPD2; GPR137C; GPR6; HNRNPK; HOXB4; JARID2; LASP1; NAB1; NETO1; NR4A2; OSBPL7; OTX2; PEA15; PICALM; RARB; RNF14; SFPQ; SKIDA1; SLC35F1; SMARCB1; SNAI2; SNAP25; SP2; TAGLN2; TMEM135; UST; VAMP2; YRDC; YWHAZ; ZNF148; ARPC2; ELOVL6; FOXN2; KLF10; MARCKS; NEK6; NRG3; PALM2-AKAP2; VAV3; ARMCX2; DBN1; DLX1; FOXQ1; FTL; GRASP; HECTD2; IRF6; KCNS3; KLHL36; LMO3; MDK; PLK3; RAI2; SCOC; SLC28A3; TSC22D4; VIM; ARHGEF10L; DCAF12; DES; DGKB; FAF1; HNF4G; MACROD2; MBD5; NNT; NOL4; NUFIP2; PIP5KL1; PLA2G3; PTPN12; SEPT8; WNK4; ZBTB18; ADAMTS10; APOLD1; BBC3; CAPN6; CNTFR; DYNLL2; EPHA10; HIPK1; INPP5K; KAZALD1; KCNA6; LARGE; LDLRAP1; LYPLA2; MACF1; NUMBL; ONECUT2; PCDH1; PPFIA3; RIMS4; RNF5; SLC30A3; THRA; TSFM; ACSL6; ACTN2; ADAM23; AKT2; ATP1B1; ATPAF1; BAZ2A; BEND6; C11orf87; C6orf47; CA7; CCNI; CD69; CHST1; CTDSP2; CXCL12; DCAF8; DENND1A; DHX40; DUSP10; EFNA3; ESRRG; FAM168A; FHL3; FMNL2; FXYD6; GABRA1; GRIN2A; HOXC4; KLHL10; LBX1; MAPKAPK2; MBD6; MITF; MSI1; MYO1C; NECAB3; NEUROD4; NRXN3; NUCKS1; OSER1; PLA2G15; PROX1; PRR16; PXN; RAB11B; RAB9A; RNF165; RREB1; SAMD8; SASH3; SCRT2; SRC; SSBP3; STAC; SYT1; TBX3; TCF4; TFAP2A; VARS; WBP1L; WNT2B; WNT7A; ZFP36L2; ZFPM2; ETNK2; SGSM2; ZC3H4; |
| DBX2 | 5.009451796 | 1.8730647 | 6.96466E-06 | ARHGAP44; BTBD3; FGF9; MAP3K7; SEMA3A; SORCS1; SP5; TANC1; ABCB7; BLCAP; DDX5; ELF1; GLS; GPD2; KALRN; RARB; SKIDA1; TMEM178A; UTRN; FOXN2; MARCKS; LMO3; NPAS2; RAI2; VIM; FRMD3; KCNA4; MLIP; MYT1L; NOVA1; PTPN12; ZBTB18; HIPK1; KCTD15; LUZP1; MLLT3; BAZ1A; CXXC4; DENND1A; EPHA5; ESRRG; FAM126B; GRM7; HTR2C; NRXN1; PPARGC1A; PRKAB1; RCAN2; RNF38; SCRT2; SSFA2; TCF4; ZNF385D; |
| HMX1 | 3.402646503 | 2.163015178 | 9.31119E-06 | ARHGAP44; BTBD3; FOXO1; SEMA3A; TANC1; CDK14; EDN1; EYA4; OTX2; PEA15; PHF6; RABGAP1L; RARB; RSPO3; YRDC; CTNNBIP1; MARCKS; PALM2-AKAP2; DLX1; IRF6; KITLG; LMO3; ARGLU1; FRMD3; NRG1; EPHA5; ERG; ESRRG; GRM7; MITF; MTA3; SSBP3; SYT1; TCF4; ATP2B2; KLHL14; |
| HOXB9 | 6.049149338 | 1.718427589 | 1.3999E-05 | CDH7; FGF9; HAT1; PIK3R1; SEMA3A; SMOC1; SP5; ACTB; BLCAP; C5orf51; CAPZA1; CCNC; CDK14; CTBP2; FBXW7; FUBP1; GLCCI1; HACE1; HNRNPK; HOXB4; JARID2; LRCH1; MAN1C1; NAB1; NDFIP2; PEA15; PHF6; RARB; SKIDA1; SLC25A30; THBS1; ZNF800; ARPC2; DNAJB4; MARCKS; NMNAT3; DLX1; FOXQ1; GNAI1; KLF5; LMO3; PBX3; SPOPL; SEC61A2; PLEKHA2; SLC4A5; ANO4; DCAF8; DMRT3; DNAJB5; ERG; ESRRG; FAM98A; GRM7; HOXD10; MAPK10; MTA3; NRXN1; PLCB1; PRDM1; RNASE6; SYT1; TNC; TSNAX; |
| HOXD8 | 8.31758034 | 1.564485687 | 1.57695E-05 | AFF3; ARHGAP44; FGF9; MAP3K7; MYLK2; PIM1; RFFL; SEMA3A; SORCS1; SRSF3; TWF1; CBL; CCNC; CTBP2; ELF1; ELMO1; EYA4; FBXO33; GLS; MTSS1; NBEA; NETO1; PIGF; RAB5A; RARB; RSPO3; SDCBP; SFPQ; SLC10A7; SLC35F1; UTRN; VPS45; ZNF281; ARPC2; CCNT2; CTNNBIP1; PTEN; ZFP91; ARPP19; CDK13; CNKSR2; KITLG; NPAS2; RAI2; RAP1A; ARGLU1; CHL1; FRMD3; KCNA4; MBD5; MLIP; MTHFD1L; MYT1L; NNT; PPM1B; PTPN12; SCN1A; SEPT8; HIPK1; NRG1; AIDA; BAZ1A; BEND6; CNEP1R1; CTDSP2; CUL4A; CXXC4; CYYR1; DENND1A; ERG; GABRA1; GRIP1; HOXD10; HTR2C; MAPK10; MBNL2; MITF; NRXN1; NSF; PLCB1; PPARGC1A; PRKAB1; SLC17A6; SSBP3; SSFA2; STK3; SYT1; ZNF385D; |
| POU3F2 | 8.128544423 | 1.571564546 | 1.66729E-05 | AFF3; ARHGAP44; FOXO1; SEMA3A; SORCS1; CASK; CDK14; CDK4; CLTC; ELMO1; EYA4; FBXW7; FN1; FNBP1L; FUBP1; GLS; KALRN; MAB21L1; MAP4K3; MIPOL1; NBEA; OTX2; PDE7A; RABGAP1L; RARB; RSPO3; SMAP1; TMEM178A; UST; UTRN; ZNF800; CCNG1; MARCKS; PTEN; VAV3; CDK13; DLX1; HECTD2; KITLG; LMO3; NPAS2; PAPD5; SMIM15; VIM; ARHGEF10L; FAM169A; KCNA4; MBD5; MTUS1; MYT1L; NONO; NUFIP2; PPP1CB; LARGE; MACF1; NRG1; PHC2; ACTN2; AJUBA; ARHGAP42; BAZ1A; CXXC4; DENND1A; ESRRG; FAM126B; GRIP1; GRM7; HERPUD2; LBX1; MAPK10; MBNL2; MITF; NREP; NRXN1; PPARGC1A; PRDM1; PRKAB1; RAPGEF5; SCRT2; SLMAP; SSBP3; SSFA2; TAF7L; TFAP2C; TNC; ZFPM2; |
| TFAP4 | 12.00378072 | 1.430508148 | 1.82503E-05 | BTBD3; RAPGEFL1; ST5; STK4; TANC1; ACTB; ARFIP1; AZIN1; BACH2; CDC42SE1; CDK4; CLTC; COL4A3BP; CTBP2; EIF4E; EYA4; FBXL14; FGF17; FLI1; GLCCI1; HELZ2; HSP90B1; IGF1; JARID2; KALRN; KCNJ2; KLF4; MEOX2; MIER1; MTX1; NBEA; NFATC2; SNAI2; SNAP25; SPRED1; TACR1; TAGLN2; TMEM178A; TMSB4X; TNPO2; TPM4; TSPAN4; UTRN; YWHAZ; FRMD5; KLF10; NEK6; PALM2-AKAP2; TMEM39A; CDK13; CNKSR2; GRASP; KCNS3; KLF13; MYL12A; RAI2; RBM24; ARHGEF10L; CACNA2D1; CHTOP; COL1A1; DES; KCNA4; MLIP; NOL4; NOVA1; PABPC1L; PELI3; RAB14; SLC35G1; USF1; ZBTB18; DYNLL2; DYRK1B; EPHA10; HM13; MACF1; NUMBL; POLL; PPM1N; PPP2R5B; RAB37; RPRD2; RTN2; SNX20; THRA; ACSL6; ACVR1; AIDA; AJUBA; AKT2; ANKRD13A; ANXA7; BCAP31; CCNG2; CTDSP2; CXCL12; DTX2; DUSP10; DVL2; EPHA5; GLIS2; GPCPD1; GRIP1; GRM7; HAPLN2; HOXD10; KDM4A; KDM5B; LURAP1L; MBNL2; MITF; NRXN1; PPTC7; RWDD4; SAMD8; SLC5A7; TMEM229B; WBP1L; WNT2B; ZFPM2; ZFYVE16; ZMAT4; ATP2B2; ITGA6; KLHL14; SETD8; |
| BARX1 | 4.631379962 | 1.849591212 | 2.1538E-05 | AFF3; DCBLD2; TSC22D2; ARG2; BACH2; EHMT2; EYA4; GJA1; GLCCI1; HACE1; KALRN; MAB21L1; MXD1; OTX2; PRKRIR; RSBN1; TACR1; TMEM178A; UTRN; CTNNBIP1; EVI5; MSX1; NEK6; KITLG; NPAS2; SPOPL; GNAQ; MYT1L; WAPAL; LDLRAP1; PHC2; ESRRG; FMNL2; GORASP2; GRAMD3; GRIP1; IFT20; MBD6; PPTC7; RNF38; SIPA1L2; SSBP3; ST3GAL3; TNFAIP1; UBXN4; VAX1; ZFPM2; ZMAT4; KLHL14; |
| TCF3 | 18.43100189 | 1.313732252 | 2.18737E-05 | ARMC8; CDH7; DCBLD2; FGF9; RFFL; SMOC1; ST5; TANC1; TXLNG; ZNF740; ADAM12; AKAP12; ANP32B; BDNF; BLCAP; CDC42SE1; CDON; CERS2; CITED2; CLTC; CORO1C; CPEB1; CTBP2; CTTNBP2NL; DGKZ; DLG4; E2F5; ELF1; ELMO1; EYA4; FBXW7; FGF17; FLI1; FNBP1L; GLCCI1; HACE1; HELZ2; HNRNPU; IGF1; KALRN; KCNIP3; KCNJ2; LRCH1; MTSS1; MXD1; NETO1; NFATC2; NR4A2; OSBPL7; OTX2; PCDH17; PDE7A; PEA15; PFN2; RAB11FIP1; SMAP1; SMARCB1; SNAI2; SNAP25; STC2; TACR1; TERF2; USP33; VASP; YPEL2; ZC2HC1C; CTNNBIP1; FRMD5; KLF10; MAP3K3; NEK6; NMNAT3; PALM2-AKAP2; PDIK1L; SEMA4B; ARMCX2; DBN1; DDX42; DESI2; DLX1; EFS; GRASP; IRF6; LMO3; NPAS2; PBX3; RBM24; SCOC; SLC10A3; SLC28A3; TSC22D4; CACNA2D1; CELSR2; CHL1; CHTOP; DMRT2; FAM169A; KCNA4; LSM5; MLIP; MTUS1; NOL4; PABPC1L; PPM1B; SCAI; SLCO3A1; TFG; TM6SF1; USF1; AAK1; ATG2B; ATP8B2; BBC3; DYRK1B; GAB2; HIPK1; HM13; HMGA1; INPP5K; LARGE; LUZP1; MACF1; NUMBL; PHC2; PLEKHA2; POLL; PPM1N; PPP2R5B; PRELP; RIMS4; RTN2; SLC30A3; THRA; TSFM; ZC3H7B; ACSL6; ACVR1; AJUBA; ANKRD13A; ATP1B1; BCAP31; C11orf87; C18orf25; CA7; CD83; CHST9; CTDSP2; DENND1A; DNAJB5; DSP; DTX2; EFEMP2; EFNA3; EHBP1L1; ERG; ESRRG; EZH2; FAM98A; FHL3; GORASP2; GREM2; GRIN2A; HERPUD2; HNRNPDL; IFT20; ING2; KDM4A; KIT; LINGO2; PCGF5; PDE1B; PLCB1; PPP1R14B; PPTC7; PROX1; PTGES2; PXN; RAPGEF5; RCAN2; RCOR2; RPL28; SAMD8; SCRT2; SLC35A4; ST3GAL3; TCF4; TMEM229B; TNFAIP1; WNT2B; WNT7A; ZCCHC2; ZNF710; ATP2B2; ITGA6; KLHL14; |
| NKX6-1 | 12.9489603 | 1.402853466 | 2.19491E-05 | ARMC8; BTBD3; COPS7B; FGF9; FOXO1; MAP3K7; PIK3R1; RFFL; SEMA3A; SORCS1; TWF1; ANXA4; BACH2; BDNF; CASK; CDK14; CLTC; CTBP2; CXCR4; DGKH; EDN1; ELMO1; EYA4; FLI1; FN1; FNBP1L; GLS; HNRNPK; JARID2; KALRN; LRCH1; MAB21L1; MTSS1; NBEA; NFATC2; OTX2; PCDH17; PDE7A; PHF6; RARB; RNF111; SH3BGRL; SKIDA1; SLC25A30; TERF2; TMEM178A; TTR; UTRN; ZC2HC1C; CCNT2; CD2AP; CTNNBIP1; DLG5; DNAJB4; MAP3K3; MARCKS; PALM2-AKAP2; PTEN; RAP2C; VAV3; XRCC5; ZFP91; CNKSR2; IRF6; KITLG; KLF5; LMO3; NABP1; PBX3; RAI2; SCOC; VIM; ARGLU1; ARHGEF10L; CACNA2D1; DES; DGKB; FRMD3; ICOS; KCNA4; MACROD2; MLIP; MTHFD1L; MYT1L; NOVA1; NTPCR; PTPN12; RAB14; HIPK1; LUZP1; MACF1; NRG1; ONECUT2; PHC2; ACSL6; ADAM23; AHR; BAZ1A; BNIP2; CTNNA3; CXXC4; DCAF8; DENND1A; EPHA5; ESRRG; FAM98A; GABRA1; GORASP2; GRIP1; GRM7; HOXC4; ING2; KDM1A; KDM4A; LBX1; LINGO2; LUZP2; MAPK10; MTA3; NRXN1; PPARGC1A; PPTC7; PRDM1; PRKAB1; PTPN2; SCRT2; SIPA1L2; SLMAP; SSBP3; SSFA2; SYT1; TCF4; VMA21; WNT7A; ZFPM2; ZNF385D; ITGA6; |
| EOMES | 3.780718336 | 1.982103922 | 2.52134E-05 | AFF3; FGF9; PIK3R1; SEMA3A; SP5; FN1; GLCCI1; GPD2; KIF2A; NR4A2; PDGFA; SMARCB1; TMCC1; YPEL2; YRDC; EVI5; NEK6; NRG3; CDH20; CNKSR2; KIAA1429; MYT1L; NONO; CNTFR; GAB2; LARGE; MLLT3; PLEKHA2; SLC30A3; ACSL6; ACTN2; AHCYL1; ALDH1A2; ESRRG; FMNL2; GABRA1; NCOA2; NUCKS1; RCAN2; UGCG; |
| ATF1 | 10.01890359 | 1.473769867 | 2.82727E-05 | CSPG5; CTDSPL2; FGF9; SRSF3; TSC22D2; ZNF740; ADAM12; ARG2; BACH2; BET1; BRI3BP; C1GALT1; C5orf51; CALM2; CDC42; CHSY1; CLCN3; DLG4; FN1; FOSB; GPR6; IGF1; JUND; KCNA5; LRRC8A; MSANTD2; NINJ1; PDE7A; PTPRS; SLC10A7; SNAI2; STX12; TACR1; TGIF2; TNKS2; TPM4; VASP; CD2AP; PALM2-AKAP2; TMEM39A; BANP; DDX42; IRF6; MSI2; SLC10A3; VPS37B; YWHAH; CHL1; COX4I1; ESYT2; FAF1; MAPK1IP1L; NIPAL1; NOL4; SLC35F5; HM13; IPO11; KCTD15; LARGE; RNF44; TSFM; ATP1B1; BNIP3L; C11orf87; C7orf31; CCNI; CHGA; CSTF2T; DUS2; DUSP1; EZH2; GPCPD1; GPRC5A; HLF; HTR2C; IFT20; INPP5A; KLF11; MAFK; MAPK10; MBNL2; NEUROD4; NRXN1; PAQR3; PDLIM3; PPARGC1A; PRDM1; PXN; RAB11B; RAPGEF5; SIK1; SLC5A7; SPHK2; TBX3; TNFAIP1; UGCG; YTHDC1; ZBTB21; ZBTB7A; ZCCHC2; ZNF516; ZNF710; ATP2B2; C17orf96; SGSM2; ZC3H4; |
| PLAU | 10.01890359 | 1.473769867 | 2.82727E-05 | CSPG5; CTDSPL2; FGF9; SRSF3; TSC22D2; ZNF740; ADAM12; ARG2; BACH2; BET1; BRI3BP; C1GALT1; C5orf51; CALM2; CDC42; CHSY1; CLCN3; DLG4; FN1; FOSB; GPR6; IGF1; JUND; KCNA5; LRRC8A; MSANTD2; NINJ1; PDE7A; PTPRS; SLC10A7; SNAI2; STX12; TACR1; TGIF2; TNKS2; TPM4; VASP; CD2AP; PALM2-AKAP2; TMEM39A; BANP; DDX42; IRF6; MSI2; SLC10A3; VPS37B; YWHAH; CHL1; COX4I1; ESYT2; FAF1; MAPK1IP1L; NIPAL1; NOL4; SLC35F5; HM13; IPO11; KCTD15; LARGE; RNF44; TSFM; ATP1B1; BNIP3L; C11orf87; C7orf31; CCNI; CHGA; CSTF2T; DUS2; DUSP1; EZH2; GPCPD1; GPRC5A; HLF; HTR2C; IFT20; INPP5A; KLF11; MAFK; MAPK10; MBNL2; NEUROD4; NRXN1; PAQR3; PDLIM3; PPARGC1A; PRDM1; PXN; RAB11B; RAPGEF5; SIK1; SLC5A7; SPHK2; TBX3; TNFAIP1; UGCG; YTHDC1; ZBTB21; ZBTB7A; ZCCHC2; ZNF516; ZNF710; ATP2B2; C17orf96; SGSM2; ZC3H4; |
| MYC | 10.39697543 | 1.460374103 | 2.92779E-05 | PIM1; STK4; TXLNG; AKAP12; ANP32B; ARG2; BDNF; BSCL2; C5orf51; CD164; CDK4; CPEB1; CTBP2; DDX5; EDN1; EIF4E; FBXW7; GJA1; HELZ2; HIAT1; KLF4; MBLAC2; MXD1; MXD4; NBEA; NCL; NET1; NME1-NME2; NME2; NR4A2; PFN2; PICALM; POGK; POLR3G; PTMA; PTPRS; RYBP; SEPT2; TMEM55B; TNPO2; TSPAN4; YPEL2; ZNF800; ATP6V1G1; FOXN2; NMNAT3; PTEN; DAZAP1; DDX42; FUS; MYL12A; NABP1; PPCS; SYNGR2; TMEM108; APEX1; CELSR2; MBD5; MLIP; MTHFD1L; MTUS1; NNT; YBX3; AMMECR1L; DYNLL2; IPO11; LYPLA2; PHC2; RNF44; SOX12; WASF2; ABHD6; ACVR1; AJUBA; BEND6; BLOC1S5; CCNG2; DHX40; DNAJB5; DVL2; EN2; ESRRA; HNRNPDL; HOXD10; LONRF3; MBTPS2; MTDH; NAB2; NEUROD1; NKAIN1; NRXN1; OSGEP; PIAS4; PPP1R14B; RANBP1; RAPGEF5; SASH3; SLC6A9; SSR1; SUGP2; TFAP2A; TNC; TPCN1; TWIST1; UNC79; XPO4; ZNF710; BCAS3; C17orf96; MTSS1L; |
| LHX3 | 11.34215501 | 1.429862442 | 3.23701E-05 | ARHGAP44; BTAF1; BTBD3; PIK3R1; SEMA3A; SMAD2; SORCS1; ABCB7; ARFIP1; ARID2; ASPH; BLCAP; CASK; CDK14; CDK4; CNN3; ELMO1; EYA4; FAM107B; FAM63B; FBXO33; FBXW7; GJA1; GPD2; GPR6; HNRNPK; KALRN; LRCH1; MAB21L1; MXD1; NBEA; NETO1; NXT2; OTX2; PAX3; RAB5A; RABGAP1L; RARB; RSPO3; SEC63; SLC25A30; SMAP1; SP2; TMEM178A; UTRN; YRDC; ZNF281; MARCKS; NRG3; PTEN; TAB3; VAV3; ZFP91; HECTD2; KLF5; LMO3; SPOPL; VIM; ARHGEF10L; CACNA2D1; CHL1; DES; FAM120A; FAM169A; FRMD3; KCNA4; MBD5; MTUS1; MYT1L; NOVA1; PPM1B; PPP1CB; SCAI; ZBTB18; AAK1; CAD; HIPK1; HMGA1; JDP2; MACF1; MLLT3; NRG1; ONECUT2; PHC2; ACTN2; ANO4; B3GALT2; BAZ1A; CCNY; CXXC4; DENND1A; ERG; ESRRG; FAM126B; FAM98A; GORASP2; GRIP1; GRM7; HOXC4; LBX1; MAPK10; MBNL2; MTA3; NREP; NRXN1; PDLIM3; PPARGC1A; PRKAB1; PTPN2; RNF38; RRM2B; SCRT2; SLMAP; SSBP3; SSFA2; ST3GAL3; SYT1; TNC; WNT2B; ZNF385D; |
| EN1 | 5.293005671 | 1.732750843 | 3.8001E-05 | AFF3; ARHGAP44; SEMA3A; SORCS1; ABCB7; BLCAP; ELMO1; EYA4; FBXW7; GJA1; KALRN; LRCH1; MAB21L1; MIPOL1; PDE7A; RARB; TMEM178A; UTRN; ZNF800; EVI5; MARCKS; CDH20; CDK13; KITLG; KLF5; LMO3; NPAS2; SPOPL; VIM; ARGLU1; CACNA2D1; MBD5; MLIP; MTUS1; MYT1L; NOL4; NTPCR; LUZP1; ANO4; CXXC4; ESRRG; FAM126B; FMNL2; GRM7; MBD6; METTL9; MITF; MTA3; PRDM1; PRKAB1; RNF38; SCRT2; SLMAP; SRC; ST3GAL3; VMA21; |
| ESX1 | 3.497164461 | 2.005796663 | 3.8737E-05 | ARHGAP44; RFFL; SORCS1; BDNF; CDK14; CDK4; GPR137C; KALRN; KRAS; MAB21L1; PDE7A; PLEKHA1; SKIDA1; TMEM178A; UTRN; ZNF800; PTEN; LMO3; NPAS2; SMIM15; VIM; KCNA4; NOVA1; CAPN6; JDP2; LUZP1; BAZ1A; CXXC4; DENND1A; ERG; ESRRG; HERPUD2; MITF; PRDM1; PRKAB1; RNF38; KLHL14; |
| HOXA5 | 8.979206049 | 1.49532256 | 4.32981E-05 | AFF3; CTDSPL2; DCBLD2; MAP3K7; SMOC1; ACER2; ASPH; BACH2; CASK; CDK4; CDON; CTTNBP2NL; DLG4; ELMO1; EYA4; FAM155A; FBXW7; FN1; GJA1; HNRNPK; HOXB4; IGF1; KALRN; MAB21L1; MAP4K3; MSANTD2; NBEA; NETO1; PIGF; RABGAP1L; RARB; SEC23B; SH3BGRL; SKIDA1; SLC29A3; SMAP1; SNAP25; SP2; TMEM178A; UTRN; ZNF148; ZNF281; ZNF800; EVI5; PTEN; SRSF7; XRCC5; DESI2; FUS; GNAI1; KLF5; LMO3; NKX2-4; NPAS2; PBX3; TMEM108; CACNA2D1; MBD5; MTUS1; MYT1L; N4BP2L1; PTPN12; SEPT8; TNFSF11; LDLRAP1; MACF1; PCDH1; PHC2; PLEKHA2; SMG7; ACVR1; CDC37L1; CYYR1; DENND1A; EFNA3; ENHO; ERG; ESRRG; FMNL2; GRAMD3; MAPK10; MBNL2; MITF; NREP; NRXN1; PPARGC1A; PPTC7; PRDM1; RAPGEF5; RNF38; SSBP3; ST3GAL3; TMED5; ZNF385D; ZNF516; |
| IRF1 | 8.034026465 | 1.511157008 | 7.67212E-05 | ARHGAP5; ARID4B; BTAF1; C5orf64; SP5; STK4; TSC22D2; ANP32B; ATG13; BDNF; CASK; CITED2; CLTC; CTBP2; CTTNBP2NL; CXCR4; DLG4; ELF1; ELMO1; EYA4; FBXW7; FLI1; GLCCI1; GPR137C; MAP4K3; MATR3; MPP5; NR4A2; PAX3; PIGF; PTBP1; RRBP1; SKIDA1; THBS1; TMEM55B; USP33; YRDC; CCDC6; ELOVL6; EVI5; MSX1; PALM2-AKAP2; VAV3; BANP; CD274; FOXQ1; HECTD2; LMO3; TMEM108; C12orf4; HNF4G; N4BP2L1; NDFIP1; PABPC1L; USF1; ZBTB18; BBC3; CNTFR; HIPK1; KAZALD1; MLLT3; RAB4B; RNF44; SMG7; B3GALT2; CTDSP2; DNAJB5; EPHA5; ESRRG; FAM126B; FAM168A; FAM98A; FHL3; IDH1; LURAP1L; MAPK10; NREP; NRXN1; PPARGC1A; RAPGEF5; TFAP2A; TFAP2C; TMEM229B; TMEM33; ITGA6; |
| POU4F3 | 6.994328922 | 1.5530775 | 9.54585E-05 | MAP3K7; MYLK2; PIK3R1; SEMA3A; SORCS1; SP5; ADAM12; BDNF; BLCAP; CEP44; DDX5; ELMO1; EYA4; FN1; FUBP1; GLCCI1; GLS; KALRN; MON2; MTSS1; RARB; TMEM178A; UTRN; CCNT2; DNAJB4; EVI5; MARCKS; PTEN; VAV3; ZFP91; CDH20; EMC7; LMO3; NPAS2; PBX3; RAI2; VIM; ARHGEF10L; CACNA2D1; FRMD3; LTA4H; MLIP; MTUS1; MYT1L; NOVA1; NUFIP2; PTPN12; HIPK1; LUZP1; MLLT3; BAZ1A; CXXC4; DENND1A; E2F6; ESRRG; FAM126B; HERPUD2; HOXD10; HTR2C; LBX1; MAPK10; NRXN1; NUCKS1; OSER1; PPARGC1A; PRKAB1; RNF38; SIPA1L2; SSFA2; SYT1; TCF4; VMA21; ZFPM2; ZNF385D; |
| NOBOX | 6.427221172 | 1.5788467 | 0.000112163 | AFF3; DCBLD2; EIF5A2; NFE2L1; ASPH; BET1; CBL; CCNC; CDK14; CXCR4; FN1; FNBP1L; GPR6; MAB21L1; MTSS1; OSTF1; PFN2; PHF6; PIGF; PLEKHA1; RABGAP1L; RARB; SFPQ; SKIDA1; SRSF1; TACR1; TMEM178A; TNKS2; UTRN; CTNNBIP1; MARCKS; KLF5; LMO3; MSI2; NABP1; NPAS2; PAPD5; VIM; CACNA2D1; MACROD2; MBD5; MTUS1; NONO; NTPCR; APOLD1; CAPN6; NRG1; NYNRIN; B3GALT2; DCAF8; ERG; ESRRG; EZH2; GORASP2; GRM7; HOXC4; LBX1; MARCH8; MBD6; MITF; MTA3; PLCB1; PRDM1; SLMAP; TBC1D12; ZNF385D; BCAS3; ITGA6; |
| PHOX2A | 4.914933837 | 1.696369835 | 0.000123221 | SORCS1; TWF1; BLCAP; CDK14; ELMO1; FBXW7; FNBP1L; GPR6; KALRN; KRAS; MAB21L1; PAX3; RABGAP1L; RARB; SEC23B; SKIDA1; TMEM178A; UTRN; ZNF800; MARCKS; VAV3; EMC7; KLF5; NPAS2; SMIM15; VIM; ARHGEF10L; IPO9; KCNA4; MBD5; NOVA1; SCAI; SCN1A; CAPN6; KCTD15; LUZP1; NRG1; BAZ1A; ESRRG; FAM126B; HCRT; HERPUD2; LBX1; MITF; NRXN1; PRDM1; PRKAB1; RAPGEF5; RNF38; SSFA2; VMA21; KLHL14; |
| FOXD1 | 3.969754253 | 1.813255463 | 0.00012817 | CELF2; FGF9; RFFL; SEMA3A; ST5; CLTC; FUBP1; GLS; KLF4; MAN1C1; MXD1; NCK2; SKIDA1; TMCC1; TPM3; ZNF800; CDH20; GNAI1; KITLG; LMO3; PBX3; PLEKHA5; SMIM15; CACNA2D1; CHTOP; ICOS; NIPAL1; RNF44; AHCYL1; ALDH1A2; B3GALT2; DUSP1; ERG; GRM7; MBNL2; MITF; NR1H4; PRKAA1; PROX1; RREB1; TMEM229B; TWIST1; |
| VAX2 | 4.820415879 | 1.694421174 | 0.000146879 | AFF3; SMAD2; SORCS1; CASK; ELMO1; EYA4; FBXW7; FNBP1L; GPR137C; GPR6; HIAT1; MAB21L1; MIPOL1; PAX3; PDE7A; PLEKHA1; RABGAP1L; SEC23B; TMEM178A; UTRN; ZNF800; MARCKS; PTEN; CDK13; CNKSR2; EMC7; KITLG; LMO3; NPAS2; SMIM15; ARHGEF10L; MBD5; NONO; SCAI; CAPN6; JDP2; LUZP1; LYPLA2; NRG1; THRA; ANO4; BAZ1A; HERPUD2; LBX1; MPC1; NRXN1; PRDM1; PRKAB1; RNF38; SLMAP; TMED5; |
| YY1 | 11.8147448 | 1.368426466 | 0.000154047 | ARID4B; ARMC8; CELF2; FLRT2; H3F3B; NFE2L1; PIM1; PRRC2C; SRSF3; ANP32E; ARCN1; AZIN1; BLCAP; C2CD5; CD164; COL4A3BP; DDX5; DGKH; DHX15; DLG4; FAM155A; FBXW7; FUBP1; GPR6; HIAT1; JARID2; LRRC8A; MAP4K3; MATR3; METTL23; MPZL1; MSANTD2; NBEA; NDRG3; NET1; NR1H3; PFN2; PICALM; POGK; PTBP1; PTMA; RAB5A; RAP1B; SFPQ; SKIDA1; SNAP25; SRSF1; STARD7; TMSB4X; UNC119B; XPO6; CCDC6; CCNT2; DNAJB4; FBXO9; PDIK1L; SRSF7; ZFP91; ARPP19; DESI2; FTL; FUS; GRASP; RAC1; ARGLU1; GNAQ; LGALSL; MTHFD1L; NONO; NOVA1; NUFIP2; RAB14; TBC1D8; UBE2G1; UBE2Q1; USF1; CAD; CCAR2; DYRK1B; EPHA10; HIPK1; HMGA1; HOXB6; IPO11; JDP2; LYPLA2; MLLT3; RNF5; RPRD2; SLC30A3; SMG7; ACVR1; CCNG2; CSNK1A1; CSTF2T; DUSP10; EFNA3; EIF4G2; FAM196B; GRIN2A; HOXC4; KDM5B; MAPKAPK2; MARK3; MTDH; NCOA2; NRXN1; NSF; PPP1R14B; PRADC1; PTGFRN; PTPN5; RAB9A; SLC35A4; SRSF6; SZRD1; TBC1D12; TCF4; UBXN4; UNC79; YBX1; ZC3H6; ATP2B2; PSMB5; ZC3H4; |
| HSF1 | 7.844990548 | 1.484758316 | 0.000168234 | AFF3; ARHGAP5; ARID4B; COPS7B; CTDSPL2; DYNLL1; FOXO1; MAP3K7; ABHD2; AKAP12; ANKRD34B; ANXA4; ARID2; ATP6V1A; BDNF; CLTC; DGKH; EHMT2; FBXL14; FOSB; GPD2; HSPD1; HSPE1; JARID2; KCNA5; MTSS1; MXD1; NME1-NME2; NME2; NXT2; OTX2; RRBP1; SEC63; SERP1; STX12; TGIF2; THBS1; TMEM68; TPM4; WDR61; CTNNBIP1; KLF5; RCN2; SMIM15; FKBP1A; HEXIM1; HNF4G; SCAI; CAD; LZIC; MACF1; NRG1; PPM1N; RTN2; STOML1; ALDH1A2; BAZ2A; BCAP31; BEND3; BNIP2; DIRAS2; DNAJB5; DUSP1; E2F6; EBAG9; FAM168A; FKBP4; FMNL2; HCRT; L3MBTL4; MBNL2; MBTPS2; PRKAB1; PROX1; RAPGEF5; RPGRIP1L; SAMD8; SIPA1L2; SSFA2; TAF7L; TNC; YTHDC1; ATP2B2; |
| BARX2 | 3.59168242 | 1.851216741 | 0.00017259 | SEMA3A; CTTNBP2NL; ELF1; NBEA; RARB; RSBN1; SFPQ; SKIDA1; PTEN; ZFP91; KITLG; NPAS2; VIM; ARHGEF10L; FRMD3; KCNA4; MYT1L; PTPN12; ZBTB18; LUZP1; BAZ1A; BEND6; ESRRG; FMNL2; GRIP1; HERPUD2; LBX1; MITF; NRXN1; PPTC7; PRDM1; SCRT2; SLC17A6; SSFA2; ST3GAL3; TNC; ZFPM2; ATP2B2; |
| HNF4A | 24.1020794 | 1.219366437 | 0.00018952 | ABHD17B; ARID4B; BTBD3; C5orf64; COL6A6; FGF9; PIK3R1; PIM1; SEMA3A; SMARCD2; SMOC1; SORCS1; SP5; ST6GALNAC6; STK4; TANC1; TXLNG; ADAM12; ADPGK; BACH2; BET1; BLCAP; BSCL2; CAPZA1; CCNC; CD164; CDK4; CDON; CEP44; CLTC; CNN3; CORO1C; CTBP2; EHMT2; ELF1; EYA4; FAM107B; FAM155A; FGF17; FN1; FUBP1; GCH1; GPD2; HELZ2; HNRNPK; HOXB4; HSP90B1; HSPD1; HSPE1; IGF1; KALRN; KANK4; KCNIP3; KTN1; LASP1; MAN1C1; MAP4K3; MMD; MOB3C; MON2; MTSS1; MYEF2; NAB1; NET1; NR1H3; PAX3; PHF6; PRKRIR; RABGAP1L; RAP1B; RARB; RRBP1; RSPO3; SFPQ; SH3BGRL; SKIDA1; SLC10A7; SLC25A22; SLC29A3; SMAP1; SYN3; TAGLN2; THBS1; TMEM135; TMEM178A; TNKS2; TPM3; TSPAN4; TTR; USP33; UTRN; YRDC; ARPC2; CCDC6; CD2AP; FRMD5; SEMA4B; SLC22A3; TOMM70A; ZFP91; ARPP19; CNKSR2; DLX1; ETFA; FOXQ1; FUS; GNAI1; IRF6; KITLG; KLF5; LMO3; NPAS2; PAPD5; PBX3; PCSK7; SCOC; STARD4; ARGLU1; CELSR2; COL1A1; COPS4; DGKB; DMRT2; FAF1; FRMD3; GNAQ; HNF4G; ICOS; MACROD2; MAFB; MBD5; MTHFD1L; MTUS1; MYT1L; NONO; NOVA1; PELI3; PLA2G3; PPM1B; PTPN12; SLCO3A1; TNFSF11; UBE2Q1; AAK1; ACTR1A; ATG2B; CNTFR; DYNLL2; DYRK1B; GAB2; INPP5K; JDP2; LARGE; LDLRAP1; LUZP1; LYPLA2; MACF1; PCDH1; PHC2; PLLP; PPFIA3; RAB37; RNF44; TGFB1; ACSL6; ACVR1; ADAM23; AHCYL1; AIDA; AJUBA; AKT2; ARHGAP42; B4GALT5; BAZ2A; BCAP31; BNIP2; BNIP3L; C2orf57; CHGA; CSNK1A1; CXXC4; DCAF8; DENND1A; DHX40; DIRAS2; DMRT3; DTX2; DUSP10; EBAG9; ELOVL1; EPHA7; ERG; ESRRA; ESRRG; ETFB; FHL3; FITM2; FMNL2; GABRA1; GORASP2; GRIP1; GRM7; HNRNPDL; HOXD10; IDH1; IFT20; MBD6; MBNL2; MBOAT2; MIA3; MITF; MRGBP; MYO1C; NCOA2; NRXN1; PDS5A; PEX3; PLCB1; PPARGC1A; PPP1R14B; PRADC1; PRDM1; PROX1; PTGFRN; PTPN5; PXN; RAPGEF5; RASIP1; RCOR2; RREB1; SASH3; SLC17A6; SLC25A5; SLC5A7; SLMAP; SRC; SS18; SSFA2; ST3GAL3; SYT1; TPCN1; UBXN4; UNC79; VARS; VAX1; WNT2B; WNT7A; YTHDC1; ZMAT4; ZNF385D; ATP2B2; BCAS3; ETNK2; MEGF8; SETD8; |
| POU5F1 | 4.253308129 | 1.739619713 | 0.00019616 | CDH7; CDK14; CDON; CITED2; FN1; GJA1; GLS; IGF1; JARID2; OSBPL7; DLX1; GNAI1; KITLG; KLF5; SPOPL; STAT4; ARGLU1; CACNA2D1; DGKB; HNF4G; MTUS1; NUFIP2; RAB14; SEC61A2; ZBTB18; LARGE; MACF1; MLLT3; THRA; ALDH1A2; C11orf87; CTDSP2; DENND1A; E2F7; ESRRG; GREM2; HMGN3; HOXD10; NRXN1; PDS5A; PRDM1; SSFA2; TFAP2A; ZNF516; ITGA6; |
| FOXJ2 | 3.59168242 | 1.832643281 | 0.000212401 | HAT1; MAP3K7; ATG13; FOSB; GPR6; HNRNPK; KALRN; SKIDA1; SLC10A7; SLC44A1; UST; MARCKS; PTEN; FOXQ1; GNAI1; CACNA2D1; DGKB; MACROD2; MBD5; MTUS1; NOVA1; ZBTB18; ANO4; DENND1A; ESRRG; FAM196B; FSTL1; GREM2; LRRC10B; MITF; NREP; PPARGC1A; PRDM1; SSBP3; SSFA2; TWIST1; WNT2B; ATP2B2; |
| PAX6 | 5.765595463 | 1.579078845 | 0.000249261 | ARHGAP44; PIK3R1; SEMA3A; SMOC1; SORCS1; ABCB7; CASK; CDK14; ELMO1; EYA4; FAM63B; FBXW7; GPR6; KALRN; NBEA; RARB; SKIDA1; TMEM178A; UTRN; VAMP4; DLG5; PTEN; EMC7; LMO3; MSI2; NPAS2; VIM; ARGLU1; CACNA2D1; KCNA4; MACROD2; MBD5; NOVA1; AAK1; EPHA10; HMGA1; LUZP1; MACF1; NRG1; BAZ1A; CXXC4; DENND1A; ERG; ESRRG; FAM126B; GRIP1; HERPUD2; HTR2C; LBX1; LINGO2; MBNL2; MITF; NRXN1; PRKAB1; RAPGEF5; SSFA2; TFAP2A; TNC; UBXN4; YTHDC1; ATP2B2; |
| ASCL2 | 13.04347826 | 1.327308294 | 0.0002694 | DCBLD2; PIK3R1; RAPGEFL1; ST5; STK4; TANC1; ACER2; ARFIP1; BACH2; BDNF; CASK; CERS2; CITED2; CORO1C; CPEB1; DGKZ; EYA4; FBXW7; FN1; GLCCI1; HELZ2; HNRNPU; HS3ST3B1; KALRN; KCNJ2; LASP1; MON2; NBEA; NETO1; OSBPL7; RRBP1; SEC63; SH3BGRL; SMARCB1; SNAI2; TACR1; TMEM178A; TMSB4X; TNPO2; TPM3; CD2AP; FOXN2; KLF10; XRCC5; DBN1; DDX42; DLX1; GRASP; MED21; MYL12A; PBX3; PCSK7; RAI2; YWHAH; ARHGEF10L; CACNA2D1; CHTOP; DES; DGKB; MACROD2; MBD5; MTHFD1L; N4BP2L1; NOVA1; TBC1D8; UBE2Q1; USF1; ZBTB18; ADAMTS10; DYRK1B; HM13; KCNA6; LARGE; MACF1; ONECUT2; PHC2; POLL; PPP2R5B; RPRD2; SLC30A3; THRA; ACSL6; ACVR1; AIDA; AJUBA; AKT2; ANKRD13A; ANXA7; ATP1B1; B3GALT2; BAZ2A; CA7; CHGA; DCAF8; DENND1A; DEXI; DUSP1; DUSP10; EHBP1L1; EIF4G2; EPHA5; FHL3; FITM2; FMNL2; FURIN; GREM2; HCRT; HLF; HMGN3; HNRNPDL; HOXD10; ICMT; ING2; KDM4A; LRRC10B; LURAP1L; MLC1; NEUROD1; NRXN1; PPP1R14B; PRADC1; PXN; RAB9A; RNF165; SAMD8; SHROOM2; SLC12A2; TBX3; TFAP2C; TNC; ZFPM2; ZNF385D; ZNF710; BCAS3; CYB561D1; ITGA6; MTSS1L; SGSM2; |
| DLX3 | 4.536862004 | 1.675856859 | 0.000294483 | AFF3; FGF9; MYLK2; BDNF; CDK14; CERS2; ELMO1; FAM155A; FUBP1; HNRNPK; HOXB4; OTX2; RARB; RRBP1; SH3BGRL; ZNF800; CCNT2; ZFP91; DLX1; LMO3; NPAS2; TMEM106B; CHL1; MBD5; SEPT8; USF1; WAPAL; YBX3; APOLD1; ATP8B2; HMGA1; PCDH1; ESRRG; FAM126B; GABRA1; GPR158; HOXD10; PAQR3; PPTC7; PRADC1; RNF38; SLC46A3; SLMAP; SSBP3; SSFA2; STAC; STK3; UBXN4; |
| HOXC9 | 5.860113422 | 1.560146254 | 0.000308099 | MAP3K7; SEMA3A; SORCS1; ACER2; AKAP12; ANXA4; BDNF; CBL; CCNC; CEP44; CNN3; GLS; HNRNPK; LRCH1; NBEA; NDRG3; OTX2; PIGF; RARB; SEC23B; SFPQ; SLC25A30; TMEM178A; TTR; CTNNBIP1; DNAJB4; FRMD5; OPRK1; PDIK1L; XRCC5; ZFP91; DLX1; GNAI1; KLF5; LMO3; PBX3; TMEM106B; VIM; ARHGEF10L; CHL1; FAM120A; MTHFD1L; MYT1L; NIPAL1; NONO; AMMECR1L; HOXB6; IPO11; MACF1; ACVR1; ADAM23; GABRA1; GREM2; KDM4A; LBX1; LONRF3; MARCH7; NR1H4; NRXN1; SIPA1L2; ST3GAL3; BCAS3; |
| LMO2 | 6.143667297 | 1.536416611 | 0.000339509 | ARMC8; CSPG5; FGF9; FOXO1; HAT1; SEMA3A; ACTB; C9orf152; CPEB1; ELMO1; HIAT1; HNRNPU; HSP90B1; KALRN; LRCH1; MTX1; NAB1; NBEA; NR4A2; OTX2; POGK; RAB43; TAGLN2; TMEM55B; YWHAZ; FRMD5; OPRK1; PALM2-AKAP2; DBN1; PBX3; TSC22D4; ARGLU1; FAM169A; HNF4G; NOL4; NOVA1; SEPT8; UBE2Q1; CNTFR; DYNLL2; KAZALD1; LDLRAP1; MACF1; NUMBL; PLLP; POLL; ZC3H7B; ANXA7; ATP1B1; CA7; DCAF8; GORASP2; GRIN2A; HAPLN2; HNRNPDL; ING2; PPTC7; RCOR2; SLC6A9; ST3GAL3; TBX3; TDRD7; ZBTB21; ZCCHC2; ZFPM2; |
| PAX4 | 2.36294896 | 2.060234281 | 0.000443988 | C5orf64; RFFL; SEMA3A; TANC1; BACH2; FLI1; KALRN; MSX1; NEK6; CDK13; ARGLU1; CACNA2D1; MACROD2; MYT1L; SCN1A; YBX3; HMGA1; KAZALD1; ESRRG; HOXD10; HTR2C; RNF38; SSBP3; TCF4; KLHL14; |
| ETS1 | 20.13232514 | 1.230323881 | 0.000462206 | ABHD17B; ARMC8; CELF2; CSPG5; MAP3K7; PIK3R1; RFFL; STK4; TANC1; ACER2; ANP32B; ARCN1; ARFIP1; ARG2; ARID2; ARPC3; BAG4; BET1; BLCAP; BSCL2; C9orf152; CALM2; CDK14; CITED2; CNN3; COL4A3BP; CORO1C; CTTNBP2NL; DDX5; DLG4; E2F5; EIF1AD; ELMO1; FLI1; G6PD; GLCCI1; HNRNPK; HSP90B1; IGF1; KALRN; KCNIP3; LIN7C; LRRC8A; MAN1C1; METTL23; MOB3C; MSANTD2; MXD1; NCK2; NDFIP2; NME1-NME2; NME2; NR1H3; NR4A2; OSTF1; PEA15; RAB43; RABGAP1L; RAP1B; SEC63; SERP1; SH3GL1; SNAP25; SPRED1; TACR1; TAGLN2; THBS1; TIGD6; TMEM178A; TSPAN4; UST; UTRN; VASP; VPS45; ZNF800; ARPC2; CPNE8; FOXN2; GATAD1; MSX1; NEK6; NRG3; PTEN; RAP2C; TMEM39A; TOMM70A; VAV3; ARPP19; CCR7; CDH20; CDK13; CNKSR2; GRASP; KCNS3; KITLG; KLF13; LMO3; NABP1; NPAS2; RAC1; RAI2; SCOC; SDHD; SMIM15; SPOPL; STAT4; TIMM8B; TSC22D4; TUSC3; VPS37B; ARHGEF10L; CACNA2D1; DES; FKBP1A; GNG12; KIAA1429; LEMD3; MBD5; MTHFD1L; MTUS1; NOVA1; PLA2G3; PPP1CB; TBC1D8; TFG; TNFSF11; UBLCP1; USF1; WAPAL; AAK1; APOLD1; ATP8B2; CCAR2; EPN1; FKBP8; GMPR; HMGA1; IPO11; LDLRAP1; LYPLA2; MACF1; PCDH1; STOML1; WASF2; ABHD6; ACVR1; AIDA; AKT2; APPL2; ATP1B1; BAZ2A; BCAP31; BNIP2; CDC37L1; CSNK1A1; DENND1A; DNAJB5; DR1; DUSP4; EHBP1L1; ELOVL1; ESRRG; FAM196B; FHL3; FURIN; GABRA1; GRIN2A; HLF; HOXD10; HTR2C; IFT20; ING2; KDM1A; LURAP1L; MAFK; MAPKAPK2; MBNL2; METTL9; MITF; MSI1; MYO1C; NAB2; NIPBL; NRXN3; PDE1B; PDS5A; PLCB1; PPARGC1A; PRDM1; PRKAB1; RCOR2; RNF165; RPL37; RWDD4; SH3TC1; SLC25A5; SNX25; SRC; SRPR; SS18; SSBP3; STK38; SZRD1; TDRD7; TSNAX; TWIST1; ZBTB7A; ZFYVE16; CYB5D1; ETNK2; LAS1L; SETD8; SGSM2; |
| HOXC6 | 2.930056711 | 1.88625706 | 0.000478997 | DCBLD2; PRRC2C; SEMA3A; SORCS1; CDK4; EYA4; KALRN; OTX2; PDE7A; RARB; SP2; UTRN; MARCKS; LMO3; DES; MBD5; MYT1L; LDLRAP1; MACF1; ONECUT2; BAZ1A; CXXC4; ESRRG; FAM126B; GRM7; MBNL2; NRXN1; PPARGC1A; PRDM1; PRKAB1; SCRT2; |
| GATA1 | 6.994328922 | 1.465611425 | 0.000550755 | CELF2; SP5; CASK; CORO1C; EDN1; ELF1; EYA4; FBXW7; FLI1; GLCCI1; GPD2; KALRN; KTN1; MAP4K3; NR4A2; PFN2; PIGF; RABGAP1L; RARB; SKIDA1; SNAP25; SOX9; SRSF1; TMEM178A; TPK1; TPM3; TTR; UTRN; VAMP4; FOXQ1; FUS; IRF6; KITLG; LMO3; MSI2; PBX3; ARGLU1; DCAF12; HNF4G; MBD5; MYT1L; WNK4; AAK1; MACF1; ONECUT2; PHC2; ACSL6; ACVR1; C11orf87; CTNNA3; CXXC4; DENND1A; DUS2; ERG; ESRRG; FMNL2; FURIN; GABRA1; GRIP1; HIC1; MITF; MTA3; MYBPC3; MYO1C; PDE1B; PPARGC1A; SLMAP; SYT1; TNC; WNT7A; ZFPM2; ZNF385D; ETNK2; KLHL14; |
| DBX1 | 2.930056711 | 1.870473143 | 0.000553642 | ARHGAP44; C5orf64; SEMA3A; BDNF; EYA4; GLS; GPD2; HNRNPK; IGF1; TMEM178A; MARCKS; PBX3; ARGLU1; CHL1; KCNA4; MBD5; NOVA1; KAZALD1; ACSL6; BAZ1A; CXXC4; DENND1A; ERG; HOXD10; MBNL2; NRXN3; PPARGC1A; PRDM1; SLC17A6; SYT1; ATP2B2; |
| TGIF1 | 9.357277883 | 1.380448166 | 0.000571836 | ARMC8; HCN1; MYLK2; PIM1; ADAM12; ARFIP1; ARID2; AZIN1; BLCAP; CDK14; COL4A3BP; EIF1AX; ELF1; ELMO1; EYA4; FBXW7; FN1; FNBP1L; GLS; HSPD1; KCNJ2; LRCH1; MAP4K3; MSANTD2; PDE7A; RABGAP1L; RARB; RNF145; RSBN1; RYBP; SKIDA1; SMAP1; SPRED1; TGIF2; TSPAN4; YWHAZ; ZNF597; ARPC2; DNAJB4; FOXN2; FRMD5; TAB3; TOMM70A; XRCC5; GRASP; HECTD2; NPAS2; PBX3; PLK3; CHL1; COPS4; DES; ESYT2; FRMD3; GNAQ; HUNK; MACROD2; MLIP; TBC1D8; CAPN6; EPHA10; HM13; HOXB6; LARGE; LDLRAP1; MACF1; NGFR; ONECUT2; PHC2; PPM1N; RTN2; THRA; AHR; B3GALT2; BCL2L13; BNIP3L; CNEP1R1; DENND1A; DIRAS2; EFNA3; ESRRG; GPCPD1; GRIP1; GRM7; HNRNPDL; KRT1; LRRC10B; MBNL2; MITF; MOB4; NIPBL; PPTC7; RAPGEF5; RCAN2; SAR1B; SSBP3; ST3GAL3; VASH2; C17orf96; |
| PBX1 | 7.088846881 | 1.45935476 | 0.000572903 | FGF9; SEMA3A; ADAM12; BACH2; C1GALT1; EYA4; FAM63B; FBXW7; GJA1; HNRNPK; KLF4; MAB21L1; MEOX2; MTSS1; NDFIP2; NETO1; NR4A2; PAX3; PDE7A; POGK; RABGAP1L; SERP1; SP2; SYN3; TMEM178A; UTRN; C9orf72; CCNG1; CD2AP; FRMD5; NEK6; XRCC5; CD274; CDH20; CNKSR2; EFS; GNAI1; KITLG; LMO3; NABP1; TMEM106B; CACNA2D1; MBD5; NNT; PPM1B; PTPN12; SEC61A2; SEPT8; ZBTB18; AAK1; GAB2; HIPK1; HMGA1; MACF1; ANO4; CHORDC1; CHST9; CTNNA3; DENND1A; DUSP10; FAM196B; GRIP1; GRM7; HOXD10; MBNL2; MTA3; NRXN1; PDE1B; RANBP1; SCRT2; SIPA1L2; SLC6A9; SRSF6; ZFPM2; MAPK4; |
| ELF3 | 4.631379962 | 1.616810459 | 0.000583821 | CTDSPL2; DYNLL1; SMAD2; ST5; TWF1; ARFIP1; ARID2; CBL; CDC42SE1; ELF1; ELMO1; FAM155A; G6PD; MXD1; NBEA; NR4A2; PICALM; RABGAP1L; SH3BGRL; SKIDA1; SRSF9; TAGLN2; UTRN; C9orf72; MAP3K3; TOMM70A; IRF6; NABP1; CACNA2D1; DCAF12; MBD5; N4BP2L1; NOL4; NUFIP2; PPM1B; KCTD15; PHC2; ACVR1; AHCYL1; ESRRA; PRDM1; RAB11B; RNASE6; SPTY2D1; SSBP3; TMEM33; VAX1; ZNF385D; ITGA6; |
| FOXK1 | 1.79584121 | 2.264783219 | 0.000649812 | CELF2; HOXB4; NR4A2; ZNF800; DNAJB4; PLEKHA5; HEXIM1; INPP5K; MACF1; CXXC4; ERG; ESRRG; MBNL2; NRXN3; PDLIM3; PRDM1; TCF4; TWIST1; BCAS3; |
| CACD | 5.198487713 | 1.542893179 | 0.000860775 | ATXN7L3; EIF5A2; PRRC2C; ST5; TANC1; ACTB; CALM2; CITED2; ELF1; FLI1; HOXB4; HS3ST3B1; LRRC59; MBLAC2; MIER1; MTSS1; MTX1; POLR3G; PRKRIR; SH3BGRL3; TAGLN2; YWHAZ; ZNF148; MAP3K3; RAP2C; ARMCX2; FUS; IRF6; MYL12A; RAC1; TSC22D4; VIM; ATP10D; DES; KCNA4; SEPT8; APOLD1; HIPK1; IPO11; MLLT3; WASF2; BCL2L13; CTDSP2; EDIL3; HOXD10; MITF; NAB2; NRXN1; NUCKS1; PLA2G15; PPP1R14B; PXN; RASIP1; RNF165; SYT1; |
| NANOG | 2.551984877 | 1.908705438 | 0.000900149 | DYNLL1; SMARCD2; BACH2; FAM91A1; FNBP1L; GLS; MEOX2; MYEF2; NFATC2; ARPC2; PTEN; DLX1; RAC1; CACNA2D1; HIPK1; HMGA1; PPAN; RAB37; BEND3; CSNK1A1; EIF2S3; HERPUD2; MTDH; SGPL1; SS18; WBP1L; BCAS3; |
| HOXA9 | 6.899810964 | 1.443825292 | 0.000910625 | ARID4B; CDH7; DCBLD2; FGF9; HAT1; MAP3K7; PIK3R1; SEMA3A; SRSF3; ARID2; C2orf69; CCNC; CDK14; CEP44; CLTC; DGKE; DHX15; EDN1; FN1; GLCCI1; HACE1; HOXB4; IGF1; LRCH1; MPP5; MYEF2; NDRG3; SEC23B; SH3GL1; SKIDA1; TMEM178A; UST; DLX1; GNAI1; KLF5; LMO3; PBX3; PCSK7; DES; DGKB; FOPNL; MACROD2; MTUS1; NIPAL1; NONO; SCN1A; SLC35F5; HIPK1; NRG1; ACSL6; ADAM23; AHCYL1; AHR; ANO4; DCAF8; DNAJB5; ERG; FAM98A; GREM2; HLF; HOXC4; KDM4A; MAPK10; MBNL2; MIA3; PPTC7; SSFA2; SYT1; UBXN4; YBX1; ZCCHC2; ZFPM2; ZNF385D; |
| ETV7 | 9.357277883 | 1.360708867 | 0.000925802 | ABHD17A; BTAF1; COPS7B; HAT1; MAP3K7; MARK1; PIK3R1; SMAD2; ARFIP1; BAG4; CAAP1; CEP44; CNN3; CORO1C; CTTNBP2NL; DLG4; E2F5; ELMO1; G6PD; HNRNPU; HSP90B1; IGF1; LRRC8A; METTL23; MSANTD2; NDFIP2; NME1-NME2; NME2; NR1H3; NR4A2; NXT2; PIGF; RAB43; RABGAP1L; SERP1; SNAI2; SNAP25; TAGLN2; TGIF2; TMEM68; UTRN; WDR1; XPO6; ZNF800; CPNE8; ELOVL6; FOXN2; KLF10; RAP2C; TOMM70A; CDK13; LMO3; NABP1; PCSK7; STAT4; TUSC3; ARGLU1; DCAF12; DES; LEMD3; LSM5; PTPN12; SEPT8; UBLCP1; EPN1; HM13; HMGA1; IPO11; KAZALD1; LDLRAP1; LYPLA2; MACF1; POLL; PPAN; ACTN2; APPL2; BNIP2; CDC37L1; CSNK1A1; CUL4A; CYB5R4; ESRRA; ESRRG; FURIN; HIC1; ING2; LURAP1L; MAPKAPK2; MARK3; NIPBL; RNASE6; RPS13; SASH3; SLC25A5; SPTY2D1; SSFA2; TDRD7; UBXN4; LAS1L; |
| HOXB3 | 3.686200378 | 1.678742023 | 0.001016275 | CTDSPL2; SEMA3A; CDK4; CLTC; ELF1; EYA4; FBXW7; KALRN; KCNJ2; MAP4K3; NAB1; RABGAP1L; RARB; SEC23B; SKIDA1; TMEM178A; KCNG3; PTEN; LMO3; NPAS2; PAPD5; SMIM15; VIM; MBD5; MTUS1; MYT1L; NONO; NOVA1; CAPN6; MACF1; B3GALT2; HERPUD2; MAPK10; MITF; NCOA2; PRDM1; SIPA1L2; ST3GAL3; TMEM218; |
| GBX2 | 3.402646503 | 1.718967163 | 0.001022769 | BLCAP; CASK; FBXW7; GCH1; KALRN; MAP4K3; MIER1; NR4A2; OSTF1; RABGAP1L; SKIDA1; TMEM178A; UTRN; MARCKS; NPAS2; PAPD5; PBX3; IPO9; MBD5; MTUS1; PTPN12; LUZP1; MACF1; AACS; CNEP1R1; CTNNA3; E2F6; FMNL2; GRAMD3; HCRT; MITF; PRDM1; SIPA1L2; SSFA2; ST3GAL3; KLHL14; |
| NKX2-1 | 6.710775047 | 1.439824589 | 0.001148062 | ARID4B; ARMC8; BTBD3; NFE2L1; SEMA3A; TWF1; BACH2; BET1; CDC42; CDK14; CORO1C; ELMO1; EYA4; GLS; KALRN; LRCH1; MAB21L1; MSANTD2; NBEA; NFATC2; RABGAP1L; RAP1B; RARB; RRAGC; SLC10A7; SNAP25; STC2; STX12; UTRN; YRDC; DNAJB4; MAP3K3; ZFP91; CDK13; DLX1; EMC7; HECTD2; LMO3; NABP1; PCSK7; STAT4; CACNA2D1; DGKB; FRS2; HEXIM1; HNF4G; NTPCR; PTPN12; SLC35F5; HIPK1; MLLT3; POLL; AKT2; AP3S1; BLOC1S5; DNAJB5; EIF2S3; ESRRG; GRM7; HOXD10; LUZP2; MBNL2; MITF; NR1H4; NRXN1; NSF; PEX3; PPARGC1A; SASH3; SYT1; ZBTB7A; |
| RUNX2 | 3.497164461 | 1.693793753 | 0.001149665 | ARID4B; PIM1; SEMA3A; CBL; CLTC; CNN3; GLS; HOXB4; MTX1; RABGAP1L; RYBP; SFPQ; NEK6; CCR7; DLX1; FUS; LMO3; TSC22D4; VIM; MACROD2; MTHFD1L; ZBTB18; AAK1; HOXB6; KCNA6; MLLT3; AKT2; CXXC4; DCAF8; ERG; ESRRG; FMNL2; HOXC4; NR1H4; PRDM1; SASH3; WNT2B; |
| BARHL1 | 5.103969754 | 1.529725989 | 0.001162264 | ARHGAP44; NFE2L1; RFFL; BET1; CDK14; EYA4; FNBP1L; KALRN; PHF6; PIGF; RABGAP1L; RARB; SFPQ; TMEM178A; UTRN; YRDC; CTNNBIP1; EVI5; MARCKS; PTEN; DLX1; KITLG; KLF5; LMO3; NPAS2; VIM; ARGLU1; FRMD3; MBD5; MTUS1; MYT1L; NTPCR; ATP8B2; HIPK1; NRG1; PHC2; ADAM23; ERG; ESRRG; EZH2; FMNL2; GRIP1; GRM7; LUZP2; MARCH8; MBD6; MITF; MTA3; NRXN1; PLCB1; RAPGEF5; RNF38; SYT1; TCF4; |
| JUNB | 14.46124764 | 1.257631865 | 0.001365208 | C5orf64; FGF9; FLRT2; GOLGA3; H3F3B; MARK1; PIM1; RFFL; SEMA3A; ABHD2; ACTB; ANP32E; ANXA2; BACH2; BLCAP; BSCL2; C1GALT1; C5orf51; CASK; CDK14; CHSY1; CLTC; CORO1C; CPEB1; CTTNBP2NL; CXCL11; EDN1; ELF1; ELMO1; FLI1; FNBP1L; GJA1; GLS; KALRN; KTN1; LASP1; MXD1; NAB1; NET1; NR4A2; OSBPL7; PCDH17; PDGFA; PEA15; RIT2; RYBP; SDCBP; SH3BGRL3; SKIDA1; SNAI2; SNAP25; SPRED1; TMCC1; TMSB4X; UTRN; VASP; ZC2HC1C; ZNF800; ARPC2; CD2AP; CPNE8; DLG5; EVI5; FRMD5; NEK6; PALM2-AKAP2; PTEN; ARL8B; CCR7; FTL; GNAI1; GRASP; HECTD2; IRF6; KITLG; NABP1; NPAS2; RAC1; SCOC; STAT4; TPD52L2; VIM; DGKB; RTCB; SEPT8; SLCO3A1; USF1; YBX3; AAK1; CAPN6; DIRAS1; GAB2; HM13; INPP5K; MACF1; RAB37; SLC4A5; ACSL6; ACVR1; AHCYL1; AJUBA; ANXA7; BCAP31; BEND6; CA7; CAPN2; CHST1; CTNNA3; DENND1A; DMRT3; DNAJB5; DR1; DTX2; DUSP10; EHBP1L1; ENHO; ERG; ESRRA; FHL3; GPR158; GRM7; HIC1; KCNAB3; KDM4A; KIAA1671; MARK3; MPP1; MSI1; NECAB3; NRXN1; PDE1B; PPARGC1A; PPP1R14B; PRKAA1; RASIP1; RCAN2; SCRT2; SGPL1; SHROOM2; SLAMF9; SNX25; SPTY2D1; SRC; ST18; TNC; VARS; VASH2; WNT2B; ZFPM2; ZNF385D; ZNF516; ETNK2; SGSM2; |
| JUND | 14.46124764 | 1.257631865 | 0.001365208 | C5orf64; FGF9; FLRT2; GOLGA3; H3F3B; MARK1; PIM1; RFFL; SEMA3A; ABHD2; ACTB; ANP32E; ANXA2; BACH2; BLCAP; BSCL2; C1GALT1; C5orf51; CASK; CDK14; CHSY1; CLTC; CORO1C; CPEB1; CTTNBP2NL; CXCL11; EDN1; ELF1; ELMO1; FLI1; FNBP1L; GJA1; GLS; KALRN; KTN1; LASP1; MXD1; NAB1; NET1; NR4A2; OSBPL7; PCDH17; PDGFA; PEA15; RIT2; RYBP; SDCBP; SH3BGRL3; SKIDA1; SNAI2; SNAP25; SPRED1; TMCC1; TMSB4X; UTRN; VASP; ZC2HC1C; ZNF800; ARPC2; CD2AP; CPNE8; DLG5; EVI5; FRMD5; NEK6; PALM2-AKAP2; PTEN; ARL8B; CCR7; FTL; GNAI1; GRASP; HECTD2; IRF6; KITLG; NABP1; NPAS2; RAC1; SCOC; STAT4; TPD52L2; VIM; DGKB; RTCB; SEPT8; SLCO3A1; USF1; YBX3; AAK1; CAPN6; DIRAS1; GAB2; HM13; INPP5K; MACF1; RAB37; SLC4A5; ACSL6; ACVR1; AHCYL1; AJUBA; ANXA7; BCAP31; BEND6; CA7; CAPN2; CHST1; CTNNA3; DENND1A; DMRT3; DNAJB5; DR1; DTX2; DUSP10; EHBP1L1; ENHO; ERG; ESRRA; FHL3; GPR158; GRM7; HIC1; KCNAB3; KDM4A; KIAA1671; MARK3; MPP1; MSI1; NECAB3; NRXN1; PDE1B; PPARGC1A; PPP1R14B; PRKAA1; RASIP1; RCAN2; SCRT2; SGPL1; SHROOM2; SLAMF9; SNX25; SPTY2D1; SRC; ST18; TNC; VARS; VASH2; WNT2B; ZFPM2; ZNF385D; ZNF516; ETNK2; SGSM2; |
| FOSB | 14.46124764 | 1.257631865 | 0.001365208 | C5orf64; FGF9; FLRT2; GOLGA3; H3F3B; MARK1; PIM1; RFFL; SEMA3A; ABHD2; ACTB; ANP32E; ANXA2; BACH2; BLCAP; BSCL2; C1GALT1; C5orf51; CASK; CDK14; CHSY1; CLTC; CORO1C; CPEB1; CTTNBP2NL; CXCL11; EDN1; ELF1; ELMO1; FLI1; FNBP1L; GJA1; GLS; KALRN; KTN1; LASP1; MXD1; NAB1; NET1; NR4A2; OSBPL7; PCDH17; PDGFA; PEA15; RIT2; RYBP; SDCBP; SH3BGRL3; SKIDA1; SNAI2; SNAP25; SPRED1; TMCC1; TMSB4X; UTRN; VASP; ZC2HC1C; ZNF800; ARPC2; CD2AP; CPNE8; DLG5; EVI5; FRMD5; NEK6; PALM2-AKAP2; PTEN; ARL8B; CCR7; FTL; GNAI1; GRASP; HECTD2; IRF6; KITLG; NABP1; NPAS2; RAC1; SCOC; STAT4; TPD52L2; VIM; DGKB; RTCB; SEPT8; SLCO3A1; USF1; YBX3; AAK1; CAPN6; DIRAS1; GAB2; HM13; INPP5K; MACF1; RAB37; SLC4A5; ACSL6; ACVR1; AHCYL1; AJUBA; ANXA7; BCAP31; BEND6; CA7; CAPN2; CHST1; CTNNA3; DENND1A; DMRT3; DNAJB5; DR1; DTX2; DUSP10; EHBP1L1; ENHO; ERG; ESRRA; FHL3; GPR158; GRM7; HIC1; KCNAB3; KDM4A; KIAA1671; MARK3; MPP1; MSI1; NECAB3; NRXN1; PDE1B; PPARGC1A; PPP1R14B; PRKAA1; RASIP1; RCAN2; SCRT2; SGPL1; SHROOM2; SLAMF9; SNX25; SPTY2D1; SRC; ST18; TNC; VARS; VASH2; WNT2B; ZFPM2; ZNF385D; ZNF516; ETNK2; SGSM2; |
| FOS | 14.46124764 | 1.257631865 | 0.001365208 | C5orf64; FGF9; FLRT2; GOLGA3; H3F3B; MARK1; PIM1; RFFL; SEMA3A; ABHD2; ACTB; ANP32E; ANXA2; BACH2; BLCAP; BSCL2; C1GALT1; C5orf51; CASK; CDK14; CHSY1; CLTC; CORO1C; CPEB1; CTTNBP2NL; CXCL11; EDN1; ELF1; ELMO1; FLI1; FNBP1L; GJA1; GLS; KALRN; KTN1; LASP1; MXD1; NAB1; NET1; NR4A2; OSBPL7; PCDH17; PDGFA; PEA15; RIT2; RYBP; SDCBP; SH3BGRL3; SKIDA1; SNAI2; SNAP25; SPRED1; TMCC1; TMSB4X; UTRN; VASP; ZC2HC1C; ZNF800; ARPC2; CD2AP; CPNE8; DLG5; EVI5; FRMD5; NEK6; PALM2-AKAP2; PTEN; ARL8B; CCR7; FTL; GNAI1; GRASP; HECTD2; IRF6; KITLG; NABP1; NPAS2; RAC1; SCOC; STAT4; TPD52L2; VIM; DGKB; RTCB; SEPT8; SLCO3A1; USF1; YBX3; AAK1; CAPN6; DIRAS1; GAB2; HM13; INPP5K; MACF1; RAB37; SLC4A5; ACSL6; ACVR1; AHCYL1; AJUBA; ANXA7; BCAP31; BEND6; CA7; CAPN2; CHST1; CTNNA3; DENND1A; DMRT3; DNAJB5; DR1; DTX2; DUSP10; EHBP1L1; ENHO; ERG; ESRRA; FHL3; GPR158; GRM7; HIC1; KCNAB3; KDM4A; KIAA1671; MARK3; MPP1; MSI1; NECAB3; NRXN1; PDE1B; PPARGC1A; PPP1R14B; PRKAA1; RASIP1; RCAN2; SCRT2; SGPL1; SHROOM2; SLAMF9; SNX25; SPTY2D1; SRC; ST18; TNC; VARS; VASH2; WNT2B; ZFPM2; ZNF385D; ZNF516; ETNK2; SGSM2; |
| JUN | 14.46124764 | 1.257631865 | 0.001365208 | C5orf64; FGF9; FLRT2; GOLGA3; H3F3B; MARK1; PIM1; RFFL; SEMA3A; ABHD2; ACTB; ANP32E; ANXA2; BACH2; BLCAP; BSCL2; C1GALT1; C5orf51; CASK; CDK14; CHSY1; CLTC; CORO1C; CPEB1; CTTNBP2NL; CXCL11; EDN1; ELF1; ELMO1; FLI1; FNBP1L; GJA1; GLS; KALRN; KTN1; LASP1; MXD1; NAB1; NET1; NR4A2; OSBPL7; PCDH17; PDGFA; PEA15; RIT2; RYBP; SDCBP; SH3BGRL3; SKIDA1; SNAI2; SNAP25; SPRED1; TMCC1; TMSB4X; UTRN; VASP; ZC2HC1C; ZNF800; ARPC2; CD2AP; CPNE8; DLG5; EVI5; FRMD5; NEK6; PALM2-AKAP2; PTEN; ARL8B; CCR7; FTL; GNAI1; GRASP; HECTD2; IRF6; KITLG; NABP1; NPAS2; RAC1; SCOC; STAT4; TPD52L2; VIM; DGKB; RTCB; SEPT8; SLCO3A1; USF1; YBX3; AAK1; CAPN6; DIRAS1; GAB2; HM13; INPP5K; MACF1; RAB37; SLC4A5; ACSL6; ACVR1; AHCYL1; AJUBA; ANXA7; BCAP31; BEND6; CA7; CAPN2; CHST1; CTNNA3; DENND1A; DMRT3; DNAJB5; DR1; DTX2; DUSP10; EHBP1L1; ENHO; ERG; ESRRA; FHL3; GPR158; GRM7; HIC1; KCNAB3; KDM4A; KIAA1671; MARK3; MPP1; MSI1; NECAB3; NRXN1; PDE1B; PPARGC1A; PPP1R14B; PRKAA1; RASIP1; RCAN2; SCRT2; SGPL1; SHROOM2; SLAMF9; SNX25; SPTY2D1; SRC; ST18; TNC; VARS; VASH2; WNT2B; ZFPM2; ZNF385D; ZNF516; ETNK2; SGSM2; |
| REST | 7.088846881 | 1.41357168 | 0.001398371 | HCN1; MAML3; SEMA3A; ST5; STK4; TSC22D2; ANKRD29; ASPH; BACH2; BDNF; CERS2; COL4A3BP; CTBP2; DLG4; ELMO1; FAM155A; FGF17; FNBP1L; GLCCI1; GPR6; JARID2; MAB21L1; NR4A2; PCDH17; PEA15; RNF145; SFPQ; SNAP25; TGIF2; TNKS2; ZNF148; ARPC2; CBFB; LMO3; ARGLU1; CHL1; COX4I1; MBD5; PTPN12; ZBTB18; CAD; DYNLL2; EPHA10; INPP5K; KCNA6; LARGE; MACF1; PCDH1; PPM1N; PRELP; RNF5; RTN2; BAZ2A; C18orf25; CHGA; CXCL12; DFFB; EIF4G2; GRIN2A; GRIP1; GRM7; HLF; KCNAB3; LRRC10B; NEUROD4; NRXN1; PPARGC1A; PRADC1; PRKAB1; PRR16; RPL28; SLC17A6; ZC3H6; ZNF385D; ATP2B2; |
| HOXB4 | 6.710775047 | 1.425787498 | 0.001486892 | ARHGAP44; MAP3K7; SEMA3A; SORCS1; TWF1; ACER2; ADAM12; ARFIP1; CASK; CBL; ELF1; ELMO1; EYA4; FBXW7; FN1; GLS; GPD2; GPR6; KALRN; MAB21L1; MAP4K3; NBEA; OTX2; PLEKHA1; RARB; SEC63; SMIM14; SP2; TMEM178A; TMSB4X; UTRN; VAMP4; ZNF800; MARCKS; VAV3; CNKSR2; HECTD2; KITLG; SMIM15; ARHGEF10L; DES; KCNA4; MYT1L; N4BP2L1; NONO; NOVA1; PPP1CB; SCAI; CAPN6; LUZP1; MACF1; NRG1; SLC4A5; ACTN2; ANO4; BAZ1A; CXXC4; ESRRG; FAM126B; LBX1; MAPK10; MITF; NREP; NRXN1; PPARGC1A; PRDM1; PRKAB1; RNF38; SSFA2; SYT1; VMA21; |
| HNF1A | 7.561436673 | 1.388045821 | 0.001641897 | BTAF1; CTDSPL2; FGF9; PIK3R1; SEMA3A; STK4; TANC1; BDNF; CTBP2; CTTNBP2NL; ELF1; GLS; GPR137C; GPR6; HNRNPK; HSPD1; HSPE1; IGF1; KCNIP3; MAP4K3; MTSS1; NET1; PDE7A; PTBP1; RABGAP1L; RRBP1; TAGLN2; UST; UTRN; YRDC; ZNF800; ARPC2; NRG3; TMEM39A; ZFP91; ARMCX2; KITLG; KLF5; LMO3; NPAS2; RAI2; SDHD; TIMM8B; ARHGEF10L; CACNA2D1; CHL1; HNF4G; KCNA4; MLIP; MYT1L; PTPN12; RAB23; UBE2G1; MACF1; NRG1; RPRD2; SMG7; BEND3; CXXC4; ESRRG; GLIS2; GORASP2; HOXD10; MBNL2; MITF; NR1H4; NREP; NRXN1; PPARGC1A; PRDM1; RCAN2; RREB1; SLC12A2; SLC17A6; SLC30A4; SRC; SRSF6; SSFA2; ZNF385D; BCAS3; |
| NHLH1 | 9.924385633 | 1.323325314 | 0.001662102 | ARHGAP44; ARHGAP5; CELF2; ST5; ACER2; ACTB; C9orf152; CDC42SE1; CERS2; CTBP2; EHMT2; GPD2; HELZ2; KALRN; KCNA5; KCNJ2; MIER1; MPP5; NBEA; NECAB1; NR4A2; OSBPL7; PICALM; PTPRS; SLC29A3; TACR1; TAGLN2; TMEM178A; TMSB4X; FOXN2; FRMD5; MAP3K3; NEK6; ZFP91; CDK13; DDX42; GRASP; KITLG; KLF13; KLHL36; PBX3; PCSK7; PLK3; RBM24; TMEM108; TSC22D4; ARHGEF10L; B4GALT7; CELSR2; DGKB; PABPC1L; PIP5KL1; SEPT8; SLCO3A1; TBC1D8; USF1; DYNLL2; MACF1; NGFR; POLL; PPP2R5B; RNF44; SLC4A5; SMG7; ACSL6; AKT2; B3GALT2; BAZ2A; CEND1; CSNK1A1; DCAF8; DENND1A; DNAJB5; DSP; DUSP10; EFNA3; EIF4G2; ERG; FHL3; FMNL2; IFT20; KDM4A; LRRC10B; LURAP1L; MTA3; NAT8L; NECAB3; OSER1; PPARGC1A; PPTC7; PXN; RNF165; RPL28; SAMD8; SASH3; SCRT2; SLC46A3; SYT1; TBX3; TNC; TNFAIP1; TPCN1; WBP1L; ZNF385D; MEGF8; |
| HENMT1 | 9.924385633 | 1.323325314 | 0.001662102 | ARHGAP44; ARHGAP5; CELF2; ST5; ACER2; ACTB; C9orf152; CDC42SE1; CERS2; CTBP2; EHMT2; GPD2; HELZ2; KALRN; KCNA5; KCNJ2; MIER1; MPP5; NBEA; NECAB1; NR4A2; OSBPL7; PICALM; PTPRS; SLC29A3; TACR1; TAGLN2; TMEM178A; TMSB4X; FOXN2; FRMD5; MAP3K3; NEK6; ZFP91; CDK13; DDX42; GRASP; KITLG; KLF13; KLHL36; PBX3; PCSK7; PLK3; RBM24; TMEM108; TSC22D4; ARHGEF10L; B4GALT7; CELSR2; DGKB; PABPC1L; PIP5KL1; SEPT8; SLCO3A1; TBC1D8; USF1; DYNLL2; MACF1; NGFR; POLL; PPP2R5B; RNF44; SLC4A5; SMG7; ACSL6; AKT2; B3GALT2; BAZ2A; CEND1; CSNK1A1; DCAF8; DENND1A; DNAJB5; DSP; DUSP10; EFNA3; EIF4G2; ERG; FHL3; FMNL2; IFT20; KDM4A; LRRC10B; LURAP1L; MTA3; NAT8L; NECAB3; OSER1; PPARGC1A; PPTC7; PXN; RNF165; RPL28; SAMD8; SASH3; SCRT2; SLC46A3; SYT1; TBX3; TNC; TNFAIP1; TPCN1; WBP1L; ZNF385D; MEGF8; |
| NR1H3 | 8.979206049 | 1.342859794 | 0.001766738 | COPS7B; SMARCD2; TWF1; ABCB7; ACER2; ADPGK; ASPH; BDNF; BET1; BSCL2; CASK; CLCN3; CTBP2; DLG4; ELF1; ELMO1; FNBP1L; GLCCI1; IGF1; JARID2; KALRN; LASP1; MOB3C; MPP5; MSANTD2; PTPRS; RARB; RNF145; RRBP1; SH3BGRL; SKIDA1; SLC25A22; SLC45A4; TIMP3; TMEM135; TPM3; TSPAN4; YWHAZ; ZBTB8A; ELOVL6; FRMD5; NRG3; PALM2-AKAP2; SLC22A3; TOMM70A; CD274; DBN1; LMO3; PBX3; RAP1A; CHL1; DMRT2; FAM120A; FRMD3; MBD5; NOVA1; RNF114; SLC30A9; CAD; CNTFR; DYRK1B; GAB2; INPP5K; NYNRIN; PPFIA3; RNF44; SLC30A3; SLC4A5; ACSL6; ATP1B1; CXXC4; DNAJB5; ELOVL1; EPHA7; ESRRA; HOXD10; KIT; KLF15; MBNL2; MITF; MYO1C; NPC1; NRXN1; NRXN3; PPARGC1A; PPTC7; RAB11B; RREB1; SSFA2; TPCN1; WNT2B; ZNF385D; ZNF710; MAPK4; SETD8; |
| GABPA | 21.07750473 | 1.189880009 | 0.002023091 | ABHD17A; AFF3; BTAF1; DCBLD2; EIF5A2; GOLGA3; H3F3B; HAT1; MAP3K7; PRRC2C; SEMA3A; STK4; TWF1; ABCB7; ACTB; AKAP11; AKAP12; ANKRD34B; ARCN1; ARFIP1; ARPC3; BAG4; CAAP1; CD164; CDC42; CDK4; CEP44; COL4A3BP; CORO1C; CPEB1; CPLX2; CTTNBP2NL; DCAF12L1; DDX5; DLG4; EAF1; EIF1AD; EIF4E; ELOF1; FAM63B; FAM91A1; FBXO33; G6PD; GPD2; HNRNPU; HS3ST3B1; HSP90B1; KALRN; LASP1; LRRC8A; MAN1C1; METTL23; MMD; MON2; MSANTD2; NCK2; NCL; NME1-NME2; NME2; NR1H3; NR4A2; NXT2; OSTF1; PHAX; PHF6; PIGF; POGK; PPIB; PTBP1; RAB43; RAB5A; RABEPK; RRBP1; SERP1; SH3GL1; SLC10A7; SLC35B3; SNAP25; SP2; TERF2; TIGD6; TMEM68; TMX1; TNKS2; TPK1; UTRN; WDR1; WDR61; XPO6; ZNF26; ZNF580; ZNF597; ARPC2; CCDC6; CCNG1; CPNE8; ELOVL6; GATAD1; NEK6; RAP2C; TOMM70A; CDK13; DBN1; FOXQ1; KLHL36; MYL12A; PAPOLA; PBX3; PCSK7; SCOC; SDHD; SMIM15; SPOPL; TIMM8B; TUSC3; VDAC1; VPS37B; ARGLU1; CACNA2D1; DCAF12; FOPNL; FRS2; KIAA1429; LEMD3; LSM5; METTL21A; PVALB; RAB14; SEPT8; SLC30A9; SLC35G1; TFG; UBE2G1; UBE2Q1; UBLCP1; USF1; APOLD1; ATG2B; CCAR2; EPN1; HM13; HMGA1; LDLRAP1; LYPLA2; LZIC; POLL; PPAN; RAB4B; STOML1; TGFB1; AKT2; APPL2; ARHGEF18; BCAP31; BNIP2; C18orf25; C6orf47; CABLES2; CDC37L1; CSNK1A1; CUL4A; CYB5R4; DENND1A; DTX2; E2F7; EBAG9; EN2; ETFB; FAM172A; FSTL1; GABRA1; GORASP2; HERPUD2; HIC1; HOXC4; ICMT; ING2; KDM5B; MAPKAPK2; MARK3; MBTPS2; MIA3; MTF1; NCOA2; NECAB3; NECAP1; NIPBL; NKPD1; NPC1; PLCB1; PPP1R14B; PRADC1; PRKAB1; PTPN2; QKI; RAB11B; RANBP1; RASIP1; RNF4; RPGRIP1L; RPL28; RPL37; RREB1; SASH3; SDR39U1; SGPL1; SIRT6; SLC12A2; SLC25A5; SLC35A4; SLC46A3; SNX25; SPTY2D1; STAC; SZRD1; TAF12; TMEM33; TSNAX; UBXN4; ZFYVE16; LAS1L; SETD8; SGSM2; |
| MYF5 | 15.02835539 | 1.235122144 | 0.002324775 | ARHGAP44; BTBD3; FGF9; H3F3B; SMOC1; ST5; TXLNG; ACER2; AKAP12; C1GALT1; C9orf152; CPEB1; CTBP2; CTTNBP2NL; DLG4; E2F5; ELF1; ELMO1; EPB41L4B; ETS1; EYA4; FAM107B; FBXW7; FGF17; FLI1; G6PD; GCFC2; GCH1; HACE1; HIAT1; IGF1; KALRN; KCNIP3; KCNJ2; KRAS; LRCH1; MAN1C1; MCTP1; METTL23; MIER1; MTX1; NETO1; NR1H3; RABGAP1L; RRBP1; SERP1; SLC29A3; SLC35F1; SLC44A1; SMARCB1; STC2; TACR1; TMCC1; TMEM178A; TPM3; TTR; VASP; YPEL2; YWHAZ; ZC2HC1C; CTNNBIP1; KLF10; NEK6; NMNAT3; ARL8B; CD274; DBN1; DESI2; DLX1; FUS; HECTD2; KCNS3; LMO3; NPAS2; RAI2; TSC22D4; YWHAH; CACNA2D1; CHTOP; DMRT2; GNAQ; IPO9; KCNA4; MBD5; MLIP; NOL4; NONO; NOVA1; PELI3; RAB14; SCN1A; UBE2Q1; USF1; AAK1; ADAMTS10; ATG2B; DYRK1B; GMPR; HM13; HMGA1; INPP5K; LARGE; MACF1; NRG1; NUMBL; PCDH1; PLEKHA2; POLL; PPAN; RIMS4; VKORC1L1; ACVR1; AKT2; ANKRD13A; ATP1B1; C18orf25; CA7; CHGA; CTDSP2; DENND1A; DNAJB5; DSP; EHBP1L1; EPHA5; ESRRG; EZH2; GABRA1; GORASP2; GREM2; GRIN2A; GRIP1; KIT; L3MBTL4; LBX1; LMBR1L; MLC1; MTA3; NPC1; NRXN3; PDE1B; PRDM1; RCAN2; SAMD8; SCRT2; SLAMF9; STK3; SZRD1; TCF4; TDRD7; TFAP2C; TNC; UGCG; WNT2B; WNT7A; YTHDC1; ZC3H6; ZNF385D; ATP2B2; KLHL14; |
| PRRX2 | 5.671077505 | 1.449131522 | 0.002327579 | MAP3K7; SEMA3A; SORCS1; CASK; CDK14; CDK4; CLTC; DDX5; ELF1; ELMO1; FN1; HIAT1; MAB21L1; MAP4K3; MIER1; MXD1; NR4A2; PIGF; RABGAP1L; RAP1B; SH3BGRL; SMAP1; TMEM178A; TMSB4X; UTRN; CD2AP; HECTD2; KITLG; LMO3; NABP1; PBX3; STAT4; ARHGEF10L; CACNA2D1; DGKB; MTUS1; NOL4; PTPN12; CAPN6; MACF1; NRG1; ACVR1; CNEP1R1; CTNNA3; CXXC4; ESRRG; GRIN2A; GRIP1; HOXD10; HTR2C; MBNL2; MITF; MTA3; NRXN1; PIAS2; PLCB1; RNF38; SIPA1L2; TAF12; ZFPM2; |
| ISL2 | 5.009451796 | 1.483911241 | 0.002489449 | ARHGAP44; HAT1; SEMA3A; SORCS1; BET1; CDK14; CLTC; EYA4; GJA1; KALRN; NETO1; NR4A2; PAX3; PHF6; RABGAP1L; RARB; RIT2; SKIDA1; THBS1; UTRN; EVI5; MARCKS; PTEN; GNAI1; KLF5; LMO3; RAI2; SCOC; VIM; CACNA2D1; CHL1; GNG12; MYT1L; NTPCR; SCN1A; HIPK1; MACF1; MLLT3; ADAM23; BAZ1A; DENND1A; FAM98A; GRIP1; GRM7; MAPK10; MITF; NRXN1; PLCB1; PRKAB1; PTPN5; RAPGEF5; SSBP3; UBXN4; |
| TLX2 | 1.039697543 | 2.645023133 | 0.002505415 | ATG13; EYA4; FUBP1; TNPO2; DNAJB4; IRF6; KLF5; ARGLU1; NUFIP2; HOXD10; KIAA1671; |
| POU3F4 | 3.780718336 | 1.575946183 | 0.002853249 | ARHGAP44; BTBD3; SEMA3A; SORCS1; ABCB7; CDK14; EYA4; HNRNPK; KALRN; NETO1; OTX2; RARB; TMEM178A; UST; UTRN; MARCKS; NRG3; PTEN; KITLG; LMO3; VIM; KCNA4; MBD5; MYT1L; NOVA1; PPM1B; ZBTB18; MACF1; BAZ1A; CXXC4; EPHA5; FAM126B; GRM7; HOXD10; NRXN1; PPARGC1A; PRKAB1; RNF38; SCRT2; SLC6A9; |
| SPI1 | 7.183364839 | 1.346191285 | 0.004657228 | SMAD2; STK4; ARPC3; BRI3BP; CALM2; CASK; CBL; CDC42SE1; CTTNBP2NL; EIF1AD; ELMO1; FBXW7; FN1; FNBP1L; KCNJ2; MSANTD2; OSTF1; PAX3; PEA15; RABGAP1L; RRAGC; SNAP25; TMEM178A; TMSB4X; USP33; VASP; ARPC2; FOXN2; NEK6; PDIK1L; XRCC5; ARPP19; EMC7; GRASP; ARHGEF10L; DCAF12; FKBP1A; MYT1L; PTPN12; TM6SF1; USF1; ZBTB18; FBRS; NUAK2; TSFM; WASF2; ADCY2; BAZ2A; BNIP3L; CYB5R4; DENND1A; ESRRA; HIC1; MBNL2; MITF; MTF1; NRXN1; NRXN3; NSF; PDE1B; PRDM1; PRKAB1; RPL37; RRM2B; SAMD8; SCRT2; SIPA1L2; SLC30A4; SLMAP; SPTY2D1; SRPR; TBC1D12; TFAP2A; TNC; TWIST1; BCAS3; |
| ZEB1 | 3.969754253 | 1.510300135 | 0.00489608 | RFFL; SEMA3A; CDK14; CLTC; CPEB1; CTBP2; CXCR4; HSP90B1; NR4A2; OTX2; PDE7A; THBS1; USP33; VAMP2; CD2AP; IRF6; KITLG; MYL12A; PHF1; ARHGEF10L; FAF1; FAM169A; MTUS1; N4BP2L1; NGFR; NUMBL; PHC2; SMG7; THRA; TSFM; ZC3H7B; EFNA3; ENHO; HLF; LINGO2; RAPGEF5; SASH3; SIPA1L2; TFAP2A; ZNF710; ATP2B2; MAPK4; |
| POU6F1 | 5.95463138 | 1.386843105 | 0.005031237 | ARMC8; BTBD3; COPS7B; MAP3K7; SEMA3A; CHSY1; CLTC; CTBP2; FN1; FUBP1; HNRNPK; HSP90B1; JARID2; KALRN; MIER1; RNF111; RRAGC; SEC63; SMARCB1; TAGLN2; TMEM178A; TTR; UST; ZC2HC1C; ZNF800; CCNG1; EVI5; NRG3; PTEN; DDX42; KLF5; LMO3; NKX2-4; NPAS2; ARGLU1; CACNA2D1; MACROD2; MBD5; NOL4; NOVA1; SCN1A; ATP8B2; IPO11; LDLRAP1; BEND6; CACFD1; CD69; CXXC4; DENND1A; DUSP1; EN2; ESRRG; FMNL2; KDM1A; LBX1; MAPK10; MBNL2; NRXN1; PPARGC1A; SSBP3; TDRD7; ZFPM2; MAPK4; |
| CTCF | 20.03780718 | 1.173309896 | 0.005141601 | COPS7B; DCBLD2; DYNLL1; GPX8; HCN1; PIK3R1; PIM1; PRRC2C; RFFL; SORCS1; ACTB; AKAP12; ANP32B; ANXA4; ARG2; ATG13; ATP6V1A; BDNF; BLCAP; CASK; CBL; CD164; CDK14; CITED2; CLCN3; CPEB1; CXCR4; DDX5; DGKH; DGKZ; DLG4; EDN1; EHMT2; ELMO1; ELOF1; EPB41L4B; ETS1; FGF17; G6PD; GCH1; GDF6; GPR6; HIAT1; IGF1; KALRN; KCNIP3; KCNJ2; LASP1; LRCH1; LRRC59; MAN1C1; MAP4K3; MIER1; MTX1; NR1H3; PTBP1; RRBP1; SH3GL1; SLC29A3; SRSF9; SYN3; TACR1; TAGLN2; THBS1; TIMP3; ARPC2; CNIH1; CPNE8; EFNB2; FBXO9; KCNG3; MARCKS; NEK6; OPRK1; RAP2C; ZFP91; ARL8B; BANP; CCR7; CNKSR2; COPS2; DBN1; GRASP; KLHDC9; MDK; NAA20; PLEKHA5; TSC22D4; VDAC1; ATP10D; B4GALT7; CACNA2D1; CELSR2; COL1A1; DGKB; FRMD3; GNAQ; HNF4G; LSM5; MAFB; MTHFD1L; MTUS1; MYT1L; PABPC1L; SCAI; SEPT8; SLC35F5; SLCO3A1; UBE2Q1; ACTR1A; AMMECR1L; BBC3; CAD; CNTFR; DYNLL2; EPHA10; HIPK1; HM13; HOXB6; INPP5K; KAZALD1; KCTD15; KMT2B; LARGE; LZIC; NGFR; NRG1; NUMBL; PCDH1; PHC2; PLEKHA2; RAB37; RIMS4; SLC30A3; SLC4A5; SRF; TGFB1; TSFM; AACS; ACSL6; ACTN2; AHCYL1; AKT2; ALDH1A2; ATP1B1; BAZ2A; BEND3; C18orf25; C7orf31; CAPN2; CDC42EP2; CTNNA3; CXCL12; DCDC2; DENND1A; DEXI; DTX2; DUSP1; EFNA3; EHBP1L1; EIF2S3; ELOVL2; EPHA5; ESRRA; FAM196B; FITM2; GLIS2; GORASP2; GPR158; GRIN2A; HIC1; IDH1; KDELR3; KDM4A; MARCH8; MITF; MPC1; MSI1; MTA3; MTF1; MYO1D; NCOA2; NECAB3; NPC1; NRXN1; PLCB1; PPTC7; PRKAB1; PTPN5; PXN; RCAN2; RCOR2; SAMD8; SGPL1; SLC25A5; SLC30A4; SLC43A2; SLC6A8; SLMAP; SRC; SS18; ST18; TAF7L; TCTN3; TDRD7; TFAP2A; TFAP2C; WIF1; WNT2B; CYB561D1; ITGA6; MEGF8; |
| SPDEF | 2.173913043 | 1.755082836 | 0.006010216 | ARID4B; SMARCD2; ARFIP1; DLG4; EAF1; FN1; HSP90B1; HSPD1; MAP4K3; MIER1; SKIDA1; YWHAZ; TUSC3; TFG; UBLCP1; USF1; WNK4; LYPLA2; BCAP31; CEP63; MOB4; PIAS4; SETD8; |
| HOXA7 | 4.914933837 | 1.422766989 | 0.006319654 | SEMA3A; TWF1; ACER2; ARFIP1; CASK; ELMO1; EYA4; GPD2; GPR6; KALRN; LRCH1; MAP4K3; OTX2; RABGAP1L; RARB; SEC63; SMIM14; SP2; TMEM178A; UTRN; MARCKS; LMO3; SMIM15; ARHGEF10L; DES; FRS2; GNG12; MTHFD1L; MYT1L; NOVA1; PPP1CB; CAPN6; HIPK1; MLLT3; PLEKHA2; ACTN2; BAZ1A; ESRRG; FAM126B; GRIP1; GRM7; LBX1; MAPK10; MITF; NRXN1; NRXN3; PPARGC1A; RNF38; SCRT2; SYT1; TNC; VMA21; |
| PAX7 | 4.442344045 | 1.451208634 | 0.006411188 | RFFL; SEMA3A; SORCS1; CDK14; CDK4; EYA4; FNBP1L; GLS; GPR137C; GPR6; KALRN; MAP4K3; PDE7A; PLEKHA1; RABGAP1L; TMEM178A; UTRN; EVI5; MARCKS; HECTD2; LMO3; NPAS2; SMIM15; VIM; CACNA2D1; FAM169A; MBD5; PPP1CB; CAPN6; LUZP1; MLLT3; ACVR1; BAZ1A; CD69; CTNNA3; DENND1A; FAM126B; HERPUD2; MITF; MPC1; NREP; NRXN1; PDE1B; PHF3; PRDM1; PRKAB1; ZMAT4; |
| ONECUT1 | 5.198487713 | 1.406114294 | 0.006426054 | HCN1; RFFL; TANC1; ADAM12; ARID2; CTBP2; EDN1; EYA4; GPD2; KALRN; NR1H3; RARB; SEC63; TTR; CCNT2; EFNB2; MARCKS; PTEN; KLF5; PBX3; RAC1; RAI2; ARGLU1; CACNA2D1; CHL1; DGKB; HNF4G; MACROD2; MBD5; MYT1L; NDFIP1; NOVA1; AAK1; HIPK1; KCTD15; ACSL6; AHR; ERG; ESRRG; GORASP2; GRIP1; HOXD10; LINGO2; MAPK10; MARCH7; MITF; NEUROD4; NRXN1; PTGFRN; RAPGEF5; SIK1; SLC12A2; TNC; ZNF385D; KLHL14; |
| CUX1 | 3.497164461 | 1.524419217 | 0.006861069 | PRRC2C; ARID2; BET1; C5orf51; CAPZA1; GLCCI1; GPD2; GPR137C; MPZL1; NR4A2; TACR1; TTR; UTRN; ELOVL6; RAP2C; DLX1; INSM2; PLEKHA5; TMEM108; CHL1; FRMD3; FRS2; MTHFD1L; MTUS1; N4BP2L1; NDFIP1; PTPN12; NRG1; BAZ1A; BNIP2; GRIP1; HOXD10; SLC6A9; SYT1; TNC; ZNF516; KLHL14; |
| HERPUD1 | 4.064272212 | 1.472796738 | 0.006901586 | HAT1; ARID2; BDNF; CLTC; CORO1C; DLG4; E2F5; ELF1; FBXW7; KCNA5; LASP1; NR4A2; USP33; ZNF800; CCDC6; DLG5; TOMM70A; CNKSR2; DBN1; NAA20; NOL4; ZBTB18; HIPK1; HM13; LYPLA2; ONECUT2; PHC2; ACSL6; CAPN2; E2F7; ESRRG; LURAP1L; MYO1C; PRR16; RCAN2; RNF38; SLMAP; TCTN3; THNSL1; TPCN1; VMA21; ZC3H3; KIF3B; |
| PPARG | 15.78449905 | 1.194305896 | 0.006931352 | MARK1; SMARCD2; SMOC1; ST6GALNAC6; TANC1; TXLNG; ABCB7; ACER2; ADPGK; ARPC3; ASPH; AXL; BAG4; BDNF; BET1; CASK; CBL; CNN3; CTTNBP2NL; DLG4; ELMO1; EYA4; FN1; G6PD; GLCCI1; GLS; HSPD1; HSPE1; IGF1; KALRN; KCNIP3; LASP1; LRRC8A; MAN1C1; MMD; MMD2; MOB3C; MPP5; MXD4; NDFIP2; NR1H3; NR4A2; PAX3; PGD; RARB; RRBP1; RYBP; SFPQ; SKIDA1; SLC25A22; SP2; SRSF1; TAGLN2; TIMP3; TMEM135; TNKS2; USP33; UTRN; VASP; YRDC; ZNF148; ZNF800; ARPC2; C6orf106; KLF10; NEK6; SLC22A3; TOMM70A; ZFP91; CDK13; ETFA; FOXQ1; FUS; KCNS3; KITLG; NPAS2; PPCS; RAP1A; ARHGEF10L; CACNA2D1; DMRT2; F10; MTHFD1L; MTUS1; NIPAL1; NOVA1; PELI3; RNF114; RTCB; SLC35G1; UBE2Q1; USF1; AMMECR1L; CAPN6; CNTFR; GAB2; INPP5K; JDP2; LARGE; LDLRAP1; LYPLA2; MACF1; NYNRIN; PCDH1; PHC2; PPFIA3; RAB37; WASF2; AGPAT3; AHCYL1; CSNK1A1; CXCL12; CXXC4; DENND1A; DTX2; EFNA3; ELOVL1; EPHA7; ESRRA; ETFB; FITM2; FMNL2; FURIN; HNRNPDL; HOXD10; HTR2C; IDH1; KLF15; LRRC10B; MBNL2; MITF; MYO1C; NCOA2; NEUROD1; PEX3; PPARGC1A; PPTC7; PTPN5; PXN; RANBP1; RAPGEF5; RASIP1; RNF165; RREB1; SAMD8; SASH3; SHROOM2; SLC25A5; SLC6A8; SLC6A9; SSBP3; SSFA2; ST3GAL3; SYNDIG1; TMEM218; TPCN1; UBXN4; USF2; VAX1; WNT2B; YBX1; ZFPM2; ZNF385D; ATP2B2; ETNK2; MTSS1L; SETD8; |
| FOXO1 | 2.173913043 | 1.727661095 | 0.007253197 | ARMC8; FOXO1; AKAP12; BLCAP; CITED2; GLCCI1; NR4A2; YPEL2; CD2AP; LMO3; PLEKHA5; HEXIM1; AAK1; INPP5K; CTDSP2; CXXC4; ERG; FHL3; MAPK10; NRXN3; PRDM1; SSBP3; TWIST1; |
| FOXA1 | 6.994328922 | 1.327072765 | 0.007288535 | ARHGAP44; FGF9; HAT1; MAP3K7; NFE2L1; RFFL; ST5; AKAP12; ATG13; CITED2; EIF4E; ELMO1; FN1; FUBP1; GPD2; HNRNPK; IGF1; MAN1C1; PCDH17; RARB; SKIDA1; SNAP25; TPM3; TTR; UST; YRDC; ARPC2; FRMD5; PTEN; CDH20; FOXQ1; KITLG; KLF5; LMO3; PBX3; PLEKHA5; SMIM15; CACNA2D1; CHTOP; DGKB; MACROD2; MBD5; MTUS1; MYT1L; NOVA1; ZBTB18; MACF1; NUAK2; ONECUT2; AHCYL1; ALDH1A2; ANO4; BNIP2; CXXC4; DUSP1; DUSP4; ERG; ESRRG; GABRA1; MAFK; MAPK10; MBNL2; MED27; MITF; PPARGC1A; PRDM1; PROX1; RAPGEF5; SSBP3; SSFA2; WBP1L; WNT2B; ZMAT4; ATP2B2; |
| MAFB | 3.497164461 | 1.51575799 | 0.007500147 | BTBD3; HAT1; SORCS1; BDNF; CNN3; ELF1; FBXW7; FLI1; GJA1; HACE1; KRAS; OTX2; TMCC1; TTR; UTRN; ZBTB8A; FRMD5; NEK6; PALM2-AKAP2; ARGLU1; CHL1; MBD5; MTHFD1L; NUFIP2; SCAI; ATG2B; NYNRIN; SLC4A5; DENND1A; ESRRG; MAPK10; MITF; NRXN1; NRXN3; RNF38; SGPL1; TBC1D12; |
| RFX1 | 16.72967864 | 1.182524457 | 0.008008654 | ARHGAP44; BTAF1; BTBD3; C5orf64; DYNLL1; EIF5A2; FGF9; PIK3R1; RAPGEFL1; RFFL; SORCS1; AKAP11; ANXA2; ARFIP1; ARG2; BDNF; BSCL2; CDK4; CLTC; COL4A3BP; CTBP2; DLG4; ELMO1; FAM107B; FBXL14; FBXW7; FGF17; FOSB; FZD7; HSP90B1; KALRN; KCNJ2; KLF4; MMD; MTX1; MXD1; MXD4; NECAB1; NR4A2; PAX3; PCDH17; PEA15; PLEKHA1; RIT2; RNF138; SEC63; SFPQ; SH3GL1; SLC25A22; SLC44A1; TAGLN2; TPM3; TSPAN4; TTR; USP33; UTRN; YPEL2; YWHAQ; ARPC2; CCDC6; DLG5; EVI5; FOXN2; MARCKS; OPRK1; PALM2-AKAP2; PTEN; SEMA4B; TOMM70A; VAV3; XRCC5; BANP; CDH20; COPS2; DBN1; GRASP; LMO3; PAPD5; PBX3; POLR1C; SLC28A3; STAT4; TSC22D4; CACNA2D1; COL1A1; DGKB; FAF1; MBD5; MTUS1; MYT1L; N4BP2L1; NONO; NTPCR; PABPC1L; PTPN12; SEC61A2; SEPT8; SLC35F5; TBC1D8; USF1; ZBTB18; AAK1; CCAR2; DYRK1B; EPHA10; HIPK1; HM13; IPO11; LARGE; LYPLA2; PCDH1; PHC2; POLL; PPAN; PRRT2; RAB4B; RNF5; SLC4A5; SMG7; THRA; ACSL6; ACVR1; AIDA; AKT2; B3GALT2; BAZ2A; BEND3; BNIP2; CACFD1; CTDSP2; DENND1A; E2F7; EFNA3; ESRRA; FAM196B; FAM98A; FXYD6; GALNT1; GRM7; HIC1; HOXC4; HOXD10; ING2; KDM4A; LRRC10B; MAFK; MAPK10; MARK3; METTL9; MIA3; MITF; MPC1; PLCB1; PPP1R37; PRKAA1; RCAN2; RPGRIP1L; RREB1; SCRT2; SIK1; SIPA1L2; SLC17A6; SLC5A7; SLC6A8; SRC; SWT1; TCTN3; THNSL1; TMED5; TMEM218; TMEM229B; TPCN1; UBXN4; WBP1L; ZMAT4; ATP2B2; KIF3B; |
| VSX2 | 5.387523629 | 1.381314462 | 0.008014865 | AFF3; ARID4B; BTBD3; DCBLD2; ST5; BSCL2; CTBP2; ELF1; EYA4; FAM155A; FN1; GPR137C; HIAT1; KALRN; MAB21L1; MAP4K3; RABGAP1L; RARB; SEC23B; SEC63; SKIDA1; TMEM178A; UTRN; VAMP4; ZBTB8A; ZNF800; CCNG1; EVI5; PTEN; CDH20; CDK13; DLX1; PBX3; RAC1; CACNA2D1; FAM169A; MBD5; MTUS1; PTPN12; SCN1A; CAPN6; ECHDC1; LDLRAP1; LUZP1; ABHD5; CNEP1R1; CTNNA3; ESRRG; LBX1; MITF; PDE1B; SIPA1L2; SSFA2; UGCG; ZMAT4; KLHL14; ZC3H4; |
| ARNT | 3.875236295 | 1.46703248 | 0.008747271 | PIM1; SMAD2; BDNF; CD164; CDK4; CPEB1; CPLX2; DDX5; EIF4E; FBXO33; NET1; NME1-NME2; NME2; PICALM; POGK; PTBP1; PTPRS; ATP6V1G1; PTEN; BANP; RGS9BP; FAF1; HNF4G; MBD5; UBE2Q1; AMMECR1L; BBC3; CCNG2; DUSP1; DVL2; HOXD10; LAPTM4B; LONRF3; NAB2; NEUROD1; NKAIN1; RAB9A; SSR1; THNSL1; UNC79; VARS; |
| CDX2 | 2.646502836 | 1.596030567 | 0.009620907 | AFF3; FGF9; HAT1; MAP3K7; PIK3R1; SP5; BLCAP; CTBP2; HNRNPK; HOXB4; MAN1C1; NDFIP2; SKIDA1; THBS1; UST; UTRN; CCDC6; DNAJB4; MARCKS; DLX1; FOXQ1; LMO3; PBX3; NIPAL1; DCAF8; GRM7; ZFPM2; ZNF385D; |
| MAF | 4.347826087 | 1.423386294 | 0.009746595 | FGF9; H3F3B; PIM1; ANP32E; BACH2; BSCL2; CASK; CDC42SE1; CDK14; CPEB1; GJA1; HNRNPK; MPP5; PFN2; RIT2; SNAP25; ZC2HC1C; PTEN; DLX1; FTL; PBX3; RAI2; SCOC; STAT4; CACNA2D1; CHTOP; MACROD2; MYT1L; SLCO3A1; CAPN6; FBRS; MACF1; THRA; AHCYL1; BAZ2A; BCAP31; BEND6; CA7; DUSP10; ESRRG; NECAB3; PRDM1; PRKAA1; RREB1; SHROOM2; WNT2B; |
| RAB40B | 6.899810964 | 1.305893321 | 0.011007921 | ABHD17B; BTBD3; PIM1; SEMA3A; STK4; ADPGK; BDNF; CDK14; CDK4; DGKH; ELMO1; EYA4; GCFC2; LASP1; MOB3C; MSANTD2; NAB1; SMIM14; TMEM135; TNKS2; TPM3; USP33; ARPC2; CCDC6; CNIH1; NEK6; PTEN; TAB3; TOMM70A; CD274; CDH20; CDK13; FUS; HECTD2; LMO3; NPAS2; PBX3; PCSK7; SMIM15; ARGLU1; MBD5; PELI3; SCN1A; SEC61A2; CAD; HIPK1; NRG1; ONECUT2; ACVR1; ATP1B1; BLOC1S5; BNIP3L; CXXC4; DNAJB5; DUSP4; EBAG9; EPHA7; ESRRA; FHL3; GPCPD1; GPR158; IDH1; MAPKAPK2; MBOAT2; MITF; OSER1; PHF3; PLCB1; RANBP1; RAPGEF5; RREB1; TFAP2A; ZFPM2; |
| RARA | 6.899810964 | 1.305893321 | 0.011007921 | ABHD17B; BTBD3; PIM1; SEMA3A; STK4; ADPGK; BDNF; CDK14; CDK4; DGKH; ELMO1; EYA4; GCFC2; LASP1; MOB3C; MSANTD2; NAB1; SMIM14; TMEM135; TNKS2; TPM3; USP33; ARPC2; CCDC6; CNIH1; NEK6; PTEN; TAB3; TOMM70A; CD274; CDH20; CDK13; FUS; HECTD2; LMO3; NPAS2; PBX3; PCSK7; SMIM15; ARGLU1; MBD5; PELI3; SCN1A; SEC61A2; CAD; HIPK1; NRG1; ONECUT2; ACVR1; ATP1B1; BLOC1S5; BNIP3L; CXXC4; DNAJB5; DUSP4; EBAG9; EPHA7; ESRRA; FHL3; GPCPD1; GPR158; IDH1; MAPKAPK2; MBOAT2; MITF; OSER1; PHF3; PLCB1; RANBP1; RAPGEF5; RREB1; TFAP2A; ZFPM2; |
| CEBPD | 1.417769376 | 1.914827252 | 0.011284473 | SORCS1; CITED2; CXCR4; ELMO1; FN1; FOSB; GPR137C; STC2; KLF10; PBX3; APOLD1; DUSP1; EPHA5; MARCH8; PDE1B; |
| BSX | 2.741020794 | 1.560495471 | 0.011453014 | SEMA3A; TANC1; ANP32E; BACH2; CAPZA1; ELF1; IGF1; JARID2; MXD1; SEC23B; ZNF800; MSX1; PALM2-AKAP2; LMO3; NPAS2; PBX3; SCOC; FRS2; MTUS1; MYT1L; HIPK1; LDLRAP1; ONECUT2; ESRRG; MBD6; MBNL2; SIPA1L2; SSBP3; ZFPM2; |
| PRRX1 | 3.686200378 | 1.445709275 | 0.012980033 | SEMA3A; BDNF; CDK4; ELMO1; FBXW7; FN1; GPR6; KALRN; MAP4K3; OTX2; RARB; SH3BGRL; TMEM178A; UTRN; VAMP4; MARCKS; PTEN; VAV3; CDH20; KITLG; KLF5; LMO3; VIM; FRMD3; MBD5; NOL4; NONO; CAPN6; CTNNA3; DENND1A; ESRRG; GRM7; HERPUD2; LBX1; MITF; PRKAB1; RNF38; ST3GAL3; KLHL14; |
| BACH1 | 5.671077505 | 1.335081264 | 0.013132806 | PIM1; ANP32E; ANXA2; BLCAP; C1GALT1; CHSY1; ELF1; FN1; FNBP1L; GJA1; GLS; KLF4; LASP1; NAB1; PDGFA; PEA15; RAB5A; RABGAP1L; RIT2; SDCBP; SH3BGRL3; TAGLN2; EVI5; NEK6; FTL; IRF6; NABP1; SCOC; VIM; CACNA2D1; ESYT2; FRMD3; RTCB; SLCO3A1; AAK1; ATP8B2; BBC3; CAPN6; DIRAS1; GAB2; HMGA1; LYPLA2; ACVR1; ANO4; BCAP31; BEND6; CHST1; CTNNA3; DMRT3; DNAJB5; DR1; DTX2; ERG; FHL3; MAFK; NRXN1; PPARGC1A; SPTY2D1; TNC; VARS; |
| ATF3 | 6.805293006 | 1.297666753 | 0.013204549 | ATXN7L3; SMOC1; TSC22D2; ARG2; AXL; BACH2; BSCL2; C1GALT1; CDC42; CNN3; DLG4; FOSB; GPR6; LIN7C; LRRC8A; MAN1C1; MXD1; MYEF2; PDE7A; PLEKHA1; POGK; PTPRS; TGIF2; TMSB4X; TNKS2; TPM3; TPM4; CD2AP; NEK6; PALM2-AKAP2; TMEM39A; BANP; IRF6; KLF13; LMO3; MSI2; TMEM108; ARHGEF10L; COX4I1; DES; MAPK1IP1L; NOL4; SLC35F5; KCTD15; PPFIA3; RNF44; ATP1B1; BNIP3L; C11orf87; CCNI; DNAJB5; DUS2; DUSP1; DUSP10; FHL3; HLF; INPP5A; KDM4A; KLF11; LUZP2; MBNL2; MOB4; OSER1; PDLIM3; PPARGC1A; RAB11B; SPHK2; SRSF6; YTHDC1; ZBTB21; SGSM2; ZC3H4; |
| E4F1 | 1.79584121 | 1.734456156 | 0.013291857 | BTAF1; MAP3K7; SRSF3; ADPGK; FOSB; NINJ1; PDE7A; PTMA; SH3GL1; TMEM178A; DESI2; VPS37B; ATP10D; SLC30A9; APOLD1; KCNA6; BEND3; KDELR3; YTHDC1; |
| ELK1 | 15.31190926 | 1.176051049 | 0.013518203 | BTAF1; CSPG5; EIF5A2; FOXO1; GOLGA3; GPX8; HAT1; MAP3K7; SEMA3A; TANC1; TXLNG; ACTB; ARCN1; ARFIP1; ARID2; ARPC3; BLCAP; BPNT1; CDK14; CHSY1; COL4A3BP; CORO1C; CTTNBP2NL; DDX5; DLG4; EAF1; EIF1AD; FAM107B; FN1; G6PD; HACE1; HELZ2; HNRNPU; KCNIP3; LASP1; LIN7C; MAN1C1; MAP4K3; MATR3; METTL23; NCK2; NCL; NFATC2; NME1-NME2; NME2; NR1H3; NXT2; PEA15; PFN2; PHAX; PIGF; PLEKHA1; POGK; RAP1B; SERP1; SMIM14; TAGLN2; TERF2; TIGD6; TMEM68; TSPAN4; UTRN; VASP; WDR1; XPO6; ZNF26; ZNF580; ZNF800; ARPC2; CTNNBIP1; ELOVL6; GATAD1; KLF10; MAP3K3; NEK6; PTEN; RAP2C; ARL8B; CDH20; CDK13; KCNS3; MYL12A; NABP1; PAPOLA; PCSK7; RAC1; SCOC; SDHD; SMIM15; SPOPL; STAT4; TIMM8B; VPS37B; CHL1; FRS2; KIAA1429; LEMD3; LSM5; MTHFD1L; NTPCR; SEC61A2; TFG; TM6SF1; UBLCP1; APOLD1; ATG2B; CCAR2; EPN1; HMGA1; ISCA2; LYPLA2; LZIC; MACF1; POLL; PPAN; SMG7; STOML1; ACVR1; ARHGEF18; BCAP31; CDC37L1; CHST9; CSNK1A1; CUL4A; CYB5R4; ETFB; FAM126B; FURIN; GABRA1; GPR158; GRAMD3; HERPUD2; IFT20; KDELR3; KDM5B; MARK3; NAB2; NEUROD1; NIPBL; NSF; PCGF5; PPP1R14B; PTPN5; RAB11B; RASIP1; RPGRIP1L; RPL37; RPS13; SAMD8; SASH3; SIRT6; SLC30A4; SLC35A4; SPTY2D1; TAF12; TFAP2A; TNFAIP1; TSNAX; ZFYVE16; BCAS3; LAS1L; SETD8; |
| JDP2 | 6.52173913 | 1.303909892 | 0.013631924 | STK4; TSC22D2; ARG2; AZIN1; BACH2; BSCL2; CALM2; CDC42; CLCN3; FOSB; GPR137C; GPR6; LRRC8A; MXD1; MYEF2; NINJ1; PDE7A; PLEKHA1; PTPRS; SH3GL1; STX12; TMSB4X; VASP; CCDC6; CD2AP; TAB3; TMEM39A; HECTD2; IRF6; LMO3; MSI2; NPAS2; TRIAP1; VPS37B; YWHAH; COX4I1; LSM5; NOL4; SLC35F5; MACF1; PPFIA3; PPP2R5B; RNF44; AHCYL1; ANO4; BEND6; C11orf87; C2orf57; CCNI; CHGA; DNAJB5; DUS2; DUSP1; DUSP10; HLF; IFT20; INPP5A; KLF11; LUZP2; MAFK; MBNL2; PDLIM3; RAB11B; RCAN2; SPHK2; TNFAIP1; YTHDC1; ZBTB21; ZC3H4; |
| ALX1 | 0.850661626 | 2.319129503 | 0.014228783 | NETO1; LMO3; NPAS2; ZBTB18; CXXC4; EPHA7; ESRRG; MBNL2; SSBP3; |
| FEV | 4.725897921 | 1.37065652 | 0.014267931 | PIK3R1; SMAD2; TWF1; BPNT1; BSCL2; CDC42SE1; CDK14; CLCN3; CLTC; FAM107B; HNRNPK; HNRNPU; KALRN; LIN7C; PLEKHA1; RAB5A; RABGAP1L; RARB; TNKS2; TPM3; XPO6; CCDC6; KCNG3; NEK6; LMO3; NPAS2; RAC1; ARGLU1; GNG12; PTPN12; ZBTB18; APOLD1; HOXB6; LUZP1; MACF1; PCDH1; PHC2; CTNNA3; DR1; DUSP4; EHBP1L1; ERG; FHL3; FSTL1; MTA3; MYO1C; PRDM1; RAPGEF5; RNF165; SS18; |
| TAL1 | 9.073724008 | 1.24360708 | 0.014339943 | ARID4B; COPS7B; H3F3B; SORCS1; TXLNG; ZNF740; ANXA4; BACH2; BLCAP; CDC42SE1; CDK14; CDK4; CPEB1; CTBP2; DLG4; ETS1; EYA4; GLCCI1; HELZ2; HS3ST3B1; LRCH1; MAN1C1; MAP4K3; MMD2; MXD1; MXD4; OSBPL7; SEC61A1; SNAI2; TACR1; TMEM178A; NEK6; TMEM39A; BANP; DBN1; EFS; GRASP; KCNS3; KLF5; KLHDC9; LMO3; NPAS2; PAPD5; RAC1; ARGLU1; ATP10D; CHTOP; KCNA4; MACROD2; MBD5; MLIP; NNT; PLA2G3; SLC35F5; UBE2Q1; ZBTB18; ATP8B2; DYRK1B; ECHDC1; FBRS; LARGE; MACF1; MLLT3; NUMBL; ONECUT2; RAB37; RAB4B; SMG7; ACSL6; CXXC4; CYB5R4; DENND1A; E2F7; EN2; EPHA5; ETFB; GRIP1; KIT; KLF15; LBX1; MED27; MITF; NR1H4; NRXN1; PTPN5; RAPGEF5; RAVER2; SLAMF9; SLC35A4; SPHK2; SSBP3; TCF12; TMEM229B; TNC; ATP2B2; ITGA6; |
| DMBX1 | 3.497164461 | 1.44985726 | 0.014618853 | ABHD17B; EIF5A2; HAT1; STK4; ABCB7; CALM2; CCNC; ELMO1; GLS; NBEA; SKIDA1; SPRED1; TMEM135; TNPO2; TPM3; ARPC2; LMO3; MYL12A; CHTOP; SEC61A2; WAPAL; AAK1; MLLT3; PPM1N; RTN2; SLC30A3; B3GALT2; EIF4G2; GABRA1; KDM4A; MBNL2; MIA3; PTGFRN; RWDD4; SSFA2; TFAP2A; TNC; |
| DLX2 | 1.890359168 | 1.686907869 | 0.015003917 | DCBLD2; BACH2; JARID2; RARB; SNAP25; ZNF800; MSX1; PTEN; CDK13; PBX3; CACNA2D1; MYT1L; SEPT8; HMGA1; MACF1; ZC3H7B; BNIP2; ESRRG; MITF; NEUROD1; |
| NR4A2 | 8.506616257 | 1.245330593 | 0.01686501 | ABHD17B; ARHGAP44; BTBD3; GOLGA3; SMARCD2; STK4; ATP6V1A; CALM2; CLTC; EIF1AD; HNRNPK; KCNIP3; LASP1; MEOX2; NAB1; NDRG3; NR1H3; RAB11FIP1; SKIDA1; SMAP1; TMCC1; TMEM135; USP33; UST; VAMP2; VPS45; YWHAQ; ZNF800; ARPC2; PTEN; SEMA4B; ZFP91; ARL8B; CDH20; CDK13; DAZAP1; HECTD2; KLHL36; LMO3; NPAS2; VDAC1; ARGLU1; CACNA2D1; FAM120A; FAM169A; MTUS1; NUFIP2; PABPC1L; PELI3; PIP5KL1; DIRAS1; HIPK1; JDP2; LARGE; ONECUT2; PHC2; RPRD2; ACSL6; ACTN2; AHCYL1; AKT2; ANO4; ATP1B1; BCL2L13; CEND1; CHGA; DENND1A; DTX2; DUSP10; EPHA7; IDH1; MAPK10; MAPKAPK2; MARCH8; NCOA2; NRXN1; NUCKS1; PTGFRN; RANBP1; RAPGEF5; RNF4; RPGRIP1L; SIK1; SLC25A5; SSBP3; WBP1L; ZFPM2; ZMAT4; ATP2B2; SGSM2; |
| OTX2 | 3.119092628 | 1.468766954 | 0.017088946 | CTDSPL2; BDNF; ELF1; GLS; HNRNPU; MAB21L1; MYEF2; NBEA; NETO1; SEC61A1; TACR1; TMEM135; FBXO9; KCNG3; MSX1; XRCC5; CACNA2D1; HNF4G; GMPR; PHC2; PPM1N; RTN2; SLC5A2; ZC3H7B; CA7; GRM7; INSL5; KDM4A; MBD6; MITF; MTA3; NSF; SSFA2; |
| INSM1 | 4.347826087 | 1.3620095 | 0.020122537 | CDH7; CSPG5; ST6GALNAC6; CASK; FGF17; FLI1; MEOX2; MXD1; PHF6; RARB; RNF145; SKIDA1; SLC44A1; TGIF2; NEK6; ZFP91; PBX3; TSC22D4; NOL4; PIP5KL1; EPHA10; FBRS; GAB2; KAZALD1; RAB37; RAB4B; ANO4; CCNG2; DNAJB5; EFNA3; FMNL2; HTR2C; MBD6; MITF; MYBPC3; NAT8L; PPP1R14B; PROX1; RANBP1; SLC22A18; SLC6A9; SSBP3; SZRD1; WNT7A; ATP2B2; MTSS1L; |
| ZNF143 | 5.293005671 | 1.306579538 | 0.023684212 | ATXN7L3; DYNLL1; PIM1; BET1; C5orf51; COL4A3BP; DDX5; DLG4; EHMT2; GCH1; HACE1; HSP90B1; JARID2; KCNIP3; RABGAP1L; SKIDA1; SNAP25; SRSF1; SRSF9; TERF2; VASP; ZNF148; CNIH1; ZFP91; BANP; DAZAP1; DESI2; ARGLU1; CACNA2D1; LTA4H; MYT1L; PTPN12; ZNF140; INPP5K; KMT2B; RAB4B; ZCCHC3; C6orf47; CSNK1A1; DENND1A; DNAJB5; DTX2; FAM168A; GRIN2A; HOXC4; MBD6; MBNL2; MED27; PRDM1; PROX1; RPGRIP1L; RREB1; SWT1; VKORC1; ZFPM2; ZNF710; |
| HOXC10 | 1.039697543 | 1.959361044 | 0.023704538 | CDH7; FGF9; RABGAP1L; UTRN; DNAJB4; DLX1; LMO3; DCAF8; ESRRG; HOXD10; ZFPM2; |
| HOXA13 | 2.930056711 | 1.446755076 | 0.024520686 | BTBD3; CTDSPL2; MAP3K7; SMOC1; CDK14; FN1; GJA1; GLS; PFN2; RABGAP1L; RYBP; SNAP25; VPS45; FBXO9; OPRK1; PBX3; TMEM108; CACNA2D1; DES; SLC35F5; TBC1D8; APOLD1; RPRD2; SLC4A5; DENND1A; ESRRG; KRT1; NR1H4; NRXN1; PPARGC1A; TBC1D12; |
| ZNF238 | 6.616257089 | 1.263204445 | 0.024768147 | CELF2; FGF9; RFFL; ST5; STK4; BDNF; HNRNPK; HS3ST3B1; IGF1; JUND; KALRN; KCNA5; MAP4K3; NR4A2; PPP4R2; SNAI2; TAGLN2; TSPAN4; UTRN; FRMD5; MARCKS; VAV3; BANP; CDK13; EFS; KITLG; KLF5; PBX3; SCOC; ARGLU1; CACNA2D1; KCNA4; MTUS1; PPM1B; PPP1CB; PVALB; SEPT8; SLCO3A1; AAK1; ADAMTS10; GMPR; HM13; KCTD15; LDLRAP1; PCDH1; PRELP; SLC4A5; ACVR1; CDC42EP2; CXXC4; DTX2; DUS2; ERG; ESRRG; FHL3; FMNL2; L3MBTL4; LBX1; MIA3; NRXN1; PHF3; PLCB1; PRDM1; RCOR2; SPTLC1; SSBP3; TCF12; TNC; ZNF385D; ATP2B2; |
| ZNF513 | 6.616257089 | 1.263204445 | 0.024768147 | CELF2; FGF9; RFFL; ST5; STK4; BDNF; HNRNPK; HS3ST3B1; IGF1; JUND; KALRN; KCNA5; MAP4K3; NR4A2; PPP4R2; SNAI2; TAGLN2; TSPAN4; UTRN; FRMD5; MARCKS; VAV3; BANP; CDK13; EFS; KITLG; KLF5; PBX3; SCOC; ARGLU1; CACNA2D1; KCNA4; MTUS1; PPM1B; PPP1CB; PVALB; SEPT8; SLCO3A1; AAK1; ADAMTS10; GMPR; HM13; KCTD15; LDLRAP1; PCDH1; PRELP; SLC4A5; ACVR1; CDC42EP2; CXXC4; DTX2; DUS2; ERG; ESRRG; FHL3; FMNL2; L3MBTL4; LBX1; MIA3; NRXN1; PHF3; PLCB1; PRDM1; RCOR2; SPTLC1; SSBP3; TCF12; TNC; ZNF385D; ATP2B2; |
| NRF1 | 15.40642722 | 1.153683998 | 0.025008147 | ARID4B; ARMC8; CTDSPL2; DYNLL1; FLRT2; ACTB; BAG4; BLCAP; BPNT1; CASK; CDC42; CXCR4; ELOF1; EYA4; HMGN1; HSPD1; HSPE1; JUND; KCNJ2; KIF2A; LRCH1; MCTP1; MIER1; MPP5; NANP; NBEA; NDRG3; NETO1; NR4A2; PDE7A; PIGF; PPP4R2; PRKRIR; PTBP1; PTMA; RASA1; RNF111; RSBN1; SERP1; SH3GL1; SLC35B3; SMAP1; SMARCB1; SRSF1; SRSF9; STX12; THBS1; TMEM135; TMEM55B; TNKS2; TSPAN4; YRDC; ZC2HC1C; ZNF26; CBFB; CNIH1; MAP1LC3B; SRSF7; VAV3; ZFP91; BANP; CNKSR2; DDX42; DPY30; FUS; GNAI1; PAPOLA; PHF1; POLE4; SDHD; STARD4; TIMM8B; TMEM106B; TMEM108; YWHAH; ARL5A; CHL1; FKBP1A; FOPNL; FRMD3; HEXIM1; MACROD2; N4BP2L1; NONO; NTPCR; PI4K2B; PPP1CB; RAB14; SLC35G1; TFG; WAPAL; ACTR1A; C16orf45; DYRK1B; FBRS; FKBP8; IPO11; KAZALD1; PHC2; RNF5; RPRD2; ABHD5; AIDA; AKT2; ARHGEF18; BAZ1A; C3orf58; CACFD1; CCNI; CEP63; CHORDC1; CSNK1A1; CTBP1; CTSF; DFFB; DUSP1; DVL2; EIF1; ETFB; FAM98A; FITM2; FSTL1; GJC1; HIC1; HNRNPDL; HOXC4; ICMT; IMPA2; ING2; KLHL10; MBD6; MRGBP; OSER1; PHF3; PIAS4; PPP1R37; PTPN2; QKI; RAB11B; RAPGEF5; RAVER2; SAMD8; SIRT6; SLC35A4; SPTLC1; SPTY2D1; SRSF6; STK38; SUGP2; TAF12; TBC1D12; TDRD7; TFAP2A; THNSL1; TMED5; TNC; UBE2G2; UNC79; USF2; ZBTB21; ZC3H3; ZFYVE16; MEGF8; |
| NR5A1 | 8.979206049 | 1.213213717 | 0.026692959 | CTDSPL2; EIF5A2; FGF9; HCN1; NFE2L1; ADPGK; BET1; BLCAP; EIF1AD; GLS; KCNIP3; KCNJ2; NCK2; NDRG3; OTX2; RNF14; RRAGC; RSPO3; SLC10A7; VASP; YWHAQ; ZNF148; ARPC2; NEK6; PALM2-AKAP2; SEMA4B; ZFP91; ARL8B; DAZAP1; ETFA; IRF6; KLHL36; MSI2; PHF1; SCOC; SDHD; TIMM8B; ARHGEF10L; CHL1; COX4I1; DGKB; FAM169A; NDFIP1; PVALB; TBC1D8; CAD; DIRAS1; LARGE; LDLRAP1; LUZP1; MLLT3; NYNRIN; RPRD2; SLC4A5; TEAD3; ACSL6; ACTN2; ACVR1; AKT2; ATP1B1; B3GALT2; BAZ2A; BCAP31; BCL2L13; BEND6; CA7; CHGA; CHORDC1; CXXC4; DEXI; ERG; GLIS2; HOXD10; KDM4A; KIAA1671; MAFK; MAPKAPK2; MITF; NRXN3; PLA2G15; RANBP1; RCAN2; RNF4; SIPA1L2; SLC17A6; SLC5A7; SLC6A8; SLMAP; SSBP3; ST3GAL3; TWIST1; WBP1L; XPO4; ATP2B2; SGSM2; |
| SF1 | 8.979206049 | 1.213213717 | 0.026692959 | CTDSPL2; EIF5A2; FGF9; HCN1; NFE2L1; ADPGK; BET1; BLCAP; EIF1AD; GLS; KCNIP3; KCNJ2; NCK2; NDRG3; OTX2; RNF14; RRAGC; RSPO3; SLC10A7; VASP; YWHAQ; ZNF148; ARPC2; NEK6; PALM2-AKAP2; SEMA4B; ZFP91; ARL8B; DAZAP1; ETFA; IRF6; KLHL36; MSI2; PHF1; SCOC; SDHD; TIMM8B; ARHGEF10L; CHL1; COX4I1; DGKB; FAM169A; NDFIP1; PVALB; TBC1D8; CAD; DIRAS1; LARGE; LDLRAP1; LUZP1; MLLT3; NYNRIN; RPRD2; SLC4A5; TEAD3; ACSL6; ACTN2; ACVR1; AKT2; ATP1B1; B3GALT2; BAZ2A; BCAP31; BCL2L13; BEND6; CA7; CHGA; CHORDC1; CXXC4; DEXI; ERG; GLIS2; HOXD10; KDM4A; KIAA1671; MAFK; MAPKAPK2; MITF; NRXN3; PLA2G15; RANBP1; RCAN2; RNF4; SIPA1L2; SLC17A6; SLC5A7; SLC6A8; SLMAP; SSBP3; ST3GAL3; TWIST1; WBP1L; XPO4; ATP2B2; SGSM2; |
| CREB1 | 7.844990548 | 1.229925746 | 0.027696627 | BTAF1; TANC1; TSC22D2; ARG2; BACH2; BET1; C2orf69; CALM2; CDC42; CLCN3; FAM63B; FN1; FOSB; GLS; GPR137C; GPR6; HNRNPU; JUND; LRRC8A; NINJ1; NR4A2; PDE7A; PTPRS; RNF138; SH3GL1; SNAP25; SRSF1; STX12; TAGLN2; VAMP2; VASP; CD2AP; TMEM39A; BANP; CCR7; DBN1; DDX42; KLF13; MSI2; VPS37B; YWHAH; CHL1; COX4I1; ESYT2; NIPAL1; NOL4; PI4K2B; RTCB; SLC30A9; SLC35F5; RNF44; RNF5; TSFM; AHCYL1; ATP1B1; C11orf87; CCNI; CHGA; DHX40; DUS2; DUSP1; EIF2S3; GPCPD1; HLF; IFT20; INPP5A; KLF11; MAPK10; MBNL2; MITF; NRXN1; PDLIM3; PPARGC1A; RAB11B; RAPGEF5; SIK1; SPHK2; SZRD1; TNFAIP1; YTHDC1; ZBTB21; ZNF516; ZC3H4; |
| FOXC1 | 2.835538752 | 1.437311347 | 0.028750329 | CTDSPL2; SMAD2; BDNF; CDK14; ELMO1; GPD2; HSP90B1; RIT2; SNAP25; TPM3; YPEL2; IL6ST; NEK6; FUS; KITLG; PLEKHA5; HEXIM1; HUNK; MBD5; ACSL6; ACTN2; BNIP2; CXXC4; MITF; NIPBL; PRDM1; RAPGEF5; SGPL1; SHROOM2; SUZ12; |
| NR5A2 | 4.253308129 | 1.335148555 | 0.028832234 | EIF5A2; FGF9; SMARCD2; ARID2; BLCAP; EPB41L4B; FUBP1; GDF6; IGF1; KANK4; RNF111; SKIDA1; VASP; PALM2-AKAP2; FUS; MSI2; CHL1; COX4I1; DGKB; RTCB; TBC1D8; HM13; LARGE; MLLT3; NRG1; ACSL6; ACVR1; APPL2; BAZ2A; BCL2L13; CDC42EP2; DCAF8; DENND1A; ESRRG; HOXD10; ING2; LMBR1L; LURAP1L; NRXN3; PTGFRN; RCAN2; SIPA1L2; SLC17A6; ZNF385D; LAS1L; |
| ATF4 | 2.930056711 | 1.419198711 | 0.03090809 | FGF9; TSC22D2; ARG2; CDC42; FN1; GPR137C; GPR6; KCNA5; KCNJ2; KLF4; NR4A2; PDE7A; PTMA; TAGLN2; TGIF2; CD2AP; MSI2; PPCS; VPS37B; MAPK1IP1L; NOL4; AHCYL1; BNIP3L; C11orf87; HOXD10; INPP5A; KLF11; KLF15; NRXN1; YTHDC1; ZNF516; |
| NFYA | 18.80907372 | 1.127773455 | 0.031119603 | ABHD17B; AFF3; CELF2; FOXO1; H3F3B; NFE2L1; PIK3R1; SMAD2; SMOC1; ST6GALNAC6; TANC1; TXLNG; ACTB; BDNF; CALM2; CCNC; CDK14; CNN3; CTBP2; DDX5; ELMO1; FN1; FOSB; FZD7; GCH1; GLS; GPR137C; HMGN1; HOXB4; HSP90B1; JARID2; KCNIP3; KLF4; MAB21L1; MIPOL1; MMD; MPP5; MXD1; MXD4; NCK2; NET1; PAX3; PCDH17; PDE7A; PHF6; PPP1R3B; PTBP1; PTMA; RRAGC; RRBP1; SEC23B; SH3BGRL3; SH3GL1; SOX9; SP2; TGIF2; THBS1; TMCC1; TMEM55B; TNPO2; VAMP4; YWHAQ; ZNF597; CCNG1; IL6ST; KLF10; MAP3K3; MARCKS; MSX1; PALM2-AKAP2; PDIK1L; ZFP91; CD274; CNKSR2; DBN1; DLX1; IRF6; LMO3; MED21; PHF1; RBM24; SCOC; STARD4; TMEM108; VIM; COL1A1; DGKB; DMRT2; FAM120A; FOPNL; LEMD3; MACROD2; MBD5; MTUS1; MYT1L; N4BP2L1; NIPAL1; NNT; NOL4; PIP5KL1; PLA2G3; PPM1B; SCAI; SEC61A2; SEPT8; TNFSF11; ACTR1A; APOLD1; DYRK1B; FKBP8; ISCA2; MACF1; NUMBL; ONECUT2; PHC2; POLL; PPFIA3; RNF5; RPRD2; SNX20; WASF2; ACVR1; ALDH8A1; ANO4; ATP1B1; BAZ1A; BCL2L13; BNIP2; BNIP3L; CA7; CABLES2; CCNG2; CTDSP2; CTSF; CXCL12; DEXI; DMRT3; DNAJB5; DUSP10; EBAG9; EPHA7; ERG; EZH2; FAM126B; FMNL2; GPCPD1; GRIN2A; HIC1; HMGN3; HNRNPDL; HOXC4; HOXD10; HTR2C; KDM1A; KDM4A; KIT; KLF11; LMBR1L; MARK3; MBNL2; MBOAT2; MIA3; MPC1; NCOA2; NIPBL; NRXN3; NUCKS1; PDE1B; PIAS2; PIAS4; PLA2G15; PRADC1; RAB11B; RCAN2; RNF165; RNF4; SASH3; SLC1A5; SLC43A2; SLC46A3; SRSF6; SSBP3; ST3GAL3; SUZ12; SYT1; TADA2B; TCTN3; TMEM218; TMEM33; TPCN1; VASH2; VKORC1; ZCCHC2; ZFP36L2; ZFPM2; ZNF449; BCAS3; GINS4; LAS1L; |
| OTX1 | 6.899810964 | 1.241215406 | 0.031367315 | ARMC8; BTAF1; SORCS1; BDNF; ELF1; ELMO1; FBXW7; FNBP1L; GCLC; GLS; KCNJ2; MAB21L1; MIER1; NETO1; PDE7A; RABGAP1L; RASA1; RRAGC; RYBP; TMEM178A; UTRN; VAMP4; ZNF800; EVI5; FBXO9; ZFP91; KITLG; LMO3; RAP1A; RBM24; RCN2; CACNA2D1; CHL1; DCAF12; DGKB; MACROD2; MAFB; MBD5; MTUS1; NOL4; PTPN12; RNF114; SCN1A; CAD; ECHDC1; HIPK1; HMGA1; IPO11; ARHGAP42; ARHGEF18; BCL2L13; C18orf25; CA7; CD83; CTNNA3; DUSP10; DUSP4; FAM126B; INSL5; MBNL2; MBOAT2; MITF; MTA3; NREP; NRXN1; PDLIM3; PHF3; SLMAP; SRPR; SS18; SSBP3; WIF1; ATP2B2; |
| PITX1 | 6.899810964 | 1.241215406 | 0.031367315 | ARMC8; BTAF1; SORCS1; BDNF; ELF1; ELMO1; FBXW7; FNBP1L; GCLC; GLS; KCNJ2; MAB21L1; MIER1; NETO1; PDE7A; RABGAP1L; RASA1; RRAGC; RYBP; TMEM178A; UTRN; VAMP4; ZNF800; EVI5; FBXO9; ZFP91; KITLG; LMO3; RAP1A; RBM24; RCN2; CACNA2D1; CHL1; DCAF12; DGKB; MACROD2; MAFB; MBD5; MTUS1; NOL4; PTPN12; RNF114; SCN1A; CAD; ECHDC1; HIPK1; HMGA1; IPO11; ARHGAP42; ARHGEF18; BCL2L13; C18orf25; CA7; CD83; CTNNA3; DUSP10; DUSP4; FAM126B; INSL5; MBNL2; MBOAT2; MITF; MTA3; NREP; NRXN1; PDLIM3; PHF3; SLMAP; SRPR; SS18; SSBP3; WIF1; ATP2B2; |
| LHX4 | 3.497164461 | 1.361092753 | 0.034663482 | SEMA3A; BACH2; BET1; CDK14; CTTNBP2NL; FBXW7; GJA1; GLCCI1; KALRN; MAP4K3; SKIDA1; UTRN; ZBTB8A; ZC2HC1C; EVI5; XRCC5; GNAI1; LMO3; MYL12A; NPAS2; PBX3; IPO9; MBD5; MYT1L; PTPN12; APOLD1; LDLRAP1; PHC2; CTNNA3; FMNL2; KDM4A; LINGO2; MITF; MYO1D; PRDM1; RAPGEF5; KLHL14; |
| NFE2 | 4.442344045 | 1.308331777 | 0.035187263 | AFF3; FGF9; FLRT2; RFFL; ACTB; BLCAP; CASK; CDK14; FN1; KCNJ2; NETO1; NFATC2; RABGAP1L; SPRED1; TMCC1; TMSB4X; VASP; ARPC2; CPNE8; EVI5; DDX42; FTL; KITLG; NABP1; NPAS2; PLK3; SLC35F5; SLCO3A1; AAK1; MACF1; AHCYL1; ANXA7; BAZ2A; BEND6; ESRRA; GRM7; MITF; NECAB3; PIAS2; RCAN2; SCRT2; SHROOM2; SNX25; SSBP3; TNC; ZFPM2; ZNF385D; |
| TFAP2A | 2.457466919 | 1.453347928 | 0.035297663 | RFFL; CLTC; CTTNBP2NL; FLI1; NBEA; OTX2; PAX3; PICALM; PTMA; SPRED1; YWHAQ; ZNF148; VAV3; CHL1; MYT1L; PTPN12; ACTR1A; CNTFR; MACF1; SLC5A2; AJUBA; GRIN2A; PROX1; SWT1; ZNF385D; LAS1L; |
| T | 0.661625709 | 2.196501818 | 0.037819209 | BTBD3; TXLNG; GLCCI1; FUS; GAB2; HIPK1; HOXB6; |
| HOXA3 | 4.820415879 | 1.28339075 | 0.039310623 | SEMA3A; SRSF3; TANC1; ACER2; ADAM12; CCNC; CDK14; ELF1; ELMO1; ETS1; EYA4; FGF17; GLS; GPR137C; GPR6; MAP4K3; MPZL1; NAB1; OTX2; RABGAP1L; SEC23B; SEC63; SEPT2; SMIM14; TMEM178A; UTRN; VAMP4; MARCKS; VAV3; HECTD2; LMO3; NPAS2; MBD5; MTUS1; PTPN12; SCN1A; WAPAL; CAPN6; LDLRAP1; NRG1; ESRRG; FAM126B; GORASP2; HERPUD2; MBD6; MBNL2; MITF; MTA3; SYT1; ZNF710; ZC3H4; |
| ZIC1 | 4.631379962 | 1.289323806 | 0.039906232 | ARMC8; DYNLL1; FLRT2; PIM1; AKAP11; ANP32E; BDNF; BLCAP; C5orf51; CPEB1; CTBP2; DLG4; EAF1; HELZ2; HS3ST3B1; PCDH17; RYBP; SNAP25; UST; UTRN; YPEL2; KLF10; MARCKS; FUS; RAI2; COL1A1; MTUS1; NOL4; PTPN12; LYPLA2; MACF1; NRG1; NUMBL; TGFB1; DNAJB5; EFNA3; FHL3; KDELR3; NKAIN1; RCOR2; RNF38; SCRT2; SRC; SYNDIG1; TMEM218; TNC; VASH2; ATP2B2; MTSS1L; |
| TEAD1 | 3.686200378 | 1.332658859 | 0.040654507 | ATG13; BSCL2; CERS2; CTTNBP2NL; DLG4; ELMO1; FUBP1; JUND; KANK4; MTX1; PEA15; POGK; RNF145; THBS1; DNAJB4; KLF10; PALM2-AKAP2; FUS; MYL12A; ARGLU1; MLIP; LDLRAP1; MACF1; PRRT2; SLC5A2; ACSL6; ACVR1; AJUBA; DSP; FMNL2; GPCPD1; GRM7; HOXC4; NSF; PRDM1; SLC12A2; SZRD1; WNT2B; LAS1L; |
| GFI1 | 4.631379962 | 1.279981049 | 0.044375027 | DCBLD2; SEMA3A; ASPH; BDNF; CDK14; CDON; EDN1; FBXW7; FNBP1L; HNRNPK; RARB; SKIDA1; SLC44A1; TMCC1; UTRN; VPS45; YWHAQ; ZNF800; DLG5; NEK6; ARL8B; KLF5; LSM5; MTUS1; RTCB; SEC61A2; IPO11; MACF1; MLLT3; RPRD2; ATP1B1; BCL2L13; CXXC4; DNAJB5; ESRRG; GREM2; KDM5B; MYO1C; NRXN1; NRXN3; OSER1; RAPGEF5; RNF165; SNX25; SSBP3; ST18; TNC; WNT2B; ZFPM2; |
| HOXB6 | 1.323251418 | 1.641963502 | 0.045653742 | FGF9; PRRC2C; BACH2; CITED2; FOSB; MYT1L; NOL4; SLC35F5; WAPAL; LDLRAP1; PCDH1; ERG; ESRRG; RAPGEF5; |
| HOXA10 | 0.378071834 | 2.889103274 | 0.04570572 | HAT1; DNAJB4; LMO3; ESRRG; |
| DLX5 | 2.079395085 | 1.442259486 | 0.053269189 | OSTF1; ZBTB8A; MSX1; ZFP91; NPAS2; ARHGEF10L; CACNA2D1; MACROD2; NOL4; RGS7BP; KCTD15; LDLRAP1; THRA; ESRRG; HK3; LBX1; PDS5A; PRDM1; RAPGEF5; SNX25; VMA21; ZC3H4; |
| PAX5 | 1.512287335 | 1.548965339 | 0.053755414 | ANP32B; FOSB; KCNJ2; FRMD5; MACROD2; LARGE; NRG1; DNAJB5; HMGCLL1; PLCB1; RNF38; SUZ12; TPCN1; UBXN4; YTHDC1; ZFP36L2; |
| LHX8 | 2.079395085 | 1.422857762 | 0.060039033 | H3F3B; ANXA4; MAP4K3; MIER1; SKIDA1; UTRN; ZBTB8A; CCNG1; NPAS2; PBX3; IPO9; MBD5; PTPN12; PHC2; HCRT; KDM4A; MITF; PRDM1; SLC43A2; TMEM218; ZMAT4; KLHL14; |
| ELF5 | 0.378071834 | 2.626576852 | 0.06189933 | FLRT2; BLCAP; UTRN; SSBP3; |
| FOXD3 | 3.119092628 | 1.318232682 | 0.063163731 | AFF3; FGF9; SEMA3A; BLCAP; C2CD5; CLTC; FBXW7; MPZL1; SLC10A7; UST; ZNF800; MARCKS; PTEN; KLF5; CACNA2D1; MBD5; NOVA1; ZBTB18; MACF1; DENND1A; FSTL1; GRIP1; KDM1A; MBNL2; MED27; MITF; MPP1; NREP; PROX1; RAPGEF5; SSBP3; TDRD7; WNT2B; |
| NR1H4 | 0.756143667 | 1.862243929 | 0.063594256 | HCN1; BDNF; GLS; FBXO9; PBX3; CTNNA3; FHL3; IFT20; |
| TCF12 | 8.412098299 | 1.167623802 | 0.067525382 | ATXN7L3; COPS7B; FLRT2; RAPGEFL1; TANC1; ACTB; C2orf69; CASK; CTBP2; DGKZ; FAM91A1; FLI1; GLCCI1; IGF1; KANK4; KLF4; KTN1; LRRC59; METTL23; MOB3C; MTX1; PPP1R3B; RNF111; RRBP1; SLC25A22; SNAI2; SNX2; TACR1; TAGLN2; TMEM178A; TNPO2; YWHAZ; NEK6; RAP2C; DLX1; EFS; KCNS3; SCOC; SPOPL; TSC22D4; ARHGEF10L; MBD5; MLIP; NOL4; PABPC1L; PVALB; RAB14; SLC35G1; YBX3; DYNLL2; HM13; JDP2; MACF1; MLLT3; NUMBL; PLEKHA2; POLL; PPAN; PPM1N; PPP2R5B; RTN2; SLC30A3; SNX20; TEAD3; ACVR1; AKT2; BCAP31; DNAJB5; EFNA3; GLIS2; GRIP1; GRM7; HOXD10; KRT1; MARCH8; MITF; NECAB3; PPTC7; PTPN2; RCOR2; RREB1; SLC35A4; SLC5A7; ST3GAL3; TDRD7; TMEM229B; WNT2B; ATP2B2; ITGA6; |
| EHF | 5.95463138 | 1.206353305 | 0.068250855 | DCBLD2; EIF5A2; MAP3K7; TWF1; ARFIP1; CEP44; CTTNBP2NL; EAF1; EIF1AD; G6PD; GPD2; HSP90B1; HSPD1; HSPE1; MIER1; NR4A2; NXT2; PIGF; SERP1; SH3BGRL; SKIDA1; SLC10A7; SLC35B3; TAGLN2; TMEM178A; WDR1; ZNF26; ELOVL6; TOMM70A; CDK13; EMC7; NABP1; SCOC; ARGLU1; DCAF12; LEMD3; NNT; SLC30A9; TFG; TM6SF1; UBLCP1; USF1; ATG2B; HM13; LYPLA2; POLL; ADCY2; AIDA; APPL2; CDC37L1; ESRRA; ESRRG; FAM126B; GORASP2; LURAP1L; MARK3; MTF1; RCAN2; RNASE6; SLC25A5; TDRD7; TMEM33; YTHDC1; |
| PLAG1 | 0.094517958 | 14.41669738 | 0.069363404 | MBNL2; |
| SPZ1 | 0.094517958 | 14.41669738 | 0.069363404 | LMO3; |
| FOXF2 | 0.094517958 | 14.41669738 | 0.069363404 | PRDM1; |
| NR6A1 | 6.049149338 | 1.20000104 | 0.072174985 | ARMC8; FGF9; FOXO1; HCN1; SEMA3A; TANC1; CASK; CDK14; EPB41L4B; FAM107B; FUBP1; HSPD1; KALRN; KCNJ2; LASP1; NAB1; OSTF1; PDE7A; RSPO3; SEC23B; SLC29A3; TGIF2; TMSB4X; UST; NEK6; NMNAT3; PALM2-AKAP2; KLHL36; PBX3; SCOC; CHL1; FRMD3; MBD5; SEPT8; SLC35G1; ACTR1A; CAD; LARGE; LDLRAP1; MLLT3; PPM1N; ACSL6; AKT2; BEND3; BNIP2; DENND1A; DUSP1; HOXC4; KCNAB3; MAPK10; MBNL2; MITF; NIPBL; NRXN1; PPTC7; PRDM1; RAPGEF5; SGPL1; SSBP3; ST3GAL3; TMEM33; WBP1L; ZFPM2; ZMAT4; |
| PITX3 | 2.36294896 | 1.360558475 | 0.072395893 | CTDSPL2; RASSF3; STK4; CTBP2; DLG4; FN1; GPR137C; KALRN; MAB21L1; MEOX2; TPM3; CACNA2D1; AAK1; MACF1; ACSL6; AJUBA; C21orf91; CHST1; GABRA1; LONRF3; MBNL2; MITF; NSF; PRDM1; RCAN2; |
| ISX | 2.36294896 | 1.360558475 | 0.072395893 | AFF3; ARID4B; CDK14; MAB21L1; MIER1; NR4A2; PHF6; SKIDA1; ZNF800; MARCKS; NPAS2; PAPD5; IPO9; MBD5; PTPN12; SCN1A; HMGA1; HCRT; HOXC4; IDH1; MITF; PDE1B; PRDM1; TAF7L; KLHL14; |
| PLAGL1 | 0.189035917 | 4.133746326 | 0.079936658 | RAPGEFL1; FOSB; |
| ESR1 | 5.482041588 | 1.201581321 | 0.082090549 | HCN1; CTBP2; DLG4; EIF1AX; KALRN; LASP1; MAN1C1; MMD2; PHF6; SYN3; THBS1; TPM3; TTR; VAV3; XRCC5; ETFA; PBX3; PHF1; VIM; CELSR2; COL1A1; DES; ICOS; NUFIP2; PVALB; ATP8B2; DYNLL2; LARGE; SLC4A5; ACSL6; AHCYL1; AKT2; ATP1B1; B3GALT2; CTNNA3; EBAG9; EHBP1L1; FKBP4; KLHL17; MAPK10; MAPKAPK2; MBD6; MSI1; NECAB3; NEUROD1; NRXN1; OSER1; RCOR2; SCRT2; SS18; SYNDIG1; TFAP2C; TMEM229B; TPCN1; WBP1L; ITGA6; MTSS1L; SETD8; |
| ATF6 | 1.79584121 | 1.405371095 | 0.08382655 | ELF1; LRRC59; SERP1; SMIM14; SNAP25; ZBTB8A; ARMCX2; PCSK7; NOL4; KCNA6; SLC30A3; AHCYL1; BLOC1S5; DHX40; DNAJB5; KDELR3; PXN; RAPGEF5; ZNF449; |
| RBPJ | 2.079395085 | 1.350204287 | 0.093088052 | DYNLL1; NFE2L1; CXCR4; DGKH; NCK2; PDGFA; SLC45A4; SRSF9; PTEN; KCNA4; YBX3; MACF1; BAZ2A; BEND3; CEND1; ESRRG; GRIN2A; HLF; LURAP1L; PPARGC1A; TFAP2A; TMED5; |
| MNX1 | 2.646502836 | 1.298388135 | 0.093992395 | SMOC1; BDNF; BLCAP; FBXW7; FNBP1L; GLS; RABGAP1L; SKIDA1; TMEM178A; ZNF800; CDK13; LMO3; NPAS2; RAI2; SMIM15; HUNK; MBD5; LUZP1; THRA; BAZ1A; FMNL2; HCRT; PPTC7; PRDM1; SSFA2; ST3GAL3; VMA21; KLHL14; |
| ESRRA | 6.994328922 | 1.163542135 | 0.094269048 | ABHD17B; CTDSPL2; FGF9; NFE2L1; PIM1; SEMA3A; TANC1; ABHD2; CDK14; CXCL11; GJA1; PIGF; RNF14; RRAGC; RSPO3; TMCC1; TPM3; VASP; VPS45; ZNF800; ARPC2; NEK6; PALM2-AKAP2; CDK13; ETFA; KLHL36; MSI2; PBX3; PCSK7; SCOC; SDHD; STARD4; STAT4; TIMM8B; ARGLU1; CHL1; FAM120A; MYT1L; NDFIP1; PVALB; DIRAS1; EPHA10; HIPK1; LARGE; LDLRAP1; LUZP1; MLLT3; NRG1; RNF5; SLC4A5; ACSL6; ACVR1; ATP1B1; BEND6; CXXC4; DUSP10; GPR158; HOXD10; IDH1; MAPK10; MAPKAPK2; NRXN3; PLA2G15; RAPGEF5; SLC17A6; SLC5A7; SLMAP; SSBP3; SWT1; WBP1L; XPO4; ZMAT4; ATP2B2; SGSM2; |
| SOX1 | 2.36294896 | 1.306335283 | 0.10321508 | CDC42SE1; CITED2; GPR137C; MPZL1; MXD1; PDE7A; PTMA; RARB; ZNF800; MARCKS; KITLG; LMO3; PBX3; CACNA2D1; DGKB; ZBTB18; HIPK1; KMT2B; NGFR; ACVR1; ALDH1A2; BAZ1A; E2F7; LURAP1L; ITGA6; |
| RXRA | 8.31758034 | 1.139983951 | 0.104939843 | ABHD17B; CELF2; COPS7B; CTDSPL2; SMARCD2; ABCB7; ACTB; ADPGK; ANKRD34B; ASPH; BDNF; BET1; CASK; CLCN3; CNN3; EAF1; ELF1; ELMO1; GDF6; GLCCI1; IGF1; KALRN; KCNJ2; LASP1; MOB3C; MPP5; RARB; RRBP1; SH3BGRL; SKIDA1; SLC25A22; SLC35F1; TPM3; ZNF148; ZNF800; MARCKS; MSX1; SLC22A3; TOMM70A; MDK; PBX3; RAP1A; STAT4; CHL1; DMRT2; FAM120A; MBD5; NOVA1; RNF114; SLC30A9; CNTFR; GAB2; HM13; INPP5K; KAZALD1; MLLT3; NYNRIN; PPFIA3; SNX20; ANO4; BEND6; CA7; CEND1; CXXC4; DNAJB5; DUSP4; ELOVL1; EPHA7; ESRRA; ESRRG; HIC1; HOXD10; INPP5A; MBNL2; MTA3; MYO1C; NRXN3; PPARGC1A; PPTC7; RAB11B; RREB1; SSFA2; TCTN3; TPCN1; ZNF385D; ZNF516; C17orf96; SETD8; |
| MAFK | 1.228733459 | 1.465207663 | 0.106775259 | MAP3K7; SORCS1; FLI1; GJA1; PBX3; ARGLU1; CDC37L1; ESRRG; HOXC4; MBNL2; MITF; NRXN3; ST18; |
| RUNX1 | 0.472589792 | 1.951571302 | 0.110330489 | NR4A2; CTNNA3; DENND1A; MBNL2; PPARGC1A; |
| POU3F3 | 0.661625709 | 1.712608857 | 0.112544269 | FUBP1; CHTOP; SCN1A; HIPK1; GPCPD1; MITF; ZFPM2; |
| HOXD10 | 0.56710775 | 1.804714669 | 0.11291073 | HAT1; HNRNPK; SKIDA1; LMO3; PBX3; NRXN1; |
| PATZ1 | 0.472589792 | 1.900227674 | 0.120215832 | SNAP25; SRSF1; FUS; MBD5; MTF1; |
| CRX | 2.835538752 | 1.239635221 | 0.130921382 | DCBLD2; AKAP12; CTBP2; DLG4; FBXL14; FN1; HNRNPU; KALRN; MEOX2; TMSB4X; TPM3; VASP; EMC7; ARGLU1; FAM169A; MYT1L; ECHDC1; PHC2; SLC4A5; ACSL6; ESRRG; FMNL2; HLF; MBD6; MITF; NIPBL; NSF; PRDM1; RCAN2; UBXN4; |
| ELF2 | 5.860113422 | 1.146112748 | 0.142739569 | RFFL; BAG4; C9orf152; CALM2; CLTC; DLG4; E2F5; ELMO1; LASP1; METTL23; MOB3C; MPZL1; NR4A2; RNF138; TGIF2; THBS1; TMEM178A; UTRN; ZNF148; CPNE8; KLF10; NEK6; FUS; GRASP; IRF6; KLF13; PLK3; TUSC3; ICOS; PLA2G3; SEPT8; SLCO3A1; WAPAL; WNK4; HM13; HMGA1; MACF1; NRG1; APPL2; BAZ2A; DENND1A; DUSP4; EFEMP2; ELOVL1; ESRRA; ESRRG; FHL3; FURIN; HERPUD2; ING2; LURAP1L; METTL9; NIPBL; NRXN1; PDS5A; PRDM1; RANBP1; SH3TC1; SLC9A3R2; SRPR; TDRD7; TWIST1; |
| HOXB8 | 1.890359168 | 1.282067973 | 0.15168855 | MAP3K7; SEMA3A; ADAM12; CDK14; CDK4; GJA1; NBEA; OTX2; RARB; KITLG; LMO3; ARHGEF10L; DGKB; FRMD3; BAZ1A; ESRRG; GRIP1; RAPGEF5; RCAN2; TCF4; |
| EBF1 | 1.701323251 | 1.266497829 | 0.179797348 | AFF3; CDON; COL4A3BP; CTTNBP2NL; FLI1; OSBPL7; RYBP; KLF10; LMO3; FBRS; NUMBL; PPFIA3; CA7; ESRRG; GRM7; NCOA2; RAPGEF5; RNF165; |
| GSC | 2.930056711 | 1.179551426 | 0.192170749 | ARID4B; H3F3B; CHSY1; CTBP2; FN1; GLS; KALRN; KRAS; MAP4K3; PGD; SEC61A1; SLC44A1; TPM3; OPRK1; GNAI1; CACNA2D1; DGKB; FRMD3; AAK1; KAZALD1; MLLT3; PPM1N; RTN2; SLC4A5; ACSL6; ARHGAP42; INSL5; RAPGEF5; TFAP2A; TMEM33; YBX1; |
| CEBPA | 0.094517958 | 4.837496465 | 0.19400191 | FUS; |
| BACH2 | 4.631379962 | 1.12508135 | 0.211747218 | GOLGA3; PIM1; RFFL; TXLNG; ABHD2; ANP32E; ANXA2; CBL; CPEB1; CTBP2; DLG4; GLS; KLF4; MCTP1; NAB1; NET1; RAB5A; SNAI2; TAGLN2; VASP; NEK6; SRSF7; IRF6; PBX3; DGKB; DMRT2; FRMD3; HMOX2; DIRAS1; HM13; HMGA1; LDLRAP1; MACF1; ACVR1; CHST1; CTNNA3; DMRT3; DNAJB5; DTX2; ERG; HCRT; MAFK; MSI1; PAQR3; PRDM1; SHROOM2; SNX25; SPTY2D1; TNC; |
| PKNOX1 | 0.850661626 | 1.367166019 | 0.212307838 | BACH2; FAM63B; RABGAP1L; CD274; CNKSR2; NABP1; ZBTB18; MACF1; MTA3; |
| NKX1-1 | 1.228733459 | 1.284577994 | 0.213040549 | CASK; ELMO1; MAB21L1; OSTF1; SEC23B; SKIDA1; LMO3; NPAS2; NONO; LUZP1; HERPUD2; MARK3; PRDM1; |
| SMAD1 | 0.189035917 | 2.22732988 | 0.226629347 | ELF1; ZBTB18; |
| GLI1 | 1.228733459 | 1.267220005 | 0.227045815 | DYNLL1; PIM1; DLG4; NAB1; PHF6; TGIF2; DGKB; MTUS1; TGFB1; EFNA3; ESRRG; PPARGC1A; RCOR2; |
| TBX5 | 1.606805293 | 1.202039226 | 0.250040152 | BTBD3; COL4A3BP; GLCCI1; HSP90B1; PICALM; SLC29A3; NEK6; MYT1L; GAB2; NUAK2; PLEKHA2; ACTN2; DENND1A; HOXD10; LBX1; SLC6A9; UGCG; |
| STRA13 | 4.15879017 | 1.105344597 | 0.264051656 | EIF5A2; PRRC2C; ATP6V1A; AZIN1; BDNF; C5orf51; CD164; CDK4; CPEB1; CPLX2; FBXO33; HSPD1; HSPE1; MEOX2; PICALM; PTBP1; RRAGC; SNX2; TMEM55B; TNKS2; TNPO2; ATP6V1G1; ARMCX2; PPCS; APEX1; UBE2Q1; BBC3; DYRK1B; RNF44; SLC30A3; AHR; DUSP1; DVL2; HOXD10; KLF15; OSGEP; PIAS2; PIAS4; RAB9A; RWDD4; SASH3; SHROOM2; ZBTB21; ZMAT4; |
| ESR2 | 3.402646503 | 1.118823458 | 0.264104987 | CTBP2; EIF1AX; KALRN; KANK4; RARB; RRBP1; SYN3; TPM3; XRCC5; ETFA; PBX3; NUFIP2; PVALB; USF1; ATP8B2; LARGE; PPM1N; RNF44; RTN2; TEAD3; AHCYL1; ATP1B1; CEP128; EBAG9; EHBP1L1; FKBP4; MITF; NKPD1; OSER1; SYNDIG1; TFAP2C; TPCN1; WBP1L; WNT2B; ATP2B2; ITGA6; |
| PPARA | 2.741020794 | 1.130316454 | 0.272305852 | BET1; DLG4; IGF1; MMD; MOB3C; NET1; RARB; TMEM135; ZNF800; KLF10; TOMM70A; ZFP91; ETFA; MTUS1; RNF114; CNTFR; DENND1A; ELOVL1; ESRRA; ETFB; FITM2; HOXD10; MBNL2; NCOA2; NEUROD1; PPARGC1A; RREB1; SLC25A5; ETNK2; |
| ITGAL | 1.323251418 | 1.13464373 | 0.352422636 | DLG4; FLI1; CCDC6; CNKSR2; PTPN12; APOLD1; PCDH1; PPP2R5B; EPHA7; HIC1; RANBP1; RCOR2; YBX1; CYB5D1; |
| FOXJ1 | 0.283553875 | 1.399363403 | 0.365317375 | MBNL2; NRXN3; PRDM1; |
| SIX1 | 0.189035917 | 1.524332548 | 0.383542678 | BTBD3; NIPBL; |
| NKX3-1 | 0.472589792 | 1.183865824 | 0.417855587 | ATG13; HNRNPK; PALM2-AKAP2; CACNA2D1; PVALB; |
| MEIS2 | 3.59168242 | 1.039787988 | 0.425585442 | ARID4B; MYLK2; AKAP12; FBXW7; FNBP1L; MAP4K3; MSANTD2; RARB; SKIDA1; THBS1; UTRN; PALM2-AKAP2; ARMCX2; EFS; MSI2; PLK3; TSC22D4; ONECUT2; PHC2; AHR; B4GALT5; BCL2L13; CCNG2; DNAJB5; ESRRG; FMNL2; GRIP1; HNRNPDL; KRT1; LRRC10B; METTL9; PPP1R14B; RCAN2; SLC12A2; SS18; SSBP3; STK3; UNC79; |
| TP53 | 0.189035917 | 1.259346447 | 0.480622235 | NONO; DNAJB5; |
| HOXB13 | 3.497164461 | 1.014357085 | 0.489622726 | FGF9; HAT1; PIK3R1; TANC1; FBXW7; FN1; GLCCI1; KALRN; PGD; PHF6; RABGAP1L; RASA1; RIT2; RRAGC; SKIDA1; STARD7; VPS45; ZNF800; DLX1; FOXQ1; IPO11; PHC2; ACVR1; AHCYL1; BCL2L13; DENND1A; DNAJB5; ERG; ESRRG; FMNL2; GREM2; HOXD10; KDM1A; MAPK10; NRXN1; TCF4; ZFPM2; |
| GBX1 | 1.323251418 | 1.014913473 | 0.518213716 | SMOC1; MAB21L1; OSTF1; SKIDA1; YWHAQ; NPAS2; IPO9; MYT1L; ZBTB18; LUZP1; HOXC4; MITF; SIPA1L2; ST3GAL3; |
| WT1 | 0.094517958 | 0.856017893 | 0.705575923 | SSBP3; |

**Supplementary Table 2. Target genes of miRNAs.**

| miRNA Product | Target genes |
| --- | --- |
| hsa-miR-574-5p | - |
| hsa-miR-1225-5p | - |
| hsa-miR-595 | - |
| hsa-miR-486-5p | ABHD17A; ABHD17B; AFF3; ARHGAP44; ARHGAP5; ARID4B; ARMC8; ASB4; ATXN7L3; BTAF1; BTBD3; C5orf64; CDH7; CELF2; COL6A6; COPS7B; CSPG5; CTDSPL2; DCBLD2; DOCK3; DYNLL1; EIF5A2; FGF9; FLRT2; FOXO1; GOLGA3; GPR153; GPX8; H3F3B; HAT1; HCN1; MAML3; MAP3K7; MARK1; MYLK2; NFE2L1; PIK3R1; PIM1; PRRC2C; RAPGEFL1; RASSF3; RBM7; RFFL; SEMA3A; SMAD2; SMARCD2; SMOC1; SORCS1; SP5; SRSF3; ST5; ST6GALNAC6; STK4; TANC1; TOB1; TSC22D2; TWF1; TXLNG; ZNF740; |
| hsa-miR-1207-5p | - |
| hsa-miR-630 | - |
| hsa-miR-206 | ABCB7; ABHD2; ACER2; ACTB; ADAM12; ADPGK; AKAP11; AKAP12; ANKRD29; ANKRD34B; ANP32B; ANP32E; ANXA2; ANXA4; AP1S1; ARCN1; ARFIP1; ARG2; ARID2; ARPC3; ASH2L; ASPH; ATG13; ATP6V1A; AXL; AZIN1; BACH2; BAG4; BDNF; BET1; BLCAP; BPNT1; BRI3BP; BSCL2; C10orf126; C16orf47; C1GALT1; C2CD5; C2orf69; C5orf51; C7orf43; C9orf152; CAAP1; CALM2; CAPZA1; CASK; CBL; CCNC; CCSAP; CD164; CDC42; CDC42SE1; CDK14; CDK4; CDON; CEP44; CERS2; CHSY1; CITED2; CLCN3; CLTC; CNN3; COL4A3BP; CORO1C; CPEB1; CPED1; CPLX2; CTBP2; CTTNBP2NL; CXCL11; CXCR4; DCAF12L1; DDX5; DGKE; DGKH; DGKZ; DHX15; DLG4; E2F5; EAF1; EBPL; EDN1; EHMT2; EIF1AD; EIF1AX; EIF4E; EIF4E3; ELF1; ELMO1; ELOF1; EPB41L4B; ERMP1; ETS1; EYA4; FAM107B; FAM155A; FAM63B; FAM91A1; FBXL14; FBXO33; FBXW7; FGF17; FLI1; FN1; FNBP1L; FOSB; FUBP1; FZD7; G6PD; GCFC2; GCH1; GCLC; GDF6; GJA1; GLCCI1; GLS; GPD2; GPR137C; GPR6; H3F3B; H3F3C; HACE1; HCN1; HELZ2; HIAT1; HMGN1; HNRNPA3; HNRNPK; HNRNPU; HOXB4; HS3ST3B1; HSP90B1; HSPD1; HSPE1; IGF1; JARID2; JUND; KALRN; KANK4; KCNA5; KCNIP3; KCNJ2; KIF2A; KLF4; KMT2E; KRAS; KTN1; LASP1; LIN7C; LMBR1; LRCH1; LRRC59; LRRC8A; MAB21L1; MAL2; MAN1C1; MAP4K3; MATR3; MBLAC2; MCTP1; MECOM; MEOX2; METTL23; MIER1; MIPOL1; MMD; MMD2; MOB3C; MON2; MPP5; MPZL1; MSANTD2; MTSS1; MTX1; MXD1; MXD4; MYEF2; MYLK; NAB1; NANP; NAP1L5; NBEA; NCK2; NCL; NDFIP2; NDRG3; NECAB1; NET1; NETO1; NFATC2; NINJ1; NME1-NME2; NME2; NR1H3; NR4A2; NXT2; OSBPL7; OSTF1; OTX2; PAX3; PCDH17; PDCD10; PDE7A; PDGFA; PEA15; PFN2; PGD; PHAX; PHF6; PICALM; PIGF; PIRT; PLEKHA1; PLEKHO2; POGK; POLR3G; PPIB; PPP1R3B; PPP4R2; PRKRIR; PTBP1; PTMA; PTPRS; RAB11FIP1; RAB43; RAB5A; RABEPK; RABGAP1L; RAP1B; RARB; RASA1; RIT2; RNF111; RNF138; RNF14; RNF141; RNF145; RRAGC; RRBP1; RSBN1; RSBN1L; RSPO3; RYBP; SDCBP; SEC23B; SEC61A1; SEC62; SEC63; SEPT2; SERP1; SFPQ; SH3BGRL; SH3BGRL3; SH3GL1; SKIDA1; SLC10A7; SLC25A22; SLC25A30; SLC29A3; SLC35B3; SLC35F1; SLC37A3; SLC44A1; SLC45A4; SMAP1; SMARCB1; SMIM14; SNAI2; SNAP25; SNX2; SOX9; SP2; SPRED1; SRGAP2; SRI; SRSF1; SRSF3; SRSF9; STARD7; STC2; STX12; SYN3; TACR1; TAGLN2; TBC1D15; TERF2; TGIF2; THBS1; TIGD6; TIMP3; TKT; TMCC1; TMEM135; TMEM178A; TMEM243; TMEM55B; TMEM68; TMSB4X; TMX1; TNKS2; TNPO2; TPK1; TPM3; TPM4; TPPP; TRANK1; TRIM2; TSPAN4; TTR; TWF1; UBE2H; UNC119B; USP33; UST; UTRN; VAMP2; VAMP4; VASP; VPS45; WDR1; WDR48; WDR61; XPO6; YPEL2; YRDC; YWHAQ; YWHAZ; ZBTB8A; ZC2HC1C; ZNF148; ZNF26; ZNF280C; ZNF281; ZNF580; ZNF597; ZNF800; |
| hsa-miR-297 | - |
| hsa-miR-940 | - |
| hsa-miR-1207-3p | - |
| hsa-miR-325 | - |
| hsa-miR-578 | - |
| hsa-miR-1246 | - |
| hsa-miR-188-5p | AKAP2; ARPC2; ATP6V1G1; BAG5; C6orf106; C9orf72; CBFB; CCDC6; CCNG1; CCNT2; CD2AP; CNIH1; CPNE8; CTNNBIP1; DLG5; DNAJB4; EFNB2; ELOVL6; EVI5; FBXO45; FBXO9; FOXN2; FRMD5; GATAD1; IL6ST; KCNG3; KLF10; MAP1LC3B; MAP3K3; MARCKS; MSX1; NEK6; NMNAT3; NRG3; OPRK1; PALM2-AKAP2; PDIK1L; PTEN; RAP2C; RASA1; RNF170; RSPO3; SEMA4B; SLC22A3; SPRED1; SRSF7; SUMO2; TAB3; TMEM39A; TOMM70A; UBE2I; VAV3; XRCC5; ZFP91; |
| hsa-miR-450b-3p | - |
| hsa-miR-1293 | - |
| hsa-miR-483-5p | - |
| hsa-miR-1913 | - |
| hsa-miR-1825 | - |
| hsa-miR-885-5p | - |
| hsa-miR-320c | ARFIP1; ARL8B; ARMCX2; ARPP19; ASH2L; BANP; BCAP29; CCR7; CD274; CDH20; CDK13; CNKSR2; COPS2; DAZAP1; DBN1; DDX42; DESI2; DHX15; DLX1; DPY30; EFS; EIF2B1; EMC7; EOGT; ETFA; FOXQ1; FTL; FUS; GNAI1; GRASP; HECTD2; HOXA10; INSM2; IRF6; KCNS3; KITLG; KLF13; KLF5; KLHDC9; KLHL36; LMO3; MDK; MED21; MSI2; MYL12A; NAA20; NABP1; NKX2-4; NPAS2; PAPD5; PAPOLA; PBX3; PCSK7; PHF1; PLEKHA5; PLK3; POLE4; POLR1C; PPCS; PRKAG2; PYGO2; RAC1; RAD51; RAI2; RAP1A; RASA1; RBM24; RCN2; RGS9BP; SCOC; SDHD; SFTA3; SLC10A3; SLC10A7; SLC28A3; SMARCD2; SMIM15; SPOPL; STARD4; STAT4; SYNGR2; TFRC; TIMM8B; TMEM106B; TMEM108; TPD52L2; TPM3; TRIAP1; TSC22D4; TUSC3; VDAC1; VIM; VPS37B; YWHAH; |
| hsa-miR-338-3p | APEX1; ARGLU1; ARHGEF10L; ARL5A; ARPC1B; ATP10D; ATXN7L1; B4GALT7; C12orf4; C16orf87; C7orf55-LUC7L2; CACNA2D1; CELSR2; CHCHD4; CHL1; CHTOP; COL1A1; COPS4; COX4I1; DCAF12; DES; DGKB; DMRT2; ESYT2; ETS1; F10; FAF1; FAM120A; FAM169A; FGD1; FKBP1A; FOPNL; FRMD3; FRS2; GNAQ; GNG12; GPD2; HEXIM1; HMOX2; HNF4G; HUNK; ICOS; IPO9; KCNA4; KCND2; KIAA1429; LEMD3; LGALSL; LSM5; LTA4H; MACROD2; MAFB; MAPK1IP1L; MBD5; MECOM; METTL21A; MLIP; MTHFD1L; MTUS1; MYT1L; N4BP2L1; NDFIP1; NIPAL1; NNT; NOL4; NONO; NOVA1; NTPCR; NUFIP2; PABPC1L; PELI3; PI4K2B; PIP5KL1; PLA2G3; PPM1B; PPP1CB; PTEN; PTN; PTPN12; PVALB; RAB14; RAB23; RBBP5; RGS7BP; RNF114; RTCB; SCAI; SCN1A; SEC61A2; SEPT8; SLC30A9; SLC35F5; SLC35G1; SLCO3A1; SPHAR; TBC1D8; TERF2; TFG; THBS1; TIA1; TM6SF1; TNFSF11; UBE2G1; UBE2Q1; UBLCP1; USF1; VAV3; WAPAL; WNK4; YBX3; ZBTB10; ZBTB18; ZNF140; |
| hsa-miR-338-5p | - |
| hsa-miR-1275 | - |
| hsa-miR-875-3p | - |
| hsa-miR-296-5p | AAK1; ACTR1A; ADAMTS10; AMMECR1L; APOLD1; ATG2B; ATP8B2; ATXN7L3; BBC3; C16orf45; C16orf96; CACNG8; CAD; CAPN6; CCAR2; CNTFR; DIRAS1; DYNLL2; DYRK1B; ECHDC1; EPHA10; EPN1; FAM222B; FBRS; FKBP8; GAB2; GDI1; GMPR; GPC2; HIPK1; HM13; HMGA1; HOXB6; INPP5K; IPO11; ISCA2; ISY1-RAB43; JDP2; KAZALD1; KCNA6; KCTD15; KMT2B; LARGE; LDLRAP1; LEP; LRFN1; LUZP1; LYPLA2; LZIC; MACF1; MLLT3; NGFR; NRG1; NUAK2; NUMBL; NYNRIN; ONECUT2; PCDH1; PHC2; PLEKHA2; PLLP; POLL; PPAN; PPFIA3; PPM1N; PPP2R5B; PRELP; PRRT2; RAB37; RAB43; RAB4B; RIMS4; RNF44; RNF5; RPRD2; RTN2; SCNN1G; SLC30A3; SLC4A5; SLC5A2; SMG7; SNX20; SOX12; SRF; STOML1; TEAD3; TFRC; TGFB1; THRA; TMEM115; TMEM135; TMEM158; TRNP1; TSFM; USF1; VKORC1L1; WASF2; ZC3H7B; ZCCHC3; |
| hsa-miR-137 | AACS; ABHD5; ABHD6; ACSL6; ACTN2; ACVR1; ADAM23; ADCY2; AGPAT3; AHCYL1; AHR; AIDA; AJUBA; AKAP2; AKT2; ALDH1A2; ALDH8A1; ANKRD13A; ANO4; ANXA7; AP3S1; APPL2; ARHGAP42; ARHGAP44; ARHGAP5; ARHGEF18; ARID4B; ASPH; ATP1B1; ATPAF1; B3GALT2; B4GALT5; BAG4; BAZ1A; BAZ2A; BCAP31; BCL2L13; BEND3; BEND6; BLOC1S5; BNIP2; BNIP3L; C11orf87; C18orf25; C21orf91; C2orf57; C3orf58; C6orf47; C7orf31; CA7; CABLES2; CACFD1; CAPN2; CCDC153; CCNG2; CCNI; CCNY; CCZ1; CCZ1B; CD2AP; CD69; CD83; CDC37L1; CDC42; CDC42EP2; CEACAM19; CEND1; CEP128; CEP63; CHGA; CHORDC1; CHRM2; CHST1; CHST10; CHST9; CLDN11; CLDN22; CNEP1R1; COPS2; CSNK1A1; CSTF2T; CTBP1; CTDSP2; CTNNA3; CTSF; CTTNBP2NL; CUL4A; CXCL12; CXXC4; CYB5R4; CYYR1; DCAF8; DCDC2; DCP1A; DENND1A; DEXI; DFFB; DGKG; DHX40; DIRAS2; DMRT2; DMRT3; DNAJB5; DR1; DSP; DTX2; DUS2; DUSP1; DUSP10; DUSP4; DVL2; E2F6; E2F7; EBAG9; EDIL3; EFEMP2; EFNA3; EHBP1L1; EIF1; EIF2S3; EIF4G2; ELOVL1; ELOVL2; EN2; ENHO; EPHA5; EPHA7; ERG; ESRRA; ESRRG; ETFB; EZH2; FAM117B; FAM126B; FAM168A; FAM172A; FAM196B; FAM20C; FAM221B; FAM3C; FAM98A; FBXL7; FBXW7; FHL3; FITM2; FKBP1A; FKBP4; FMNL2; FNBP1L; FSTL1; FURIN; FXYD6; GABRA1; GALNT1; GCA; GJC1; GLIS2; GNAT1; GORASP2; GPCPD1; GPR137B; GPR158; GPRC5A; GPX7; GRAMD3; GREM2; GRIN2A; GRIP1; GRM7; H3F3C; HAPLN2; HCRT; HERPUD2; HIC1; HK3; HLA-DQA1; HLA-DQA2; HLF; HLTF; HMGCLL1; HMGN3; HNRNPDL; HOXC4; HOXD10; HSPE1-MOB4; HTR2C; HYPK; ICMT; IDH1; IFT20; IMPA2; ING2; INPP5A; INSL5; JDP2; KANK4; KCNAB3; KCNMB2; KDELR3; KDM1A; KDM4A; KDM5B; KIAA1671; KIT; KLF11; KLF12; KLF15; KLHL10; KLHL17; KRT1; L3MBTL4; LAPTM4B; LBX1; LEMD3; LEPROT; LINGO2; LMBR1L; LONRF3; LRRC10B; LURAP1L; LUZP2; MAFK; MAPK10; MAPKAPK2; MARCH7; MARCH8; MARK3; MBD6; MBNL2; MBOAT2; MBTPS2; MED27; METTL9; MIA3; MITF; MLC1; MOB4; MPC1; MPP1; MRGBP; MSANTD2; MSI1; MTA3; MTDH; MTF1; MXD1; MYBPC3; MYO1C; MYO1D; NAA20; NAB2; NABP1; NAT8L; NCK1; NCOA2; NECAB3; NECAP1; NETO1; NEUROD1; NEUROD4; NFATC2; NIPBL; NKAIN1; NKPD1; NOVA1; NPC1; NR1H4; NREP; NRG3; NRXN1; NRXN3; NSF; NT5DC2; NUCKS1; NXT2; OSER1; OSGEP; PALM2-AKAP2; PAQR3; PCGF5; PDCD6; PDCL; PDE1B; PDE7A; PDHB; PDLIM3; PDS5A; PEX3; PHF3; PHTF2; PIAS2; PIAS4; PIP5KL1; PLA2G15; PLCB1; PLEKHA5; PLEKHO2; PPARGC1A; PPP1CB; PPP1R14B; PPP1R37; PPP4R2; PPTC7; PRADC1; PRDM1; PRKAA1; PRKAB1; PROX1; PRR16; PRRT4; PTBP1; PTGES2; PTGFRN; PTN; PTPN2; PTPN4; PTPN5; PXN; QKI; RAB11B; RAB9A; RANBP1; RAP2C; RAPGEF5; RASIP1; RAVER2; RBM24; RCAN2; RCOR2; RGS7BP; RNASE6; RNF165; RNF38; RNF4; RPGRIP1L; RPL15; RPL28; RPL37; RPS13; RREB1; RRM2B; RWDD4; SAMD8; SAR1B; SASH3; SCRT2; SDR39U1; SERP1; SERPINA3; SGCG; SGPL1; SH3BP5; SH3TC1; SHROOM2; SIK1; SIPA1L2; SIRT6; SLAMF9; SLC12A2; SLC17A6; SLC1A5; SLC22A18; SLC25A5; SLC30A4; SLC35A4; SLC43A2; SLC46A3; SLC5A7; SLC6A8; SLC6A9; SLC9A3R2; SLMAP; SNX25; SPATA16; SPHK2; SPTLC1; SPTY2D1; SRC; SRPR; SRSF6; SS18; SSBP3; SSFA2; SSR1; ST18; ST3GAL3; STAC; STK3; STK38; SUGP2; SUZ12; SWT1; SYNDIG1; SYT1; SZRD1; TADA2B; TAF12; TAF7L; TBC1D12; TBX3; TCF12; TCF4; TCHP; TCTN3; TDRD7; TFAP2A; TFAP2C; THBS4; THNSL1; TMED5; TMEM218; TMEM229B; TMEM33; TMSB15B; TMX1; TNC; TNFAIP1; TPBGL; TPCN1; TSNAX; TTC26; TUT1; TWIST1; UBE2G1; UBE2G2; UBE2H; UBE2W; UBXN4; UGCG; UNC79; UNC93B1; USF2; VARS; VASH2; VAX1; VKORC1; VMA21; VWA5B2; WBP1L; WIF1; WNT2B; WNT7A; XPO4; YBX1; YBX3; YTHDC1; ZBTB21; ZBTB7A; ZC3H3; ZC3H6; ZCCHC2; ZFP36L2; ZFPM2; ZFYVE16; ZMAT4; ZNF385D; ZNF449; ZNF516; ZNF710; ZNF804A; ZNHIT6; |
| hsa-miR-509-3p | - |
| hsa-miR-127-3p | ATP2B2; BCAS3; C17orf96; CYB561D1; CYB5D1; ETNK2; GINS4; ISCA2; ITGA6; KIF3B; KLHL14; LAS1L; MAPK4; MEGF8; MTSS1L; PSMB5; RAB37; RIMS4; SEPT7; SETD8; SGSM2; WNT7A; ZC3H4; |
| hsa-miR-1290 | - |
| hsa-miR-568 | - |
| hsa-miR-1261 | - |
| hsa-miR-592 | - |
| hsa-miR-621 | - |
| miRNA Product | Target genes |
| hsa-miR-574-5p | - |
| hsa-miR-1225-5p | - |
| hsa-miR-595 | - |
| hsa-miR-486-5p | ABHD17A; ABHD17B; AFF3; ARHGAP44; ARHGAP5; ARID4B; ARMC8; ASB4; ATXN7L3; BTAF1; BTBD3; C5orf64; CDH7; CELF2; COL6A6; COPS7B; CSPG5; CTDSPL2; DCBLD2; DOCK3; DYNLL1; EIF5A2; FGF9; FLRT2; FOXO1; GOLGA3; GPR153; GPX8; H3F3B; HAT1; HCN1; MAML3; MAP3K7; MARK1; MYLK2; NFE2L1; PIK3R1; PIM1; PRRC2C; RAPGEFL1; RASSF3; RBM7; RFFL; SEMA3A; SMAD2; SMARCD2; SMOC1; SORCS1; SP5; SRSF3; ST5; ST6GALNAC6; STK4; TANC1; TOB1; TSC22D2; TWF1; TXLNG; ZNF740; |
| hsa-miR-1207-5p | - |
| hsa-miR-630 | - |
| hsa-miR-206 | ABCB7; ABHD2; ACER2; ACTB; ADAM12; ADPGK; AKAP11; AKAP12; ANKRD29; ANKRD34B; ANP32B; ANP32E; ANXA2; ANXA4; AP1S1; ARCN1; ARFIP1; ARG2; ARID2; ARPC3; ASH2L; ASPH; ATG13; ATP6V1A; AXL; AZIN1; BACH2; BAG4; BDNF; BET1; BLCAP; BPNT1; BRI3BP; BSCL2; C10orf126; C16orf47; C1GALT1; C2CD5; C2orf69; C5orf51; C7orf43; C9orf152; CAAP1; CALM2; CAPZA1; CASK; CBL; CCNC; CCSAP; CD164; CDC42; CDC42SE1; CDK14; CDK4; CDON; CEP44; CERS2; CHSY1; CITED2; CLCN3; CLTC; CNN3; COL4A3BP; CORO1C; CPEB1; CPED1; CPLX2; CTBP2; CTTNBP2NL; CXCL11; CXCR4; DCAF12L1; DDX5; DGKE; DGKH; DGKZ; DHX15; DLG4; E2F5; EAF1; EBPL; EDN1; EHMT2; EIF1AD; EIF1AX; EIF4E; EIF4E3; ELF1; ELMO1; ELOF1; EPB41L4B; ERMP1; ETS1; EYA4; FAM107B; FAM155A; FAM63B; FAM91A1; FBXL14; FBXO33; FBXW7; FGF17; FLI1; FN1; FNBP1L; FOSB; FUBP1; FZD7; G6PD; GCFC2; GCH1; GCLC; GDF6; GJA1; GLCCI1; GLS; GPD2; GPR137C; GPR6; H3F3B; H3F3C; HACE1; HCN1; HELZ2; HIAT1; HMGN1; HNRNPA3; HNRNPK; HNRNPU; HOXB4; HS3ST3B1; HSP90B1; HSPD1; HSPE1; IGF1; JARID2; JUND; KALRN; KANK4; KCNA5; KCNIP3; KCNJ2; KIF2A; KLF4; KMT2E; KRAS; KTN1; LASP1; LIN7C; LMBR1; LRCH1; LRRC59; LRRC8A; MAB21L1; MAL2; MAN1C1; MAP4K3; MATR3; MBLAC2; MCTP1; MECOM; MEOX2; METTL23; MIER1; MIPOL1; MMD; MMD2; MOB3C; MON2; MPP5; MPZL1; MSANTD2; MTSS1; MTX1; MXD1; MXD4; MYEF2; MYLK; NAB1; NANP; NAP1L5; NBEA; NCK2; NCL; NDFIP2; NDRG3; NECAB1; NET1; NETO1; NFATC2; NINJ1; NME1-NME2; NME2; NR1H3; NR4A2; NXT2; OSBPL7; OSTF1; OTX2; PAX3; PCDH17; PDCD10; PDE7A; PDGFA; PEA15; PFN2; PGD; PHAX; PHF6; PICALM; PIGF; PIRT; PLEKHA1; PLEKHO2; POGK; POLR3G; PPIB; PPP1R3B; PPP4R2; PRKRIR; PTBP1; PTMA; PTPRS; RAB11FIP1; RAB43; RAB5A; RABEPK; RABGAP1L; RAP1B; RARB; RASA1; RIT2; RNF111; RNF138; RNF14; RNF141; RNF145; RRAGC; RRBP1; RSBN1; RSBN1L; RSPO3; RYBP; SDCBP; SEC23B; SEC61A1; SEC62; SEC63; SEPT2; SERP1; SFPQ; SH3BGRL; SH3BGRL3; SH3GL1; SKIDA1; SLC10A7; SLC25A22; SLC25A30; SLC29A3; SLC35B3; SLC35F1; SLC37A3; SLC44A1; SLC45A4; SMAP1; SMARCB1; SMIM14; SNAI2; SNAP25; SNX2; SOX9; SP2; SPRED1; SRGAP2; SRI; SRSF1; SRSF3; SRSF9; STARD7; STC2; STX12; SYN3; TACR1; TAGLN2; TBC1D15; TERF2; TGIF2; THBS1; TIGD6; TIMP3; TKT; TMCC1; TMEM135; TMEM178A; TMEM243; TMEM55B; TMEM68; TMSB4X; TMX1; TNKS2; TNPO2; TPK1; TPM3; TPM4; TPPP; TRANK1; TRIM2; TSPAN4; TTR; TWF1; UBE2H; UNC119B; USP33; UST; UTRN; VAMP2; VAMP4; VASP; VPS45; WDR1; WDR48; WDR61; XPO6; YPEL2; YRDC; YWHAQ; YWHAZ; ZBTB8A; ZC2HC1C; ZNF148; ZNF26; ZNF280C; ZNF281; ZNF580; ZNF597; ZNF800; |
| hsa-miR-297 | - |
| hsa-miR-940 | - |
| hsa-miR-1207-3p | - |
| hsa-miR-325 | - |
| hsa-miR-578 | - |
| hsa-miR-1246 | - |
| hsa-miR-188-5p | AKAP2; ARPC2; ATP6V1G1; BAG5; C6orf106; C9orf72; CBFB; CCDC6; CCNG1; CCNT2; CD2AP; CNIH1; CPNE8; CTNNBIP1; DLG5; DNAJB4; EFNB2; ELOVL6; EVI5; FBXO45; FBXO9; FOXN2; FRMD5; GATAD1; IL6ST; KCNG3; KLF10; MAP1LC3B; MAP3K3; MARCKS; MSX1; NEK6; NMNAT3; NRG3; OPRK1; PALM2-AKAP2; PDIK1L; PTEN; RAP2C; RASA1; RNF170; RSPO3; SEMA4B; SLC22A3; SPRED1; SRSF7; SUMO2; TAB3; TMEM39A; TOMM70A; UBE2I; VAV3; XRCC5; ZFP91; |
| hsa-miR-450b-3p | - |
| hsa-miR-1293 | - |
| hsa-miR-483-5p | - |
| hsa-miR-1913 | - |
| hsa-miR-1825 | - |
| hsa-miR-885-5p | - |
| hsa-miR-320c | ARFIP1; ARL8B; ARMCX2; ARPP19; ASH2L; BANP; BCAP29; CCR7; CD274; CDH20; CDK13; CNKSR2; COPS2; DAZAP1; DBN1; DDX42; DESI2; DHX15; DLX1; DPY30; EFS; EIF2B1; EMC7; EOGT; ETFA; FOXQ1; FTL; FUS; GNAI1; GRASP; HECTD2; HOXA10; INSM2; IRF6; KCNS3; KITLG; KLF13; KLF5; KLHDC9; KLHL36; LMO3; MDK; MED21; MSI2; MYL12A; NAA20; NABP1; NKX2-4; NPAS2; PAPD5; PAPOLA; PBX3; PCSK7; PHF1; PLEKHA5; PLK3; POLE4; POLR1C; PPCS; PRKAG2; PYGO2; RAC1; RAD51; RAI2; RAP1A; RASA1; RBM24; RCN2; RGS9BP; SCOC; SDHD; SFTA3; SLC10A3; SLC10A7; SLC28A3; SMARCD2; SMIM15; SPOPL; STARD4; STAT4; SYNGR2; TFRC; TIMM8B; TMEM106B; TMEM108; TPD52L2; TPM3; TRIAP1; TSC22D4; TUSC3; VDAC1; VIM; VPS37B; YWHAH; |
| hsa-miR-338-3p | APEX1; ARGLU1; ARHGEF10L; ARL5A; ARPC1B; ATP10D; ATXN7L1; B4GALT7; C12orf4; C16orf87; C7orf55-LUC7L2; CACNA2D1; CELSR2; CHCHD4; CHL1; CHTOP; COL1A1; COPS4; COX4I1; DCAF12; DES; DGKB; DMRT2; ESYT2; ETS1; F10; FAF1; FAM120A; FAM169A; FGD1; FKBP1A; FOPNL; FRMD3; FRS2; GNAQ; GNG12; GPD2; HEXIM1; HMOX2; HNF4G; HUNK; ICOS; IPO9; KCNA4; KCND2; KIAA1429; LEMD3; LGALSL; LSM5; LTA4H; MACROD2; MAFB; MAPK1IP1L; MBD5; MECOM; METTL21A; MLIP; MTHFD1L; MTUS1; MYT1L; N4BP2L1; NDFIP1; NIPAL1; NNT; NOL4; NONO; NOVA1; NTPCR; NUFIP2; PABPC1L; PELI3; PI4K2B; PIP5KL1; PLA2G3; PPM1B; PPP1CB; PTEN; PTN; PTPN12; PVALB; RAB14; RAB23; RBBP5; RGS7BP; RNF114; RTCB; SCAI; SCN1A; SEC61A2; SEPT8; SLC30A9; SLC35F5; SLC35G1; SLCO3A1; SPHAR; TBC1D8; TERF2; TFG; THBS1; TIA1; TM6SF1; TNFSF11; UBE2G1; UBE2Q1; UBLCP1; USF1; VAV3; WAPAL; WNK4; YBX3; ZBTB10; ZBTB18; ZNF140; |
| hsa-miR-338-5p | - |
| hsa-miR-1275 | - |
| hsa-miR-875-3p | - |
| hsa-miR-296-5p | AAK1; ACTR1A; ADAMTS10; AMMECR1L; APOLD1; ATG2B; ATP8B2; ATXN7L3; BBC3; C16orf45; C16orf96; CACNG8; CAD; CAPN6; CCAR2; CNTFR; DIRAS1; DYNLL2; DYRK1B; ECHDC1; EPHA10; EPN1; FAM222B; FBRS; FKBP8; GAB2; GDI1; GMPR; GPC2; HIPK1; HM13; HMGA1; HOXB6; INPP5K; IPO11; ISCA2; ISY1-RAB43; JDP2; KAZALD1; KCNA6; KCTD15; KMT2B; LARGE; LDLRAP1; LEP; LRFN1; LUZP1; LYPLA2; LZIC; MACF1; MLLT3; NGFR; NRG1; NUAK2; NUMBL; NYNRIN; ONECUT2; PCDH1; PHC2; PLEKHA2; PLLP; POLL; PPAN; PPFIA3; PPM1N; PPP2R5B; PRELP; PRRT2; RAB37; RAB43; RAB4B; RIMS4; RNF44; RNF5; RPRD2; RTN2; SCNN1G; SLC30A3; SLC4A5; SLC5A2; SMG7; SNX20; SOX12; SRF; STOML1; TEAD3; TFRC; TGFB1; THRA; TMEM115; TMEM135; TMEM158; TRNP1; TSFM; USF1; VKORC1L1; WASF2; ZC3H7B; ZCCHC3; |
| hsa-miR-137 | AACS; ABHD5; ABHD6; ACSL6; ACTN2; ACVR1; ADAM23; ADCY2; AGPAT3; AHCYL1; AHR; AIDA; AJUBA; AKAP2; AKT2; ALDH1A2; ALDH8A1; ANKRD13A; ANO4; ANXA7; AP3S1; APPL2; ARHGAP42; ARHGAP44; ARHGAP5; ARHGEF18; ARID4B; ASPH; ATP1B1; ATPAF1; B3GALT2; B4GALT5; BAG4; BAZ1A; BAZ2A; BCAP31; BCL2L13; BEND3; BEND6; BLOC1S5; BNIP2; BNIP3L; C11orf87; C18orf25; C21orf91; C2orf57; C3orf58; C6orf47; C7orf31; CA7; CABLES2; CACFD1; CAPN2; CCDC153; CCNG2; CCNI; CCNY; CCZ1; CCZ1B; CD2AP; CD69; CD83; CDC37L1; CDC42; CDC42EP2; CEACAM19; CEND1; CEP128; CEP63; CHGA; CHORDC1; CHRM2; CHST1; CHST10; CHST9; CLDN11; CLDN22; CNEP1R1; COPS2; CSNK1A1; CSTF2T; CTBP1; CTDSP2; CTNNA3; CTSF; CTTNBP2NL; CUL4A; CXCL12; CXXC4; CYB5R4; CYYR1; DCAF8; DCDC2; DCP1A; DENND1A; DEXI; DFFB; DGKG; DHX40; DIRAS2; DMRT2; DMRT3; DNAJB5; DR1; DSP; DTX2; DUS2; DUSP1; DUSP10; DUSP4; DVL2; E2F6; E2F7; EBAG9; EDIL3; EFEMP2; EFNA3; EHBP1L1; EIF1; EIF2S3; EIF4G2; ELOVL1; ELOVL2; EN2; ENHO; EPHA5; EPHA7; ERG; ESRRA; ESRRG; ETFB; EZH2; FAM117B; FAM126B; FAM168A; FAM172A; FAM196B; FAM20C; FAM221B; FAM3C; FAM98A; FBXL7; FBXW7; FHL3; FITM2; FKBP1A; FKBP4; FMNL2; FNBP1L; FSTL1; FURIN; FXYD6; GABRA1; GALNT1; GCA; GJC1; GLIS2; GNAT1; GORASP2; GPCPD1; GPR137B; GPR158; GPRC5A; GPX7; GRAMD3; GREM2; GRIN2A; GRIP1; GRM7; H3F3C; HAPLN2; HCRT; HERPUD2; HIC1; HK3; HLA-DQA1; HLA-DQA2; HLF; HLTF; HMGCLL1; HMGN3; HNRNPDL; HOXC4; HOXD10; HSPE1-MOB4; HTR2C; HYPK; ICMT; IDH1; IFT20; IMPA2; ING2; INPP5A; INSL5; JDP2; KANK4; KCNAB3; KCNMB2; KDELR3; KDM1A; KDM4A; KDM5B; KIAA1671; KIT; KLF11; KLF12; KLF15; KLHL10; KLHL17; KRT1; L3MBTL4; LAPTM4B; LBX1; LEMD3; LEPROT; LINGO2; LMBR1L; LONRF3; LRRC10B; LURAP1L; LUZP2; MAFK; MAPK10; MAPKAPK2; MARCH7; MARCH8; MARK3; MBD6; MBNL2; MBOAT2; MBTPS2; MED27; METTL9; MIA3; MITF; MLC1; MOB4; MPC1; MPP1; MRGBP; MSANTD2; MSI1; MTA3; MTDH; MTF1; MXD1; MYBPC3; MYO1C; MYO1D; NAA20; NAB2; NABP1; NAT8L; NCK1; NCOA2; NECAB3; NECAP1; NETO1; NEUROD1; NEUROD4; NFATC2; NIPBL; NKAIN1; NKPD1; NOVA1; NPC1; NR1H4; NREP; NRG3; NRXN1; NRXN3; NSF; NT5DC2; NUCKS1; NXT2; OSER1; OSGEP; PALM2-AKAP2; PAQR3; PCGF5; PDCD6; PDCL; PDE1B; PDE7A; PDHB; PDLIM3; PDS5A; PEX3; PHF3; PHTF2; PIAS2; PIAS4; PIP5KL1; PLA2G15; PLCB1; PLEKHA5; PLEKHO2; PPARGC1A; PPP1CB; PPP1R14B; PPP1R37; PPP4R2; PPTC7; PRADC1; PRDM1; PRKAA1; PRKAB1; PROX1; PRR16; PRRT4; PTBP1; PTGES2; PTGFRN; PTN; PTPN2; PTPN4; PTPN5; PXN; QKI; RAB11B; RAB9A; RANBP1; RAP2C; RAPGEF5; RASIP1; RAVER2; RBM24; RCAN2; RCOR2; RGS7BP; RNASE6; RNF165; RNF38; RNF4; RPGRIP1L; RPL15; RPL28; RPL37; RPS13; RREB1; RRM2B; RWDD4; SAMD8; SAR1B; SASH3; SCRT2; SDR39U1; SERP1; SERPINA3; SGCG; SGPL1; SH3BP5; SH3TC1; SHROOM2; SIK1; SIPA1L2; SIRT6; SLAMF9; SLC12A2; SLC17A6; SLC1A5; SLC22A18; SLC25A5; SLC30A4; SLC35A4; SLC43A2; SLC46A3; SLC5A7; SLC6A8; SLC6A9; SLC9A3R2; SLMAP; SNX25; SPATA16; SPHK2; SPTLC1; SPTY2D1; SRC; SRPR; SRSF6; SS18; SSBP3; SSFA2; SSR1; ST18; ST3GAL3; STAC; STK3; STK38; SUGP2; SUZ12; SWT1; SYNDIG1; SYT1; SZRD1; TADA2B; TAF12; TAF7L; TBC1D12; TBX3; TCF12; TCF4; TCHP; TCTN3; TDRD7; TFAP2A; TFAP2C; THBS4; THNSL1; TMED5; TMEM218; TMEM229B; TMEM33; TMSB15B; TMX1; TNC; TNFAIP1; TPBGL; TPCN1; TSNAX; TTC26; TUT1; TWIST1; UBE2G1; UBE2G2; UBE2H; UBE2W; UBXN4; UGCG; UNC79; UNC93B1; USF2; VARS; VASH2; VAX1; VKORC1; VMA21; VWA5B2; WBP1L; WIF1; WNT2B; WNT7A; XPO4; YBX1; YBX3; YTHDC1; ZBTB21; ZBTB7A; ZC3H3; ZC3H6; ZCCHC2; ZFP36L2; ZFPM2; ZFYVE16; ZMAT4; ZNF385D; ZNF449; ZNF516; ZNF710; ZNF804A; ZNHIT6; |
| hsa-miR-509-3p | - |
| hsa-miR-127-3p | ATP2B2; BCAS3; C17orf96; CYB561D1; CYB5D1; ETNK2; GINS4; ISCA2; ITGA6; KIF3B; KLHL14; LAS1L; MAPK4; MEGF8; MTSS1L; PSMB5; RAB37; RIMS4; SEPT7; SETD8; SGSM2; WNT7A; ZC3H4; |
| hsa-miR-1290 | - |
| hsa-miR-568 | - |
| hsa-miR-1261 | - |
| hsa-miR-592 | - |
| hsa-miR-621 | - |

**Supplementary Table 3. MF of DE-miRNAs.**

| **Molecular function** | **Percentage of genes** | **p-value** | **p = 0.05 reference** |
| --- | --- | --- | --- |
| Transcription factor activity | 7.387862797 | 2.471214597 | 1.301029996 |
| Cytoskeletal anchoring activity | 0.703605981 | 0.249159945 | 1.301029996 |
| Neurotransmitter receptor activity | 0.175901495 | 0.055145254 | 1.301029996 |
| RNA binding | 3.166226913 | 0 | 1.301029996 |
| Heat shock protein activity | 0.527704485 | 0 | 1.301029996 |
| GTPase activity | 2.110817942 | 0 | 1.301029996 |
| Transcription regulator activity | 6.156552331 | 0 | 1.301029996 |
| Protein serine/threonine kinase activity | 2.638522427 | 0 | 1.301029996 |
| Protein translocase activity | 0.175901495 | 0 | 1.301029996 |
| Receptor signaling complex scaffold activity | 2.638522427 | 0 | 1.301029996 |
| Regulator of G-protein signaling activity | 0.175901495 | 0 | 1.301029996 |
| Voltage-gated ion channel activity | 1.231310466 | 0 | 1.301029996 |
| Auxiliary transport protein activity | 2.55057168 | 0 | 1.301029996 |
| Clathrin binding | 0.087950748 | 0 | 1.301029996 |
| Protein tyrosine phosphatase activity | 0.35180299 | 0 | 1.301029996 |
| Hormone activity | 0.35180299 | 0 | 1.301029996 |
| Galactosyltransferase activity | 0.439753738 | 0 | 1.301029996 |
| Lipid phosphatase activity | 0.35180299 | 0 | 1.301029996 |
| Translation regulator activity | 0.879507476 | 0 | 1.301029996 |
| Receptor binding | 1.055408971 | 0 | 1.301029996 |
| GTPase activator activity | 1.143359719 | 0 | 1.301029996 |
| Calcium ion binding | 1.407211961 | 0 | 1.301029996 |
| Nucleocytoplasmic transporter activity | 0.087950748 | 0 | 1.301029996 |
| Receptor signaling protein tyrosine kinase activity | 0.087950748 | 0 | 1.301029996 |
| Protein serine/threonine phosphatase activity | 0.439753738 | 0 | 1.301029996 |
| Lipid binding | 0.175901495 | 0 | 1.301029996 |
| Binding | 0.263852243 | 0 | 1.301029996 |
| Transporter activity | 3.693931398 | 0 | 1.301029996 |
| Transferase activity, transferring aldehyde or ketonic groups | 0.087950748 | 0 | 1.301029996 |
| Nucleotide binding | 0.087950748 | 0 | 1.301029996 |
| Storage protein | 0.087950748 | 0 | 1.301029996 |
| Cytoskeletal protein binding | 1.495162709 | 0 | 1.301029996 |
| Deacetylase activity | 0.175901495 | 0 | 1.301029996 |
| MRNA binding | 0.087950748 | 0 | 1.301029996 |
| Protein transporter activity | 0.087950748 | 0 | 1.301029996 |
| Intracellular transporter activity | 0.087950748 | 0 | 1.301029996 |
| Sulfotransferase activity | 0.439753738 | 0 | 1.301029996 |
| Growth factor activity | 0.791556728 | 0 | 1.301029996 |
| Phosphoric diester hydrolase activity | 0.263852243 | 0 | 1.301029996 |
| Neurotransmitter transporter activity | 0.087950748 | 0 | 1.301029996 |
| Peptidase activity | 0.439753738 | 0 | 1.301029996 |
| Signal transducer activity | 0.263852243 | 0 | 1.301029996 |
| Phospholipase activity | 0.35180299 | 0 | 1.301029996 |
| Isomerase activity | 0.35180299 | 0 | 1.301029996 |
| Phosphoprotein phosphatase activity | 0.087950748 | 0 | 1.301029996 |
| Receptor regulator activity | 0.087950748 | 0 | 1.301029996 |
| Protein domain specific binding | 0.087950748 | 0 | 1.301029996 |
| ATP binding | 0.087950748 | 0 | 1.301029996 |
| Sialyltransferase activity | 0.175901495 | 0 | 1.301029996 |
| Protein binding | 1.143359719 | 0 | 1.301029996 |
| Structural constituent of cytoskeleton | 0.879507476 | 0 | 1.301029996 |
| DNA-directed DNA polymerase activity | 0.175901495 | 0 | 1.301029996 |
| Metal ion binding | 0.087950748 | 0 | 1.301029996 |
| Palmitoyltransferase activity | 0.087950748 | 0 | 1.301029996 |
| Motor activity | 0.527704485 | 0 | 1.301029996 |
| Ligand-dependent nuclear receptor activity | 0.263852243 | 0 | 1.301029996 |
| Lyase activity | 0.175901495 | 0 | 1.301029996 |
| ATPase activity | 0.703605981 | 0 | 1.301029996 |
| Enzyme inhibitor activity | 0.087950748 | 0 | 1.301029996 |
| Guanyl-nucleotide exchange factor activity | 0.703605981 | 0 | 1.301029996 |
| Kinase regulator activity | 0.175901495 | 0 | 1.301029996 |
| Ion transporter activity | 0.175901495 | 0 | 1.301029996 |
| Nucleic acid binding | 0.087950748 | 0 | 1.301029996 |
| Ubiquitin binding | 0.087950748 | 0 | 1.301029996 |
| Transmembrane receptor protein tyrosine kinase activity | 0.35180299 | 0 | 1.301029996 |
| Extracellular matrix structural constituent | 0.967458223 | 0 | 1.301029996 |
| Adenylate cyclase activity | 0.087950748 | 0 | 1.301029996 |
| Ribonucleoprotein | 0.263852243 | 0 | 1.301029996 |
| DNA repair protein | 0.35180299 | 0 | 1.301029996 |
| Ubiquitin-specific protease activity | 2.110817942 | 0 | 1.301029996 |
| Nucleotidyltransferase activity | 0.087950748 | 0 | 1.301029996 |
| Methyltransferase activity | 0.35180299 | 0 | 1.301029996 |
| DNA-directed RNA polymerase activity | 0.175901495 | 0 | 1.301029996 |
| Ligase activity | 0.615655233 | 0 | 1.301029996 |
| Acyltransferase activity | 0.439753738 | 0 | 1.301029996 |
| Kinase activity | 0.087950748 | 0 | 1.301029996 |
| Inward rectifier channel | 0.087950748 | 0 | 1.301029996 |
| Receptor signaling protein serine/threonine kinase activity | 0.087950748 | 0 | 1.301029996 |
| Transmembrane receptor activity | 0.263852243 | 0 | 1.301029996 |
| Receptor signaling protein tyrosine phosphatase activity | 0.087950748 | 0 | 1.301029996 |
| Phosphatase regulator activity | 0.087950748 | 0 | 1.301029996 |
| Lipid kinase activity | 0.175901495 | 0 | 1.301029996 |
| Deoxyribonuclease activity | 0.087950748 | 0 | 1.301029996 |
| Cytokine activity | 0.527704485 | 0 | 1.301029996 |
| Protein tyrosine/serine/threonine phosphatase activity | 0.175901495 | 0 | 1.301029996 |
| MHC class I receptor activity | 0.175901495 | 0 | 1.301029996 |
| Protein-tyrosine kinase activity | 0.175901495 | 0 | 1.301029996 |
| MHC class II receptor activity | 0.175901495 | 0 | 1.301029996 |
| Deaminase activity | 0.087950748 | 0 | 1.301029996 |
| Phosphorylase activity | 0.087950748 | 0 | 1.301029996 |
| Peroxidase activity | 0.087950748 | 0 | 1.301029996 |
| Carboxy-lyase activity | 0.087950748 | 0 | 1.301029996 |
| Extracellular ligand-gated ion channel activity | 0.175901495 | 0 | 1.301029996 |
| Ion channel activity | 0.263852243 | 0 | 1.301029996 |
| Cysteine-type peptidase activity | 0.175901495 | 0 | 1.301029996 |
| Hydrolase activity | 0.879507476 | 0 | 1.301029996 |
| Protein threonine/tyrosine kinase activity | 0.087950748 | 0 | 1.301029996 |
| Aminopeptidase activity | 0.087950748 | 0 | 1.301029996 |
| Chemokine activity | 0.175901495 | 0 | 1.301029996 |
| Intracellular ligand-gated ion channel activity | 0.087950748 | 0 | 1.301029996 |
| Metallopeptidase activity | 0.35180299 | 0 | 1.301029996 |
| Cell adhesion molecule activity | 1.495162709 | 0 | 1.301029996 |
| DNA binding | 2.90237467 | 0 | 1.301029996 |
| Catalytic activity | 2.286719437 | 0 | 1.301029996 |
| Protease inhibitor activity | 0.263852243 | 0 | 1.301029996 |
| Ribonuclease activity | 0.087950748 | 0 | 1.301029996 |
| Transferase activity | 0.439753738 | 0 | 1.301029996 |
| Oxidoreductase activity | 0.439753738 | 0 | 1.301029996 |
| Structural molecule activity | 0.879507476 | 0 | 1.301029996 |
| Defense/immunity protein activity | 0.087950748 | 0 | 1.301029996 |
| Serine-type peptidase activity | 0.175901495 | 0 | 1.301029996 |
| Structural constituent of ribosome | 0.35180299 | 0 | 1.301029996 |
| Molecular function unknown | 27.96833773 | 0 | 1.301029996 |
| Receptor activity | 1.055408971 | 0 | 1.301029996 |
| Chaperone activity | 0.175901495 | 0 | 1.301029996 |
| G-protein coupled receptor activity | 1.231310466 | 0 | 1.301029996 |

**Supplementary Table 4. CC of DE-miRNAs.**

| **Cellular component** | **Percentage of genes** | **p-value** | **p = 0.05 reference** |
| --- | --- | --- | --- |
| Cytoplasm | 49.27536232 | 7.886767591 | 1.301029996 |
| Nucleus | 48.44720497 | 4.462836917 | 1.301029996 |
| Lysosome | 15.2173913 | 1.534241789 | 1.301029996 |
| Apical membrane | 0.621118012 | 0.527764861 | 1.301029996 |
| Actin cytoskeleton | 2.070393375 | 0.46927373 | 1.301029996 |
| Endosome | 3.726708075 | 0.418141851 | 1.301029996 |
| Golgi aparatus | 8.695652174 | 0.220797826 | 1.301029996 |
| MLL5-L complex | 0.414078675 | 0.069087403 | 1.301029996 |
| Centrosome | 6.52173913 | 0 | 1.301029996 |
| Cytoskeleton | 4.554865424 | 0 | 1.301029996 |
| Synaptic vesicle | 0.621118012 | 0 | 1.301029996 |
| Cytoplasmic cyclin-dependent protein kinase holoenzyme complex | 0.207039337 | 0 | 1.301029996 |
| Exosomes | 16.97722567 | 0 | 1.301029996 |
| Perinuclear region | 1.759834369 | 0 | 1.301029996 |
| Arp2/3 protein complex | 0.310559006 | 0 | 1.301029996 |
| Perinuclear region of cytoplasm | 1.552795031 | 0 | 1.301029996 |
| Cytoplasmic part | 0.310559006 | 0 | 1.301029996 |
| Lamellipodium | 0.517598344 | 0 | 1.301029996 |
| ESC/E(Z) complex | 0.310559006 | 0 | 1.301029996 |
| Stress fiber | 0.414078675 | 0 | 1.301029996 |
| Ruffle | 0.517598344 | 0 | 1.301029996 |
| Heterogeneous nuclear ribonucleoprotein complex | 0.414078675 | 0 | 1.301029996 |
| Muscle thin filament tropomyosin | 0.207039337 | 0 | 1.301029996 |
| Cytosol | 9.83436853 | 0 | 1.301029996 |
| Platelet alpha granule lumen | 0.621118012 | 0 | 1.301029996 |
| Histone methyltransferase complex | 0.310559006 | 0 | 1.301029996 |
| Integral to Golgi membrane | 0.414078675 | 0 | 1.301029996 |
| Early endosome | 0.931677019 | 0 | 1.301029996 |
| Dendritic spine | 0.207039337 | 0 | 1.301029996 |
| AP-1 adaptor complex | 0.207039337 | 0 | 1.301029996 |
| Paraspeckles | 0.207039337 | 0 | 1.301029996 |
| Golgi-associated vesicle membrane | 0.207039337 | 0 | 1.301029996 |
| CRD-mediated mRNA stability complex | 0.207039337 | 0 | 1.301029996 |
| Intracellular | 1.6563147 | 0 | 1.301029996 |
| Late endosome | 0.621118012 | 0 | 1.301029996 |
| Nucleolus | 10.14492754 | 0 | 1.301029996 |
| Coated pit | 0.310559006 | 0 | 1.301029996 |
| Cell projection | 0.621118012 | 0 | 1.301029996 |
| AP-2 adaptor complex | 0.207039337 | 0 | 1.301029996 |
| C zone | 0.103519669 | 0 | 1.301029996 |
| Apical cortex | 0.103519669 | 0 | 1.301029996 |
| Clathrin sculpted glutamate transport vesicle membrane | 0.103519669 | 0 | 1.301029996 |
| Actomyosin | 0.103519669 | 0 | 1.301029996 |
| ACF complex | 0.103519669 | 0 | 1.301029996 |
| Fascia adherens | 0.103519669 | 0 | 1.301029996 |
| Clathrin coat of coated pit | 0.103519669 | 0 | 1.301029996 |
| Cell fraction | 0.103519669 | 0 | 1.301029996 |
| AMP-activated protein kinase complex | 0.103519669 | 0 | 1.301029996 |
| VCB complex | 0.103519669 | 0 | 1.301029996 |
| Ciliary neurotrophic factor receptor complex | 0.103519669 | 0 | 1.301029996 |
| CD95 death-inducing signaling complex | 0.103519669 | 0 | 1.301029996 |
| Golgi membrane | 0.724637681 | 0 | 1.301029996 |
| Fibrinogen complex | 0.207039337 | 0 | 1.301029996 |
| Nuclear telomere cap complex | 0.207039337 | 0 | 1.301029996 |
| Basal plasma membrane | 0.207039337 | 0 | 1.301029996 |
| Eukaryotic translation initiation factor 4F complex | 0.207039337 | 0 | 1.301029996 |
| Filamentous actin | 0.207039337 | 0 | 1.301029996 |
| Membrane | 3.002070393 | 0 | 1.301029996 |
| Clathrin-coated vesicle | 0.310559006 | 0 | 1.301029996 |
| Striated myosin muscle thick filament | 0.103519669 | 0 | 1.301029996 |
| Ku70:Ku80 complex | 0.103519669 | 0 | 1.301029996 |
| Cohesin loading complex | 0.103519669 | 0 | 1.301029996 |
| Intracellular ferritin complex | 0.103519669 | 0 | 1.301029996 |
| DNA-directed RNA polymerase I complex | 0.103519669 | 0 | 1.301029996 |
| Collagen type I | 0.103519669 | 0 | 1.301029996 |
| Signal recognition particle receptor complex | 0.103519669 | 0 | 1.301029996 |
| Striated muscle thick filament | 0.103519669 | 0 | 1.301029996 |
| Nuclear telomeric heterochromatin | 0.103519669 | 0 | 1.301029996 |
| Plus-end kinesin complex | 0.103519669 | 0 | 1.301029996 |
| Activin receptor complex | 0.103519669 | 0 | 1.301029996 |
| Golgi cisterna | 0.103519669 | 0 | 1.301029996 |
| SPOTS complex | 0.103519669 | 0 | 1.301029996 |
| Myosin | 0.103519669 | 0 | 1.301029996 |
| Ribonucleoprotein complex | 0.724637681 | 0 | 1.301029996 |
| Nuclear inner membrane | 0.207039337 | 0 | 1.301029996 |
| Filopodium | 0.207039337 | 0 | 1.301029996 |
| Cell cortex | 0.310559006 | 0 | 1.301029996 |
| Integral to endoplasmic reticulum membrane | 0.414078675 | 0 | 1.301029996 |
| Transcription factor complex | 0.517598344 | 0 | 1.301029996 |
| Membrane fraction | 2.898550725 | 0 | 1.301029996 |
| Intermediate filament cytoskeleton | 0.207039337 | 0 | 1.301029996 |
| Golgi lumen | 0.207039337 | 0 | 1.301029996 |
| CUL4 RING ubiquitin ligase complex | 0.207039337 | 0 | 1.301029996 |
| Focal adhesion | 0.517598344 | 0 | 1.301029996 |
| Endocytic vesicle membrane | 0.310559006 | 0 | 1.301029996 |
| Cytoplasmic vesicle | 1.449275362 | 0 | 1.301029996 |
| SAGA complex | 0.103519669 | 0 | 1.301029996 |
| Coated vesicle | 0.103519669 | 0 | 1.301029996 |
| Oncostatin-M receptor complex | 0.103519669 | 0 | 1.301029996 |
| Chromaffin granule | 0.103519669 | 0 | 1.301029996 |
| Interleukin-6 receptor complex | 0.103519669 | 0 | 1.301029996 |
| Integral to nuclear inner membrane | 0.103519669 | 0 | 1.301029996 |
| Clathrin coat of trans-Golgi network vesicle | 0.103519669 | 0 | 1.301029996 |
| Clathrin sculpted acetylcholine transport vesicle membrane | 0.103519669 | 0 | 1.301029996 |
| ULK1-ATG13-FIP200 complex | 0.103519669 | 0 | 1.301029996 |
| Pore complex | 0.103519669 | 0 | 1.301029996 |
| Activin responsive factor complex | 0.103519669 | 0 | 1.301029996 |
| Intrinsic to membrane | 0.103519669 | 0 | 1.301029996 |
| Clathrin coat | 0.103519669 | 0 | 1.301029996 |
| Signalosome | 0.103519669 | 0 | 1.301029996 |
| Zymogen granule | 0.414078675 | 0 | 1.301029996 |
| Plasma membrane | 25.05175983 | 0 | 1.301029996 |
| Microtubule associated complex | 0.310559006 | 0 | 1.301029996 |
| SWI/SNF complex | 0.207039337 | 0 | 1.301029996 |
| Intermediate filament | 0.414078675 | 0 | 1.301029996 |
| Endoplasmic reticulum | 8.281573499 | 0 | 1.301029996 |
| Microtubule | 1.138716356 | 0 | 1.301029996 |
| Synaptonemal complex | 0.103519669 | 0 | 1.301029996 |
| Polysomal ribosome | 0.103519669 | 0 | 1.301029996 |
| Secretory granule membrane | 0.103519669 | 0 | 1.301029996 |
| Sarcoglycan complex | 0.103519669 | 0 | 1.301029996 |
| Dynactin complex | 0.103519669 | 0 | 1.301029996 |
| Spindle midzone | 0.103519669 | 0 | 1.301029996 |
| Phagocytic vesicle | 0.103519669 | 0 | 1.301029996 |
| CCAAT-binding factor complex | 0.103519669 | 0 | 1.301029996 |
| Photoreceptor inner segment | 0.103519669 | 0 | 1.301029996 |
| Vesicle membrane | 0.103519669 | 0 | 1.301029996 |
| A band | 0.103519669 | 0 | 1.301029996 |
| SOSS complex | 0.103519669 | 0 | 1.301029996 |
| Endosome membrane | 0.310559006 | 0 | 1.301029996 |
| Dendrite | 0.310559006 | 0 | 1.301029996 |
| MLL1 complex | 0.310559006 | 0 | 1.301029996 |
| N-methyl-D-aspartate selective glutamate receptor complex | 0.103519669 | 0 | 1.301029996 |
| Connexon complex | 0.103519669 | 0 | 1.301029996 |
| Lipopolysaccharide receptor complex | 0.103519669 | 0 | 1.301029996 |
| Keratin filament | 0.103519669 | 0 | 1.301029996 |
| MMXD complex | 0.103519669 | 0 | 1.301029996 |
| Cortical actin cytoskeleton | 0.103519669 | 0 | 1.301029996 |
| COPII vesicle coat | 0.103519669 | 0 | 1.301029996 |
| Serine C-palmitoyltransferase complex | 0.103519669 | 0 | 1.301029996 |
| mRNA cap binding complex | 0.103519669 | 0 | 1.301029996 |
| AP-type membrane coat adaptor complex | 0.103519669 | 0 | 1.301029996 |
| Pericentriolar material | 0.103519669 | 0 | 1.301029996 |
| Mre11 complex | 0.103519669 | 0 | 1.301029996 |
| Cilium axoneme | 0.103519669 | 0 | 1.301029996 |
| Pseudopodium | 0.103519669 | 0 | 1.301029996 |
| Photoreceptor outer segment | 0.103519669 | 0 | 1.301029996 |
| Proton-transporting two-sector ATPase complex | 0.103519669 | 0 | 1.301029996 |
| Endoplasmic reticulum membrane | 1.242236025 | 0 | 1.301029996 |
| Voltage-gated potassium channel complex | 0.414078675 | 0 | 1.301029996 |
| ER-Golgi intermediate compartment | 0.310559006 | 0 | 1.301029996 |
| Intracellular membrane-bounded organelle | 1.035196687 | 0 | 1.301029996 |
| Inclusion body | 0.103519669 | 0 | 1.301029996 |
| Secretory vesicle (None) | 0.103519669 | 0 | 1.301029996 |
| Nonhomologous end joining complex | 0.103519669 | 0 | 1.301029996 |
| Sodium:potassium-exchanging ATPase complex | 0.103519669 | 0 | 1.301029996 |
| Mitochondrial outer membrane translocase complex | 0.103519669 | 0 | 1.301029996 |
| PCAF complex | 0.103519669 | 0 | 1.301029996 |
| Smooth endoplasmic reticulum | 0.103519669 | 0 | 1.301029996 |
| Eukaryotic translation initiation factor 2B complex | 0.103519669 | 0 | 1.301029996 |
| Aggresome | 0.103519669 | 0 | 1.301029996 |
| Cell leading edge | 0.103519669 | 0 | 1.301029996 |
| DNA-directed RNA polymerase III complex | 0.103519669 | 0 | 1.301029996 |
| Cyclin-dependent protein kinase activating kinase holoenzyme complex | 0.103519669 | 0 | 1.301029996 |
| F-actin capping protein complex | 0.103519669 | 0 | 1.301029996 |
| Axin-APC-beta-catenin-GSK3B complex | 0.103519669 | 0 | 1.301029996 |
| Adherens junction | 0.103519669 | 0 | 1.301029996 |
| Trans-Golgi network transport vesicle | 0.103519669 | 0 | 1.301029996 |
| Pre-snoRNP complex | 0.103519669 | 0 | 1.301029996 |
| Mitochondrial nucleoid | 0.310559006 | 0 | 1.301029996 |
| Mitochondrial inner membrane | 0.517598344 | 0 | 1.301029996 |
| Chromatin | 0.310559006 | 0 | 1.301029996 |
| Sarcoplasmic reticulum | 0.310559006 | 0 | 1.301029996 |
| Cytoplasmic membrane-bounded vesicle | 0.207039337 | 0 | 1.301029996 |
| Synapse | 0.310559006 | 0 | 1.301029996 |
| Melanosome | 0.103519669 | 0 | 1.301029996 |
| Cortical cytoskeleton | 0.103519669 | 0 | 1.301029996 |
| Sin3 complex | 0.103519669 | 0 | 1.301029996 |
| Gap junction | 0.103519669 | 0 | 1.301029996 |
| Cul4A-RING ubiquitin ligase complex | 0.103519669 | 0 | 1.301029996 |
| Postsynaptic membrane | 0.103519669 | 0 | 1.301029996 |
| Cyclin-dependent protein kinase holoenzyme complex | 0.103519669 | 0 | 1.301029996 |
| Early endosome membrane | 0.103519669 | 0 | 1.301029996 |
| WASH complex | 0.103519669 | 0 | 1.301029996 |
| Unconventional myosin complex | 0.103519669 | 0 | 1.301029996 |
| Voltage-gated calcium channel complex | 0.207039337 | 0 | 1.301029996 |
| Basement membrane | 0.207039337 | 0 | 1.301029996 |
| Stress granule | 0.103519669 | 0 | 1.301029996 |
| Internal side of plasma membrane | 0.103519669 | 0 | 1.301029996 |
| Z disc | 0.103519669 | 0 | 1.301029996 |
| Intracellular vesicle (None) | 0.103519669 | 0 | 1.301029996 |
| Microtubule basal body | 0.103519669 | 0 | 1.301029996 |
| IkappaB kinase complex | 0.103519669 | 0 | 1.301029996 |
| Integral to membrane of membrane fraction | 0.103519669 | 0 | 1.301029996 |
| Mitochondrial respiratory chain | 0.103519669 | 0 | 1.301029996 |
| Lateral plasma membrane | 0.103519669 | 0 | 1.301029996 |
| Growth cone | 0.103519669 | 0 | 1.301029996 |
| Nuclear matrix | 0.310559006 | 0 | 1.301029996 |
| Cul3-RING ubiquitin ligase complex | 0.103519669 | 0 | 1.301029996 |
| Mitochondrial inner membrane presequence translocase complex | 0.103519669 | 0 | 1.301029996 |
| Oligosaccharyltransferase complex | 0.103519669 | 0 | 1.301029996 |
| U12-type spliceosomal complex | 0.103519669 | 0 | 1.301029996 |
| Golgi transport complex | 0.103519669 | 0 | 1.301029996 |
| Microsome | 1.138716356 | 0 | 1.301029996 |
| Spindle pole | 0.207039337 | 0 | 1.301029996 |
| Endoplasmic reticulum lumen | 0.207039337 | 0 | 1.301029996 |
| Spindle microtubule | 0.207039337 | 0 | 1.301029996 |
| Cell surface | 1.449275362 | 0 | 1.301029996 |
| Cilium | 0.103519669 | 0 | 1.301029996 |
| Platelet alpha granule | 0.103519669 | 0 | 1.301029996 |
| Nuclear envelope | 0.517598344 | 0 | 1.301029996 |
| Integral to peroxisomal membrane | 0.103519669 | 0 | 1.301029996 |
| Ruffle membrane | 0.103519669 | 0 | 1.301029996 |
| Microvillus | 0.103519669 | 0 | 1.301029996 |
| Heterotrimeric G-protein complex | 0.103519669 | 0 | 1.301029996 |
| Peroxisomal matrix | 0.103519669 | 0 | 1.301029996 |
| Extracellular matrix | 0.82815735 | 0 | 1.301029996 |
| Nuclear chromosome, telomeric region | 0.103519669 | 0 | 1.301029996 |
| NuRD complex | 0.103519669 | 0 | 1.301029996 |
| STAGA complex | 0.103519669 | 0 | 1.301029996 |
| Neuron projection | 0.103519669 | 0 | 1.301029996 |
| Nuclear body | 0.103519669 | 0 | 1.301029996 |
| Mitochondrial outer membrane | 0.207039337 | 0 | 1.301029996 |
| Nucleoplasm | 3.002070393 | 0 | 1.301029996 |
| Vesicle (None) | 0.103519669 | 0 | 1.301029996 |
| Extrinsic to membrane | 0.103519669 | 0 | 1.301029996 |
| DNA-directed RNA polymerase II, core complex | 0.103519669 | 0 | 1.301029996 |
| Peroxisomal membrane | 0.103519669 | 0 | 1.301029996 |
| Microtubule cytoskeleton | 0.310559006 | 0 | 1.301029996 |
| Transcription factor TFTC complex | 0.103519669 | 0 | 1.301029996 |
| NuA4 histone acetyltransferase complex | 0.103519669 | 0 | 1.301029996 |
| Trans-Golgi network | 0.207039337 | 0 | 1.301029996 |
| Ribosome | 0.931677019 | 0 | 1.301029996 |
| Sarcomere | 0.103519669 | 0 | 1.301029996 |
| SCF ubiquitin ligase complex | 0.103519669 | 0 | 1.301029996 |
| Secretory granule | 0.310559006 | 0 | 1.301029996 |
| Endocytic vesicle | 0.103519669 | 0 | 1.301029996 |
| Transcription factor TFIID complex | 0.103519669 | 0 | 1.301029996 |
| Nuclear chromosome | 0.103519669 | 0 | 1.301029996 |
| Chromosome, telomeric region | 0.103519669 | 0 | 1.301029996 |
| Proteinaceous extracellular matrix | 0.414078675 | 0 | 1.301029996 |
| Membrane raft | 0.207039337 | 0 | 1.301029996 |
| Cytosolic small ribosomal subunit | 0.207039337 | 0 | 1.301029996 |
| Cytosolic large ribosomal subunit | 0.207039337 | 0 | 1.301029996 |
| PML body | 0.207039337 | 0 | 1.301029996 |
| Cell junction | 0.207039337 | 0 | 1.301029996 |
| Midbody | 0.103519669 | 0 | 1.301029996 |
| Stored secretory granule | 0.103519669 | 0 | 1.301029996 |
| Cornified envelope | 0.103519669 | 0 | 1.301029996 |
| Chromosome | 0.310559006 | 0 | 1.301029996 |
| Transport vesicle | 0.103519669 | 0 | 1.301029996 |
| Histone deacetylase complex | 0.103519669 | 0 | 1.301029996 |
| Extracellular region | 2.691511387 | 0 | 1.301029996 |
| Integral to plasma membrane | 5.900621118 | 0 | 1.301029996 |
| Apical plasma membrane | 0.207039337 | 0 | 1.301029996 |
| Mitochondrial matrix | 0.414078675 | 0 | 1.301029996 |
| Ubiquitin ligase complex | 0.207039337 | 0 | 1.301029996 |
| Mediator complex | 0.103519669 | 0 | 1.301029996 |
| Actin filament | 0.103519669 | 0 | 1.301029996 |
| Caveola | 0.103519669 | 0 | 1.301029996 |
| Integral to membrane | 10.86956522 | 0 | 1.301029996 |
| Others | 0.207039337 | 0 | 1.301029996 |
| Spindle | 0.103519669 | 0 | 1.301029996 |
| Nuclear speck | 0.103519669 | 0 | 1.301029996 |
| Mitochondrion | 7.660455487 | 0 | 1.301029996 |
| Mitochondrial membrane | 0.207039337 | 0 | 1.301029996 |
| Nuclear membrane | 0.310559006 | 0 | 1.301029996 |
| Proteasome complex | 0.103519669 | 0 | 1.301029996 |
| External side of plasma membrane | 0.103519669 | 0 | 1.301029996 |
| Cell-cell junction | 0.103519669 | 0 | 1.301029996 |
| Protein complex | 0.207039337 | 0 | 1.301029996 |
| Extracellular space | 2.070393375 | 0 | 1.301029996 |
| Basolateral plasma membrane | 0.103519669 | 0 | 1.301029996 |
| Peroxisome | 0.414078675 | 0 | 1.301029996 |
| Kinetochore | 0.310559006 | 0 | 1.301029996 |
| Extracellular | 10.86956522 | 0 | 1.301029996 |
| Soluble fraction | 0.724637681 | 0 | 1.301029996 |

**Supplementary Table 10. BP of interaction mRNA of miRNA-mRNA regulatory network.**

| ID | Description | GeneRatio | p.adjust | Count |
| --- | --- | --- | --- | --- |
| GO:0042119 | neutrophil activation | 50/348 | 3.50E-19 | 50 |
| GO:0002283 | neutrophil activation involved in immune response | 48/348 | 2.74E-18 | 48 |
| GO:0043312 | neutrophil degranulation | 47/348 | 9.30E-18 | 47 |
| GO:0002446 | neutrophil mediated immunity | 47/348 | 2.27E-17 | 47 |
| GO:0042110 | T cell activation | 40/348 | 6.58E-13 | 40 |
| GO:0006968 | cellular defense response | 15/348 | 1.93E-11 | 15 |
| GO:0031349 | positive regulation of defense response | 30/348 | 3.29E-09 | 30 |
| GO:0030217 | T cell differentiation | 24/348 | 1.42E-08 | 24 |
| GO:0030595 | leukocyte chemotaxis | 23/348 | 1.42E-08 | 23 |
| GO:0030593 | neutrophil chemotaxis | 16/348 | 2.37E-08 | 16 |
| GO:0046651 | lymphocyte proliferation | 25/348 | 3.23E-08 | 25 |
| GO:0032943 | mononuclear cell proliferation | 25/348 | 3.52E-08 | 25 |
| GO:0071621 | granulocyte chemotaxis | 17/348 | 3.52E-08 | 17 |
| GO:0030098 | lymphocyte differentiation | 28/348 | 3.52E-08 | 28 |
| GO:1903131 | mononuclear cell differentiation | 30/348 | 3.63E-08 | 30 |
| GO:0070661 | leukocyte proliferation | 26/348 | 3.63E-08 | 26 |
| GO:0045088 | regulation of innate immune response | 26/348 | 4.21E-08 | 26 |
| GO:0007159 | leukocyte cell-cell adhesion | 28/348 | 4.56E-08 | 28 |
| GO:0060326 | cell chemotaxis | 25/348 | 1.02E-07 | 25 |
| GO:0006909 | phagocytosis | 28/348 | 1.02E-07 | 28 |
| GO:0019932 | second-messenger-mediated signaling | 25/348 | 1.02E-07 | 25 |
| GO:0050808 | synapse organization | 29/348 | 1.24E-07 | 29 |
| GO:1990266 | neutrophil migration | 16/348 | 1.56E-07 | 16 |
| GO:0045089 | positive regulation of innate immune response | 21/348 | 1.62E-07 | 21 |
| GO:0002429 | immune response-activating cell surface receptor signaling pathway | 31/348 | 2.28E-07 | 31 |
| GO:0002757 | immune response-activating signal transduction | 31/348 | 2.28E-07 | 31 |
| GO:0002703 | regulation of leukocyte mediated immunity | 20/348 | 2.70E-07 | 20 |
| GO:0097530 | granulocyte migration | 17/348 | 2.70E-07 | 17 |
| GO:0050863 | regulation of T cell activation | 25/348 | 2.73E-07 | 25 |
| GO:0007204 | positive regulation of cytosolic calcium ion concentration | 24/348 | 4.35E-07 | 24 |
| GO:0097529 | myeloid leukocyte migration | 20/348 | 4.94E-07 | 20 |
| GO:0002831 | regulation of response to biotic stimulus | 28/348 | 5.64E-07 | 28 |
| GO:0050670 | regulation of lymphocyte proliferation | 20/348 | 5.88E-07 | 20 |
| GO:0051480 | regulation of cytosolic calcium ion concentration | 25/348 | 6.47E-07 | 25 |
| GO:0032944 | regulation of mononuclear cell proliferation | 20/348 | 6.47E-07 | 20 |
| GO:0002833 | positive regulation of response to biotic stimulus | 21/348 | 1.47E-06 | 21 |
| GO:0070663 | regulation of leukocyte proliferation | 20/348 | 2.24E-06 | 20 |
| GO:0072503 | cellular divalent inorganic cation homeostasis | 29/348 | 2.24E-06 | 29 |
| GO:0019722 | calcium-mediated signaling | 18/348 | 2.72E-06 | 18 |
| GO:0036336 | dendritic cell migration | 8/348 | 3.36E-06 | 8 |
| GO:0022407 | regulation of cell-cell adhesion | 27/348 | 4.03E-06 | 27 |
| GO:0022409 | positive regulation of cell-cell adhesion | 21/348 | 4.03E-06 | 21 |
| GO:0002695 | negative regulation of leukocyte activation | 17/348 | 4.03E-06 | 17 |
| GO:0050867 | positive regulation of cell activation | 26/348 | 4.30E-06 | 26 |
| GO:1903037 | regulation of leukocyte cell-cell adhesion | 23/348 | 4.30E-06 | 23 |
| GO:0072507 | divalent inorganic cation homeostasis | 29/348 | 4.30E-06 | 29 |
| GO:0006874 | cellular calcium ion homeostasis | 27/348 | 4.49E-06 | 27 |
| GO:1903039 | positive regulation of leukocyte cell-cell adhesion | 19/348 | 5.62E-06 | 19 |
| GO:0050870 | positive regulation of T cell activation | 18/348 | 6.19E-06 | 18 |
| GO:0055074 | calcium ion homeostasis | 27/348 | 7.27E-06 | 27 |
| GO:0002819 | regulation of adaptive immune response | 16/348 | 7.61E-06 | 16 |
| GO:0002683 | negative regulation of immune system process | 25/348 | 9.50E-06 | 25 |
| GO:0001819 | positive regulation of cytokine production | 26/348 | 1.18E-05 | 26 |
| GO:0002407 | dendritic cell chemotaxis | 7/348 | 1.19E-05 | 7 |
| GO:0050866 | negative regulation of cell activation | 17/348 | 1.25E-05 | 17 |
| GO:0001906 | cell killing | 16/348 | 1.41E-05 | 16 |
| GO:0002221 | pattern recognition receptor signaling pathway | 17/348 | 2.11E-05 | 17 |
| GO:0050727 | regulation of inflammatory response | 23/348 | 2.17E-05 | 23 |
| GO:0002696 | positive regulation of leukocyte activation | 24/348 | 2.87E-05 | 24 |
| GO:0002237 | response to molecule of bacterial origin | 22/348 | 3.11E-05 | 22 |
| GO:0046631 | alpha-beta T cell activation | 14/348 | 4.01E-05 | 14 |
| GO:0002699 | positive regulation of immune effector process | 17/348 | 4.01E-05 | 17 |
| GO:0042098 | T cell proliferation | 16/348 | 4.01E-05 | 16 |
| GO:0032496 | response to lipopolysaccharide | 21/348 | 4.30E-05 | 21 |
| GO:0007416 | synapse assembly | 15/348 | 4.31E-05 | 15 |
| GO:0017157 | regulation of exocytosis | 16/348 | 4.33E-05 | 16 |
| GO:0034341 | response to interferon-gamma | 16/348 | 4.33E-05 | 16 |
| GO:0051251 | positive regulation of lymphocyte activation | 22/348 | 4.43E-05 | 22 |
| GO:0002449 | lymphocyte mediated immunity | 22/348 | 5.26E-05 | 22 |
| GO:0002224 | toll-like receptor signaling pathway | 14/348 | 6.58E-05 | 14 |
| GO:0002706 | regulation of lymphocyte mediated immunity | 14/348 | 6.58E-05 | 14 |
| GO:0032680 | regulation of tumor necrosis factor production | 14/348 | 7.94E-05 | 14 |
| GO:0071216 | cellular response to biotic stimulus | 17/348 | 7.94E-05 | 17 |
| GO:0002709 | regulation of T cell mediated immunity | 10/348 | 7.94E-05 | 10 |
| GO:0032640 | tumor necrosis factor production | 14/348 | 9.06E-05 | 14 |
| GO:2000106 | regulation of leukocyte apoptotic process | 10/348 | 9.80E-05 | 10 |
| GO:0031343 | positive regulation of cell killing | 9/348 | 0.000100817 | 9 |
| GO:1903555 | regulation of tumor necrosis factor superfamily cytokine production | 14/348 | 0.000100817 | 14 |
| GO:0071706 | tumor necrosis factor superfamily cytokine production | 14/348 | 0.000123366 | 14 |
| GO:0042116 | macrophage activation | 11/348 | 0.000125396 | 11 |
| GO:0071887 | leukocyte apoptotic process | 11/348 | 0.000125396 | 11 |
| GO:0002755 | MyD88-dependent toll-like receptor signaling pathway | 7/348 | 0.000127461 | 7 |
| GO:1903706 | regulation of hemopoiesis | 23/348 | 0.000130906 | 23 |
| GO:0032637 | interleukin-8 production | 11/348 | 0.000133173 | 11 |
| GO:0002456 | T cell mediated immunity | 11/348 | 0.000144779 | 11 |
| GO:0007272 | ensheathment of neurons | 12/348 | 0.00015497 | 12 |
| GO:0008366 | axon ensheathment | 12/348 | 0.00015497 | 12 |
| GO:0050890 | cognition | 18/348 | 0.000162973 | 18 |
| GO:0032760 | positive regulation of tumor necrosis factor production | 10/348 | 0.000180657 | 10 |
| GO:0002886 | regulation of myeloid leukocyte mediated immunity | 8/348 | 0.000180657 | 8 |
| GO:1902105 | regulation of leukocyte differentiation | 18/348 | 0.000183244 | 18 |
| GO:0099560 | synaptic membrane adhesion | 6/348 | 0.000191353 | 6 |
| GO:0071346 | cellular response to interferon-gamma | 14/348 | 0.000207049 | 14 |
| GO:0018108 | peptidyl-tyrosine phosphorylation | 21/348 | 0.000207877 | 21 |
| GO:1902107 | positive regulation of leukocyte differentiation | 13/348 | 0.000213488 | 13 |
| GO:1903708 | positive regulation of hemopoiesis | 13/348 | 0.000213488 | 13 |
| GO:0070227 | lymphocyte apoptotic process | 9/348 | 0.000219146 | 9 |
| GO:0070228 | regulation of lymphocyte apoptotic process | 8/348 | 0.000219146 | 8 |
| GO:1903557 | positive regulation of tumor necrosis factor superfamily cytokine production | 10/348 | 0.000219146 | 10 |
| GO:0002822 | regulation of adaptive immune response based on somatic recombination of immune receptors built from immunoglobulin superfamily domains | 13/348 | 0.000219146 | 13 |
| GO:0018212 | peptidyl-tyrosine modification | 21/348 | 0.000219146 | 21 |
| GO:0002697 | regulation of immune effector process | 24/348 | 0.000219164 | 24 |
| GO:0042742 | defense response to bacterium | 20/348 | 0.000231701 | 20 |
| GO:0001909 | leukocyte mediated cytotoxicity | 11/348 | 0.000266128 | 11 |
| GO:0050764 | regulation of phagocytosis | 10/348 | 0.000309876 | 10 |
| GO:0032757 | positive regulation of interleukin-8 production | 8/348 | 0.000310862 | 8 |
| GO:0032677 | regulation of interleukin-8 production | 10/348 | 0.000395425 | 10 |
| GO:1990868 | response to chemokine | 10/348 | 0.000395425 | 10 |
| GO:1990869 | cellular response to chemokine | 10/348 | 0.000395425 | 10 |
| GO:0043300 | regulation of leukocyte degranulation | 7/348 | 0.000401129 | 7 |
| GO:0002758 | innate immune response-activating signal transduction | 11/348 | 0.000409853 | 11 |
| GO:0031341 | regulation of cell killing | 10/348 | 0.000421643 | 10 |
| GO:0045576 | mast cell activation | 8/348 | 0.000430339 | 8 |
| GO:0045785 | positive regulation of cell adhesion | 22/348 | 0.000457712 | 22 |
| GO:0042129 | regulation of T cell proliferation | 13/348 | 0.000458295 | 13 |
| GO:0099003 | vesicle-mediated transport in synapse | 14/348 | 0.000474244 | 14 |
| GO:0019835 | cytolysis | 6/348 | 0.000569762 | 6 |
| GO:0042552 | myelination | 11/348 | 0.000569824 | 11 |
| GO:0046629 | gamma-delta T cell activation | 5/348 | 0.000580588 | 5 |
| GO:0002218 | activation of innate immune response | 12/348 | 0.000581201 | 12 |
| GO:0070665 | positive regulation of leukocyte proliferation | 12/348 | 0.000581201 | 12 |
| GO:0050777 | negative regulation of immune response | 12/348 | 0.000656473 | 12 |
| GO:0001774 | microglial cell activation | 7/348 | 0.000656473 | 7 |
| GO:0045619 | regulation of lymphocyte differentiation | 13/348 | 0.000656473 | 13 |
| GO:0099504 | synaptic vesicle cycle | 13/348 | 0.000734272 | 13 |
| GO:0051250 | negative regulation of lymphocyte activation | 12/348 | 0.000777829 | 12 |
| GO:0006959 | humoral immune response | 20/348 | 0.000795715 | 20 |
| GO:0015800 | acidic amino acid transport | 8/348 | 0.000865808 | 8 |
| GO:0033630 | positive regulation of cell adhesion mediated by integrin | 5/348 | 0.000909233 | 5 |
| GO:0032621 | interleukin-18 production | 4/348 | 0.000923483 | 4 |
| GO:0099172 | presynapse organization | 7/348 | 0.000934663 | 7 |
| GO:0002532 | production of molecular mediator involved in inflammatory response | 9/348 | 0.000941264 | 9 |
| GO:0070098 | chemokine-mediated signaling pathway | 9/348 | 0.000941264 | 9 |
| GO:0071219 | cellular response to molecule of bacterial origin | 14/348 | 0.000941264 | 14 |
| GO:0001508 | action potential | 11/348 | 0.001091259 | 11 |
| GO:0050671 | positive regulation of lymphocyte proliferation | 11/348 | 0.001091259 | 11 |
| GO:0086012 | membrane depolarization during cardiac muscle cell action potential | 5/348 | 0.001091259 | 5 |
| GO:0032946 | positive regulation of mononuclear cell proliferation | 11/348 | 0.001144578 | 11 |
| GO:1903305 | regulation of regulated secretory pathway | 11/348 | 0.001144578 | 11 |
| GO:0045730 | respiratory burst | 6/348 | 0.001146189 | 6 |
| GO:0086010 | membrane depolarization during action potential | 6/348 | 0.001146189 | 6 |
| GO:0050803 | regulation of synapse structure or activity | 14/348 | 0.001146189 | 14 |
| GO:0060333 | interferon-gamma-mediated signaling pathway | 9/348 | 0.001146189 | 9 |
| GO:0046633 | alpha-beta T cell proliferation | 6/348 | 0.001331567 | 6 |
| GO:0048709 | oligodendrocyte differentiation | 9/348 | 0.001343163 | 9 |
| GO:0002460 | adaptive immune response based on somatic recombination of immune receptors built from immunoglobulin superfamily domains | 19/348 | 0.001366232 | 19 |
| GO:0001912 | positive regulation of leukocyte mediated cytotoxicity | 7/348 | 0.001563758 | 7 |
| GO:0019730 | antimicrobial humoral response | 11/348 | 0.001593149 | 11 |
| GO:0002220 | innate immune response activating cell surface receptor signaling pathway | 10/348 | 0.001593149 | 10 |
| GO:0042100 | B cell proliferation | 9/348 | 0.001656535 | 9 |
| GO:1902476 | chloride transmembrane transport | 9/348 | 0.001656535 | 9 |
| GO:0042063 | gliogenesis | 16/348 | 0.001818396 | 16 |
| GO:0071222 | cellular response to lipopolysaccharide | 13/348 | 0.001818396 | 13 |
| GO:0006816 | calcium ion transport | 20/348 | 0.001825666 | 20 |
| GO:0050830 | defense response to Gram-positive bacterium | 9/348 | 0.001896589 | 9 |
| GO:0014047 | glutamate secretion | 6/348 | 0.001928704 | 6 |
| GO:0045580 | regulation of T cell differentiation | 11/348 | 0.001928704 | 11 |
| GO:0034121 | regulation of toll-like receptor signaling pathway | 8/348 | 0.001928704 | 8 |
| GO:0140029 | exocytic process | 8/348 | 0.002101795 | 8 |
| GO:0045059 | positive thymic T cell selection | 4/348 | 0.002164856 | 4 |
| GO:0033003 | regulation of mast cell activation | 6/348 | 0.002164856 | 6 |
| GO:0001910 | regulation of leukocyte mediated cytotoxicity | 8/348 | 0.002258974 | 8 |
| GO:0045621 | positive regulation of lymphocyte differentiation | 9/348 | 0.002280417 | 9 |
| GO:0002705 | positive regulation of leukocyte mediated immunity | 10/348 | 0.002351415 | 10 |
| GO:0050807 | regulation of synapse organization | 13/348 | 0.002392428 | 13 |
| GO:0050851 | antigen receptor-mediated signaling pathway | 17/348 | 0.002392428 | 17 |
| GO:0010518 | positive regulation of phospholipase activity | 7/348 | 0.002392428 | 7 |
| GO:0032615 | interleukin-12 production | 7/348 | 0.002392428 | 7 |
| GO:0032655 | regulation of interleukin-12 production | 7/348 | 0.002392428 | 7 |
| GO:0032612 | interleukin-1 production | 10/348 | 0.002424334 | 10 |
| GO:0007214 | gamma-aminobutyric acid signaling pathway | 5/348 | 0.002477069 | 5 |
| GO:0019882 | antigen processing and presentation | 14/348 | 0.002477069 | 14 |
| GO:0007611 | learning or memory | 14/348 | 0.002569099 | 14 |
| GO:0042113 | B cell activation | 17/348 | 0.002569099 | 17 |
| GO:0030888 | regulation of B cell proliferation | 7/348 | 0.002569099 | 7 |
| GO:0031348 | negative regulation of defense response | 14/348 | 0.002646103 | 14 |
| GO:0002468 | dendritic cell antigen processing and presentation | 4/348 | 0.002646103 | 4 |
| GO:0034329 | cell junction assembly | 20/348 | 0.002652716 | 20 |
| GO:0051924 | regulation of calcium ion transport | 14/348 | 0.002844118 | 14 |
| GO:0010001 | glial cell differentiation | 13/348 | 0.002938214 | 13 |
| GO:0042391 | regulation of membrane potential | 20/348 | 0.003144167 | 20 |
| GO:0043304 | regulation of mast cell degranulation | 5/348 | 0.00333166 | 5 |
| GO:1904862 | inhibitory synapse assembly | 4/348 | 0.003362287 | 4 |
| GO:0002708 | positive regulation of lymphocyte mediated immunity | 9/348 | 0.003362287 | 9 |
| GO:0032611 | interleukin-1 beta production | 9/348 | 0.003362287 | 9 |
| GO:0098742 | cell-cell adhesion via plasma-membrane adhesion molecules | 15/348 | 0.00338987 | 15 |
| GO:0006821 | chloride transport | 9/348 | 0.003545552 | 9 |
| GO:0043303 | mast cell degranulation | 6/348 | 0.003545552 | 6 |
| GO:2000107 | negative regulation of leukocyte apoptotic process | 6/348 | 0.003545552 | 6 |
| GO:0032729 | positive regulation of interferon-gamma production | 7/348 | 0.003545552 | 7 |
| GO:0033006 | regulation of mast cell activation involved in immune response | 5/348 | 0.003750134 | 5 |
| GO:0050804 | modulation of chemical synaptic transmission | 19/348 | 0.003750134 | 19 |
| GO:0046635 | positive regulation of alpha-beta T cell activation | 7/348 | 0.003805862 | 7 |
| GO:0099177 | regulation of trans-synaptic signaling | 19/348 | 0.003805862 | 19 |
| GO:0010959 | regulation of metal ion transport | 14/348 | 0.003805862 | 14 |
| GO:0002279 | mast cell activation involved in immune response | 6/348 | 0.003805862 | 6 |
| GO:0002448 | mast cell mediated immunity | 6/348 | 0.003805862 | 6 |
| GO:0002823 | negative regulation of adaptive immune response based on somatic recombination of immune receptors built from immunoglobulin superfamily domains | 6/348 | 0.003805862 | 6 |
| GO:0099054 | presynapse assembly | 6/348 | 0.003805862 | 6 |
| GO:0023061 | signal release | 21/348 | 0.003805862 | 21 |
| GO:0002286 | T cell activation involved in immune response | 9/348 | 0.003809595 | 9 |
| GO:0045582 | positive regulation of T cell differentiation | 8/348 | 0.003865276 | 8 |
| GO:0071674 | mononuclear cell migration | 12/348 | 0.003865276 | 12 |
| GO:0016045 | detection of bacterium | 4/348 | 0.003868903 | 4 |
| GO:0034134 | toll-like receptor 2 signaling pathway | 4/348 | 0.003868903 | 4 |
| GO:0001913 | T cell mediated cytotoxicity | 6/348 | 0.004103408 | 6 |
| GO:0033628 | regulation of cell adhesion mediated by integrin | 6/348 | 0.004103408 | 6 |
| GO:0045058 | T cell selection | 6/348 | 0.004103408 | 6 |
| GO:0050731 | positive regulation of peptidyl-tyrosine phosphorylation | 12/348 | 0.00431737 | 12 |
| GO:0050864 | regulation of B cell activation | 12/348 | 0.00431737 | 12 |
| GO:0014068 | positive regulation of phosphatidylinositol 3-kinase signaling | 8/348 | 0.004324556 | 8 |
| GO:0070231 | T cell apoptotic process | 6/348 | 0.004517341 | 6 |
| GO:0038094 | Fc-gamma receptor signaling pathway | 10/348 | 0.004591773 | 10 |
| GO:0007202 | activation of phospholipase C activity | 5/348 | 0.004591773 | 5 |
| GO:0002523 | leukocyte migration involved in inflammatory response | 4/348 | 0.004652613 | 4 |
| GO:0070269 | pyroptosis | 4/348 | 0.004652613 | 4 |
| GO:0030001 | metal ion transport | 21/348 | 0.004770188 | 21 |
| GO:0006801 | superoxide metabolic process | 7/348 | 0.004896111 | 7 |
| GO:0010517 | regulation of phospholipase activity | 7/348 | 0.004896111 | 7 |
| GO:0086019 | cell-cell signaling involved in cardiac conduction | 5/348 | 0.005185238 | 5 |
| GO:1903307 | positive regulation of regulated secretory pathway | 6/348 | 0.00541096 | 6 |
| GO:0051259 | protein complex oligomerization | 13/348 | 0.005550312 | 13 |
| GO:0002544 | chronic inflammatory response | 4/348 | 0.005600584 | 4 |
| GO:0098543 | detection of other organism | 4/348 | 0.005600584 | 4 |
| GO:0002688 | regulation of leukocyte chemotaxis | 9/348 | 0.005703316 | 9 |
| GO:0032652 | regulation of interleukin-1 production | 9/348 | 0.005703316 | 9 |
| GO:0002431 | Fc receptor mediated stimulatory signaling pathway | 10/348 | 0.005710439 | 10 |
| GO:0046640 | regulation of alpha-beta T cell proliferation | 5/348 | 0.005745353 | 5 |
| GO:0070232 | regulation of T cell apoptotic process | 5/348 | 0.005745353 | 5 |
| GO:0032602 | chemokine production | 8/348 | 0.005745353 | 8 |
| GO:0002820 | negative regulation of adaptive immune response | 6/348 | 0.005762222 | 6 |
| GO:0050730 | regulation of peptidyl-tyrosine phosphorylation | 14/348 | 0.005880044 | 14 |
| GO:0098661 | inorganic anion transmembrane transport | 9/348 | 0.005885271 | 9 |
| GO:0033077 | T cell differentiation in thymus | 7/348 | 0.00591609 | 7 |
| GO:0060193 | positive regulation of lipase activity | 7/348 | 0.00591609 | 7 |
| GO:0002704 | negative regulation of leukocyte mediated immunity | 6/348 | 0.006236254 | 6 |
| GO:0009620 | response to fungus | 6/348 | 0.006236254 | 6 |
| GO:0050852 | T cell receptor signaling pathway | 12/348 | 0.006308574 | 12 |
| GO:0071260 | cellular response to mechanical stimulus | 7/348 | 0.006324734 | 7 |
| GO:0070233 | negative regulation of T cell apoptotic process | 4/348 | 0.006439503 | 4 |
| GO:0022898 | regulation of transmembrane transporter activity | 14/348 | 0.007097473 | 14 |
| GO:0006691 | leukotriene metabolic process | 5/348 | 0.007126804 | 5 |
| GO:1902742 | apoptotic process involved in development | 5/348 | 0.007126804 | 5 |
| GO:0042102 | positive regulation of T cell proliferation | 8/348 | 0.007157975 | 8 |
| GO:0002698 | negative regulation of immune effector process | 9/348 | 0.007553381 | 9 |
| GO:0006836 | neurotransmitter transport | 12/348 | 0.007581804 | 12 |
| GO:0046641 | positive regulation of alpha-beta T cell proliferation | 4/348 | 0.0075917 | 4 |
| GO:0060402 | calcium ion transport into cytosol | 10/348 | 0.0075917 | 10 |
| GO:0032731 | positive regulation of interleukin-1 beta production | 6/348 | 0.007957193 | 6 |
| GO:0060760 | positive regulation of response to cytokine stimulus | 6/348 | 0.007957193 | 6 |
| GO:0046634 | regulation of alpha-beta T cell activation | 8/348 | 0.007957193 | 8 |
| GO:0014066 | regulation of phosphatidylinositol 3-kinase signaling | 9/348 | 0.008211806 | 9 |
| GO:0061844 | antimicrobial humoral immune response mediated by antimicrobial peptide | 7/348 | 0.008211806 | 7 |
| GO:0014065 | phosphatidylinositol 3-kinase signaling | 10/348 | 0.008211806 | 10 |
| GO:1904062 | regulation of cation transmembrane transport | 16/348 | 0.008522531 | 16 |
| GO:0031295 | T cell costimulation | 6/348 | 0.008522531 | 6 |
| GO:0086065 | cell communication involved in cardiac conduction | 6/348 | 0.008522531 | 6 |
| GO:0032479 | regulation of type I interferon production | 9/348 | 0.008522531 | 9 |
| GO:0001914 | regulation of T cell mediated cytotoxicity | 5/348 | 0.008638365 | 5 |
| GO:0002710 | negative regulation of T cell mediated immunity | 4/348 | 0.008683599 | 4 |
| GO:0045061 | thymic T cell selection | 4/348 | 0.008683599 | 4 |
| GO:0032651 | regulation of interleukin-1 beta production | 8/348 | 0.008688152 | 8 |
| GO:0032606 | type I interferon production | 9/348 | 0.008844652 | 9 |
| GO:0019884 | antigen processing and presentation of exogenous antigen | 11/348 | 0.008952729 | 11 |
| GO:0002765 | immune response-inhibiting signal transduction | 3/348 | 0.008960484 | 3 |
| GO:0032060 | bleb assembly | 3/348 | 0.008960484 | 3 |
| GO:0032661 | regulation of interleukin-18 production | 3/348 | 0.008960484 | 3 |
| GO:0034776 | response to histamine | 3/348 | 0.008960484 | 3 |
| GO:0031663 | lipopolysaccharide-mediated signaling pathway | 6/348 | 0.008967707 | 6 |
| GO:0002821 | positive regulation of adaptive immune response | 8/348 | 0.008989467 | 8 |
| GO:0007612 | learning | 9/348 | 0.009075409 | 9 |
| GO:0032675 | regulation of interleukin-6 production | 10/348 | 0.009361939 | 10 |
| GO:0009914 | hormone transport | 15/348 | 0.009437851 | 15 |
| GO:0062207 | regulation of pattern recognition receptor signaling pathway | 8/348 | 0.009437851 | 8 |
| GO:0140353 | lipid export from cell | 7/348 | 0.009516656 | 7 |
| GO:0031294 | lymphocyte costimulation | 6/348 | 0.00958091 | 6 |
| GO:0043302 | positive regulation of leukocyte degranulation | 4/348 | 0.009761547 | 4 |
| GO:0007229 | integrin-mediated signaling pathway | 8/348 | 0.009903043 | 8 |
| GO:0002285 | lymphocyte activation involved in immune response | 11/348 | 0.010074383 | 11 |
| GO:0060759 | regulation of response to cytokine stimulus | 11/348 | 0.010074383 | 11 |
| GO:0032735 | positive regulation of interleukin-12 production | 5/348 | 0.010127991 | 5 |
| GO:0050729 | positive regulation of inflammatory response | 9/348 | 0.010273714 | 9 |
| GO:0032409 | regulation of transporter activity | 14/348 | 0.01027611 | 14 |
| GO:0032649 | regulation of interferon-gamma production | 8/348 | 0.01027611 | 8 |
| GO:0046632 | alpha-beta T cell differentiation | 8/348 | 0.01027611 | 8 |
| GO:0050853 | B cell receptor signaling pathway | 9/348 | 0.010649659 | 9 |
| GO:0097553 | calcium ion transmembrane import into cytosol | 9/348 | 0.010649659 | 9 |
| GO:0032635 | interleukin-6 production | 10/348 | 0.010721833 | 10 |
| GO:0032753 | positive regulation of interleukin-4 production | 4/348 | 0.011027309 | 4 |
| GO:0098581 | detection of external biotic stimulus | 4/348 | 0.011027309 | 4 |
| GO:0002718 | regulation of cytokine production involved in immune response | 7/348 | 0.011077955 | 7 |
| GO:0032493 | response to bacterial lipoprotein | 3/348 | 0.011077955 | 3 |
| GO:0033632 | regulation of cell-cell adhesion mediated by integrin | 3/348 | 0.011077955 | 3 |
| GO:0046643 | regulation of gamma-delta T cell activation | 3/348 | 0.011077955 | 3 |
| GO:0070371 | ERK1 and ERK2 cascade | 15/348 | 0.011296322 | 15 |
| GO:0050920 | regulation of chemotaxis | 12/348 | 0.011583065 | 12 |
| GO:0019933 | cAMP-mediated signaling | 6/348 | 0.011598976 | 6 |
| GO:0045921 | positive regulation of exocytosis | 7/348 | 0.01170912 | 7 |
| GO:0030183 | B cell differentiation | 9/348 | 0.011964707 | 9 |
| GO:0032755 | positive regulation of interleukin-6 production | 7/348 | 0.012424334 | 7 |
| GO:0001562 | response to protozoan | 4/348 | 0.012424334 | 4 |
| GO:0042832 | defense response to protozoan | 4/348 | 0.012424334 | 4 |
| GO:0021782 | glial cell development | 8/348 | 0.012951411 | 8 |
| GO:0032609 | interferon-gamma production | 8/348 | 0.012951411 | 8 |
| GO:0002433 | immune response-regulating cell surface receptor signaling pathway involved in phagocytosis | 9/348 | 0.012951411 | 9 |
| GO:0038096 | Fc-gamma receptor signaling pathway involved in phagocytosis | 9/348 | 0.012951411 | 9 |
| GO:0051899 | membrane depolarization | 7/348 | 0.013041002 | 7 |
| GO:0032732 | positive regulation of interleukin-1 production | 6/348 | 0.013227452 | 6 |
| GO:0002664 | regulation of T cell tolerance induction | 3/348 | 0.01372886 | 3 |
| GO:0032490 | detection of molecule of bacterial origin | 3/348 | 0.01372886 | 3 |
| GO:0034135 | regulation of toll-like receptor 2 signaling pathway | 3/348 | 0.01372886 | 3 |
| GO:0038110 | interleukin-2-mediated signaling pathway | 3/348 | 0.01372886 | 3 |
| GO:2000116 | regulation of cysteine-type endopeptidase activity | 12/348 | 0.01374997 | 12 |
| GO:0010863 | positive regulation of phospholipase C activity | 5/348 | 0.014085718 | 5 |
| GO:0072678 | T cell migration | 6/348 | 0.01519893 | 6 |
| GO:0002707 | negative regulation of lymphocyte mediated immunity | 5/348 | 0.015462464 | 5 |
| GO:0034122 | negative regulation of toll-like receptor signaling pathway | 5/348 | 0.015462464 | 5 |
| GO:0090025 | regulation of monocyte chemotaxis | 4/348 | 0.015878093 | 4 |
| GO:0050806 | positive regulation of synaptic transmission | 9/348 | 0.015878093 | 9 |
| GO:0060401 | cytosolic calcium ion transport | 10/348 | 0.015878093 | 10 |
| GO:0031640 | killing of cells of other organism | 6/348 | 0.016081908 | 6 |
| GO:0050766 | positive regulation of phagocytosis | 6/348 | 0.016081908 | 6 |
| GO:0046879 | hormone secretion | 14/348 | 0.016544104 | 14 |
| GO:0051932 | synaptic transmission, GABAergic | 5/348 | 0.016544104 | 5 |
| GO:1900274 | regulation of phospholipase C activity | 5/348 | 0.016544104 | 5 |
| GO:0002685 | regulation of leukocyte migration | 11/348 | 0.016544104 | 11 |
| GO:0002923 | regulation of humoral immune response mediated by circulating immunoglobulin | 3/348 | 0.016544104 | 3 |
| GO:0033008 | positive regulation of mast cell activation involved in immune response | 3/348 | 0.016544104 | 3 |
| GO:0043306 | positive regulation of mast cell degranulation | 3/348 | 0.016544104 | 3 |
| GO:0070486 | leukocyte aggregation | 3/348 | 0.016544104 | 3 |
| GO:0071352 | cellular response to interleukin-2 | 3/348 | 0.016544104 | 3 |
| GO:0097091 | synaptic vesicle clustering | 3/348 | 0.016544104 | 3 |
| GO:0002700 | regulation of production of molecular mediator of immune response | 9/348 | 0.016745245 | 9 |
| GO:0008306 | associative learning | 6/348 | 0.016745245 | 6 |
| GO:0051090 | regulation of DNA-binding transcription factor activity | 18/348 | 0.016912406 | 18 |
| GO:0002507 | tolerance induction | 4/348 | 0.017305071 | 4 |
| GO:0002367 | cytokine production involved in immune response | 7/348 | 0.017484668 | 7 |
| GO:0006835 | dicarboxylic acid transport | 7/348 | 0.017484668 | 7 |
| GO:0043254 | regulation of protein-containing complex assembly | 18/348 | 0.017522447 | 18 |
| GO:0038093 | Fc receptor signaling pathway | 12/348 | 0.017522447 | 12 |
| GO:0001959 | regulation of cytokine-mediated signaling pathway | 10/348 | 0.017563476 | 10 |
| GO:0002478 | antigen processing and presentation of exogenous peptide antigen | 10/348 | 0.017563476 | 10 |
| GO:0051260 | protein homooligomerization | 10/348 | 0.017563476 | 10 |
| GO:0015718 | monocarboxylic acid transport | 10/348 | 0.01825865 | 10 |
| GO:0060337 | type I interferon signaling pathway | 7/348 | 0.019366176 | 7 |
| GO:0001505 | regulation of neurotransmitter levels | 11/348 | 0.019758122 | 11 |
| GO:0002517 | T cell tolerance induction | 3/348 | 0.019758122 | 3 |
| GO:0035589 | G protein-coupled purinergic nucleotide receptor signaling pathway | 3/348 | 0.019758122 | 3 |
| GO:0070669 | response to interleukin-2 | 3/348 | 0.019758122 | 3 |
| GO:0071357 | cellular response to type I interferon | 7/348 | 0.020272103 | 7 |
| GO:0001961 | positive regulation of cytokine-mediated signaling pathway | 5/348 | 0.020467828 | 5 |
| GO:0071622 | regulation of granulocyte chemotaxis | 5/348 | 0.020467828 | 5 |
| GO:0006865 | amino acid transport | 9/348 | 0.02087116 | 9 |
| GO:0033627 | cell adhesion mediated by integrin | 6/348 | 0.021118107 | 6 |
| GO:0086003 | cardiac muscle cell contraction | 6/348 | 0.021118107 | 6 |
| GO:0060191 | regulation of lipase activity | 7/348 | 0.021118107 | 7 |
| GO:0032743 | positive regulation of interleukin-2 production | 4/348 | 0.021118107 | 4 |
| GO:0045577 | regulation of B cell differentiation | 4/348 | 0.021118107 | 4 |
| GO:0072593 | reactive oxygen species metabolic process | 13/348 | 0.022352958 | 13 |
| GO:0002292 | T cell differentiation involved in immune response | 6/348 | 0.022377759 | 6 |
| GO:0007249 | I-kappaB kinase/NF-kappaB signaling | 13/348 | 0.02292588 | 13 |
| GO:0007269 | neurotransmitter secretion | 9/348 | 0.02299899 | 9 |
| GO:0099643 | signal release from synapse | 9/348 | 0.02299899 | 9 |
| GO:0002824 | positive regulation of adaptive immune response based on somatic recombination of immune receptors built from immunoglobulin superfamily domains | 7/348 | 0.02299899 | 7 |
| GO:0019233 | sensory perception of pain | 7/348 | 0.02299899 | 7 |
| GO:0045579 | positive regulation of B cell differentiation | 3/348 | 0.02299899 | 3 |
| GO:0070207 | protein homotrimerization | 3/348 | 0.02299899 | 3 |
| GO:1900225 | regulation of NLRP3 inflammasome complex assembly | 3/348 | 0.02299899 | 3 |
| GO:0045761 | regulation of adenylate cyclase activity | 4/348 | 0.02299899 | 4 |
| GO:0070229 | negative regulation of lymphocyte apoptotic process | 4/348 | 0.02299899 | 4 |
| GO:0099174 | regulation of presynapse organization | 4/348 | 0.02299899 | 4 |
| GO:1905606 | regulation of presynapse assembly | 4/348 | 0.02299899 | 4 |
| GO:0043277 | apoptotic cell clearance | 5/348 | 0.02308406 | 5 |
| GO:0032418 | lysosome localization | 6/348 | 0.02308406 | 6 |
| GO:0002832 | negative regulation of response to biotic stimulus | 7/348 | 0.023873959 | 7 |
| GO:0002576 | platelet degranulation | 8/348 | 0.024507225 | 8 |
| GO:0034340 | response to type I interferon | 7/348 | 0.025121209 | 7 |
| GO:0032673 | regulation of interleukin-4 production | 4/348 | 0.025361787 | 4 |
| GO:0090022 | regulation of neutrophil chemotaxis | 4/348 | 0.025361787 | 4 |
| GO:0090322 | regulation of superoxide metabolic process | 4/348 | 0.025361787 | 4 |
| GO:0086001 | cardiac muscle cell action potential | 6/348 | 0.025934493 | 6 |
| GO:0032102 | negative regulation of response to external stimulus | 16/348 | 0.026039075 | 16 |
| GO:0044546 | NLRP3 inflammasome complex assembly | 3/348 | 0.026881406 | 3 |
| GO:0071800 | podosome assembly | 3/348 | 0.026881406 | 3 |
| GO:0050728 | negative regulation of inflammatory response | 9/348 | 0.02705841 | 9 |
| GO:0007200 | phospholipase C-activating G protein-coupled receptor signaling pathway | 7/348 | 0.027397349 | 7 |
| GO:0002724 | regulation of T cell cytokine production | 4/348 | 0.027774868 | 4 |
| GO:0032633 | interleukin-4 production | 4/348 | 0.027774868 | 4 |
| GO:0050869 | negative regulation of B cell activation | 4/348 | 0.027774868 | 4 |
| GO:0032412 | regulation of ion transmembrane transporter activity | 12/348 | 0.027774868 | 12 |
| GO:0007626 | locomotory behavior | 9/348 | 0.027774868 | 9 |
| GO:0048015 | phosphatidylinositol-mediated signaling | 10/348 | 0.027805822 | 10 |
| GO:0086002 | cardiac muscle cell action potential involved in contraction | 5/348 | 0.02813956 | 5 |
| GO:0097479 | synaptic vesicle localization | 5/348 | 0.02813956 | 5 |
| GO:0016079 | synaptic vesicle exocytosis | 7/348 | 0.028261753 | 7 |
| GO:0006898 | receptor-mediated endocytosis | 14/348 | 0.029088393 | 14 |
| GO:0048002 | antigen processing and presentation of peptide antigen | 10/348 | 0.029565323 | 10 |
| GO:0051051 | negative regulation of transport | 17/348 | 0.029706723 | 17 |
| GO:0010543 | regulation of platelet activation | 4/348 | 0.030263277 | 4 |
| GO:0030889 | negative regulation of B cell proliferation | 3/348 | 0.030681042 | 3 |
| GO:0033631 | cell-cell adhesion mediated by integrin | 3/348 | 0.030681042 | 3 |
| GO:0070206 | protein trimerization | 3/348 | 0.030681042 | 3 |
| GO:0150079 | negative regulation of neuroinflammatory response | 3/348 | 0.030681042 | 3 |
| GO:0048259 | regulation of receptor-mediated endocytosis | 7/348 | 0.030727863 | 7 |
| GO:0048017 | inositol lipid-mediated signaling | 10/348 | 0.031173842 | 10 |
| GO:0060078 | regulation of postsynaptic membrane potential | 8/348 | 0.032932391 | 8 |
| GO:0042554 | superoxide anion generation | 4/348 | 0.033016635 | 4 |
| GO:0050672 | negative regulation of lymphocyte proliferation | 6/348 | 0.033303886 | 6 |
| GO:0070372 | regulation of ERK1 and ERK2 cascade | 13/348 | 0.035215458 | 13 |
| GO:0032945 | negative regulation of mononuclear cell proliferation | 6/348 | 0.035215458 | 6 |
| GO:0002643 | regulation of tolerance induction | 3/348 | 0.035525144 | 3 |
| GO:0032695 | negative regulation of interleukin-12 production | 3/348 | 0.035525144 | 3 |
| GO:0002369 | T cell cytokine production | 4/348 | 0.03599279 | 4 |
| GO:0043368 | positive T cell selection | 4/348 | 0.03599279 | 4 |
| GO:0016358 | dendrite development | 11/348 | 0.036431647 | 11 |
| GO:0009612 | response to mechanical stimulus | 10/348 | 0.037531284 | 10 |
| GO:0070588 | calcium ion transmembrane transport | 13/348 | 0.037577153 | 13 |
| GO:0031623 | receptor internalization | 7/348 | 0.038203445 | 7 |
| GO:0071675 | regulation of mononuclear cell migration | 7/348 | 0.038203445 | 7 |
| GO:0015698 | inorganic anion transport | 9/348 | 0.038387163 | 9 |
| GO:0035637 | multicellular organismal signaling | 10/348 | 0.038387163 | 10 |
| GO:0032663 | regulation of interleukin-2 production | 5/348 | 0.038597859 | 5 |
| GO:0048713 | regulation of oligodendrocyte differentiation | 4/348 | 0.038931711 | 4 |
| GO:0002573 | myeloid leukocyte differentiation | 10/348 | 0.039443424 | 10 |
| GO:0051209 | release of sequestered calcium ion into cytosol | 7/348 | 0.039555934 | 7 |
| GO:0032288 | myelin assembly | 3/348 | 0.039596887 | 3 |
| GO:0033005 | positive regulation of mast cell activation | 3/348 | 0.039596887 | 3 |
| GO:0043031 | negative regulation of macrophage activation | 3/348 | 0.039596887 | 3 |
| GO:0043652 | engulfment of apoptotic cell | 3/348 | 0.039596887 | 3 |
| GO:0086014 | atrial cardiac muscle cell action potential | 3/348 | 0.039596887 | 3 |
| GO:0086026 | atrial cardiac muscle cell to AV node cell signaling | 3/348 | 0.039596887 | 3 |
| GO:0086066 | atrial cardiac muscle cell to AV node cell communication | 3/348 | 0.039596887 | 3 |
| GO:2000369 | regulation of clathrin-dependent endocytosis | 3/348 | 0.039596887 | 3 |
| GO:0032722 | positive regulation of chemokine production | 5/348 | 0.040151446 | 5 |
| GO:0034113 | heterotypic cell-cell adhesion | 5/348 | 0.040151446 | 5 |
| GO:0099072 | regulation of postsynaptic membrane neurotransmitter receptor levels | 5/348 | 0.040151446 | 5 |
| GO:0051283 | negative regulation of sequestering of calcium ion | 7/348 | 0.040378401 | 7 |
| GO:0030099 | myeloid cell differentiation | 16/348 | 0.041023839 | 16 |
| GO:0009595 | detection of biotic stimulus | 4/348 | 0.04104598 | 4 |
| GO:0032733 | positive regulation of interleukin-10 production | 4/348 | 0.04104598 | 4 |
| GO:0042417 | dopamine metabolic process | 4/348 | 0.04104598 | 4 |
| GO:2000249 | regulation of actin cytoskeleton reorganization | 4/348 | 0.04104598 | 4 |
| GO:0002223 | stimulatory C-type lectin receptor signaling pathway | 7/348 | 0.04178375 | 7 |
| GO:0032623 | interleukin-2 production | 5/348 | 0.042312937 | 5 |
| GO:0007009 | plasma membrane organization | 7/348 | 0.043512295 | 7 |
| GO:0051282 | regulation of sequestering of calcium ion | 7/348 | 0.043512295 | 7 |
| GO:0001764 | neuron migration | 8/348 | 0.043830033 | 8 |
| GO:1903169 | regulation of calcium ion transmembrane transport | 8/348 | 0.043830033 | 8 |
| GO:0051651 | maintenance of location in cell | 10/348 | 0.043891669 | 10 |
| GO:0002577 | regulation of antigen processing and presentation | 3/348 | 0.043891669 | 3 |
| GO:0090026 | positive regulation of monocyte chemotaxis | 3/348 | 0.043891669 | 3 |
| GO:0050832 | defense response to fungus | 4/348 | 0.043891669 | 4 |
| GO:0086091 | regulation of heart rate by cardiac conduction | 4/348 | 0.043891669 | 4 |
| GO:1903539 | protein localization to postsynaptic membrane | 4/348 | 0.043891669 | 4 |
| GO:0070664 | negative regulation of leukocyte proliferation | 6/348 | 0.044813186 | 6 |
| GO:0052548 | regulation of endopeptidase activity | 16/348 | 0.046002809 | 16 |
| GO:0019935 | cyclic-nucleotide-mediated signaling | 6/348 | 0.04683717 | 6 |
| GO:0032642 | regulation of chemokine production | 6/348 | 0.04683717 | 6 |
| GO:0017156 | calcium-ion regulated exocytosis | 5/348 | 0.04683717 | 5 |
| GO:0071677 | positive regulation of mononuclear cell migration | 5/348 | 0.04683717 | 5 |
| GO:0030890 | positive regulation of B cell proliferation | 4/348 | 0.047342062 | 4 |
| GO:0010975 | regulation of neuron projection development | 16/348 | 0.047420255 | 16 |
| GO:0051928 | positive regulation of calcium ion transport | 7/348 | 0.048144557 | 7 |
| GO:0022010 | central nervous system myelination | 3/348 | 0.049038969 | 3 |
| GO:0031629 | synaptic vesicle fusion to presynaptic active zone membrane | 3/348 | 0.049038969 | 3 |
| GO:0032291 | axon ensheathment in central nervous system | 3/348 | 0.049038969 | 3 |
| GO:0099500 | vesicle fusion to plasma membrane | 3/348 | 0.049038969 | 3 |
| GO:0042267 | natural killer cell mediated cytotoxicity | 5/348 | 0.0491442 | 5 |
| GO:0061337 | cardiac conduction | 8/348 | 0.0491442 | 8 |
