# Supplementary material for: Effective biomarkers and therapeutic targets of nerve-immunity interaction in the treatment of depression: an integrated investigation of the miRNA-mRNA regulatory networks
Source: Aging (Albany NY). 2022 Apr 25;14(8):3569–96. doi: 10.18632/aging.204030 (PMC9085226; doi:10.18632/aging.204030)
Supplement: Supplementary Tables 5-9 and 11 [file aging-14-204030-s002.pdf]

SUPPLEMENTARY TABLES

Supplementary Table 5. BP of DE-miRNAs.

Biological process	Percentage of genes	*p*-value	*p* = 0.05 references
Regulation of nucleobase, nucleoside, nucleotide and nucleic acid metabolism	19.61301671	1.723636772	1.301029996
Signal transduction	25.41776605	0.691560447	1.301029996
Cell communication	23.21899736	0	1.301029996
Synaptic transmission	0.175901495	0	1.301029996
Protein transport	0.263852243	0	1.301029996
Cytoskeleton organization and biogenesis	0.35180299	0	1.301029996
Regulation of cell growth	0.35180299	0	1.301029996
Transport	7.915567282	0	1.301029996
Nucleobase, nucleoside, nucleotide and nucleic acid transport	0.175901495	0	1.301029996
Vitamin metabolism	0.087950748	0	1.301029996
RNA localization	0.087950748	0	1.301029996
DNA metabolism	0.087950748	0	1.301029996
Cell cycle	0.263852243	0	1.301029996
Steroid metabolism	0.087950748	0	1.301029996
Wound healing	0.087950748	0	1.301029996
Endosome transport	0.087950748	0	1.301029996
Signal complex formation	0.087950748	0	1.301029996
Regulation of translation	0.175901495	0	1.301029996
Cell proliferation	0.35180299	0	1.301029996
Cell recognition	0.087950748	0	1.301029996
Regulation of endocytosis	0.087950748	0	1.301029996
Neurotransmitter transport	0.087950748	0	1.301029996
Vesicle docking	0.087950748	0	1.301029996
Regulation of gene expression, epigenetic	0.527704485	0	1.301029996
Cell migration	0.175901495	0	1.301029996
Regulation of metabolism	0.087950748	0	1.301029996
Regulation of exocytosis	0.087950748	0	1.301029996
Protein targeting	0.087950748	0	1.301029996
Regulation of cell cycle	0.439753738	0	1.301029996
Development	0.087950748	0	1.301029996
Protein modification	0.175901495	0	1.301029996
Cell organization and biogenesis	0.087950748	0	1.301029996
Carbohydrate metabolism	0.087950748	0	1.301029996
Cell surface receptor linked signal transduction	0.087950748	0	1.301029996
Regulation of immune response	0.087950748	0	1.301029996
Regulation of signal transduction	0.175901495	0	1.301029996
G-protein coupled receptor protein signaling pathway	0.087950748	0	1.301029996

RNA metabolism	0.175901495	0	1.301029996
Embryonic development	0.087950748	0	1.301029996
Apoptosis	1.231310466	0	1.301029996
Cell-cell adhesion	0.087950748	0	1.301029996
Cell differentiation	0.175901495	0	1.301029996
Anti-apoptosis	0.175901495	0	1.301029996
Protein folding	0.087950748	0	1.301029996
Cell-cell signaling	0.087950748	0	1.301029996
Vesicle-mediated transport	0.087950748	0	1.301029996
Cell motility	0.087950748	0	1.301029996
Regulation of cell proliferation	0.087950748	0	1.301029996
Transcription	0.175901495	0	1.301029996
Protein metabolism	6.596306069	0	1.301029996
Cell adhesion	0.175901495	0	1.301029996
Lipid metabolism	0.087950748	0	1.301029996
Cell growth and/or maintenance	5.277044855	0	1.301029996
Ion transport	0.087950748	0	1.301029996
DNA repair	0.087950748	0	1.301029996
Metabolism	7.299912049	0	1.301029996
Energy pathways	6.860158311	0	1.301029996
Biological_process unknown	22.51539138	0	1.301029996
Immune response	1.231310466	0	1.301029996

Supplementary Table 6. 16 core target genes of hub DE-mRNAs.

Name	SUID	Betweenness	Closeness	Degree
CACNA2D1	72	3.666666667	0.277777778	5
SNAP25	73	34.83333333	0.3125	11
GJA1	75	4.666666667	0.283018868	6
KCND2	77	54.66666667	0.306122449	9
GNAI1	79	0.5	0.25862069	3
TUSC3	81	0	0.272727273	4
CHL1	83	11.66666667	0.288461538	7
SOX9	84	0.5	0.263157895	4
PCDH17	87	1	0.263157895	4
MYT1L	92	4.5	0.283018868	6
SASH3	101	24	0.254237288	2
LMO3	103	2	0.277777778	5
NOVA1	105	2	0.277777778	5
MAL2	112	0	0.066666667	1
RAB37	113	0	0.066666667	1
RNASE6	121	0	0.211267606	1

Supplementary Table 7. Filter once after the 5 core target genes of hub DE-mRNAs.

LAC	SUID	Betweenness	Closeness	Degree
2.5	77	0.666666667	1	4
2	92	0	0.8	3
2	75	0	0.8	3
2.5	83	0.666666667	1	4
2.5	73	0.666666667	1	4

Supplementary Table 8. MF of interaction mRNA of miRNA-mRNA regulatory network.

ID	Description	GeneRatio	*p*.adjust	Count
GO:0140375	immune receptor activity	21/352	9.01E-11	21
GO:0004896	cytokine receptor activity	15/352	1.37E-07	15
GO:0035325	Toll-like receptor binding	5/352	0.000228123	5
GO:0019955	cytokine binding	13/352	0.000228123	13
GO:0004715	non-membrane spanning protein tyrosine kinase activity	8/352	0.000228123	8
GO:0019956	chemokine binding	7/352	0.000228123	7
GO:0022851	GABA-gated chloride ion channel activity	5/352	0.000228123	5
GO:0016493	C-C chemokine receptor activity	6/352	0.000228123	6
GO:0038187	pattern recognition receptor activity	6/352	0.000228123	6
GO:0019957	C-C chemokine binding	6/352	0.000269341	6
GO:0001637	G protein-coupled chemoattractant receptor activity	6/352	0.000371676	6
GO:0004950	chemokine receptor activity	6/352	0.000371676	6
GO:0005001	transmembrane receptor protein tyrosine phosphatase activity	5/352	0.000517705	5
GO:0019198	transmembrane receptor protein phosphatase activity	5/352	0.000517705	5
GO:0099095	ligand-gated anion channel activity	5/352	0.000658534	5
GO:0004890	GABA-A receptor activity	5/352	0.000752724	5
GO:0019911	structural constituent of myelin sheath	4/352	0.000752724	4
GO:0050786	RAGE receptor binding	4/352	0.000752724	4
GO:0071723	lipopeptide binding	4/352	0.000752724	4
GO:0048306	calcium-dependent protein binding	9/352	0.000926647	9
GO:0042608	T cell receptor binding	4/352	0.001054007	4
GO:0016917	GABA receptor activity	5/352	0.001295489	5
GO:0030246	carbohydrate binding	16/352	0.001517833	16
GO:0019865	immunoglobulin binding	5/352	0.001857169	5
GO:0017075	syntaxin-1 binding	5/352	0.002570744	5
GO:0023023	MHC protein complex binding	5/352	0.002570744	5
GO:0022824	transmitter-gated ion channel activity	7/352	0.002614215	7
GO:0022835	transmitter-gated channel activity	7/352	0.002614215	7
GO:0015108	chloride transmembrane transporter activity	9/352	0.002692073	9
GO:0015267	channel activity	22/352	0.002692073	22
GO:0022803	passive transmembrane transporter activity	22/352	0.002692073	22
GO:0005237	inhibitory extracellular ligand-gated ion channel activity	4/352	0.002692073	4

GO:0017124	SH3 domain binding	10/352	0.003006658	10
GO:0001784	phosphotyrosine residue binding	6/352	0.003006658	6
GO:0003953	NAD+ nucleosidase activity	4/352	0.003057546	4
GO:0050135	NAD(P)+ nucleosidase activity	4/352	0.003057546	4
GO:0061809	NAD+ nucleotidase, cyclic ADP-ribose generating	4/352	0.003057546	4
GO:0099529	neurotransmitter receptor activity involved in regulation of postsynaptic membrane potential	6/352	0.003898996	6
GO:0004713	protein tyrosine kinase activity	10/352	0.004040285	10
GO:0030594	neurotransmitter receptor activity	9/352	0.004044242	9
GO:0005230	extracellular ligand-gated ion channel activity	7/352	0.006780537	7
GO:0005254	chloride channel activity	7/352	0.007192201	7
GO:0001882	nucleoside binding	18/352	0.007480743	18
GO:0001848	complement binding	4/352	0.007837425	4
GO:0045309	protein phosphorylated amino acid binding	6/352	0.008614962	6
GO:0042169	SH2 domain binding	5/352	0.009055381	5
GO:0016176	superoxide-generating NADPH oxidase activator activity	3/352	0.009055381	3
GO:0022836	gated channel activity	16/352	0.00993357	16
GO:0005525	GTP binding	17/352	0.011094302	17
GO:0008503	benzodiazepine receptor activity	3/352	0.011094302	3
GO:0016175	superoxide-generating NAD(P)H oxidase activity	3/352	0.011094302	3
GO:0019864	IgG binding	3/352	0.011094302	3
GO:0032550	purine ribonucleoside binding	17/352	0.012346261	17
GO:0098960	postsynaptic neurotransmitter receptor activity	6/352	0.012346261	6
GO:0001883	purine nucleoside binding	17/352	0.012761612	17
GO:0032549	ribonucleoside binding	17/352	0.012761612	17
GO:0005253	anion channel activity	7/352	0.013011667	7
GO:0051219	phosphoprotein binding	7/352	0.013681766	7
GO:0003924	GTPase activity	15/352	0.013865507	15
GO:1904315	transmitter-gated ion channel activity involved in regulation of postsynaptic membrane potential	5/352	0.013865507	5
GO:0005216	ion channel activity	18/352	0.015503232	18
GO:0036041	long-chain fatty acid binding	3/352	0.015674859	3
GO:0019001	guanyl nucleotide binding	17/352	0.016334476	17
GO:0032561	guanyl ribonucleotide binding	17/352	0.016334476	17
GO:0015103	inorganic anion transmembrane transporter activity	9/352	0.016936842	9
GO:0015631	tubulin binding	16/352	0.0178735	16
GO:0045028	G protein-coupled purinergic nucleotide receptor activity	3/352	0.017942731	3
GO:0008017	microtubule binding	13/352	0.017942731	13
GO:0001846	opsonin binding	3/352	0.021467256	3
GO:0097153	cysteine-type endopeptidase activity involved in apoptotic process	3/352	0.021467256	3
GO:0031406	carboxylic acid binding	10/352	0.023664169	10
GO:0050664	oxidoreductase activity, acting on NAD(P)H, oxygen as acceptor	3/352	0.024977807	3
GO:0050700	CARD domain binding	3/352	0.024977807	3

GO:0023026	MHC class II protein complex binding	3/352	0.029105081	3
GO:0098918	structural constituent of synapse	3/352	0.029105081	3
GO:0005504	fatty acid binding	4/352	0.039026991	4
GO:0015276	ligand-gated ion channel activity	8/352	0.039026991	8
GO:0022834	ligand-gated channel activity	8/352	0.039026991	8
GO:0001540	amyloid-beta binding	6/352	0.03929177	6
GO:0016799	hydrolase activity, hydrolyzing N-glycosyl compounds	4/352	0.041154883	4
GO:0005178	integrin binding	8/352	0.042635723	8
GO:0043028	cysteine-type endopeptidase regulator activity involved in apoptotic process	4/352	0.048026669	4
GO:0001614	purinergic nucleotide receptor activity	3/352	0.048026669	3

Supplementary Table 9. CC of interaction mRNA of miRNA-mRNA regulatory network.

ID	Description	GeneRatio	*p*.adjust	Count
GO:0030667	secretory granule membrane	28/364	1.68E-09	28
GO:0098978	glutamatergic synapse	26/364	3.23E-08	26
GO:0097060	synaptic membrane	28/364	4.63E-08	28
GO:0009897	external side of plasma membrane	29/364	5.80E-08	29
GO:0098793	presynapse	31/364	2.42E-07	31
GO:0098984	neuron to neuron synapse	25/364	4.74E-07	25
GO:0034774	secretory granule lumen	24/364	5.56E-07	24
GO:0070820	tertiary granule	17/364	5.56E-07	17
GO:0060205	cytoplasmic vesicle lumen	24/364	5.56E-07	24
GO:0031983	vesicle lumen	24/364	5.63E-07	24
GO:0098982	GABA-ergic synapse	11/364	9.23E-07	11
GO:0045121	membrane raft	23/364	1.44E-06	23
GO:0098857	membrane microdomain	23/364	1.44E-06	23
GO:0099240	intrinsic component of synaptic membrane	15/364	1.65E-06	15
GO:0099699	integral component of synaptic membrane	14/364	3.30E-06	14
GO:0042734	presynaptic membrane	14/364	7.23E-06	14
GO:0043025	neuronal cell body	27/364	7.29E-06	27
GO:0099572	postsynaptic specialization	22/364	7.51E-06	22
GO:0101002	ficolin-1-rich granule	16/364	8.69E-06	16
GO:0098802	plasma membrane signaling receptor complex	21/364	1.21E-05	21
GO:0045211	postsynaptic membrane	19/364	1.21E-05	19
GO:0014069	postsynaptic density	20/364	3.54E-05	20
GO:0032279	asymmetric synapse	20/364	4.32E-05	20
GO:0098889	intrinsic component of presynaptic membrane	9/364	4.65E-05	9
GO:0031225	anchored component of membrane	14/364	6.26E-05	14
GO:0098936	intrinsic component of postsynaptic membrane	11/364	6.61E-05	11
GO:0099056	integral component of presynaptic membrane	8/364	0.00013213	8
GO:0045335	phagocytic vesicle	12/364	0.000136097	12
GO:1902495	transmembrane transporter complex	19/364	0.000159039	19

GO:0034702	ion channel complex	18/364	0.000209447	18
GO:0099055	integral component of postsynaptic membrane	10/364	0.000229285	10
GO:1902711	GABA-A receptor complex	5/364	0.000260303	5
GO:1990351	transporter complex	19/364	0.000263284	19
GO:1902710	GABA receptor complex	5/364	0.000321676	5
GO:0043197	dendritic spine	13/364	0.000336742	13
GO:0044309	neuron spine	13/364	0.000347337	13
GO:0042581	specific granule	12/364	0.000524537	12
GO:0035579	specific granule membrane	9/364	0.000537283	9
GO:0070821	tertiary granule membrane	8/364	0.000657384	8
GO:0030139	endocytic vesicle	17/364	0.000706349	17
GO:0001772	immunological synapse	6/364	0.001032584	6
GO:0043204	perikaryon	11/364	0.001232409	11
GO:0101003	ficolin-1-rich granule membrane	7/364	0.001279911	7
GO:0098686	hippocampal mossy fiber to CA3 synapse	5/364	0.001960147	5
GO:0099060	integral component of postsynaptic specialization membrane	7/364	0.002016145	7
GO:0098685	Schaffer collateral - CA1 synapse	7/364	0.002167655	7
GO:0044291	cell-cell contact zone	7/364	0.002496484	7
GO:0098948	intrinsic component of postsynaptic specialization membrane	7/364	0.002496484	7
GO:0034707	chloride channel complex	6/364	0.002652121	6
GO:0005911	cell-cell junction	21/364	0.002677307	21
GO:0030665	clathrin-coated vesicle membrane	9/364	0.002762444	9
GO:1904813	ficolin-1-rich granule lumen	9/364	0.004137096	9
GO:1904724	tertiary granule lumen	6/364	0.004137096	6
GO:0034703	cation channel complex	12/364	0.007380051	12
GO:0099634	postsynaptic specialization membrane	8/364	0.007380051	8
GO:0150034	distal axon	14/364	0.007587933	14
GO:0032590	dendrite membrane	5/364	0.008383255	5
GO:0043020	NADPH oxidase complex	3/364	0.008717461	3
GO:0019897	extrinsic component of plasma membrane	10/364	0.008969762	10
GO:0019898	extrinsic component of membrane	14/364	0.012170347	14
GO:0014704	intercalated disc	5/364	0.012916047	5
GO:0030427	site of polarized growth	10/364	0.016006508	10
GO:0008305	integrin complex	4/364	0.016159135	4
GO:0031234	extrinsic component of cytoplasmic side of plasma membrane	7/364	0.017005993	7
GO:0005767	secondary lysosome	3/364	0.018734247	3
GO:0044306	neuron projection terminus	8/364	0.018792563	8
GO:0030136	clathrin-coated vesicle	10/364	0.020118264	10
GO:0001518	voltage-gated sodium channel complex	3/364	0.02144704	3
GO:0008021	synaptic vesicle	10/364	0.023437184	10
GO:0098636	protein complex involved in cell adhesion	4/364	0.025309215	4
GO:0005938	cell cortex	13/364	0.025475952	13

GO:0044853	plasma membrane raft	7/364	0.026720144	7
GO:0032589	neuron projection membrane	5/364	0.026720144	5
GO:0031256	leading edge membrane	9/364	0.028876621	9
GO:0030426	growth cone	9/364	0.034186028	9
GO:0099738	cell cortex region	4/364	0.037264131	4
GO:0030662	coated vesicle membrane	9/364	0.037878765	9
GO:0070382	exocytic vesicle	10/364	0.037878765	10
GO:0043209	myelin sheath	4/364	0.039051334	4
GO:0042101	T cell receptor complex	8/364	0.039420801	8
GO:0030133	transport vesicle	15/364	0.044167482	15
GO:0045178	basal part of cell	11/364	0.046350166	11

Supplementary Table 11. KEGG enrichment analysis of interaction mRNA of miRNA-mRNA regulatory network.

ID	Description	GeneRatio	*p*.adjust	Count
hsa04061	Viral protein interaction with cytokine and cytokine receptor	15/183	1.25E-06	15
hsa04640	Hematopoietic cell lineage	14/183	4.47E-06	14
hsa04145	Phagosome	16/183	2.15E-05	16
hsa04060	Cytokine-cytokine receptor interaction	22/183	4.17E-05	22
hsa04650	Natural killer cell mediated cytotoxicity	14/183	5.35E-05	14
hsa04062	Chemokine signaling pathway	17/183	5.35E-05	17
hsa05152	Tuberculosis	16/183	9.02E-05	16
hsa05140	Leishmaniasis	10/183	0.000228964	10
hsa04670	Leukocyte transendothelial migration	12/183	0.000232529	12
hsa05340	Primary immunodeficiency	7/183	0.000426694	7
hsa04613	Neutrophil extracellular trap formation	15/183	0.000498815	15
hsa05033	Nicotine addiction	7/183	0.000505603	7
hsa04380	Osteoclast differentiation	12/183	0.00052506	12
hsa04658	Th1 and Th2 cell differentiation	10/183	0.000641509	10
hsa04662	B cell receptor signaling pathway	9/183	0.001359195	9
hsa04659	Th17 cell differentiation	10/183	0.002206169	10
hsa04727	GABAergic synapse	9/183	0.002271123	9
hsa04666	Fc gamma R-mediated phagocytosis	9/183	0.004130541	9
hsa04940	Type I diabetes mellitus	6/183	0.004473396	6
hsa04660	T cell receptor signaling pathway	9/183	0.006241721	9
hsa05321	Inflammatory bowel disease	7/183	0.006704208	7
hsa04664	Fc epsilon RI signaling pathway	7/183	0.008418546	7
hsa05120	Epithelial cell signaling in Helicobacter pylori infection	7/183	0.009587179	7
hsa05162	Measles	10/183	0.011069821	10
hsa05150	Staphylococcus aureus infection	8/183	0.012950723	8
hsa05134	Legionellosis	6/183	0.014983431	6
hsa04514	Cell adhesion molecules	10/183	0.016608347	10
hsa05169	Epstein-Barr virus infection	12/183	0.016616244	12

hsa05332	Graft-versus-host disease	5/183	0.018911494	5
hsa05417	Lipid and atherosclerosis	12/183	0.02595252	12
hsa04630	JAK-STAT signaling pathway	10/183	0.025973268	10
hsa04540	Gap junction	7/183	0.025973268	7
hsa05235	PD-L1 expression and PD-1 checkpoint pathway in cancer	7/183	0.026826207	7
hsa05032	Morphine addiction	7/183	0.028787971	7
hsa05166	Human T-cell leukemia virus 1 infection	12/183	0.028787971	12
hsa05133	Pertussis	6/183	0.045832147	6
hsa05142	Chagas disease	7/183	0.047903194	7
hsa04612	Antigen processing and presentation	6/183	0.047903194	6
hsa04721	Synaptic vesicle cycle	6/183	0.047903194	6
